# Supplementary material for: Multifaceted interventions for healthcare-associated infections and rational use of antibiotics in a low-to-middle-income country: Can they be sustained?
Source: PLoS One. 2020 Jun 16;15(6):e0234233. doi: 10.1371/journal.pone.0234233 (PMC7297356; doi:10.1371/journal.pone.0234233)
Supplement: S1 Data — (PDF) [file pone.0234233.s001.pdf]

`

No	Date of admissi	Date of birth	MR	Age	cat age	~~sex~~	~~weight~~
1	30-Jun-16	9-May-16	1776439	1	1	F	39
2	23-Aug-16	3-Oct-16	1782098	2	1	M	14
3	14-Aug-16	31-Jul-15	1781063	13	2	F	8,5
4	15-Aug-16	22-Oct-16	1781279	2	1	F	8,5
5	18-Aug-16	26-Oct-00	1776961	190	4	F	39
6	16-Aug-16	11-May-11	1781423	63	3	M	12
7	17-Aug-16	19-Jan-13	1781449	43	2	F	12
8	12-Aug-16	13-Jul-13	1780919	37	2	F	13
9	18-Aug-16	18-Jan-13	1624772	43	2	M	10,1
10	16-Aug-16	13-Jan-16	1781120	7	1	F	7,5
11	14-Aug-16	15-Jan-07	1781064	115	2	M	29
12	5-Aug-16	25-Jun-16	1780123	2	1	M	2,6
13	7-Aug-16	18-Jan-16	1780188	7	1	M	6,5
14	1-Aug-16	20-Mar-16	1769735	5	1	F	6,8
15	6-Aug-16	25-Aug-02	1071374	168	4	M	32
16	3-Aug-16	11-Dec-08	1779885	92	3	F	29
17	28-Jul-16	16-May-10	1681931	74	3	F	13,1
18	19-Jun-16	12-Oct-16	1775273	4	1	F	30
19	18-Jun-16	10-Feb-16	1775262	4	1	F	51
20	25-Jul-16	20-Apr-16	1778787	3	1	M	35
21	1-Jun-16	30-Mar-16	1773512	3	1	F	5
22	24-Jul-16	2-Jul-14	1721004	24	2	F	9,5
23	20-Jul-16	7-Oct-16	1778310	-3	1	F	26
24	23-Jul-16	7-Oct-14	1778584	21	2	M	9,5
25	18-Jul-16	23-Oct-14	1470198	21	2	F	19
26	15-Jul-16	24-Jun-14	1777617	25	2	F	11
27	16-Jul-16	20-May-08	1777770	98	3	M	26
28	23-Jul-16	26-Sep-05	1778558	130	4	F	60
29	22-Jul-16	12-Aug-07	1778449	107	3	F	22
30	26-Jul-16	20-Oct-15	1771209	9	1	F	5,5
31	20-Jul-16	19-Jul-16	1776532	0	1	M	2,9
32	23-Jul-16	31-Oct-16	1778568	3	1	F	7,7
33	26-Jul-16	10-Dec-00	1778794	187	4	M	49
34	28-Jul-16	6-May-16	1742904	2	1	F	5
35	3-Aug-16	5-Dec-10	1779874	68	3	M	25
36	26-Jun-16	24-Jun-06	1775915	120	4	M	46
37	3-Jul-16	17-Feb-13	1776600	41	2	M	12
38	6-Jul-16	28-Jul-15	1776780	12	2	M	10
39	3-Jul-16	26-Apr-16	1776567	3	2	F	3,4
40	4-Jun-16	16-Jun-14	1773762	24	2	F	9,5
41	2-Jun-16	26-Jul-14	1700379	23	2	M	9
42	15-Jun-16	21-Aug-08	1774994	94	3	F	34,8
43	7-Jun-16	13-Apr-02	1774143	170	4	M	40

44	23-Jun-16	19-May-15	1775651	13	2	F	7,3
45	8-Jun-16	28-Dec-10	1771085	66	3	M	14
46	29-May-16	11-Dec-14	1751955	17	2	F	7,5
47	06-Jul-16	1-Jan-14	1667737	30	2	F	8,5
48	07-Jul-16	21-Nov-15	1776838	8	1	F	7,5
49	11-Jul-16	9-Apr-16	1776978	3	1	F	7
50	21-Jun-16	24-Aug-03	1775547	154	4	F	33
51	21-Jun-16	11-Jul-07	1775542	107	3	F	27
52	25-Jun-16	8-Mar-16	1775874	3	1	M	4,2
53	15-Jun-16	27-Feb-13	1774198	40	2	M	13
54	17-Jun-16	30-Jun-15	1770888	12	2	F	8,2
55	23-Jun-16	17-Dec-00	1367505	186	4	M	40
56	22-Jun-16	16-Dec-15	1775640	6	1	M	7,2
57	17-Jun-16	29-Apr-12	1775126	50	2	F	4,9
58	21-Jun-16	13-May-15	1775546	13	2	F	10,3
59	17-Jun-16	16-Aug-11	1635771	58	2	F	8,9
60	06-Jun-16	25-Sep-07	1413961	105	3	F	13
61	18-Jun-16	9-Dec-15	1775239	6	1	M	10,3
62	10-Jun-16	9-Aug-15	1774465	10	1	M	6,7
63	29-Jun-16	19-Sep-98	1219027	213	4	M	50
64	19-Jun-16	27-May-15	1774993	13	2	F	8
65	29-Jun-16	3-Dec-13	1663013	30	3	M	6,6
66	16-Jun-16	21-Aug-07	1775125	106	3	M	22
67	21-Jun-16	24-Nov-09	1450472	79	3	F	20
68	19-Jun-16	16-Nov-14	1707851	19	2	M	6,9
69	21-Jun-16	19-Jan-04	1775531	149	4	M	30
70	25-Apr-16	8-Jan-16	1762526	3	1	M	5,45
71	19-May-16	15-Feb-16	1772013	3	1	M	2,4
72	19-May-16	16-Oct-11	1770779	55	2	F	17
73	07-May-16	12-Jun-14	1770507	23	2	M	11
74	14-May-16	5-Sep-14	1721967	20	2	M	6,7
75	06-May-16	2-Oct-15	1776563	7	1	M	7
76	29-Jul-16	6-Mar-14	1575712	28	2	F	7,5
77	19-Jul-16	12-Nov-15	1774768	8	1	M	6,3
78	18-Jul-16	1-Mar-16	1776155	4	1	F	2,3
79	31-Jul-16	3-Mar-16	1776126	4	1	M	4,2
80	06-Jul-16	6-Apr-16	1776803	3	1	F	5,3
81	09-Jul-16	5-Jan-16	1776937	6	1	F	6,5
82	08-Jul-16	25-Jul-99	1776381	204	4	F	41
83	07-Jul-16	7-Jul-15	1771820	12	2	M	8,2
84	15-Jul-16	6-Jun-15	1773795	13	2	M	33
85	10-Jul-16	26-Oct-00	1776961	189	4	F	40
86	14-Jul-16	15-Jun-12	1735411	49	2	F	10
87	29-Jul-16	29-Dec-15	1777922	7	1	F	7,3
88	15-Jul-16	21-Mar-15	1777397	16	2	M	11
89	28-Jul-16	24-Feb-15	1764506	17	2	F	7,3
90	02-Aug-16	14-Aug-14	1779730	24	2	F	6
91	15-Aug-16	1-Mar-16	1781287	5	1	F	5

92	12-Aug-16	5-Nov-99	1766416	201	4	M	46
93	11-Aug-16	27-May-08	1765369	99	3	M	15
94	09-Aug-16	21-May-16	1780392	3	1	M	4,5
95	13-Aug-16	20-Jun-16	1776532	2	1	M	3,5
96	18-Aug-16	25-Dec-14	1773574	20	2	M	9
97	26-Aug-16	16-Jan-08	1425356	103	3	F	18
98	29-Aug-16	31-Mar-12	1782864	53	2	M	13
99	16-Aug-16	20-May-16	1777475	3	1	M	5,6
100	31-Aug-16	22-Feb-03	1782948	162	4	M	46
101	19-Aug-16	19-Sep-98	1219027	215	4	M	46
102	26-Aug-16	21-Apr-16	1781550	4	1	F	2,8
103	26-Aug-16	18-Jul-16	1782611	1	1	M	3,4
104	22-Aug-16	29-May-16	1781963	3	1	M	3,4
105	23-Aug-16	19-May-16	1780657	3	1	F	2,5
106	27-Aug-16	23-Mar-16	1779429	5	1	M	4,9
107	27-Aug-16	1-Jan-08	1782645	103	3	M	25
108	01-Sep-16	19-Apr-09	1783240	89	3	F	19,5
109	02-Sep-16	17-Jul-16	1783258	2	1	F	4
110	03-Sep-16	30-Jun-16	1781099	3	1	M	3,7
111	04-Sep-16	9-Feb-16	1783430	7	1	F	8
112	05-Sep-16	8-Jul-04	1783631	146	4	F	26
113	07-Sep-16	23-Jul-10	1783924	74	3	M	16
114	07-Sep-16	10-Jan-16	1775924	8	1	M	7
115	08-Sep-16	2-May-16	1784014	4	1	M	26
116	08-Sep-16	7-May-15	1782842	16	2	M	4,2
117	08-Sep-16	17-Dec-03	1784109	153	4	M	52
118	10-Sep-16	23-Aug-05	1784244	133	4	M	36
119	14-May-16	19-Nov-05	1293365	126	4	F	20,5
120	17-May-16	8-Apr-14	1681956	25	2	F	5,9
121	22-May-16	10-May-06	1261728	120	4	F	20
122	04-Jun-16	1-Jun-99	1773791	204	4	F	42
123	07-Jun-16	26-Mar-16	1774150	3	1	M	2,3
124	09-Jun-16	17-Apr-14	1754085	26	2	F	8,5
125	10-Jun-16	20-Apr-16	1742845	2	1	M	2,8
126	10-Jun-16	19-Feb-06	1774437	124	4	F	14,5
127	10-Jun-16	1-Feb-09	1774472	88	3	F	20
128	11-Jun-16	17-Mar-14	1774503	27	2	M	10
129	12-Jun-16	8-Apr-06	1774538	122	4	F	23
130	13-Jun-16	14-Jan-13	1672820	41	2	F	8,6
131	13-Jun-16	26-Apr-16	1776567	2	1	F	3,5
132	14-Jun-16	28-Aug-15	1774756	10	1	F	6,3
133	14-Jun-16	27-Dec-08	1771646	90	3	M	23
134	14-Jun-16	3-Nov-15	1760250	7	1	F	3,6
135	15-Jun-16	10-Feb-09	1721847	88	3	F	26
136	17-Jun-16	10-Jun-15	742031	12	2	M	7,2
137	17-Jun-16	2-Jun-01	1473159	180	4	F	47
138	18-Jun-16	3-May-15	1775253	13	2	F	11
139	19-Jun-16	20-Mar-03	1727536	159	4	M	

140	20-Jun-16	6-Jul-06	1774587	119	3	M	24,5
141	21-Jun-16	1-Dec-02	1774764	162	4	M	33
142	21-Jun-16	25-Apr-02	1742263	170	4	F	48
143	24-Jun-16	29-Apr-16	1760949	2	1	M	32
144	25-Jun-16	12-May-13	1775870	37	2	M	11
145	27-Jun-16	5-May-16	1773653	1	1	M	2,7
146	27-Jun-16	10-Jan-99	1592857	209	4	M	34
147	27-Jun-16	20-Mar-03	1727536	159	4	M	36
148	27-Jun-16	5-Jan-15	1724534	17	2	F	9,1
149	29-Jun-16	4-Apr-01	1706905	182	4	F	
150	29-Jun-16	26-Dec-14	1770809	18	2	M	10
151	29-Jun-16	8-Aug-05	1776276	130	4	M	26,5
152	01-Jul-16	20-Dec-03	1220092	151	4	F	39
153	03-Jul-16	29-Apr-16	1775126	3	1	F	4,6
154	09-Jul-16	7-Mar-15	740165	16	2	M	
155	11-Jul-16	24-Jun-15	1748621	13	2	F	7
156	11-Jul-16	11-Nov-13	1691798	32	2	M	11,5
157	11-Jul-16	4-Apr-01	1706905	183	4	F	34,5
158	11-Jul-16	22-Dec-09	1653715	79	3	F	13,5
159	11-Jul-16	9-Mar-16	1774771	4	1	M	6
160	12-Jul-16	25-Apr-07	1777154	111	3	F	24
161	12-Jul-16	26-May-15	1762673	14	2	M	17
162	12-Jul-16	8-Jun-06	1508727	121	4	M	28
163	13-Jul-16	15-Jun-12	1714361	49	2	F	14,5
164	13-Jul-16	27-May-04	1433967	146	4	M	26
165	13-Jul-16	21-Jun-00	1733493	193	4	F	54
166	14-Jul-16	14-Mar-99	1019541	208	4	M	55
167	14-Jul-16	16-Jul-07	1777605	108	3	M	26
168	14-Jul-16	4-Mar-12	1617381	52	2	M	13
169	14-Jul-16	30-Apr-13	1776536	39	2	M	11
170	14-Jul-16	20-May-16	1777475	2	1	M	4,8
171	15-Jul-16	23-Jan-04	1777696	150	4	M	34
172	15-Jul-16	5-Jun-16	1774019	1	1	M	3
173	18-Jul-16	24-Dec-04	1550969	139	4	F	38
174	18-Jul-16	17-Sep-02	1777869	166	4	M	36
175	18-Jul-16	25-Jun-09	1777189	85	3	F	13
176	18-Jul-16	9-Sep-16	1724211	-2	1	M	33
177	18-Jul-16	17-Mar-14	1774503	28	2	M	9
178	19-Jul-16	27-Nov-11	1778091	56	2	M	32
179	20-Jul-16	8-Feb-08	1769288	101	3	F	24
180	20-Jul-16	12-Jul-12	1778287	48	2	M	23
181	20-Jul-16	25-Apr-02	1742263	171	4	F	47
182	24-Jul-16	15-Jun-14	1778604	25	2	M	11,5
183	25-Jul-16	17-Jan-06	1778456	126	4	M	35
184	25-Jul-16	1-Jan-01	1092970	186	4	F	40
185	25-Jul-16	24-Apr-13	1745021	39	2	M	10
186	25-Jul-16	6-Apr-06	1753781	123	4	F	22
187	27-Jul-16	9-Feb-11	1779011	65	3	M	32

188	27-Jul-16	7-May-16	1778805	2	1	F	5,8
189	27-Jul-16	3-Jan-13	1764343	42	2	F	10,3
190	27-Jul-16	6-Jun-04	1779058	145	4	M	22
191	31-Jul-16	4-Oct-03	1779339	153	4	F	45
192	30-Jul-16	20-Dec-03	1220092	151	4	F	42
193	01-Aug-16	8-Jan-16	1762526	7	1	M	6,7
194	02-Aug-16	2-Dec-00	1779586	188	4	F	60,5
195	03-Aug-16	24-May-16	1779871	3	1	F	5,1
196	05-Aug-16	9-May-16	1778641	3	1	F	4,1
197	05-Aug-16	16-Jun-09	1779949	86	3	M	15,5
198	08-Aug-16	23-May-08	1667053	99	3	F	20
199	08-Aug-16	19-May-03	1084914	159	4	M	19
200	09-Aug-16	2-Jan-09	1778484	91	3	M	24
201	09-Aug-16	11-Sep-14	1698853	23	2	M	9
202	11-Aug-16	3-Dec-11	1780767	56	2	M	17,6
203	12-Aug-16	3-Jul-01	1780217	181	4	F	42
204	12-Aug-16	13-Oct-04	1781025	142	4	F	45
205	12-Aug-16	14-Sep-04	1781027	143	4	F	25
206	12-Aug-16	8-Jul-09	1710458	85	3	M	19
207	14-Aug-16	27-Jul-07	1781082	109	3	F	
208	14-Aug-16	10-Feb-11	1732891	66	3	M	17
209	15-Aug-16	14-Jun-16	1781155	2	1	M	3,7
210	19-Aug-16	25-Apr-02	1742263	172	4	F	49
211	22-Aug-16	6-May-16	1781872	3	1	M	4,7
212	22-Aug-16	12-Apr-16	1781887	4	1	F	3,6
213	22-Aug-16	3-Aug-14	1751357	24	2	M	30
214	22-Aug-16	4-Aug-15	1780314	12	2	F	7,1
215	22-Aug-16	11-Feb-10	1719298	78	3	F	16
216	23-Aug-16	27-May-11	1782139	63	3	M	15
217	23-Aug-16	14-Sep-08	1664228	95	3	M	20
218	24-Aug-16	19-Dec-03	1782155	152	4	M	35
219	24-Aug-16	30-Jan-08	1782323	103	3	F	24
220	25-Aug-16	31-Jan-99	1782496	211	4	F	20
221	25-Aug-16	4-Apr-07	1782349	112	3	M	30
222	27-Aug-16	16-Dec-07	1782646	104	3	M	26
223	29-Aug-16	25-Jan-00	1592956	199	4	M	44
224	29-Aug-16	6-Nov-14	1765780	21	2	M	8,7
225	29-Aug-16	4-Aug-01	1751284	180	4	M	47
226	29-Aug-16	13-Apr-08	1750925	100	3	F	22
227	26-Aug-16	4-Oct-03	1779339	154	4	F	40
228	31-Aug-16	12-May-12	1665219	51	2	F	
229	01-Sep-16	1-Mar-99	1159675	210	4	F	52
230	01-Sep-16	23-Jul-07	1783204	110	3	F	17
231	02-Sep-16	6-Jun-03	1773795	159	4	M	29
232	02-Sep-16	22-Dec-08	1783249	93	3	F	19
233	05-Sep-16	25-Jan-00	1592956	200	4	M	45
234	05-Sep-16	18-Oct-15	1783473	11	1	M	6,8
235	05-Sep-16	21-Mar-16	1783453	6	1	M	6,6

236	05-Sep-16	4-Aug-01	1751284	181	4	M	37
237	07-Sep-16	23-May-08	1667053	100	3	F	20
238	07-Sep-16	13-Sep-14	1766791	24	2	M	16,5
239	07-Sep-16	13-Dec-09	1727748	81	3	M	18
240	11-Sep-16	31-May-00	1784288	196	4	F	34
241	12-Sep-16	1-Dec-15	1784287	9	1	F	7,1
242	13-Sep-16	3-Dec-13	1751177	33	2	M	12
243	14-Sep-16	13-Oct-04	1781025	143	4	F	43
244	28-Aug-16	14-Jul-16	1782661	1	1	M	4,7
245	15-Aug-16	4-Feb-08	1768966	102	3	M	20
246	07-Sep-16	1-Oct-15	1767981	11	1	F	7,6
247	09-Sep-16	2-Feb-08	1622546	103	3	M	23
248	09-Sep-16	22-Feb-11	1734915	67	2	M	16
249	12-Sep-16	28-Oct-15	1784326	11	1	F	8
250	12-Sep-16	7-Jul-09	1784345	86	3	M	18
251	13-Sep-16	9-Jun-10	1481030	75	3	M	23
252	15-Sep-16	6-Nov-15	1784798	10	1	M	8
253	15-Sep-16	13-Sep-15	1784807	12	2	M	9,1
254	18-Sep-16	19-Mar-16	1784968	6	1	F	6,9
255	07-Sep-16	2-Aug-16	1780678	1	1	F	2,6
256	14-Sep-16	18-Aug-07	1784504	109	3	M	23
257	19-Sep-16	29-May-15	1731036	16	2	F	8,3
258	04-Aug-16	2-May-16	1777971	3	1	F	3,5
259	05-Aug-16	9-Dec-14	1780128	20	2	M	8
260	08-Aug-16	7-Jul-16	1780200	1	1	M	2,3
261	11-Aug-16	1-Aug-11	1780769	60	3	F	15,5
262	21-Aug-16	27-Oct-14	1781768	22	2	M	18
263	24-Aug-16	14-Jun-10	1782326	74	3	M	18
264	26-Aug-16	5-Oct-10	1782606	70	3	M	17
265	08-Jul-16	7-Oct-07	1779737	105	3	M	37,5
266	11-Jul-16	17-Jan-09	1777112	90	3	M	16
267	14-Jul-16	7-May-03	1777612	158	4	F	30
268	15-Jul-16	31-Mar-04	1700931	148	4	F	35
269	22-Jul-16	27-Jul-07	1778450	108	3	F	21
270	26-Jul-16	18-Jul-99	1778930	204	4	M	56
271	10-Jun-16	5-Mar-12	737006	51	2	M	20
272	02-Aug-16	29-May-15	1772577	15	2	F	9
273	15-Jun-16	25-Feb-16	1773218	4	1	M	2,7
274	18-Jun-16	22-Oct-12	1775252	44	2	M	20
275	19-Jun-16	27-Aug-15	1751853	10	1	M	7,1
276	20-Jun-16	28-Mar-16	1755529	3	1	F	15
277	23-Jun-16	25-Sep-12	1756452	45	2	M	13
278	02-May-16	17-Feb-16	1770096	3	1	M	5,8
279	12-May-16	4-Dec-15	1769759	5	1	F	3,5
280	11-May-16	8-Oct-15	1768762	7	1	M	6,1
281	03-May-16	24-Jan-05	1769660	136	4	F	27
282	23-Aug-16	22-Sep-15	1782086	11	1	M	14
283	31-Aug-16	21-Feb-16	1770545	6	1	M	5

284	03-Sep-16	17-Jan-16	1782007	8	1	F	3,1
285	04-Sep-16	11-May-09	1783449	88	3	M	25
286	04-Sep-16	7-Mar-03	1610036	162	4	M	20
287	05-Sep-16	26-Dec-14	1770803	21	2	M	10
288	10-Sep-16	5-Apr-03	1080851	161	4	M	52
289	12-Sep-16	3-Feb-16	742646	7	1	F	6,4
290	16-Sep-16	6-Jun-03	1779339	159	4	M	28,5
291	16-Sep-16	23-Apr-06	1784912	125	4	F	45
292	17-Sep-16	7-Mar-15	1784935	18	2	M	11
293	19-Sep-16	5-Aug-12	1638817	49	2	F	11
294	19-Sep-16	26-Jun-03	1680697	159	4	M	34,5
295	19-Sep-16	27-Mar-16	1785054	6	1	M	13
296	19-Sep-16	9-Apr-15	1727708	17	2	M	5,6
297	19-Sep-16	11-May-13	1785162	40	2	F	18
298	19-Sep-16	10-Apr-11	1729229	65	3	F	14
299	20-Sep-16	25-Apr-12	1742263	53	2	F	51
300	20-Sep-16	19-Feb-16	1785314	7	1	M	6,9
301	21-Sep-16	23-Sep-14	1719745	24	2	F	5,7
302	22-Sep-16	15-Jan-16	1777297	8	1	M	5,8
303	24-Sep-16	21-Jul-07	1785682	110	3	F	
304	24-Sep-16	4-Mar-12	1785681	54	2	M	14
305	25-Sep-16	14-Apr-16	1785749	5	1	F	6,8
306	26-Sep-16	23-Aug-16	1785909	1	1	F	3,3
307	27-Sep-16	22-Jan-15	1726221	20	2	F	8
308	05-Sep-16	15-Jun-14	1783538	27	2	M	7,2
309	15-Sep-16	29-Dec-07	1365065	105	3	F	20
310	18-Sep-16	21-Mar-16	1783453	6	1	M	7
311	26-Sep-16	3-Dec-12	1781151	45	3	M	15
312	28-Aug-16	5-Jan-16	1758717	7	1	F	4,4
313	27-Aug-16	5-Apr-16	1782623	4	1	M	6
314	02-Sep-16	15-Mar-16	1783363	6	2	M	4,7
315	16-Sep-16	11-Mar-10	1767291	78	3	F	14
316	21-Sep-16	4-Aug-16	743132	1	1	F	4,5
317	22-Sep-16	20-Jun-16	1776532	3	1	M	4
318	23-Sep-16	1-Jul-16	1784009	2	1	F	4
319	27-Sep-16	8-Jun-12	1723544	51	3	M	14
320	27-Sep-16	17-Jul-14	1723574	26	2	F	9
321	28-Sep-16	14-Sep-08	1664228	96	3	M	20
322	28-Sep-16	19-Oct-03	1786129	155	4	F	42
323	28-Sep-16	25-May-11	1779224	64	3	F	10,5
324	28-Sep-16	25-Apr-02	1742263	173	4	F	51
325	29-Sep-16	30-May-16	1779224	4	1	F	54
326	29-Sep-16	10-Oct-13	1786165	35	2	M	12
327	30-Sep-16	30-Aug-16	1786373	1	1	F	4,4
328	02-Oct-16	24-Apr-06	727795	126	4	M	28
329	02-Oct-16	12-Jan-14	1766043	33	2	M	9
330	02-Oct-16	22-May-08	1786452	101	3	M	20,8
331	03-Oct-16	26-Jun-14	1694641	28	2	M	7,7

332	03-Oct-16	19-Apr-16	1786642	6	1	M	5,1
333	03-Oct-16	30-Jan-08	1782323	105	3	F	21,5
334	04-Oct-16	3-Mar-08	730261	103	3	M	21
335	05-Oct-16	15-Feb-16	1772013	8	1	M	5,4
336	06-Oct-16	5-Apr-99	1799950	210	4	F	36
337	28-Jul-16	18-Mar-16	1772146	4	1	M	18
338	09-Sep-16	9-May-16	1784223	4	1	M	7
339	15-Sep-16	19-Apr-16	1772506	5	1	F	3,9
340	26-Sep-16	14-Jun-16	1781155	3	1	M	4,3
341	29-Sep-16	22-Aug-10	1785769	73	3	M	19
342	01-Oct-16	27-May-16	1786387	5	1	F	3,5
343	02-Oct-16	6-Jun-03	1773795	160	4	M	30
344	05-Oct-16	8-Apr-14	1685302	30	2	M	9,2
345	05-Oct-16	2-May-13	1747900	41	2	M	12,5
346	07-Oct-16	15-Jan-01	1739224	189	4	M	44
347	07-Oct-16	14-Mar-14	1787048	31	2	M	44
348	07-Oct-16	2-Dec-00	1779586	190	4	F	66
349	09-Oct-16	29-Aug-16	1787223	2	1	M	3,5
350	11-Oct-16	15-Mar-02	1544055	175	4	F	40
351	15-Sep-16	27-Jun-16	1784792	3	1	F	4,4
352	28-Sep-16	10-May-11	1786070	64	3	F	14
353	07-Oct-16	13-Apr-16	1778763	6	1	M	6,1
354	09-Oct-16	26-Mar-14	1787222	31	2	M	5,7
355	10-Oct-16	21-May-00	1787435	197	4	F	45
356	10-Oct-16	1-Jun-08	1785846	100	4	M	25
357	10-Oct-16	10-Apr-09	1787394	90	3	M	27
358	11-Oct-16	11-Dec-14	1751955	22	2	F	7,4
359	15-Sep-16	26-Apr-16	1777197	5	1	F	4,4
360	30-Sep-16	17-Jul-16	1786278	2	1	M	3,8
361	01-Oct-16	1-Mar-14	1786392	31	2	F	10
362	01-Oct-16	16-Sep-06	1785310	121	4	M	40
363	09-Oct-16	23-Mar-06	1787201	127	4	F	19
364	12-Oct-16	3-May-05	1787687	137	4	M	28
365	13-Oct-16	29-May-14	1752446	29	2	F	8,3
366	21-Sep-16	19-Aug-00	1785311	193	4	F	35
367	11-Oct-16	16-Jan-01	1787461	189	4	M	15
368	11-Oct-16	3-Nov-06	1280553	119	3	F	16,5
369	09-Oct-16	1-Nov-11	1676010	59	2	M	13
370	30-Aug-16	27-Jul-07	1781082	109	3	F	23
371	06-Oct-16	13-Sep-13	1766791	37	2	M	10,7
372	08-Oct-16	11-Dec-15	1762028	10	1	M	4,5
373	15-Oct-16	25-Mar-07	1787972	115	3	F	26
374	15-Oct-16	5-May-09	1788032	89	3	F	15
375	17-Oct-16	20-Feb-07	1778461	116	3	F	30
376	18-Oct-16	25-Dec-01	1788415	178	4	M	56
377	18-Oct-16	29-Apr-16	1770245	6	1	M	6,9
378	15-Oct-16	15-Oct-07	1788045	108	3	M	25
379	15-Oct-16	2-Aug-99	1080421	206	4	M	53

380	30-Aug-16	27-Mar-02	1782945	173	4	M	47
381	29-Sep-16	24-Sep-13	1745021	36	2	M	10
382	21-Sep-16	12-Jul-16	1785210	2	1	F	2,6
383	29-Sep-16	22-Dec-00	1786149	189	4	F	55
384	04-Oct-16	11-Aug-16	1786779	2	1	F	26
385	07-Oct-16	24-Nov-13	1661740	35	2	M	8,5
386	17-Oct-16	18-Oct-03	1779246	156	4	F	30
387	16-Oct-16	18-Jan-16	1788057	9	1	F	6,9
388	18-Oct-16	31-May-15	1767981	17	2	F	7,5
389	18-Oct-16	6-May-13	1788288	41	2	F	11,4
390	20-Oct-16	14-Sep-08	1664228	97	3	M	22
391	09-Oct-16	19-May-12	1619718	53	2	F	13
392	14-Oct-16	22-Apr-16	1787912	6	1	F	2,6
393	16-Oct-16	23-Oct-07	1764698	108	3	F	17
394	17-Oct-16	5-Jan-12	1750856	57	2	F	12
395	18-Oct-16	26-Apr-10	1787977	78	3	F	15
396	20-Oct-16	21-Feb-14	1673518	32	2	M	13
397	23-Oct-16	11-Mar-10	1767291	79	3	F	16
398	24-Oct-16	5-Feb-06	1755886	128	4	M	25
399	24-Oct-16	3-May-14	1785809	29	2	F	14
400	25-Oct-16	16-Aug-14	1724400	26	2	M	11
401	04-Oct-16	26-Jun-16	1786696	4	1	M	5,8
402	14-Oct-16	21-Apr-16	1775960	6	1	F	4,9
403	18-Oct-16	4-May-00	1757583	197	4	M	55
404	20-Oct-16	26-Jun-16	1786960	4	1	F	6,3
405	19-Oct-16	1-Nov-03	1490012	155	4	F	17,5
406	26-Oct-16	20-Apr-07	1786532	114	3	F	23
407	20-Sep-16	27-Aug-15	1785217	13	2	F	11,5
408	19-Oct-16	9-Feb-15	1788565	20	2	F	9,3
409	12-Oct-16	22-Dec-15	1787556	10	1	M	8,5
410	26-Oct-16	16-Jun-05	1789341	136	4	F	58
411	08-Oct-16	31-May-00	1784288	197	4	F	32
412	27-Oct-16	30-Apr-04	1651516	150	4	M	42
413	19-Oct-16	17-Apr-14	1788543	30	2	M	9,8
414	23-Oct-16	17-Jan-11	1788892	69	3	F	14,5
415	26-Oct-16	29-Mar-03	1657849	163	4	F	15
416	27-Oct-16	14-Sep-08	1664228	97	3	M	22
417	27-Oct-16	26-May-09	1490700	89	3	F	10
418	28-Oct-16	26-Aug-16	743187	2	1	F	2,8
419	30-Oct-16	16-Nov-09	1789677	83	3	F	25
420	30-Oct-16	1-Oct-16	1789673	0	1	M	3
421	31-Oct-16	28-May-10	1784673	77	3	F	17
422	12-Oct-16	9-Sep-13	1724211	37	2	M	38
423	16-Oct-16	28-Jul-00	1019864	195	4	M	52
424	21-Oct-16	15-Mar-10	1526843	79	3	F	13
425	29-Oct-16	17-Aug-00	1735123	194	4	F	27
426	31-Oct-16	12-Aug-07	1789860	110	3	M	34
427	31-Oct-16	9-Jul-07	1755351	111	3	M	21,5

428	31-Oct-16	15-Nov-13	1789724	35	2	M	16
429	02-Nov-16	3-Feb-12	1756345	57	2	F	12
430	03-Nov-16	4-Jul-13	1771178	40	2	M	10,7
431	28-Oct-16	11-Jul-16	1788669	3	1	M	5,7
432	28-Oct-16	17-Jan-14	1669455	33	2	M	8
433	30-Oct-16	17-Apr-14	1753405	30	2	M	7,7
434	03-Nov-16	10-Jun-16	1778334	5	1	M	5,7
435	03-Nov-16	30-Jan-08	1782323	106	3	F	22
436	05-Nov-16	20-Nov-15	1790383	12	2	F	7,8
437	18-Oct-16	11-Dec-14	1748253	22	2	M	9,8
438	29-Oct-16	5-Jan-02	1789580	177	4	M	34
439	02-Nov-16	10-Mar-16	1790119	8	1	M	4,6
440	02-Nov-16	10-Nov-07	1789810	108	3	F	26
441	17-Oct-16	24-Jun-01	1712571	184	4	M	40
442	03-Nov-16	18-Mar-12	1772146	56	3	M	16
443	05-Nov-16	23-Nov-99	1790399	204	4	F	47,5
444	07-Nov-16	9-Jun-06	1780461	125	4	M	26
445	07-Nov-16	28-Jun-15	1773568	17	2	M	10
446	07-Nov-16	21-Sep-09	1790564	86	3	F	21
447	07-Nov-16	7-Jan-04	1790592	154	4	M	36
448	07-Nov-16	25-Aug-12	1790569	51	2	F	15
449	08-Nov-16	26-Jul-13	1790764	40	2	F	12
450	07-Nov-16	5-Apr-15	1756257	19	2	F	6,2
451	10-Nov-16	30-Mar-99	1786290	212	4	F	53
452	03-Nov-16	26-Jul-15	1743063	16	2	F	6,8
453	10-Nov-16	15-Sep-08	1405642	98	3	F	22
454	10-Nov-16	5-Dec-00	1790801	191	4	M	70
455	12-Nov-16	11-Apr-16	1791186	7	1	F	7,5
456	12-Nov-16	7-Feb-12	1791172	57	2	M	20
457	10-Oct-16	31-Jul-16	1787385	3	1	M	5,5
458	15-Oct-16	10-Dec-06	1371537	118	2	M	23,5
459	27-Oct-16	14-Mar-99	1019541	211	4	M	60
460	08-Nov-16	15-Oct-05	1676489	133	4	M	28,5
461	10-Nov-16	22-Jun-03	1624211	161	4	M	44
462	14-Nov-16	18-Apr-16	1781614	7	1	M	7,7
463	14-Nov-16	24-Jun-12	1741695	53	2	F	14,5
464	14-Nov-16	10-Nov-15	1777684	12	2	M	7
465	05-Nov-16	27-Dec-15		11	1	F	7,8
466	18-Oct-16	19-Jul-16	1788251	3	1	M	10
467	02-Nov-16	21-Feb-15	1790120	21	2	M	14
468	03-Nov-16	27-May-04	1433967	150	4	M	27
469	03-Nov-16	13-Nov-15	1790244	12	2	F	7
470	08-Nov-16	24-Jun-16	1790784	5	1	M	5,8
471	14-Nov-16	30-Sep-06	1791371	122	4	M	19,5
472	17-Nov-16	11-Oct-16	1791870	1	1	F	5,4
473	26-Oct-16	29-Nov-15	1787568	11	1	M	8,3
474	31-Oct-16	12-Feb-14	1778181	32	3	F	10,8
475	14-Nov-16	2-Mar-16	1791460	8	1	F	6,2

476	16-Nov-16	1-Jan-13	1791622	46	2	F	14
477	19-Nov-16	21-Feb-14	1714204	33	2	M	12
478	19-Nov-16	12-May-16	1791882	6	1	M	63
479	20-Nov-16	9-May-09	1792083	90	3	F	19
480	01-Oct-16	20-Mar-16	1769735	7	1	F	8,1
481	20-Oct-16	25-Apr-02	1742263	174	4	F	47,5
482	29-Oct-16	6-May-13	1788288	41	2	F	11,4
483	10-Nov-16	22-Jun-14	1709092	29	2	M	8,7
484	14-Nov-16	9-Sep-09	1437377	86	3	M	22
485	15-Nov-16	15-Aug-10	1785841	75	3	M	14
486	17-Nov-16	25-Dec-04	1787186	143	4	F	29
487	20-Nov-16	15-Apr-12	1746761	55	2	F	8,6
488	20-Nov-16	11-May-16	1772633	6	1	M	5,5
489	21-Nov-16	14-Jul-16	1777802	4	1	F	4,3
490	30-Oct-16	2-Jul-05	1788816	135	4	F	30
491	03-Nov-16	15-Nov-15	1788349	12	2	M	6,6
492	10-Nov-16	22-Nov-99	1791021	204	4	F	65
493	13-Nov-16	31-Dec-99	1284751	203	4	F	37
494	15-Nov-16	20-Apr-06	1791595	127	4	M	49
495	15-Nov-16	6-Jun-03	1773795	161	4	M	32
496	19-Nov-16	14-May-10	1792048	78	3	M	20
497	21-Nov-16	17-Oct-12	1676422	49	2	M	11
498	22-Nov-16	5-Aug-11	1792419	63	3	M	15
499	23-Nov-16	12-Sep-16	1792431	2	1	F	4
500	07-Oct-16	9-Aug-16	1787092	2	1	M	2,6
501	21-Nov-16	16-Jul-07	1792193	112	3	M	25
502	21-Nov-16	17-Nov-06	1791271	120	4	M	25
503	23-Nov-16	23-Nov-16	1653943	0	1	F	14
504	24-Nov-16	15-Aug-13	1792242	39	2	M	12
505	24-Nov-16	18-Mar-12	1772146	56	2	M	15
506	26-Nov-16	29-Apr-10	1792820	79	3	M	30
507	22-Nov-16	19-Aug-16	1787674	3	1	M	2,4
508	24-Nov-16	6-Aug-16	1792682	3	1	F	5,4
509	11-Nov-16	17-Jul-15	1791114	16	2	M	5,4
510	22-Nov-16	3-Aug-15	742189	15	2	M	6,5
511	23-Nov-16	5-Aug-15	1752415	15	2	F	5
512	24-Nov-16	21-Sep-16	1791496	2	1	M	4,6
513	26-Nov-16	7-Dec-15	1761856	11	1	F	5,7
514	27-Nov-16	18-Mar-12	1595648	56	2	F	14
515	28-Nov-16	16-Jan-08	1425356	106	3	F	16
516	28-Nov-16	10-Oct-05	1783987	133	4	F	29
517	29-Nov-16	14-Dec-08	1792578	95	3	M	30
518	03-Nov-16	9-Apr-16	1790092	7	1	F	6,9
519	18-Nov-16	13-Mar-99	1791997	212	4	F	41
520	21-Nov-16	30-Jan-14	1675444	34	2	M	10,3
521	22-Nov-16	3-Jun-09	1792428	89	3	M	28
522	28-Nov-16	6-Feb-09	1792623	93	3	F	16
523	02-Dec-16	25-Jan-15	1793399	23	2	F	8,5

524	04-Dec-16	21-Oct-16	1793555	2	1	F	2,7
525	05-Dec-16	1-May-13	1770628	43	2	F	10
526	05-Dec-16	9-Dec-14	1741283	24	2	F	7,5
527	15-Nov-16	28-Apr-16	1791440	7	1	M	7
528	21-Nov-16	11-Jun-16	1789519	5	1	M	3,9
529	05-Dec-16	18-May-07	1793571	115	3	M	28
530	05-Dec-16	5-Dec-00	1793578	192	4	M	62
531	06-Dec-16	23-Mar-16	1793742	9	1	M	7,5
532	07-Dec-16	6-May-15	1766914	19	2	F	8,2
533	07-Dec-16	30-Jan-08	1782323	107	3	F	22
534	08-Dec-16	18-Aug-14	1726583	28	2	F	8,2
535	08-Dec-16	29-Dec-15	1777922	12	2	F	6,8
536	20-Mar-16	18-Aug-10	1489134	67	3	M	14
537	21-Mar-16	2-Aug-16	1755786	-5	1	M	7,4
538	24-Mar-16	24-Feb-14	1765501	25	2	F	5,7
539	24-Mar-16	10-Jul-15	1742998	8	1	F	45
540	25-Mar-16	29-Jun-98	1765535	213	4	M	48
541	26-Mar-16	1-Feb-09	1721847	85	3	F	25
542	01-Apr-16	5-Aug-00	1766052	188	4	F	44
543	02-Apr-16	31-May-15	1762316	11	1	F	6,3
544	03-Apr-16	18-Jun-16	1745383	-2	1	M	10,5
545	04-Apr-16	16-Nov-14	1707851	17	2	M	7,6
546	05-Apr-16	25-Dec-99	1228504	196	4	M	29
547	05-Apr-16	5-Jan-12	1750856	51	3	M	11
548	07-Apr-16	7-Jun-00	1728234	190	4	M	49
549	07-Apr-16	9-Oct-13	1682724	30	2	M	9,8
550	08-Apr-16	5-Sep-14	1721967	19	2	M	6,2
551	08-Apr-16	9-Oct-01	1767349	174	4	F	41
552	08-Apr-16	13-Sep-13	1766791	31	3	M	12
553	09-Apr-16	9-Jan-16	1767374	3	1	F	29,5
554	10-Apr-16	15-Oct-15	1767413	6	1	F	5,2
555	10-Apr-16	25-Oct-01	1766855	174	4	F	47
556	11-Apr-16	27-Aug-13	1662410	32	2	M	11
557	11-Apr-16	27-Jan-03	1739427	159	4	M	35
558	11-Apr-16	9-Dec-13	1667939	28	2	F	10
559	11-Apr-16	4-May-00	1757583	191	4	M	55
560	12-Apr-16	16-Jul-13	1767643	33	2	M	7,8
561	12-Apr-16	21-Nov-04	1338948	137	4	F	28
562	13-Apr-16	26-Jan-03	1767846	159	4	M	35
563	13-Apr-16	20-Apr-03	1099599	156	4	F	28
564	13-Apr-16	12-Feb-02	1761888	170	4	M	32
565	14-Apr-16	11-Jun-02	1768133	166	4	M	51
566	14-Apr-16	3-Dec-14	1726616	16	2	M	10,5
567	16-Apr-16	30-Jun-00	1766435	190	4	M	50
568	18-Apr-16	10-Apr-03	1743281	156	4	M	18
569	18-Apr-16	21-May-05	1409807	131	4	F	55
570	18-Apr-16	9-May-14	1744150	23	2	M	8,1
571	18-Apr-16	28-Oct-12	1712785	42	2	F	9,5

572	18-Apr-16	7-Oct-05	1765672	126	4	M	29
573	19-Apr-16	10-Jul-15	1735374	9	1	M	8
574	19-Apr-16	25-Sep-15	1749302	7	1	M	6,5
575	19-Apr-16	25-Oct-01	1766855	174	4	F	44
576	19-Apr-16	21-Feb-03	1767748	158	4	M	42
577	19-Apr-16	7-Jul-07	1314693	105	3	F	30
578	19-Apr-16	12-Apr-11	1707256	60	3	M	15
579	20-Apr-16	10-Mar-14	1768320	25	2	M	
580	20-Apr-16	11-Jul-12	1768772	45	2	M	7,4
581	20-Apr-16	29-Apr-11	1676286	60	3	M	14
582	20-Apr-16	12-Jun-09	1676005	82	3	M	16
583	21-Apr-16	1-Oct-09	1768936	78	3	M	17
584	21-Apr-16	26-Apr-06	1768810	120	4	F	29
585	22-Apr-16	9-Sep-03	1724211	151	4	M	35
586	22-Apr-16	31-Oct-15	1767981	6	1	F	
587	23-Mar-16	4-Sep-15	1764942	6	1	F	3,7
588	23-Apr-16	11-Nov-09	1721827	77	3	F	25
589	24-Apr-16	14-Apr-01	1769148	180	4	M	89
590	25-Apr-16	8-Oct-15	1768762	6	1	M	5,8
591	25-Apr-16	25-Mar-15	1736926	13	2	F	5,7
592	25-Apr-16	30-Apr-15	1769281	12	2	M	7,9
593	25-Apr-16	29-Jun-15	1736065	10	1	M	8,1
594	25-Apr-16	30-May-02	1730900	167	4	F	31
595	25-Apr-16	9-Dec-04	1733907	136	4	F	39
596	25-Apr-16	27-Jul-10	1741857	69	3	F	18,5
597	25-Apr-16	12-Nov-13	1742146	29	2	F	9,6
598	25-Apr-16	30-Nov-09	1768283	77	3	M	16
599	26-Apr-16	8-Feb-08	1769288	98	3	F	21
600	26-Apr-16	4-Oct-99	1772898	198	4	F	45
601	26-Apr-16	19-Jan-04	1195160	147	4	F	21
602	27-Apr-16	11-Mar-04	1769478	145	4	F	30
603	27-Apr-16	11-Jun-98	1494368	214	4	M	38
604	27-Apr-16	17-Nov-05	1768144	125	4	M	40
605	28-Apr-16	20-Oct-14	1768858	18	2	F	9
606	28-Apr-16	9-Jun-16	1769002	-2	1	M	8
607	28-Apr-16	1-Aug-12	1769701	44	2	F	11
608	29-Apr-16	4-Feb-16	1769847	2	1	M	5,3
609	30-Apr-16	23-May-14	1769883	23	2	M	11
610	30-Apr-16	26-Oct-06	1769867	114	3	M	43
611	30-Apr-16	28-Mar-16	1769870	1	1	M	3,2
612	01-May-16	31-Mar-16	1773002	2	1	F	5
613	02-May-16	20-Jun-15	1770099	11	1	F	6,8
614	02-May-16	25-Jan-16	1769929	4	1	M	5,5
615	02-May-16	28-Nov-12	1767455	42	2	F	13
616	02-May-16	10-Oct-13	1671434	31	2	M	10
617	02-May-16	15-Mar-14	1687926	26	2	M	10
618	02-May-16	26-Feb-16	1770094	3	1	M	4,1
619	02-May-16	20-Dec-03	1220092	149	4	F	40

620	02-May-16	3-Jan-16	1758936	4	1	F	5,2
621	03-May-16	27-Jan-11	1652479	64	3	F	16,3
622	03-May-16	4-May-01	1770050	180	4	F	34
623	04-May-16	11-Aug-11	1727477	57	2	M	20
624	04-May-16	27-Aug-15	1747654	9	1	M	7
625	04-May-16	6-Jun-03	1773795	155	4	M	30
626	04-May-16	21-Jul-01	1729430	178	4	M	25
627	06-May-16	19-May-07	1770493	108	3	M	40
628	07-May-16	12-Feb-15	1721788	15	2	M	6,4
629	07-May-16	11-Oct-16		5	1	F	14
630	07-May-16	19-Mar-16	1766028	2	1	M	3,6
631	08-May-16	21-Feb-16	1770545	3	1	M	4,3
632	09-May-16	16-Jan-08	1425356	100	3	F	18
633	09-May-16	24-Oct-09	1693649	79	3	M	23
634	10-May-16	26-Dec-05	1769640	125	4	F	25
635	10-May-16	30-Mar-16	1770958	2	1	M	47
636	11-May-16	31-May-16	1694004	0	1	M	11,5
637	11-May-16	11-Jun-06	1769377	119	3	F	25
638	12-May-16	12-May-03	1574102	156	4	F	35
639	12-May-16	11-Dec-13	740233	29	2	F	7,8
640	12-May-16	23-Sep-15	1766630	8	1	F	7,5
641	13-May-16	3-Dec-13	1663013	29	2	M	
642	13-May-16	3-Jan-01	1771311	184	4	F	49
643	14-May-16	23-May-08	1667053	96	3	F	20
644	16-May-16	26-Feb-03	1710067	159	4	M	60
645	16-May-16	3-Jan-13	1764343	40	2	F	7,2
646	16-May-16	19-May-01	1768027	180	4	F	41
647	16-May-16	22-Apr-15	1727543	13	2	M	5,8
648	16-May-16	1-Nov-12	1676094	42	2	F	10
649	17-May-16	8-Nov-10	1648489	66	3	M	13,5
650	17-May-16	18-Dec-15	1768409	5	1	M	4,6
651	17-May-16	30-Apr-04	1651516	145	4	M	45
652	18-May-16	20-Jan-14	1231438	28	2	M	40
653	18-May-16	20-Oct-15	1768056	7	1	M	9
654	18-May-16	23-Jun-14	1765649	23	2	F	8
655	18-May-16	17-Aug-00	1735123	189	4	F	27
656	19-May-16	26-Jul-12	1771998	46	2	M	13,4
657	19-May-16	30-May-02	1730900	168	4	F	34
658	20-May-16	20-Apr-10	1772252	73	3	F	17
659	20-May-16	15-Jun-12	1714361	47	2	F	16,5
660	20-May-16	1-Nov-12	1631694	42	2	M	15
661	20-May-16	19-Mar-14	740570	26	2	F	11
662	21-May-16	20-Jul-15	1743063	10	1	F	6,4
663	23-May-16	18-Jan-06	1759707	124	4	M	24
664	23-May-16	2-Apr-13	1640854	37	2	M	15
665	23-May-16	14-Feb-11	1587746	63	3	F	15
666	23-May-16	30-Mar-05	1754548	134	4	F	33
667	24-May-16	23-Sep-15	1772560	8	1	F	8

668	24-May-16	8-Mar-16	1772516	2	1	F	4
669	25-May-16	19-Apr-16	1772645	1	1	F	2,7
670	26-May-16	25-Sep-14	1715451	20	2	M	10
671	26-May-16	15-Feb-14	1743432	27	2	F	8,8
672	26-May-16	8-Feb-08	1769288	99	3	F	24
673	27-May-16	17-Nov-05	1768144	126	4	M	35
674	28-May-16	20-Mar-03	1727536	158	4	M	34
675	29-May-16	6-Mar-11	1773032	62	3	M	15,5
676	30-May-16	18-Feb-16	1714408	3	1	M	36
677	30-May-16	9-Sep-03	1724211	152	4	M	31
678	31-May-16	3-Feb-16	1772346	3	1	M	5,4
679	01-Jun-16	5-Jan-07	1770184	113	3	F	24
680	01-Jun-16	26-Mar-14	1701387	27	2	F	8,6
681	01-Jun-16	21-Nov-09	1597864	79	3	F	13,9
682	01-Jun-16	3-May-12	1724509	49	2	F	13
683	02-Jun-16	19-Jul-16	728101	-1	1	M	22
684	04-Jun-16	15-Jan-16	1773797	5	1	M	8,4
685	05-Jun-16	14-Sep-08	1664228	93	4	M	20
686	06-Jun-16	6-Jun-03	1751254	156	4	F	32,5
687	06-Jun-16	9-Apr-16	1770144	2	1	M	4,4
688	06-Jun-16	26-Jun-08	730717	96	3	F	12
689	06-Jun-16	30-May-02	1730900	169	4	F	35
690	07-Jun-16	18-Jun-15	1774018	12	2	M	8,5
691	07-Jun-16	21-Jan-10	1774118	77	3	M	24
692	07-Jun-16	5-Mar-08	1369304	99	3	F	15
693	07-Jun-16	7-Sep-14	1703140	21	2	F	6,4
694	08-Jun-16	25-May-08	1774264	97	3	M	28
695	08-Jun-16	8-Jun-01	1605942	180	4	F	45
696	08-Jun-16	9-Mar-16	1358985	3	1	F	24
697	10-Jun-16	22-Aug-08	1774478	94	3	M	45
698	12-Jun-16	12-Jun-16	1774546	0	1	M	6,6
699	13-Jun-16	2-Jan-04	1774722	149	4	M	50
700	08-Sep-16	26-May-04	1171629	148	4	M	10
701	03-Nov-16	1-Mar-16	1790249	8	1	M	8
702	10-Nov-16	8-Jan-04	1791051	154	4	M	40
703	14-Nov-16	23-Jul-16	1791260	4	1	M	5
704	21-Nov-16	26-May-14	1764691	30	2	M	9
705	24-Nov-16	18-Jul-08	1792318	100	3	M	22
706	25-Nov-16	10-Mar-16	1790119	8	1	M	4,4
707	30-Nov-16	26-Oct-16	1793278	1	1	F	36
708	01-Dec-16	19-May-12	1619718	55	2	F	8,5
709	02-Dec-16	4-Aug-11	1793408	64	3	M	18
710	06-Dec-16	21-Jun-05	1793747	138	4	M	27
711	09-Dec-16	16-Nov-14	1707851	25	2	M	7,3
712	09-Dec-16	16-Mar-16	1769795	9	1	M	7
713	09-Dec-16	18-Oct-16	1794198	2	1	F	4
714	09-Dec-16	9-Apr-16	1790092	8	1	F	4,8
715	10-Dec-16	21-Jun-06	1794339	126	4	M	40,5

716	10-Dec-16	10-Jun-16	742984	6	1	M	7,6
717	12-Dec-16	8-May-12	1727461	55	2	M	24
718	27-Nov-16	23-Mar-05	1792853	140	4	F	29
719	13-Dec-16	15-Apr-12	1746761	56	2	F	9,8
720	13-Dec-16	13-Apr-00	1330087	200	4	M	66
721	14-Dec-16	25-Dec-04	1787186	144	4	F	30
722	14-Dec-16	11-May-16	1772633	7	1	M	6,2
723	15-Dec-16	2-Nov-02	1794869	169	4	F	36,5
724	15-Dec-16	28-May-15	1785594	19	2	F	6,3
725	16-Dec-16	30-Sep-16	1795001	3	1	F	4
726	16-Dec-16	13-Jul-16	1794999	5	1	M	6,9
727	18-Dec-16	4-Aug-12	737905	52	2	F	20
728	18-Dec-16	2-Nov-12	1795041	49	2	M	15
729	19-Dec-16	19-Feb-16	1766375	10	1	M	5,8
730	19-Dec-16	22-Jun-11	1725815	66	3	F	17
731	19-Dec-16	27-Oct-16	1795076	2	1	F	35
732	19-Dec-16	11-Jun-14	1716789	30	2	F	9
733	19-Dec-16	26-Jan-08	1620991	107	3	F	34
734	19-Dec-16	11-Jan-12	1795079	59	2	M	23
735	20-Dec-16	31-May-07	1793623	115	3	F	27
736	21-Dec-16	21-Dec-14	1760949	24	2	M	36
737	21-Dec-16	1-Aug-07	1795393	112	3	M	36
738	22-Dec-16	12-Jun-11	1759186	66	3	M	14
739	23-Dec-16	17-Sep-07	1795707	111	3	M	25
740	25-Dec-16	3-Oct-09		86	3	M	24
741	25-Dec-16	23-Mar-16	1795955	9	1	M	9
742	25-Dec-16	6-Oct-10	734315	74	3	M	20
743	26-Dec-16	12-Sep-06	1795980	123	4	M	27
744	26-Dec-16	3-Apr-02	1770131	176	4	M	25
745	29-Dec-16	4-May-00	1757583	199	4	M	51
746	29-Dec-16	29-Feb-08	1796065	106	3	M	18
747	29-Dec-16	26-Dec-12	1643022	48	2	M	15,5
748	29-Oct-16	1-Feb-16	1764676	8	1	F	3,5
749	29-Nov-16	6-Jun-03	1773745	161	4	M	30
750	15-Dec-16	1-Aug-04	1794619	148	4	F	40
751	19-Dec-16	31-Oct-16	1795288	2	1	M	6
752	03-Jan-17	25-Aug-13	1684370	41	2	M	13
753	03-Jan-17	12-Jan-14	1673760	36	2	M	10,9
754	24-Sep-16	24-Jul-16	1785673	2	1	F	5,1
755	24-Nov-16	27-Aug-99	1792560	207	4	M	64
756	25-Nov-16	17-Aug-16	1788252	3	1	M	3,9
757	30-Nov-16	21-Jun-16	1793183	5	1	F	6,4
758	01-Dec-16	26-Jul-16	1790633	5	1	M	4
759	02-Dec-16	20-May-14	1788968	31	2	M	11
760	19-Dec-16	13-Nov-16	1795088	1	1	M	4,5
761	14-Dec-16	10-Nov-16	1794627	1	1	M	2,1
762	26-Dec-16	16-Jul-07	1777605	113	3	M	40
763	27-Dec-16	18-Aug-03	1796051	160	4	M	24

764	30-Dec-16	13-Jul-16	1788475	5	1	M	5,2
765	31-Dec-16	18-Mar-16	1460902	9	1	M	18
766	02-Jan-17	14-Aug-04	1165954	149	4	F	15
767	02-Jan-17	23-Feb-16	1796738	11	1	F	7,2
768	03-Jan-17	27-Jun-16	1796855	7	1	M	7,5
769	03-Jan-17	2-Feb-13	1796826	47	2	M	20
770	03-Jan-17	13-Feb-04	1771316	155	4	F	30
771	03-Jan-17	21-Feb-09	1570825	95	3	M	16,5
772	03-Jan-17	29-Nov-11	1760831	62	3	F	43
773	04-Jan-17	1-May-01	1689580	188	4	F	49
774	05-Jan-17	28-Nov-10	1536346	74	3	F	13,5
775	30-Nov-16	17-Mar-16	1793283	8	1	F	4,2
776	20-Dec-16	17-Apr-07	1795293	116	3	M	30
777	05-Jan-17	30-Jan-08	1782323	108	3	F	20
778	06-Jan-17	24-Jun-01	1712571	187	4	M	42
779	06-Jan-17	23-Mar-00	1797132	202	4	M	53
780	07-Jan-17	4-Apr-04	1170520	153	4	M	32
781	09-Jan-17	27-Apr-13	1634959	45	2	M	10
782	09-Jan-17	6-Nov-14	1765780	26	2	M	9,5
783	10-Jan-17	19-Feb-16	1766375	11	1	M	5,6
784	28-Dec-16	10-Jul-06	1632804	125	4	F	14
785	09-Jan-17	16-Jul-08	1796776	102	3	M	16
786	11-Jan-17	26-Dec-12	1721026	49	2	F	8,5
787	23-Dec-16	19-Jul-16	1795904	5	1	F	5,9
788	05-Jan-17	19-May-16	1796996	8	1	M	7,6
789	06-Jan-17	6-Jun-15	1797242	19	2	F	8,9
790	07-Jan-17	29-Dec-11	1797286	61	3	F	19
791	10-Jan-17	19-Apr-16	1772506	9	1	F	6,3
792	13-Jan-17	4-Apr-16	1797915	9	1	M	6,4
793	11-Jan-17	17-Jun-08	1771960	103	3	F	18
794	13-Jan-17	28-Feb-14	1798017	35	2	M	12,5
795	14-Jan-17	24-Jun-15	1798019	19	2	F	10
796	14-Jan-17	1-Apr-03	1798039	165	4	F	64
797	14-Dec-16	20-Jul-16	1784112	5	1	M	5,2
798	15-Dec-16	8-Jan-04	1791051	155	4	M	40
799	28-Dec-16	21-May-16	1787140	7	1	F	5,4
800	03-Jan-17	11-Aug-16	743155	5	1	F	4,7
801	04-Jan-17	7-Mar-17	1796931	2	1	F	23
802	07-Jan-17	27-Mar-03	1797297	166	4	F	53
803	13-Jan-17	21-Nov-16	1798013	2	1	M	2,2
804	16-Jan-17	25-Dec-04	1787186	145	4	F	30,5
805	18-Jan-17	18-May-15	1798524	20	2	M	8,5
806	18-Jan-17	7-Apr-04	1798435	153	4	F	47
807	19-Jan-17	4-Aug-16	1790667	5	1	M	5,3
808	19-Jan-17	25-Feb-07	1798642	119	3	F	20
809	02-Dec-16	30-Mar-02	1793438	177	4	F	28
810	12-Jan-17	9-Apr-08	1797910	105	3	F	18
811	15-Jan-17	26-Nov-14	1798075	26	2	M	10

812	19-Jan-17	16-Jul-14	1709567	30	2	M	11
813	23-Jan-17	19-Jan-07	1727601	120	4	F	21
814	24-Jan-17	27-Apr-13	1709955	45	2	M	15
815	31-Dec-16	7-Oct-16	1789626	2	1	F	6,1
816	08-Jan-17	9-Jul-09	1797331	90	3	F	22
817	18-Jan-17	13-Aug-16	1798512	5	1	M	5,8
818	22-Jan-17	5-Jan-02	1789580	180	4	M	31
819	22-Jan-17	18-Jan-16	1780188	12	2	M	6,5
820	22-Jan-17	7-Oct-01	1798844	183	4	M	66
821	04-Jan-17	19-May-12	1619718	56	2	F	8,5
822	17-Jan-17	25-Nov-06	1798092	122	4	F	38
823	23-Jan-17	16-Jan-16	1797164	12	2	M	4,5
824	23-Jan-17	8-Mar-13	1798837	46	2	F	12
825	24-Jan-17	8-Jun-12	1723544	55	2	M	15
826	24-Jan-17	17-Aug-16	1798841	5	1	M	
827	25-Jan-17	2-Dec-08	1475887	97	3	F	15
828	26-Jan-17	8-Sep-16	1784304	4	1	F	6,7
829	26-Jan-17	1-Oct-00	1799351	195	4	F	40
830	27-Dec-16	19-May-16	1796224	7	1	M	6,8
831	24-Jan-17	14-May-01	1799035	188	4	F	41,5
832	26-Jan-17	6-Mar-06	1799425	130	4	F	28
833	28-Jan-17	23-Mar-05	1799558	142	4	M	91,7
834	19-Jan-17	17-Oct-14	1798505	27	2	F	10
835	26-Jan-17	5-Jan-17	1799297	0	1	F	15
836	29-Jan-17	19-Jul-16	1799629	6	1	M	7,2
837	26-Jan-17	25-Nov-03	1156393	158	4	M	20
838	27-Jan-17	28-Dec-00	1761449	193	4	M	42
839	28-Jan-17	8-Nov-04	1792782	146	4	M	27,5
840	30-Jan-17	9-Jul-09	1797331	90	3	F	20,5
841	25-Dec-16	5-Feb-16	1795948	10	1	M	9
842	06-Jan-17	4-Dec-16	1796883	1	1	F	2,8
843	20-Jan-17	5-May-15	1798756	20	2	M	5,8
844	24-Jan-17	1-Dec-09	1797072	85	3	M	19
845	27-Jan-17	9-Aug-16	1799553	5	1	M	6,9
846	30-Jan-17	19-Apr-16	1772506	9	1	F	5,8
847	26-Jan-17	27-Jun-03	1799352	163	4	F	37,5
848	02-Feb-17	15-Oct-05	1792170	136	4	M	39
849	02-Feb-17	30-Jan-08	1782323	109	3	F	21
850	04-Feb-17	20-Oct-04	1800343	148	4	M	40,5
851	06-Feb-17	31-Dec-99	1284751	206	4	F	35,5
852	05-Feb-17	26-Dec-15	1800416	14	2	F	8,5
853	24-Jan-17	17-Jul-16	1798226	6	2	F	5,5
854	26-Jan-17	26-Oct-15	1768058	15	3	M	9,1
855	31-Jan-17	13-Apr-12	1765194	57	4	M	16
856	05-Feb-17	29-Aug-16	1800377	6	2	F	7
857	06-Feb-17	9-Apr-16	1790092	10	1	F	5,6
858	08-Feb-17	25-Oct-03	1219104	160	4	M	29
859	13-Jan-17	7-Nov-16	1797923	2	1	M	4,3

860	26-Jan-17	22-Jan-15	1799314	24	2	M	50
861	01-Feb-17	1-Mar-15	1779900	23	2	F	8
862	02-Feb-17	11-Mar-10	1767291	83	3	F	16
863	07-Feb-17	6-Sep-03	1724211	161	4	M	35
864	10-Feb-17	30-Mar-02	1793438	179	4	F	27
865	05-Feb-17	9-Mar-07	1800396	119	3	F	28
866	06-Feb-17	9-Aug-16	1785923	6	1	M	5,8
867	12-Feb-17	3-Feb-16	1772346	12	2	M	7
868	24-Jan-17	3-Nov-15	1799155	14	2	F	10,5
869	01-Feb-17	25-Jul-16	1799983	7	1	F	3,6
870	10-Feb-17	29-Sep-99	1800391	209	4	F	
871	08-Feb-17	15-Jun-14	1783538	32	2	M	7,5
872	08-Feb-17	25-Feb-15	1796373	24	2	M	8,3
873	13-Feb-17	9-Jul-09	1797331	91	3	F	23
874	16-Feb-17	17-Oct-11	1726194	64	3	F	15
875	24-Dec-16	31-Oct-05	1366485	134	4	F	16
876	31-Jan-17	28-Apr-16	742879	9	1	M	8,9
877	26-Jan-17	5-Feb-00	1799319	203	4	M	31
878	08-Feb-17	11-Aug-13	1656140	42	2	M	13
879	12-Feb-17	15-Mar-11	1801115	71	3	F	20
880	13-Feb-17	9-Sep-01	1801433	185	4	M	45
881	16-Feb-17	25-Dec-04	1787186	146	4	F	30
882	23-Jan-17	20-May-07	1798937	116	3	F	20
883	31-Jan-17	5-Sep-14	1721967	28	2	M	6,7
884	17-Feb-17	18-May-15	1801823	21	2	M	9,8
885	28-Dec-16	6-May-16	1781872	7	1	M	4,5
886	27-Jan-17	2-Nov-16	1798740	2	1	F	4,3
887	07-Feb-17	22-Dec-16	1798397	2	1	M	2,4
888	10-Feb-17	5-Feb-16	1795948	12	2	M	6,5
889	16-Feb-17	24-Jul-09	1761143	91	3	F	14
890	16-Feb-17	16-Feb-09	1801644	96	3	M	20
891	18-Feb-17	26-Nov-05	1801967	135	4	F	32
892	18-Feb-17	22-Sep-15	1801971	17	2	F	7,5
893	18-Feb-17	2-Mar-99	1799368	215	4	F	35
894	20-Feb-17	26-Apr-10	1787977	82	3	F	17
895	20-Feb-17	3-Jun-14	1725639	32	2	M	10
896	20-Feb-17	7-Apr-99	1761720	214	4	M	36
897	21-Feb-17	11-Jan-09	1761837	97	3	M	23
898	10-Jan-17	25-Sep-16	1797405	4	1	M	2,7
899	05-Feb-17	7-Nov-05	1800406	135	4	F	25
900	07-Feb-17	26-Jun-03	1800731	164	4	M	30
901	08-Feb-17	5-Mar-07	1800884	119	3	M	23
902	10-Feb-17	2-May-12	1801092	57	2	M	25
903	15-Feb-17	1-Aug-03	1801609	162	4	M	26
904	17-Feb-17	11-Dec-13	740233	38	2	F	9,6
905	18-Feb-17	17-Aug-12	1801953	54	2	F	10
906	18-Feb-17	26-Oct-11	1717449	64	3	F	20
907	18-Feb-17	25-Dec-16	1795972	2	1	M	3,1

908	19-Feb-17	22-Sep-16	1789479	5	1	F	4,5
909	21-Feb-17	7-Aug-01	1798291	186	4	M	45,5
910	21-Feb-17	27-Apr-16	1802293	10	1	F	7
911	21-Feb-17	11-Sep-15	1750529	17	2	M	8,2
912	22-Feb-17	19-Jun-16	733769	8	1	M	22
913	22-Feb-17	4-May-00	1757583	201	4	M	51
914	22-Feb-17	28-Jun-10	1631987	80	3	M	17,5
915	22-Feb-17	20-Apr-16	742845	10	1	M	8,2
916	23-Feb-17	7-Jun-16	1802440	8	1	F	6,8
917	23-Feb-17	11-Mar-10	1767291	83	3	F	16
918	24-Feb-17	27-Mar-99	1802684	215	4	M	62
919	27-Feb-17	22-Aug-16	1791295	6	1	F	4,8
920	27-Feb-17	6-Mar-06	1799425	131	4	M	22
921	28-Feb-17	26-Aug-16	743187	6	1	M	7
922	28-Feb-17	17-Aug-16	1802946	6	1	F	5,3
923	01-Mar-17	12-Feb-05	1290092	145	4	F	28
924	01-Mar-17	1-Feb-16	1803195	13	2	M	5,6
925	02-Mar-17	13-May-05	1314128	142	4	M	27
926	02-Mar-17	8-Feb-08	1769288	109	3	F	25
927	02-Mar-17	17-Oct-12	1803313	53	2	M	17,5
928	02-Mar-17	3-Apr-10	1717062	83	3	M	16
929	02-Mar-17	11-Jan-12	1795079	62	3	M	20
930	06-Mar-17	10-Dec-13	1704092	39	2	F	10
931	06-Mar-17	18-Feb-15	1731783	25	2	F	9,1
932	06-Mar-17	10-Sep-12	1792584	54	2	F	13
933	06-Mar-17	11-Aug-09	1704090	91	3	F	20
934	18-Feb-17	12-Feb-16	1801630	12	2	F	9
935	02-Mar-17	27-Feb-06	1439097	133	4	F	19
936	03-Mar-17	20-Feb-12	1803320	61	3	F	13
937	06-Mar-17	21-Dec-16	1800578	3	1	M	2,2
938	06-Mar-17	31-Oct-08	1765267	101	3	F	16,5
939	22-Feb-17	18-Dec-16	1802403	2	1	F	4,1
940	06-Mar-17	1-Jul-11	1803559	68	3	M	13
941	08-Mar-17	14-Apr-01	1215316	191	4	M	60
942	10-Mar-17	30-Mar-02	1793438	180	4	F	30
943	03-Mar-17	28-Jun-16	1795545	9	1	F	6
944	09-Mar-17	15-Apr-11	1804060	71	3	F	14
945	13-Mar-17	5-Mar-12	1803504	60	3	F	12
946	13-Mar-17	13-Jun-12	1696630	57	2	M	15,5
947	14-Mar-17	25-Dec-04	1787186	147	4	F	30
948	19-Jan-17	5-Jan-14	1761913	36	2	M	9,4
949	19-Jan-17	17-Jul-15	1791114	18	2	M	5,7
950	13-Feb-17	5-Aug-16	1801442	6	1	M	6,7
951	23-Feb-17	1-Apr-16	1792550	10	1	F	22
952	23-Feb-17	3-Aug-16	1782128	6	1	F	5,6
953	04-Mar-17	14-Aug-16	1788133	7	1	M	4,8
954	10-Mar-17	3-Oct-13	1775933	41	2	M	9,3
955	13-Mar-17	14-Aug-04	1803216	151	4	M	23,5

956	17-Mar-17	16-Jul-07	1777605	116	3	M	34
957	08-Mar-17	7-Aug-15	1788096	19	2	F	8,3
958	15-Mar-17	9-Apr-04	1802957	155	4	F	32
959	16-Mar-17	6-Dec-16	1804890	3	1	F	5,1
960	16-Mar-17	14-Feb-15	1745034	25	3	M	7
961	17-Mar-17	2-Feb-17	1804005	1	1	F	4,3
962	17-Mar-17	25-Dec-16	1804902	3	1	M	3,6
963	18-Mar-17	14-Dec-15	1805019	15	2	F	9,7
964	20-Mar-17	2-Mar-99	1799368	216	4	F	35
965	20-Mar-17	25-Oct-10	1621100	77	2	F	12
966	21-Mar-17	20-Mar-03	1727536	168	4	M	41
967	21-Mar-17	6-Feb-17	1801167	1	1	F	2,7
968	21-Mar-17	20-Apr-99	1138361	215	4	M	27
969	08-Mar-17	7-Apr-04	1803833	155	4	F	37
970	09-Mar-17	4-Mar-04	1804089	156	4	F	22
971	16-Mar-17	3-Nov-00	1804767	196	4	F	50
972	18-Mar-17	19-Jun-15	1805034	21	2	M	11
973	21-Mar-17	18-Jul-14	1805413	32	2	F	13
974	21-Mar-17	27-Jun-03	1799352	165	4	F	37,5
975	22-Mar-17	1-Mar-00	1805545	204	4	M	54
976	25-Mar-17	22-Oct-16	1805821	5	1	M	5
977	25-Mar-17	20-Jun-08	1646600	105	3	F	26
978	27-Mar-17	26-Jun-16	1786960	9	1	F	6,9
979	20-Mar-17	3-Mar-06	1805198	132	4	F	39
980	22-Mar-17	6-Jun-12	1709761	57	2	M	10
981	26-Mar-17	12-Feb-12	1805851	61	3	M	18
982	09-Mar-17	28-Dec-16	1802317	3	1	F	4,3
983	10-Mar-17	25-Dec-16	1795972	3	1	M	3,6
984	13-Mar-17	1-Oct-06	1805631	125	4	F	38
985	24-Mar-17	6-Jun-03	1751254	165	4	F	36
986	25-Mar-17	10-Aug-16	1805833	7	1	M	7,9
987	27-Mar-17	19-Mar-16	1795229	12	2	F	6,5
988	29-Mar-17	16-Oct-14	1723205	29	2	M	10
989	29-Mar-17	1-May-04	1153593	154	4	F	48
990	06-Mar-17	28-Oct-15	1803489	17	2	F	9
991	08-Mar-17	22-Sep-16	1789479	6	1	F	4.7
992	13-Mar-17	16-Oct-16	1801711	5	1	M	6.4
993	14-Mar-17	29-Jun-99	1804631	213	4	F	42
994	24-Mar-17	25-Jun-16	1777700	9	1	F	4
995	24-Mar-17	4-May-16	1790267	10	1	F	7.5
996	27-Mar-17	26-Jul-15	1743063	20	2	F	7.3
997	29-Mar-17	13-Feb-16	1775306	13	2	F	5.8
998	29-Mar-17	8-Nov-04	1792782	148	4	M	27
999	29-Mar-17	28-Mar-09	1806087	96	3	F	29
1000	29-Mar-17	4-Sep-16	1793254	6	1	M	4,8
1001	30-Mar-17	17-Apr-15	1753408	23	2	M	7
1002	01-Apr-17	8-Sep-16	1806454	7	1	M	6
1003	01-Apr-17	30-Oct-16	1806474	6	1	M	7,6

1004	02-Apr-17	20-Apr-12	1806501	60	3	M	18
1005	02-Apr-17	28-Nov-16	1806486	5	1	M	6
1006	03-Apr-17	11-Jan-12	1793079	63	3	M	18
1007	03-Apr-17	9-Mar-03	1628231	169	4	M	35
1008	03-Apr-17	15-Jan-15	1806686	27	2	F	9
1009	03-Apr-17	19-May-08	1774749	107	3	F	27
1010	03-Apr-17	6-Jun-13	1668880	46	2	M	15
1011	04-Apr-17	20-Sep-16	1806701	7	1	F	6
1012	05-Apr-17	19-May-12	1619718	59	2	F	7,5
1013	05-Apr-17	7-Mar-07	1806958	121	4	F	17
1014	06-Apr-17	18-May-16	1783060	11	1	M	7,1
1015	07-Apr-17	10-Mar-17	1806370	1	1	M	4,7
1016	08-Apr-17	27-Mar-06	1799891	133	4	F	33
1017	08-Apr-17	1-May-11	1807203	71	3	F	17
1018	10-Apr-17	30-Mar-02	1793438	181	4	F	32
1019	10-Apr-17	20-Dec-03	1220092	160	4	F	32,5
1020	11-Apr-17	3-Jun-14	1807437	34	2	M	10
1021	07-Apr-17	11-Aug-16	1781034	8	1	F	6,1
1022	11-Apr-17	15-Jul-01	1751828	189	4	F	40
1023	12-Apr-17	21-May-03	1807588	167	4	F	
1024	15-Apr-17	18-Jul-14	1805413	33	2	F	13
1025	15-Apr-17	26-May-02	1673087	179	4	M	56
1026	17-Apr-17	3-Oct-00	1804767	198	4	F	42
1027	17-Apr-17	2-Mar-99	1799368	217	4	F	37
1028	17-Apr-17	31-Oct-05	1366485	138	4	F	15,5
1029	17-Mar-17	16-Jan-17	1803235	2	1	M	4,2
1030	17-Apr-17	10-Jun-14	1767076	34	2	M	8,8
1031	18-Apr-17	15-Mar-17	1808357	1	1	M	3,3
1032	19-Apr-17	8-Apr-11	1804683	72	3	F	14
1033	21-Mar-17	13-Dec-16	1805130	3	1	M	4
1034	22-Mar-17	18-Jan-16	1805417	14	2	F	6,9
1035	22-Apr-17	25-Sep-02	1160713	175	4	M	55
1036	24-Mar-17	7-May-17	1782842	-2	1	M	4,8
1037	03-Apr-17	29-Dec-15	1776344	16	2	M	9,9
1038	06-Apr-17	28-Nov-16	1806943	5	1	F	6,5
1039	11-Apr-17	8-Feb-16	1791021	14	2	F	7
1040	11-Apr-17	31-Jan-16	1793198	15	2	M	9,4
1041	11-Apr-17	31-Jan-17	1807563	3	1	M	5
1042	11-Apr-17	5-Mar-17	1807553	1	1	M	4,3
1043	11-Apr-17	20-Oct-13	1807574	42	2	M	13
1044	15-Apr-17	10-Feb-07	1807929	122	4	F	28
1045	21-Apr-17	6-Dec-10	1808314	76	2	F	15
1046	27-Apr-17	25-Nov-17	1809268	7	1	F	26
1047	11-Feb-17	29-Aug-16	1801165	6	1	M	6,8
1048	03-Apr-17		1806474	1407		M	7,6
1049	14-Apr-17	22-Jun-16	1807874	10	1	M	6,4
1050	21-Apr-17	25-Apr-02	1808027	180	4	F	42,5
1051	25-Apr-17	18-Aug-03	1796051	164	4	M	23

1052	26-Apr-17	12-Apr-11	1809012	72	3	F	15
1053	26-Apr-17	26-Apr-14	1685509	36	2	M	12
1054	26-Apr-17	1-Jul-08	1797705	105	3	F	17
1055	26-Apr-17	15-May-00	1726849	203	4	F	36
1056	26-Apr-17	27-Feb-14	1752787	38	2	M	9,2
1057	27-Apr-17	2-Nov-16	1806526	5	1	F	3,8
1058	27-Apr-17	19-Jan-16	1809166	15	2	F	6,1
1059	28-Apr-17	10-May-11	1809402	71	3	F	19
1060	30-Apr-17	31-Oct-05	1366485	138	4	F	21
1061	01-May-17	1-Jul-07	1809482	118	3	F	29
1062	02-May-17	26-Jun-05	1809321	143	4	F	25
1063	03-May-17	30-Jan-08	1782323	112	3	F	22
1064	03-May-17	2-Nov-16	1806526	6	1	F	3,5
1065	03-May-17	1-Jul-06	1809682	130	4	M	47
1066	04-May-17	13-Feb-17	1802783	3	1	F	3,9
1067	05-May-17	17-May-00	1703424	204	4	F	33
1068	15-Mar-15	1-Mar-15	1779900	0	1	F	8,1
1069	22-Mar-17	14-Aug-16	1788133	7	1	M	4,9
1070	30-Apr-17	20-Jul-09	1808943	93	3	F	7,9
1071	08-May-17	3-Jul-09	1804587	94	3	F	24
1072	09-May-17	23-Mar-17	1807179	2	1	F	2,3
1073	09-May-17	8-Jun-12	1723544	59	2	M	15
1074	19-Apr-17	13-Feb-17	1808368	2	1	F	3,5
1075	20-Apr-17	25-Jun-16	1808490	10	1	M	8,7
1076	23-Apr-17	25-Dec-16	1795972	4	1	M	4,2
1077	25-Apr-17	8-Oct-16	1795506	6	1	M	6,4
1078	25-Apr-17	10-Aug-16	1792567	8	1	M	7,5
1079	01-May-17	13-Jan-17	1809500	4	1	F	4,5
1080	02-May-02	9-Dec-02	1809521	7	1	M	45
1081	02-May-17	8-Nov-04	1504192	150	4	M	23
1082	03-May-17	19-Mar-07	1659806	122	4	F	26
1083	06-May-17	18-Jan-17	1810139	4	1	M	5,4
1084	08-May-17	18-Jan-16	1805417	16	2	F	5,8
1085	08-May-17	17-May-14	1727756	36	2	M	13
1086	10-May-17	18-Sep-16	1810383	8	1	M	5,3
1087	10-May-17	18-Mar-12	1712146	62	3	M	17
1088	11-May-17	30-Sep-09	1443376	92	3	F	15
1089	12-May-17	2-Jun-01	1473159	191	4	F	55
1090	12-May-17	19-Feb-11	1810765	75	3	F	15
1091	14-May-17	31-Mar-15	1809853	26	2	F	8,1
1092	15-May-17	26-Dec-13	1770618	41	2	M	13
1093	15-May-17	3-Oct-00	1804767	199	4	F	43
1094	16-May-17	2-Mar-99	1799368	218	4	F	37
1095	18-May-17	14-Nov-08	1809395	102	3	F	31
1096	18-May-17	26-Aug-09	1810201	93	3	F	26
1097	08-May-17	1-Jul-03	1810308	166	4	F	43
1098	21-May-17	2-Sep-08	1810294	104	3	F	28
1099	15-May-17	5-Jun-10	1811062	83	3	F	19

1100	17-May-17	25-Apr-02	1742263	181	4	F	48
1101	22-May-17	27-Dec-16	1811841	5	1	F	5.5
1102	23-May-17	16-Jan-04	1797551	160	4	F	20
1103	23-May-17	31-Dec-06	1455663	125	4	F	28
1104	24-May-17	28-Jun-10	1799101	83	3	F	15
1105	28-May-17	20-Jun-99	1812504	215	4	F	43
1106	19-Apr-17	1-Apr-04	1808081	156	4	F	24
1107	12-May-17	20-Apr-00	1810649	205	4	F	36
1108	13-May-17	6-Dec-10	1808314	77	3	F	14,5
1109	15-May-17	8-Jul-12	1613623	58	2	M	15
1110	15-May-17	9-Aug-14	1709225	33	2	M	10,5
1111	21-May-17	26-Apr-09	1811809	97	3	M	20
1112	24-May-17	14-Jul-16	1806560	10	1	F	5,5
1113	26-May-17	31-Oct-05	1366485	139	4	F	20
1114	26-May-17	25-Apr-02	1808029	181	4	F	51
1115	29-May-17	6-Jul-02	1103368	178	4	M	71
1116	30-May-17	20-Apr-17	1812839	1	1	M	3,8
1117	31-May-17	30-Mar-02	1793438	182	4	F	38,5
1118	31-May-17	27-Sep-15	1801971	20	2	F	9
1119	31-May-17	26-Jun-05	1809321	143	4	F	26
1120	30-Mar-17	6-May-00	1806342	202	4	F	40
1121	01-May-17	24-Jan-16	1809501	16	2	F	14
1122	02-May-17	28-Aug-16	743194	9	1	F	6,5
1123	04-May-17	13-Jul-16	1788475	10	1	M	4,2
1124	18-May-17	9-Aug-10	1811606	81	3	M	20
1125	10-May-17	28-Oct-15	1803489	19	2	F	8,7
1126	11-May-17	17-May-16	1810644	12	2	M	9,2
1127	14-May-17	26-Nov-16	1793734	6	1	F	3,5
1128	15-May-17	28-Jul-99	1776881	214	4	F	49
1129	15-May-17	21-Jan-09	1748118	100	3	F	14
1130	15-May-17	18-Apr-16	1781614	13	2	M	8,8
1131	17-May-17	1-Mar-07	1770895	122	4	M	27
1132	18-May-17	29-Dec-07	1365065	113	3	F	23
1133	19-May-17	22-Nov-08	1811640	102	3	F	14,5
1134	21-May-17	21-Sep-16	1811805	8	1	M	10
1135	25-May-17	17-Apr-05	1811871	145	4	F	29
1136	30-May-17	3-Oct-12	1775933	55	2	M	9,6
1137	01-Jun-17	24-Jul-09	1802700	95	3	F	14
1138	04-Jun-17	29-Apr-16	1813140	14	2	F	8,3
1139	05-Jun-17	8-Feb-08	1769298	112	3	F	24
1140	07-Apr-16	29-Jun-11	1537269	58	2	M	13,5
1141	05-May-16	13-Jul-15	1770438	10	1	F	9,1
1142	05-May-16	27-Jul-14	1770430	22	2	F	8,2
1143	08-May-16	17-Sep-16	1770557	-4	1	M	15
1144	08-May-16	4-Jul-15	1763826	10	1	M	7,3
1145	10-May-16	26-Dec-14	1770803	17	2	M	9,7
1146	12-May-16	23-Jul-09	1771136	82	3	F	22
1147	12-May-16	2-Jul-05	1770971	130	4	M	29

1148	14-May-16	5-Mar-07	1314994	110	3	F	27
1149	20-May-16	3-Sep-02	1772244	164	4	M	56
1150	20-May-16	1-Dec-15	742491	5	1	M	5
1151	21-May-16	5-Jun-16	1772284	-1	1	M	9
1152	23-May-16	16-May-07	1772499	108	3	F	35
1153	25-May-16	28-May-16	1772732	0	1	F	27
1154	25-May-16	7-Apr-15	1772647	13	2	M	7,7
1155	28-May-16	10-Dec-00	1773020	185	4	M	76
1156	11-Jun-16	9-Aug-11	1774521	58	3	M	18
1157	08-Jun-16	24-Apr-12	1774269	50	3	M	15
1158	12-Jun-16	13-Jul-15	1774550	11	1	F	8,5
1159	08-May-17	28-Aug-16	743194	9	1	F	6,5
1160	24-May-17	22-Dec-02	1758854	173	4	M	35
1161	24-May-17	7-Apr-08	1345667	109	3	F	14
1162	26-May-17	5-Apr-05	1197118	145	4	M	20
1163	30-May-17	5-Apr-04	1702822	157	4	F	54
1164	01-Jun-17	9-Sep-03	1724211	165	4	M	36
1165	05-Jun-17	10-Feb-17	1807275	4	1	F	2,4
1166	06-Jun-17	26-May-15	1740262	25	2	F	10
1167	06-Jun-17	30-Mar-08	1658018	111	3	M	30
1168	07-Jun-17	11-Aug-16	1804923	10	1	M	6,7
1169	07-Jun-17	2-Jan-16	1806405	17	2	F	5,6
1170	12-Jun-17	2-Mar-99	1799368	219	4	F	39
1171	12-Jun-17	3-Oct-00	1804767	200	4	F	39
1172	12-Jun-17	23-May-17	1667053	1	1	F	22
1173	13-Jun-17	12-Jun-10	1813641	84	3	M	19
1174	14-Jun-17	4-May-10	1663524	85	3	M	34
1175	15-Jun-17	10-Apr-08	1360837	110	3	F	17
1176	27-May-17	26-Oct-17	1603095	5	1	M	38
1177	05-Jun-17	13-Feb-17	1471316	4	1	F	30
1178	07-Jun-17	10-Jul-15	1744850	23	2	M	7,2
1179	08-Jun-17	20-Sep-16	1806701	9	1	F	6,4
1180	08-Jun-17	27-Feb-12	1813710	64	3	F	15
1181	08-Jun-17	26-Apr-09	1813721	98	3	F	20
1182	08-Jun-17	4-May-17	1813718	1	1	M	3
1183	09-Jun-17	5-Jul-14	1813815	35	2	M	10,5
1184	11-Jun-17	16-May-16	1809396	13	2	M	6,2
1185	12-Jun-17	22-Jan-17	1813915	5	1	F	3,5
1186	19-Jun-17	6-Feb-17	1806387	4	1	M	2,5
1187	28-Jun-17	15-Apr-05	1197110	146	4	M	22
1188	02-Jun-17	9-Aug-04	1813078	154	4	F	30
1189	03-Jul-17	1-Jul-10	1696688	84	3	F	14,5
1190	03-Jul-17	22-Feb-06	1761733	137	4	F	18,5
1191	03-Jul-17	3-Jun-14	1776341	37	1	M	10
1192	03-Jul-17	8-Jun-02	1796734	181	4	F	52,5
1193	04-Jul-17	23-Feb-06	1802429	137	4	M	60
1194	04-Jul-17	25-Sep-02	1160713	178	4	M	53
1195	05-Jul-17	3-Jul-01	1780217	192	4	F	36

1196	05-Jul-17	5-Apr-05	1776363	147	4	M	45
1197	05-Jul-17	10-Aug-16	1805941	11	1	F	7
1198	05-Jul-17	19-May-01	1768027	194	4	F	40
1199	06-Jul-17	1-Mar-17	1809373	4	1	M	5,5
1200	06-Jul-17	21-Apr-01	1812067	195	4	F	45
1201	06-Jul-17	17-Aug-00	1735123	203	4	F	27
1202	08-Jul-17	7-Oct-14	1816339	33	2	M	11
1203	08-Jul-17	13-Jul-14	1815765	36	2	M	15
1204	10-Jul-17	3-Oct-00	1804767	201	4	F	39
1205	10-Jul-17	4-Jun-17	1813146	1	1	F	3
1206	10-Jul-17	30-Sep-13	1657530	46	2	M	14
1207	10-Jul-17	29-Dec-15	1777922	19	2	F	7
1208	10-Jul-17	24-Dec-12	1745721	55	2	M	18
1209	10-Jul-17	30-Mar-04	1816480	160	4	M	29
1210	10-Jul-17	2-Mar-99	1799368	220	4	F	40
1211	11-Jul-17	10-May-04	1816725	158	4	M	16
1212	12-Jun-17	5-Oct-05	1470856	140	4	F	27
1213	11-Jul-17	5-Mar-16	1783977	16	2	F	5,7
1214	11-Jul-17	24-Sep-06	1807734	130	4	M	17
1215	13-Jul-17	31-Oct-05	1366485	141	4	F	20
1216	13-Jul-17	7-Jul-11	1791014	72	3	F	
1217	29-May-17	24-Sep-02	1812527	176	4	F	27
1218	01-Jul-17	13-May-07	1815533	122	4	M	19
1219	03-Jul-17	1-Feb-02	1815577	185	4	M	50
1220	03-Jul-17	16-May-16	1809396	14	2	M	6,3
1221	04-Jul-17	13-Apr-16	1784457	15	2	M	6,7
1222	04-Jul-17	19-Dec-14	1815899	31	2	M	12
1223	05-Jul-17	5-Jan-17	1815895	6	1	F	6,7
1224	15-Jul-17	21-Sep-09	1817095	94	3	M	24,5
1225	15-Jul-17	20-Apr-03	1817111	171	4	F	35
1226	15-Jul-17	19-Apr-04	1817108	159	4	F	47
1227	17-Jul-17	12-Oct-08	1811640	105	3	F	13,5
1228	18-Jul-17	28-Feb-17	1807025	5	1	F	4,9
1229	18-Jul-17	3-Dec-16	1793876	7	1	M	7,4
1230	18-Jul-17	16-Oct-13	1817347	45	2	M	11
1231	19-Jul-17	10-Mar-17	1810355	4	1	M	6,5
1232	20-Jul-17	28-Jan-09	1817705	102	3	M	30
1233	20-Jul-17	3-Feb-16	1772346	17	2	M	6,8
1234	20-Jul-17	23-Nov-12	1817688	56	2	F	13
1235	22-Jul-17	28-Jun-16	1786960	13	2	F	7,2
1236	24-Jul-17	28-Feb-07	1779091	125	4	M	16
1237	26-Jul-17	12-Nov-13	1701091	44	2	F	19
1238	27-Jul-17	28-Aug-99	1754019	215	4	F	44
1239	28-Jul-17	5-Jan-01	1818522	198	4	F	40
1240	17-Jul-17	29-Dec-14	1789134	31	2	M	9
1241	21-Jul-17	18-Oct-16	1811500	9	1	M	10,5
1242	22-Jul-17	7-Jan-17	1817842	6	1	M	7,8
1243	25-Jul-17	21-Mar-06	1817905	136	4	F	21

1244	25-Jul-17	22-Dec-13	1670149	43	2	M	10,5
1245	26-Jul-17	8-Apr-16	1778981	15	2	F	8,1
1246	26-Jul-17	7-Feb-12	1609135	65	3	M	10
1247	26-Jul-17	29-Dec-15	1794953	19	2	M	6,7
1248	27-Jul-17	2-Jun-14	1717740	37	2	M	10,6
1249	27-Jul-17	1-Dec-02	1818404	175	4	F	38
1250	27-Jul-17	25-Jul-12	1818517	60	2	M	14
1251	28-Jul-17	31-Oct-05	1366485	141	4	F	20,5
1252	29-Jul-17	31-Dec-16	1799276	7	1	M	4,5
1253	30-Jul-17	23-May-08	1667053	110	3	F	22,5
1254	31-Jul-17	5-Oct-05	1470856	141	4	F	28
1255	31-Jul-17	9-Sep-03	1724211	166	4	M	36
1256	31-Jul-17	17-Apr-16	1780499	15	2	M	6,2
1257	05-Jul-17	7-Apr-17	1815930	3	1	F	4,8
1258	08-Jul-17	17-Mar-17	1816357	4	1	F	3,5
1259	24-Jul-17	12-Feb-15	1818056	29	2	F	7,7
1260	24-Jul-17	3-Sep-13	1809700	46	2	M	14
1261	25-Jul-17	4-May-00	1757583	206	4	M	57
1262	28-Jul-17	17-Aug-00	1735123	203	4	F	27
1263	31-Jul-17	31-May-05	1818887	146	4	F	45
1264	31-Jul-17	27-Jun-16	1786857	13	2	M	8,3
1265	31-Jul-17	22-Dec-05	1227151	139	4	M	
1266	01-Aug-17	15-Jun-14	1783538	38	2	M	8
1267	01-Aug-17	8-Feb-17	1819037	6	1	M	8,5
1268	01-Aug-17	23-Apr-15	1737524	28	2	F	9,3
1269	01-Aug-17	16-Jan-08	1425356	115	3	F	14
1270	02-Aug-17	26-Jul-09	1819184	97	3	M	23
1271	02-Aug-17	21-Oct-13	1703809	46	2	M	
1272	02-Aug-17	26-Mar-08	1573156	113	3	F	34
1273	02-Aug-17	5-Feb-11	1735267	78	3	M	17
1274	04-Aug-17	31-Dec-99	1284751	212	4	F	36,5
1275	05-Aug-17	26-Jun-15	1766543	26	2	F	7,1
1276	07-Aug-17	30-Jan-08	1782323	115	3	F	22
1277	07-Aug-17	7-Jan-11	1545050	79	3	F	14
1278	08-Aug-17	15-Jun-05	1819827	146	4	M	24
1279	09-Aug-17	21-Apr-16	1770390	16	2	M	8,7
1280	09-Aug-17	8-Nov-00	1803994	201	4	F	46
1281	09-Aug-17	25-Mar-05	1350736	149	4	M	29
1282	09-Aug-17	4-Sep-16	1793254	11	1	M	6
1283	10-Aug-17	10-Aug-08	1820104	108	3	F	40
1284	11-Aug-17	9-Sep-03	1724211	167	4	M	36
1285	11-Aug-17	30-Sep-09	1818531	95	3	M	20
1286	12-Aug-17	12-Jan-01	721553	199	4	M	51
1287	14-Aug-17	31-Dec-99	1284751	212	4	F	39
1288	14-Aug-17	26-Jun-15	1766543	26	2	F	7,8
1289	14-Aug-17	23-May-16	1820273	15	1	M	7,7
1290	15-Aug-17	2-Mar-99	1799368	221	4	F	40
1291	16-Aug-17	3-Oct-00	1804767	202	4	F	50

1292	17-Aug-17	26-Jul-05	1820755	145	4	M	70
1293	03-Aug-17	22-Sep-16	1794223	11	1	M	6
1294	07-Aug-17	1-Feb-16	1802611	18	2	M	8.4
1295	14-Aug-17	14-Jan-09	1476287	103	3	F	14.8
1296	14-Aug-17	27-Jul-11	1782431	73	3	M	15
1297	14-Aug-17	1-Jan-17	1814965	7	1	M	3.2
1298	15-Aug-17	5-Jun-10	1811062	86	3	F	15
1299	16-Aug-17	27-Feb-16	1820725	18	2	F	12
1300	17-Aug-17	20-Apr-17	1820742	4	1	M	5
1301	18-Aug-17	15-Jul-08	1820650	109	3	F	21
1302	18-Aug-17	17-Apr-01	1506151	196	4	F	50
1303	18-Aug-17	4-Jan-04	1820487	163	4	F	51
1304	18-Aug-17	24-Feb-10	1820887	90	3	F	31
1305	18-Aug-17	7-Aug-11	1820781	72	3	F	15
1306	20-Aug-17	19-Apr-16	1820953	16	2	F	8.2
1307	21-Aug-17	27-Dec-14	1739683	32	2	M	10
1308	21-Aug-17	9-Feb-17	1821076	6	1	F	3.7
1309	21-Aug-17	10-Mar-17	1806370	5	1	M	6.6
1310	21-Aug-17	20-Mar-03	1727536	173	4	M	40
1311	21-Aug-17	16-Nov-15	1782526	21	2	F	8.6
1312	21-Aug-17	24-Aug-02	1820984	180	4	M	39
1313	22-Aug-17	17-Apr-17	1821297	4	1	M	6.2
1314	28-Aug-17	9-Aug-09	1821438	96	3	M	20
1315	24-Aug-17	29-Dec-15	1777922	20	2	F	7.5
1316	25-Aug-17	9-Jul-05	1821675	145	4	M	38
1317	27-Aug-17	23-May-08	1667053	111	3	F	22.5
1318	28-Aug-17	25-Apr-02	1742263	184	4	F	
1319	28-Aug-17	1-Jul-04	1340550	157	4	M	20
1320	28-Aug-17	13-Nov-04	1745475	153	4	M	30
1321	29-Aug-17	21-Nov-15	1819797	21	2	F	12.5
1322	01-Sep-17	9-Sep-09	1437377	96	3	M	23
1323	01-Sep-17	29-May-15	1821962	28	2	F	10.6
1324	04-Sep-17	23-Apr-15	1737524	29	2	F	9.7
1325	06-Sep-17	23-Oct-09	1716381	95	3	M	17
1326	07-Sep-17	2-Dec-11	1580747	69	3	M	15
1327	08-Sep-17	23-Oct-07	1751668	119	3	F	27
1328	09-Sep-17	4-Jul-08	1823229	110	3	M	27
1329	13-Sep-17	14-Oct-14	1823492	35	2	M	10
1330	16-Sep-17	14-Jan-10	1823991	92	3	M	35
1331	17-Sep-17	14-Jul-17	1817585	2	1	F	2.9
1332	18-Sep-17	7-Apr-17	1815930	5	1	F	6.2
1333	18-Sep-17	6-Dec-10	1785761	81	3	M	17
1334	20-Sep-17	15-May-17	1824383	4	1	M	7.4
1335	01-Sep-17	17-May-17	1812472	4	1	M	4.7
1336	09-Sep-17	3-Nov-15	1823217	22	2	M	11.5
1337	15-Sep-17	9-Sep-03	1724211	168	4	M	36
1338	17-Sep-17	27-Aug-01	1824035	193	4	F	70
1339	17-Sep-17	7-Jun-17	1813829	3	1	M	2.7

1340	19-Sep-17	24-May-16	1772987	16	2	M	8.9
1341	20-Sep-17	12-Jul-17	1824524	2	1	M	6.1
1342	20-Sep-17	19-Apr-17	1824532	5	1	F	8.6
1343	20-Sep-17	22-Oct-16	1824389	11	1	M	7.8
1344	20-Sep-17	29-Dec-15	1777922	21	2	F	7.2
1345	20-Sep-17	6-Dec-07	1793061	117	3	M	21
1346	25-Sep-17	17-Nov-06	1824317	130	4	F	16
1347	26-Sep-17	29-Dec-07	1365065	117	3	F	23
1348	27-Sep-17	30-Mar-17	1825082	6	1	M	7
1349	27-Sep-17	12-May-16	1801254	16	2	F	7
1350	27-Sep-17	19-Jun-16	1808228	15	2	F	6.5
1351	28-Sep-17	22-May-16	1825142	16	2	M	9.2
1352	28-Sep-17	16-Jun-10	1825293	87	3	M	18
1353	28-Sep-17	29-Mar-16	1801690	18	2	F	7
1354	29-Sep-17	24-Dec-08	1824185	105	3	M	20
1355	29-Sep-17	20-Mar-03	1727536	174	4	M	39
1356	30-Sep-17	30-Aug-17	1825501	1	1	M	3.8
1357	02-Oct-17	4-Jan-15	1715766	33	2	M	10
1358	02-Oct-17	5-Jun-17	1825265	4	1	M	6.6
1359	02-Oct-17	30-Jan-15	1718010	33	2	M	11
1360	02-Oct-17	11-Dec-13	740233	46	2	F	11.8
1361	04-Oct-17	4-Jun-16	1809916	16	2	M	7.2
1362	05-Oct-17	25-Jan-07	1825962	129	4	M	35
1363	07-Sep-17	18-Aug-03	1796051	169	4	M	26
1364	09-Oct-17	28-Aug-99	1754019	218	4	F	48
1365	18-Sep-17	12-Feb-02	1761888	187	4	F	38
1366	06-Oct-17	24-Jul-08	1826145	111	3	M	17
1367	06-Oct-17	7-Mar-07	1796931	127	4	F	21
1368	07-Oct-17	15-Oct-05	1623852	144	4	F	21
1369	09-Oct-17	26-Jun-05	1809321	148	4	F	29
1370	09-Oct-17	4-Aug-04	1826330	158	4	M	20
1371	09-Oct-17	24-Jun-13	1662835	52	2	M	11
1372	10-Oct-17	8-Jan-04	1826277	165	4	F	40
1373	10-Oct-17	13-Feb-03	1823271	176	4	F	38
1374	11-Oct-17	26-Dec-13	1826560	46	2	M	12
1375	11-Oct-17	13-Apr-17	1826487	6	1	M	5,4
1376	12-Oct-17	13-Apr-16	1778763	18	2	M	8
1377	12-Oct-17	15-Jun-06	1811301	136	4	M	19
1378	12-Oct-17	15-Nov-04	1819484	155	4	F	38
1379	13-Oct-17	28-May-02	1338625	185	4	M	28
1380	16-Oct-17	25-Sep-08	1826901	109	3	M	25
1381	16-Oct-17	3-Apr-02	1470131	186	4	M	80
1382	16-Oct-17	4-Jul-08	1823229	111	3	M	27
1383	16-Oct-17	9-Apr-17	1811457	6	1	F	6,6
1384	16-Oct-17	18-Dec-09	1818081	94	3	F	40
1385	07-Oct-17	30-Mar-17	1825082	7	1	M	5,9
1386	09-Oct-17	21-Oct-15	1826295	24	2	F	10,5
1387	09-Oct-17	17-Jul-16	1786028	15	2	F	7,3

1388	09-Oct-17	6-Jan-09	1825890	105	3	F	26
1389	09-Oct-17	2-Jun-01	1473159	196	4	F	52,5
1390	09-Sep-17	29-Jan-03	1375857	176	4	F	49
1391	19-Sep-17	29-Dec-14	1789134	33	2	M	9,7
1392	22-Sep-17	6-Aug-12	1824575	61	3	F	7,5
1393	26-Sep-17	1-Dec-13	1720950	45	2	M	14
1394	29-Sep-17	20-Jun-16	1774482	15	2	F	5,8
1395	02-Oct-17	4-May-17	1813718	5	1	M	4,3
1396	10-Oct-17	10-Nov-12	1826442	59	2	M	15
1397	10-Oct-17	17-Jan-17	1805923	9	1	F	5
1398	11-Oct-17	27-Sep-10	1825381	85	2	M	20
1399	11-Oct-17	27-Jan-17	1826600	9	1	M	6,7
1400	15-Oct-17	29-May-07	1827051	125	4	F	22,5
1401	16-Oct-17	21-Nov-14	1742210	35	2	F	9,3
1402	16-Oct-17	13-Oct-16	1798313	12	2	F	6,7
1403	16-Oct-17	6-Apr-17	1818004	6	1	M	5,4
1404	17-Oct-17	9-Jun-17	1819094	4	1	F	5,6
1405	18-Oct-17	5-Sep-07		121	4	F	31
1406	19-Oct-17	12-Mar-11	1774870	79	3	F	14
1407	19-Oct-17	22-Nov-14	1750869	35	2	F	10
1408	20-Oct-17	3-May-17	1810779	5	1	F	6
1409	22-Oct-17	16-Jan-08	1425356	117	3	F	14
1410	22-Oct-17	23-Feb-06	1802429	140	4	M	60
1411	23-Oct-17	2-Jan-05	1826256	153	4	M	34
1412	23-Oct-17	22-Apr-01	1788975	198	4	M	42
1413	23-Oct-17	24-Sep-13	1745021	49	2	M	12
1414	23-Oct-17	22-Feb-14	1674247	44	2	F	22,5
1415	23-Oct-17	10-Aug-16	1805833	14	2	M	8,7
1416	23-Oct-17	1-Oct-16	1786718	12	2	F	6
1417	24-Oct-17	28-May-00	1672517	209	4	F	
1418	25-Oct-17	7-Jul-11	1791014	75	2	F	11,2
1419	25-Oct-17	19-Apr-16	1818425	18	2	F	7,4
1420	26-Oct-17	5-Feb-16	1828050	20	2	F	7,9
1421	26-Oct-17	21-Mar-16	1808713	19	2	M	9
1422	26-Oct-17	23-Apr-15	1737524	30	2	F	9,5
1423	30-Oct-17	14-Jul-16	1827494	15	2	F	8,7
1424	20-Oct-17	1-Mar-13	1634951	55	2	M	19
1425	30-Oct-17	11-May-15	1778160	29	2	F	8,5
1426	30-Oct-17	23-Sep-13	1791573	49	2	F	12
1427	30-Oct-17	10-Aug-15	1803089	26	2	F	9
1428	30-Oct-17	18-Mar-12	1772146	67	3	M	18
1429	31-Oct-17	23-Jul-09	1824825	99	3	M	19
1430	14-Oct-17	19-Aug-17	1801619	2	1	M	27
1431	16-Oct-17	25-Apr-02	1742263	186	4	F	47,5
1432	26-Oct-17	5-Jul-17	1822282	3	1	F	2,8
1433	28-Oct-17	9-Dec-15	1827975	22	2	M	11
1434	28-Oct-17	10-Sep-17	1828637	1	1	M	40
1435	01-Nov-17	1-May-08	730491	114	3	M	20

1436	01-Nov-17	28-Feb-14	1528959	45	2	M	14
1437	02-Nov-17	7-Jul-11	1791014	76	3	F	11
1438	02-Nov-17	20-Oct-11	1829043	73	3	M	14
1439	02-Nov-17	8-Nov-16	1814920	12	2	M	6,6
1440	02-Nov-17	11-Apr-16	1827556	19	2	M	7,3
1441	03-Nov-17	28-Aug-99	1754019	219	4	F	48
1442	06-Nov-17	5-Feb-00	660922	213	4	M	25
1443	06-Nov-17	23-Apr-15	1737524	31	2	F	10,5
1444	07-Nov-17	28-Nov-10	1819965	84	3	F	22
1445	07-Nov-17	8-Mar-17	1829660	8	1	F	10
1446	07-Nov-17	6-Apr-14	1795484	43	2	F	11
1447	08-Nov-17	13-Feb-03	1823271	177	4	F	39,5
1448	09-Nov-17	19-Jun-15	1734471	29	2	F	11
1449	07-Oct-17	23-Jan-17	1826037	9	1	M	5,3
1450	09-Oct-17	18-Aug-17	1826429	2	1	M	2,8
1451	27-Oct-17	7-Jul-17	1827377	3	1	F	3,5
1452	03-Nov-17	4-Jul-17	743893	4	1	M	3
1453	03-Nov-17	1-Dec-12	1824074	59	2	M	14
1454	07-Nov-17	8-Apr-14	1829659	43	2	F	13
1455	12-Nov-17	20-Oct-07	1740632	121	4	F	26
1456	08-Nov-17	15-Nov-04	1819484	156	4	F	40
1457	09-Nov-17	4-Jun-15	1818587	29	2	F	11
1458	11-Nov-17	22-Sep-06	1826590	134	4	M	30
1459	13-Nov-17	21-Feb-16	1783486	21	2	F	8,8
1460	13-Nov-17	14-Jul-12	1794063	64	3	F	12,5
1461	16-Oct-17	17-May-17	1812472	5	1	M	5,5
1462	18-Oct-17	1-Apr-15	1758462	30	2	M	13
1463	28-Oct-17	9-Dec-01	1828516	190	4	M	32
1464	30-Oct-17	3-Dec-16	1793876	10	1	M	6
1465	03-Nov-17	21-Jul-15	1825010	28	2	F	11
1466	10-Nov-17	27-Nov-15	1802791	24	2	M	8,6
1467	07-Nov-17	16-Nov-14	1707851	36	2	M	8,6
1468	09-Nov-17	7-Sep-17	1829767	2	1	M	4,9
1469	10-Nov-17	23-Aug-14	1715829	39	2	F	6,8
1470	11-Nov-17	24-May-17	1822131	6	1	F	4,2
1471	12-Nov-17	9-Jul-17	1820737	4	1	M	4,6
1472	12-Nov-17	10-Mar-13	1810359	56	2	M	16
1473	13-Nov-17	28-Jun-05	1616039	149	4	F	42
1474	14-Nov-17	10-Oct-15	1830424	25	2	M	10,1
1475	16-Nov-17	5-Jun-14	1689713	41	2	M	10
1476	16-Nov-17	2-Dec-12	1830625	59	2	M	
1477	16-Nov-17	31-Mar-17	1830594	8	1	F	5,3
1478	17-Nov-17	4-Jul-08	1823229	112	3	M	28
1479	17-Nov-17	12-Sep-14	1830805	38	2	M	12,5
1480	19-Nov-17	19-Aug-05	1828648	147	4	M	45
1481	20-Nov-17	4-Jul-12	1602671	64	3	M	13
1482	24-Oct-17	2-Aug-17	1828054	2	1	M	4,7
1483	07-Nov-17	8-Apr-14	1829659	43	2	F	13

1484	12-Nov-17	2-Nov-02	1746877	180	4	F	35
1485	18-Nov-17	30-Apr-04	1651516	163	4	M	37
1486	19-Nov-17	9-Jun-10	1830913	89	3	M	27
1487	21-Nov-17	20-Aug-06	1820817	135	4	M	21
1488	21-Nov-17	4-May-00	1757583	210	4	M	49
1489	27-Nov-17	16-Apr-13	1637135	55	2	M	15
1490	27-Nov-17	16-Sep-06	1831133	134	4	F	37
1491	28-Nov-17	4-Apr-10	1817913	91	3	M	19
1492	28-Nov-17	18-Mar-12	1772146	68	3	M	16
1493	30-Nov-17	6-Sep-17	1829053	2	1	M	4,4
1494	30-Oct-17	27-Dec-17	1828065	-2	1	M	8,5
1495	24-Oct-17	1-Aug-12	1769701	62	3	F	10
1496	06-Nov-17	21-Jun-17	1819186	5	1	F	2,5
1497	06-Nov-17	5-Aug-16	1801442	15	2	M	7,4
1498	06-Nov-17	16-Apr-17	1808471	7	1	M	5,9
1499	12-Nov-17	26-Jan-04	1830075	166	4	F	28
1500	20-Nov-17	5-Jan-17	1815895	10	1	F	9,1
1501	21-Nov-17	29-Dec-15	1777922	23	2	F	6,7
1502	21-Nov-17	26-Sep-17	1831118	2	1	F	3,1
1503	22-Nov-17	25-Sep-00	1831303	206	4	F	49
1504	24-Nov-17	17-Aug-00	1735123	207	4	F	26
1505	04-Dec-17	23-Jul-17	1832440	5	1	F	
1506	04-Dec-17	18-Sep-11	1832343	75	3	M	17
1507	05-Dec-17	21-Sep-10	1832527	87	3	M	18
1508	05-Dec-06	8-Mar-06	1820662	9	1	F	33
1509	06-Dec-17	28-Oct-17	1832582	2	1	M	4,3
1510	06-Dec-17	14-Mar-15	1816391	33	2	F	11
1511	07-Dec-17	27-Jun-13	1832719	54	2	F	10
1512	07-Dec-17	17-Apr-14	1754085	44	2	F	11
1513	09-Dec-17	8-Oct-17	1832942	2	1	F	2,8
1514	11-Dec-17	5-Apr-15	1751301	32	2	M	10
1515	11-Dec-17	8-Dec-08	1769288	108	3	F	29
1516	11-Dec-17	18-Mar-12	1772146	69	3	M	17,5
1517	11-Dec-17	15-Nov-04	1819484	157	4	F	44
1518	12-Dec-17	13-Feb-03	1823271	178	4	F	42
1519	12-Dec-17	23-Oct-16	1828357	14	2	F	6,4
1520	14-Dec-17	6-May-02	1778754	187	4	F	39
1521	14-Dec-17	25-Jun-01	1833520	198	4	F	46
1522	14-Dec-17	4-Mar-14	1817351	45	2	M	14,5
1523	17-Dec-17	1-Jul-08	1823229	113	3	M	33
1524	16-Dec-17	3-Apr-07	1833747	128	4	M	30
1525	18-Dec-17	12-May-16	1791072	19	2	F	8
1526	18-Dec-17	28-Jan-04	1251030	167	4	F	26
1527	19-Dec-17	7-Nov-17	1834005	1	1	M	2,8
1528	19-Oct-17	11-Jul-06	1833273	135	4	F	39
1529	19-Dec-17	8-May-01	1827930	199	4	F	41
1530	20-Dec-17	14-Feb-16	1797156	22	2	M	9
1531	10-Nov-17	9-Sep-09	1437377	98	3	M	24,5

1532	01-Dec-17	17-Jun-02	1832198	186	4	M	48
1533	13-Dec-17	11-May-17	1826825	7	1	M	5,1
1534	14-Dec-17	24-May-17	1822131	7	1	F	4,1
1535	18-Dec-17	13-Apr-16	1778763	20	2	M	7,7
1536	20-Dec-17	19-Jun-17	743853	6	1	M	5,9
1537	20-Dec-17	25-Aug-13	1809531	52	2	F	10
1538	20-Dec-17	20-Mar-03	1727536	177	4	M	40
1539	23-Dec-17	23-Dec-01	1816404	192	4	M	33
1540	21-Dec-17	26-Sep-00	1831303	207	4	F	43
1541	21-Dec-17	11-Dec-13	740232	48	2	F	11,8
1542	22-Dec-17	7-Jul-11	1791014	77	3	F	12,1
1543	22-Dec-17	5-Sep-17	1833966	3	1	M	6
1544	23-Dec-17	16-Jan-01	1787461	203	4	M	47
1545	26-Dec-17	28-Dec-15	1798858	24	2	M	16,5
1546	26-Dec-17	3-May-08	1788633	115	3	F	27
1547	26-Dec-17	29-Apr-12	1616063	68	3	M	18
1548	27-Dec-17	13-Jan-17	1803252	11	1	F	6,3
1549	18-Nov-17	15-Apr-06	1830872	139	4	M	21
1550	20-Nov-17	11-Jul-17	1830936	4	1	M	5,8
1551	04-Dec-17	20-May-17	1817481	7	1	M	5,9
1552	06-Dec-17	13-Jan-13	1832702	59	2	F	20
1553	12-Dec-17	26-May-16	1832902	19	2	F	8,4
1554	19-Dec-17	4-Jan-04	1820487	167	4	F	50
1555	22-Dec-17	26-May-11	1834373	79	4	M	20
1556	23-Dec-17	14-Nov-17	1831076	1	1	M	4,2
1557	25-Dec-17	27-Sep-10	1825381	87	4	M	20
1558	27-Dec-17	9-Nov-16	1834650	13	2	M	8,7
1559	29-Dec-17	23-Aug-17	1822566	4	1	F	4
1560	30-Dec-17	7-Jul-11	1835120	77	3	F	18,5
1561	30-Dec-17	22-Jul-15	1835110	29	2	M	16
1562	02-Jan-18	21-Feb-11	1745449	83	3	F	15
1563	02-Jan-18	26-Sep-00	1831303	208	4	F	45
1564	02-Jan-18	8-Jun-15	1830753	31	2	M	11
1565	02-Jan-17	10-Jun-09	1834986	91	3	M	22
1566	04-Jan-18	24-Sep-00	1835535	208	4	F	36
1567	07-Jan-18	19-Dec-07	1834914	121	4	F	23
1568	05-Jan-18	13-Mar-04	1832228	166	4	F	22
1569	06-Jan-17	31-Jan-01	696151	192	4	F	42
1570	07-Jan-18	1-Mar-16	1790249	22	2	M	9,5
1571	08-Jan-18	31-Oct-05	1366485	147	4	F	20
1572	10-Jan-18	16-Oct-13	1817347	51	2	M	10
1573	08-Jan-18	18-Mar-12	1772146	70	3	M	15
1574	08-Jan-18	15-Nov-04	1819484	158	4	F	43
1575	10-Jan-18	13-Feb-03	1823271	179	4	F	42
1576	10-Jan-18	14-Apr-08	1782895	117	3	F	21
1577	12-Jan-18	6-Dec-10	1808314	85	3	F	16
1578	23-Oct-17	14-Jun-17	1818931	4	1	M	5,1
1579	21-Dec-17	17-Aug-00	1735123	208	4	F	26

1580	02-Jan-18	6-Nov-17	1835232	2	1	F	3,3
1581	05-Jan-18	16-Jun-03	1286515	175	4	M	16
1582	06-Jan-18	3-Dec-16	1793876	13	2	M	5,9
1583	07-Jan-18	5-Apr-11	1835811	81	3	M	23
1584	09-Jan-18	29-Mar-10	1836184	94	3	M	25
1585	09-Jan-18	6-Sep-17	1829053	4	1	M	4,3
1586	09-Jan-18	25-Dec-06	1834999	133	4	F	26
1587	09-Jan-18	4-Mar-06	1836182	142	4	F	31
1588	10-Jan-18	2-Apr-02	1836181	189	4	M	49
1589	11-Jan-18	28-Dec-05	1809197	145	4	M	28
1590	12-Jan-18	12-Apr-06	1835974	141	4	M	34
1591	12-Jan-18	11-Aug-17	1835456	5	1	M	6,3
1592	12-Jan-18	4-Jul-08	1823229	114	3	F	33
1593	15-Jan-18	17-Nov-17	1836779	2	1	M	4,2
1594	15-Jan-18	13-Apr-16	1778763	21	2	M	8,5
1595	15-Jan-18	27-Apr-07	1836784	129	4	M	40
1596	15-Jan-18	6-Jan-03	1821492	180	4	F	40
1597	15-Jan-18	4-May-00	1797583	212	4	M	50
1598	16-Jan-18	20-Nov-06	1836843	134	4	F	36
1599	16-Jan-18	23-Sep-13	1799055	52	2	F	13
1600	16-Jan-18	22-Jul-14	1836827	42	2	F	10
1601	16-Jan-18	23-May-08	1667053	116	3	F	25
1602	17-Jan-18	2-Oct-17	1826742	3	1	F	2,4
1603	18-Jan-18	8-Jan-17	1837117	12	2	M	8,7
1604	18-Jan-18	28-Mar-13	1836829	58	2	M	13,9
1605	18-Jan-18	17-Feb-17	1805483	11	1	F	7,8
1606	18-Jan-18	20-Feb-15	1769359	35	2	M	15
1607	19-Jan-18	6-Mar-01	1835463	202	4	F	37
1608	22-Jan-18	21-Feb-04	1837356	167	4	F	47,5
1609	22-Jan-18	23-Mar-01	1446369	202	4	M	37
1610	22-Jan-18	27-Oct-17	1837261	3	1	F	3,3
1611	22-Jan-18	9-Apr-15	1727708	33	2	M	7,9
1612	23-Jan-18	17-Jun-02	1832198	187	4	M	46
1613	19-Dec-17	4-Sep-10	1812418	87	3	M	17
1614	22-Dec-17	29-Aug-09	1807735	100	3	F	33
1615	01-Jan-18	1-Jan-06	1835181	144	4	M	37
1616	02-Jan-18	8-Dec-10	1835246	85	3	F	16
1617	11-Jan-18	8-Nov-04	1792782	158	4	M	27
1618	16-Jan-18	9-Feb-09	731659	107	3	M	18
1619	20-Jan-18	27-Oct-04	1836408	159	4	F	35
1620	18-Jan-18	14-Jul-17	1817585	6	1	M	4,8
1621	18-Jan-18	10-Jan-17	1800774	12	2	M	9,4
1622	20-Jan-18	12-Jun-10	1837326	91	3	M	19,5
1623	22-Jan-18	12-Jun-17	1814288	7	1	M	6,8
1624	23-Jan-18	16-Feb-15	1837792	35	2	F	14
1625	23-Jan-18	24-Aug-17	1828996	5	1	M	
1626	25-Jan-17	11-Apr-06	1832303	129	4	F	25
1627	25-Jan-18	25-Sep-17	1837801	4	1	M	

1628	25-Jan-18	9-Mar-02	1837243	190	4	M	43
1629	25-Jan-18	3-Feb-05	1837800	155	4	M	48
1630	29-Jan-18	14-May-04	1838116	164	4	F	42
1631	29-Jan-18	25-Jul-07	1363063	126	4	F	21
1632	29-Jan-18	15-Jun-17	1823507	7	1	M	7,4
1633	29-Jan-18	19-Jan-07	1727601	132	4	F	24
1634	31-Jan-18	19-Jan-06	1838241	144	4	M	48
1635	29-Jan-18	28-Sep-06	1838172	136	4	F	35
1636	29-Jan-18	20-Dec-03	1220092	169	4	F	38
1637	29-Jan-18	3-Apr-09	1838131	105	3	F	19
1638	30-Jan-18	4-Nov-17	1837495	2	1	M	5,1
1639	03-Jan-18	23-May-08	1667053	116	3	F	23
1640	30-Jan-18	22-Jun-08	1831199	115	3	M	30
1641	01-Feb-18	13-Mar-04	1832228	167	4	F	24
1642	01-Feb-18	28-Aug-07	1835709	126	4	M	27
1643	02-Feb-18	11-Apr-06	1270212	142	4	F	36,5
1644	02-Feb-18	7-Jul-11	1791014	79	3	F	10,5
1645	02-Feb-18	24-Nov-17	1836703	3	1	M	5,1
1646	05-Feb-18	2-Sep-17	1834472	5	1	F	5,3
1647	05-Feb-18	12-Jan-01	1678978	205	4	M	42
1648	05-Feb-18	5-Feb-01	1768080	204	4	F	50
1649	05-Feb-18	20-Oct-11	1805584	76	3	F	15
1650	06-Feb-18	10-Jul-06	1634804	139	4	F	15
1651	07-Feb-18	12-May-12	1803077	69	3	M	18
1652	08-Feb-18	13-Jun-13	1786677	56	2	F	16
1653	08-Feb-18	11-Mar-03	1805558	179	4	M	43
1654	08-Feb-18	25-Sep-00	1831303	209	4	F	37
1655	09-Feb-18	13-Feb-03	1823271	180	4	F	42
1656	09-Feb-18	4-Jul-08	1823229	115	3	M	34
1657	12-Feb-18	16-May-03	1839235	177	4	F	45
1658	15-Dec-17	29-Apr-17	1616063	8	1	M	18
1659	28-Dec-17	10-Nov-07	1834849	121	4	M	23
1660	01-Jan-18	15-Jun-09	1728829	103	3	M	20
1661	21-Jan-18	11-Feb-17	1837363	11	1	M	3,3
1662	28-Jan-18	21-Oct-05	1363759	147	4	F	21
1663	30-Jan-18	22-Jun-08	1831199	115	3	M	30
1664	01-Feb-18	23-Jul-11	1838640	79	3	F	22
1665	01-Feb-18	13-Jul-17	1818187	7	1	F	4,4
1666	06-Feb-18	5-Jun-06	1835181	140	4	M	34
1667	07-Feb-18	17-Jul-17	1839185	7	1	M	10
1668	09-Feb-18	5-Oct-17	1838943	4	1	F	3,2
1669	10-Feb-18	4-May-00	1757583	213	4	M	48
1670	12-Feb-18	13-May-17	1839774	9	1	F	6,5
1671	12-Feb-18	11-Jun-07	1811451	128	4	M	44
1672	12-Feb-18	15-Jun-09	1728829	104	3	M	22,5
1673	12-Feb-18	27-Aug-17	1826933	6	1	F	4,4
1674	13-Feb-18	30-Sep-15	1810403	29	2	F	10
1675	15-Feb-18	27-Dec-17	1840047	2	1	F	2,1

1676	18-Feb-18	27-Oct-04	1836408	160	4	F	37
1677	19-Feb-18	6-Sep-09	1832622	101	3	F	21
1678	19-Feb-18	18-May-16	1783060	21	2	M	8,5
1679	19-Feb-18	28-Nov-12	1840348	63	3	M	18
1680	19-Feb-18	7-Oct-12	1790188	64	3	F	11
1681	19-Feb-18	8-Aug-12	1627905	66	3	F	15
1682	20-Feb-18	31-Oct-05	1366485	148	4	F	21
1683	22-Feb-18	6-Sep-11	1812205	77	3	F	19
1684	22-Feb-18	2-Apr-02	1838795	190	4	M	42
1685	06-Jan-18	14-Jun-00	1835503	211	4	F	60
1686	05-Feb-18	28-Oct-03	1837378	172	4	M	56
1687	19-Feb-18	12-Jun-01	1840279	200	4	F	51
1688	20-Feb-18	7-Apr-04	1248228	166	4	M	34
1689	22-Feb-18	11-Nov-08	1429151	111	3	F	18
1690	26-Feb-18	26-Jan-18	1841205	1	1	M	3,1
1691	26-Feb-18	4-Jan-04	1839331	169	4	F	50
1692	26-Feb-18	20-Apr-12	1656827	70	3	M	17
1693	27-Feb-18	19-Dec-17	1841239	2	1	F	4,3
1694	01-Mar-18	20-Mar-03	1727536	180	4	M	40
1695	19-May-17	14-Apr-14	1810788	37	2	F	12,5
1696	07-Dec-16	13-Apr-03	1774990	164	4	F	33
1697	30-May-17	26-Jan-08	1812714	112	3	F	
1698	05-Jun-17	2-Nov-15	1758201	19	2	M	16,5
1699	10-Jun-17	17-Feb-17	1763822	4	1	F	
1700	12-Jun-17	20-Apr-04	1814026	158	4	M	45
1701	14-Jun-17	7-Mar-08	1819280	111	3	F	35
1702	18-Jun-17	5-May-00	1814562	205	4	M	50
1703	20-Jun-17	5-Nov-99	1815304	211	4	M	55
1704	04-Sep-17	4-Sep-16	1822615	12	2	M	10
1705	04-Sep-17	17-Apr-12	1827496	65	3	M	15
1706	05-Sep-17	24-Feb-03	1822756	175	4	M	45
1707	16-Sep-17	27-May-16	1823957	16	2	M	9
1708	17-Sep-17	12-Oct-99	1824057	215	4	F	48
1709	18-Sep-17	21-Nov-02	1821369	178	4	F	30
1710	19-Sep-17	20-Jul-16	1779192	14	2	M	13
1711	19-Sep-17	1-Mar-17	1820913	6	1	F	6,3
1712	21-Sep-17	20-Mar-01	1824563	198	4	M	70
1713	24-Sep-17	27-Sep-06	1786095	132	4	M	35
1714	27-Sep-17	9-Jan-03	1823397	176	4	M	50
1715	27-Sep-17	19-Jun-17	1743853	3	1	M	5,2
1716	30-Sep-17	9-Mar-17	743594	6	1	F	6,7
1717	17-Dec-16	15-Aug-16	1795026	4	1	F	3,3
1718	29-Nov-16	25-Apr-16	1793174	7	1	M	6,5
1719	06-Feb-17	27-Nov-16	1799323	3	1	F	4,6
1720	24-Nov-16	11-Apr-16	1792670	7	1	M	8,6
1721	28-Nov-16	9-Aug-16	1787092	3	1	M	3,1
1722	17-Apr-17	15-Aug-16	1795026	8	1	F	5,7
1723	08-Jan-17	26-Oct-10	1797317	75	3	F	16

1724	06-Dec-16	23-Mar-16	1793742	9	1	F	7,5
1725	20-Dec-16	30-Oct-00	1795059	194	4	M	55
1726	18-Feb-18	21-Oct-03	1840246	172	4	M	
1727	15-Feb-18	3-Jun-08	1840042	116	3	F	
1728	19-Feb-18	15-Jun-17	1840253	8	1	M	8,5
1729	06-Feb-18	9-Apr-14	1838820	46	2	M	13
1730	22-Jan-18	24-Mar-06	1816082	142	4	F	45
1731	28-Dec-17	20-Mar-16	1769735	21	2	F	10
1732	19-Dec-17	5-Oct-17	1827737	2	1	F	4,1
1733	05-Dec-17	5-Apr-04	1832564	164	4	M	36
1734	10-Jan-18	30-Nov-09	1836195	98	3	F	25
1735	02-Feb-18	19-Jun-17	1832504	8	1	M	5,9
1736	28-Dec-17	9-Jan-17	1833941	11	1	F	8
1737	07-Feb-18	5-Feb-16	1795948	24	2	M	14
1738	11-Feb-18	21-Feb-03	1830582	180	4	M	43
1739	29-Jan-18	14-Sep-17	1838295	4	1	F	5,8
1740	28-Jan-18	21-Dec-17	1838099	1	1	M	13
1741	29-Jan-18	7-Jan-17	1817842	12	2	M	8
1742	27-Dec-17	5-Apr-09	1414057	104	3	M	17
1743	11-Jan-18	16-May-16	1809396	20	2	F	7
1744	06-Feb-18	31-Dec-17	1839177	2	1	M	4
1745	24-Aug-17	27-Feb-16	1820725	18	2	F	12
1746	23-Aug-17	27-Aug-17	1789035	0	1	M	15
1747	30-Jan-17	5-May-17	1821681	-4	1	M	6
1748	30-Aug-17	10-Jul-17	1822259	1	1	M	2,6
1749	29-Aug-17	5-Apr-15	1822078	28	2	M	12
1750	12-Feb-18	10-Mar-04	1813744	167	4	F	30
1751	23-Aug-17	14-Feb-15	1821309	30	2	M	13
1752	22-Aug-17	7-Jul-17	1821288	1	1	M	2,6
1753	20-Aug-17	1-Mar-17	1820913	5	1	F	6,3
1754	13-Aug-17	6-Feb-01	1120922	198	4	F	59
1755	16-Aug-17	27-Jun-17	1817667	2	1	M	4,9
1756	14-Aug-17	8-Dec-12	1814877	56	2	M	18
1757	13-Aug-17	1-Jul-16	1783182	13	2	F	7
1758	11-Aug-17	4-Sep-13	1816213	47	2	M	14,3
1759	10-Aug-17	3-Jun-14	1819793	38	2	F	12,5
1760	01-Aug-17	13-Dec-13	1818868	44	2	F	12
1761	02-Jul-17	7-May-10	1599386	86	3	M	50
1762	03-Jul-17	12-Sep-08	1815576	106	3	F	10
1763	25-Jul-17	27-Apr-17	1818496	3	1	M	5,5
1764	20-Feb-18	14-Feb-13	1840461	60	3	F	17
1765	26-Feb-18	13-Feb-13	1841031	60	3	F	13
1766	25-Feb-18	15-Dec-17	1841005	2	1	F	4,2
1767	27-Feb-18	3-Dec-16	1793876	14	2	M	6,4
1768	24-Feb-18	19-Mar-07	1659806	131	3	F	20
1769	20-Feb-18	25-Jun-17	1818779	8	1	F	5,2
1770	08-Feb-18	10-Feb-16	1815913	24	2	M	9
1771	02-Mar-18	13-Jan-17	1841699	14	2	F	9,8

1772	20-Jan-18	5-Mar-05	1837337	154	4	M	54
1773	13-Feb-18	19-Oct-12	1839924	64	3	F	17
1774	23-Feb-18	9-Jan-18	1840853	1	1	M	3,2
1775	27-Feb-18	29-Aug-09	1807735	102	3	F	33
1776	01-Mar-18	10-Apr-06	1311527	143	4	M	33
1777	01-Mar-18	26-Jun-01	1838016	201	4	F	90
1778	02-Mar-18	7-Jul-17	1791014	8	1	F	12
1779	04-Mar-18	16-Sep-17	1841769	6	1	F	5,8
1780	05-Mar-18	19-Jan-15	1835905	38	2	M	12
1781	05-Mar-18	25-Apr-16	1778314	23	2	F	8,9
1782	06-Mar-18	21-Nov-06	1554167	136	4	F	25
1783	07-Apr-17	13-Jul-17	1807075	3	1	F	9
1784	05-Apr-17	7-Sep-14	1711105	31	2	F	10
1785	07-Apr-17	28-Oct-01	1807080	186	4	M	
1786	23-Mar-17	3-Nov-11	1805680	64	3	F	17
1787	31-Mar-17	13-Apr-16	1806433	11	1	M	8,7
1788	31-Mar-17	18-Jun-01	1806447	189	4	F	36
1789	03-Apr-17	30-Jul-15	1806688	21	2	F	8,8
1790	24-Apr-17	10-Jul-99	661790	213	4	M	43
1791	18-Apr-17	13-Feb-17	1805912	2	1	M	2,6
1792	16-Apr-17	27-Aug-15	1773989	20	2	F	
1793	06-Apr-17	20-Oct-16	743404	6	1	F	6,6
1794	27-May-17	13-Apr-03	1774990	169	4	F	32
1795	17-May-17	11-Sep-16	1811254	8	1	F	6,5
1796	25-Jan-17	22-Oct-14	1787550	27	2	F	10
1797	04-Sep-17	8-Apr-17	1774538	5	1	F	21
1798	08-Apr-17	9-Mar-09	1797331	97	3	F	22
1799	24-Mar-17	3-Feb-17	1805793	1	1	M	4,1
1800	26-Dec-17	4-Jul-16	1833698	17	2	F	7,4
1801	30-Aug-17	5-Jul-17	1822262	1	1	F	4,4
1802	15-Jan-18	12-Nov-17	1836785	2	1	M	4,7
1803	23-Dec-17	2-Jan-05	1834475	155	4	M	60
1804	09-Dec-17	2-May-16	1832920	19	2	F	10
1805	27-Jan-18	15-Apr-07	1836919	129	4	M	23
1806	16-May-17	1-Jun-11	1811059	71	3	M	34
1807	14-May-17	27-Sep-15	1810828	20	2	M	18,5
1808	09-Feb-18	27-Mar-00	1839425	215	4	M	54
1809	15-Feb-18	24-Sep-05	1840097	149	4	M	28
1810	20-Feb-18	25-Apr-00	1831303	214	4	F	37
1811	05-Mar-18	13-Mar-04	1832228	168	4	F	24
1812	06-Mar-18	20-Dec-03	1220092	171	4	M	38,5
1813	09-Mar-18	31-Mar-07	1842235	132	4	M	44
1814	12-Mar-18	11-Dec-13	740233	51	2	F	12
1815	12-Mar-18	14-Feb-15	1825290	37	2	F	11
1816	12-Mar-18	1-Jun-11	1838678	81	3	M	16
1817	12-Mar-18	29-Apr-09	1842580	107	3	M	23
1818	12-Mar-18	14-Aug-01	1842135	199	4	F	37
1819	12-Mar-18	6-Mar-17	1808805	12	2	F	7

1820	12-Mar-18	15-Jun-09	1728829	105	3	M	25
1821	13-Feb-18	10-Feb-04	1821344	168	4	M	31
1822	13-Mar-18	17-Nov-08	1842767	112	3	F	23
1823	14-Mar-18	25-Jul-06	1813629	140	4	M	6,2
1824	14-Mar-18	18-Apr-09	1415474	107	3	F	26
1825	14-Mar-18	23-Nov-14	1755984	40	2	M	11
1826	15-Mar-18	29-Sep-05	1803437	150	4	F	55
1827	15-Mar-18	4-Oct-01	1647079	197	4	F	57
1828	15-Mar-18	17-May-17	1825333	10	1	M	5,4
1829	16-Mar-18	4-Jan-04	1839331	170	4	F	55
1830	17-Mar-18	31-Oct-17	1843356	5	1	M	4,1
1831	19-Mar-18	5-Mar-16	1817165	24	2	F	7,6
1832	19-Mar-18	10-Sep-12	1792584	66	3	F	14
1833	19-Mar-18	8-May-01	1827930	202	4	F	41
1834	20-Mar-18	21-May-00	1379376	214	4	M	36
1835	21-Mar-18	21-Jun-09	1691330	105	3	F	28
1836	22-Mar-18	7-Jul-11	1791014	80	3	F	12
1837	22-Mar-18	12-Jun-10	1837326	93	3	M	20
1838	23-Mar-18	13-Sep-17	1843853	6	1	F	2,5
1839	10-Feb-18	26-Jun-15	1766543	32	2	F	8,8
1840	27-Feb-18	9-Nov-04	1733907	159	4	F	39
1841	28-Feb-18	10-Sep-16	1815596	17	2	M	8,4
1842	02-Mar-18	1-Dec-13	1841687	51	2	M	9,5
1843	04-Mar-18	22-Jul-11	1831517	80	3	M	15
1844	05-Mar-18	9-Jan-15	1841799	38	2	M	17
1845	05-Mar-18	11-Nov-17	1835289	4	1	F	6,7
1846	06-Mar-18	21-Jun-17	1819186	9	1	F	4,9
1847	06-Mar-18	20-Aug-17	1877609	7	1	M	6,7
1848	07-Mar-18	2-Jun-09	1842182	105	3	F	2,1
1849	21-Mar-18	24-Sep-00	1835535	210	4	F	58
1850	22-Mar-18	15-Aug-11	1838365	79	3	M	17
1851	26-Mar-18	9-Nov-04	1733907	160	4	F	35
1852	26-Mar-18	20-Aug-08	1832040	115	3	M	22
1853	26-Mar-18	14-Oct-17	1831844	5	1	M	4,7
1854	26-Mar-18	14-Mar-16	1818695	24	2	F	8,1
1855	28-Mar-18	7-Apr-09	1844558	107	3	F	22
1856							
1857				1	483		
1858				2	479		
1859				3	365		
1860				4	527		
1861					1854		
1862							
1863							
1864							
1865							
1866							
1867							

1868
1869
1870
1871
1872
1873
1874
1875
1876
1877
1878
1879
1880
1881
1882
1883
1884
1885
1886
1887
1888
1889
1890
1891
1892
1893
1894
1895
1896
1897
1898
1899
1900
1901
1902
1903
1904
1905
1906
1907
1908
1909
1910
1911
1912
1913
1914
1915

1916
1917
1918
1919
1920
1921
1922
1923
1924
1925
1926
1927
1928
1929
1930
1931
1932
1933
1934
1935
1936
1937
1938
1939
1940
1941
1942
1943
1944
1945
1946
1947
1948
1949
1950
1951
1952
1953
1954
1955
1956
1957
1958
1959
1960
1961
1962
1963

1964
1965
1966
1967
1968
1969
1970
1971
1972
1973
1974
1975
1976
1977
1978
1979
1980
1981
1982
1983
1984
1985
1986
1987
1988
1989
1990
1991
1992
1993
1994
1995
1996
1997
1998
1999
2000
2001
2002
2003
2004
2005
2006
2007
2008
2009
2010
2011

2012
2013
2014
2015
2016
2017
2018
2019
2020
2021
2022
2023
2024
2025
2026
2027
2028
2029
2030
2031
2032
2033
2034
2035
2036
2037
2038
2039
2040
2041
2042
2043
2044
2045
2046
2047
2048
2049
2050
2051
2052
2053
2054
2055
2056
2057
2058
2059

2060
2061
2062
2063
2064
2065
2066
2067
2068
2069
2070
2071
2072
2073
2074
2075
2076
2077
2078
2079
2080
2081
2082
2083
2084
2085
2086
2087
2088
2089
2090
2091
2092
2093
2094
2095
2096
2097
2098
2099
2100
2101
2102
2103
2104
2105
2106
2107

2252
2253
2254
2255
2256
2257
2258
2259
2260
2261
2262
2263
2264
2265
2266
2267
2268
2269
2270
2271
2272
2273
2274
2275
2276
2277
2278
2279
2280
2281
2282
2283
2284
2285
2286
2287
2288
2289
2290
2291
2292
2293
2294
2295
2296
2297
2298
2299

2300
2301
2302
2303
2304
2305
2306
2307
2308
2309
2310
2311
2312
2313
2314
2315
2316
2317
2318
2319
2320
2321
2322
2323
2324
2325
2326
2327
2328
2329
2330
2331
2332
2333
2334
2335
2336
2337
2338
2339
2340
2341
2342
2343
2344
2345
2346
2347

2444
2445
2446
2447
2448
2449
2450
2451
2452
2453
2454
2455
2456
2457
2458
2459
2460
2461
2462
2463
2464
2465
2466
2467
2468
2469
2470
2471
2472
2473
2474
2475
2476
2477
2478
2479
2480
2481
2482
2483
2484
2485
2486
2487
2488
2489
2490
2491

2492
2493
2494
2495
2496
2497
2498
2499
2500
2501
2502
2503
2504
2505
2506
2507
2508
2509
2510
2511
2512
2513
2514
2515
2516
2517
2518
2519
2520
2521
2522
2523
2524
2525
2526
2527
2528
2529
2530
2531
2532
2533
2534
2535
2536
2537
2538
2539

~~Height~~	Referral/community patient	Diagnosis Underlying disease1	Underlying disease2	Underlying disease3	Underlying disease4	Underlying disease5
48	Self adm	Pneumonia				
	Referral	DHF gr II				
67	Referral	Prolonged fever	Diare persisten	UTI		
75	Referral	Prolonged fever	CMV			
146	Self adm	TB abdomen	Efusi Pleura			
	Referral	DSS	Efusi Pleura			
96	Referral	KDS	ISK	CC		
95	Referral	Susp Invaginasi	SIRS	Diare Cair Akut	Tanpa Dehidrasi	
80	Self adm	CAP	Kolestasis Intra	Sirosis Hepatis	CMV	
69	Referral	DSS	Efusi Pleura	GIT Bleeding	DCA tanpa dehidrasi	
133	Referral	ISK	UTI	Fimosis		
46	Self adm	PJB sianotik	ᵓiere Robin Sync	FTT		
60	Referral	PJB sianotik				
70	Self adm	CAP dd Orthostatic	Hidrocepalus			
150	Referral	DHF gr III	Efusi Pleura			
129	Referral	DHF gr III				
108	Self adm	Asma Intermitten	Mild moderate	RFA		
148	Referral	DHF	Efusi Pleura			
148	Referral	DHF gr II	Encephalopath	Efusi Pleura		
135	Referral	Expanded dengue	Ensefalopati de	DSS H-7	Sepsis	
54	Self adm	ME	Pneumonia			
81	Referral	Epilepsi	CP			
126	Referral	DSS hr-5	Efusi Pleura	GIT Bleeding		
85	Referral	DHF gr III	Efusi Pleura	Encephalopaty Dengue		
120	Referral	DHF				
85	Referral	DHF gr I	Efusi Pleura			
	Self adm	DSS	Efusi Pleura	GIT Bleeding		
150	Referral	DSS				
122	Referral	Decomp cordis	Efusi Perikard			
63	Referral	Vomitus dg dehidrasi tak berat ec		DCA dd ISK		
	Transfer	Holoproenchepalis	Neonatal seizu	Sepsis	Diabetes Insipidius	
68	Self adm	Morbili	Pneumonia	Encephalitis	Cerebral palsy	
	Self adm	DHF	Git bleeding	DIC		
53	Self adm	ME susp ec bakterial dd viral				
113	Self adm	DHF	Multiple Caries dentis			
149	Referral	DSS				
9,5	Referral	Encephalitis	DCA tanpa dehidrasi			
73	Referral	DHF	Efusi Pleura	DCA	KDS	
49	Referral	HF	PJB asianotik	Pneumonia	DCA	Sepsis
82	Referral	Epilepsi	Atrofi cerebri	Tetraparesis		
85	Transfer	pneumonia	TB paru	Cerebral Palsy	Hidrocepalus	
	Referral	DSS				
145	Referral	encephalitis	Oedema cerebr	Demam Typoid	Fhlebitis	

80	Self adm	Kejang dg demam	ISK			
98	Self adm	TB paru	Kista Pankreas			
68	Self adm	Kolestasis Intrahep	Sirosis hepatik (	Acites		
79	Referral	GEFS+	Microcepaly	Handy Walker Syndrome		
63	Referral	DHF	Efusi Pleura			
72	Referral	Pneumonia	Asma			
153	Referral	DSS	Efusi Pleura			
124	Referral	DSS	Git bleeding	Efusi Pleura		
57	Referral	Pneumonia	PJB asianotik			
93	Referral	Encephalopathy se	Cerebral palsy	Sepsis berat		
70	Self adm	Prolonged fever ec	DCA tanpa dehidrasi			
155	Referral	Abses Cerebri dd T	TB pulmo	B20		
64	Referral	DSS	Efusi Pleura	Diare Cair Akut	Tanpa Dehidrasi	
	Referral	Severe sepsis	CAP	Edema pulmo	PJB Asianotik	Efusi Pleura
	Referral	DSS	Acites	Efusi Pleura	Git bleeding	
95	Referral	Pneumonia	Tetraparesis Spastik			
111	Referral	Meningoencephali	Epilepsi	TB paru		
68	Referral	DSS	Efusi Pleura	DCA dg dehidrasi tak berat		
62	Referral	Epilepsi	Hidrocepalus	Commoncold	Varingitis	Flebitis
	Referral	Encefalitis viral sus	Hidrocepalus obstruktif			
40	Referral	Riw epileptikus	bronkitis	ME viral dd bak	ISK	
72	Referral	DCA pd gizi buruk r	HAEC	Diare persisten		
120	Referral	Susp perdarahan ir	hemikorea ec s	infeksi HSV		
113	Self adm	Dengue				
43	Referral	CP tetraparesis spa	Ventrikulomega	Atrofi cerebral	Urtikaria	CMV
146	Referral	DHF gr I				
60	Self adm	CMV	Hernia umbilikalis			
	Referral	pneumonia	multiple infark	dermatitis		
108	Transfer	Efusi Pleura dd bronchiectasis dd masa paru dd abses paru				
86	Referral	Susp meningoence	Andonis respiratorik berat			
71	Self adm	B20	TB paru			
70	Transfer	Syok septik	Susp Meningoe	Diare Cair Akut		
74	Referral	Syok septik	Sepsis berat	Susp HAEC	Hospital accuari	Pneumonia
65	Self adm	CMV primer	ADS Severe SNI	Nystagmus Con	Down Syndrome	
46	Referral	Kolestasis Intrahepatal				
57,5	Referral	CAP	PJB sianotik	Atresia pulmonal		
55	Referral	Susp pertusis	Pneumonia			
65	Referral	DSS	Efusi Pleura	DCA tanpa dehidrasi		
115	Referral	Epilepticus overcor	Encepalitis bakt	Hipokalsemia	Crusta Scabies	
73	Referral	DSS	Efusi Pleura	Acites	Edema pulmo	DCA tanpa dehi
130	Referral	CKD	Infeksi luka HD	HT st II		
150	Referral	TB Paru	TB abdomen			
87	Self adm	Syok hipofolemik	Epilepsi	Dekubitus		
64	Referral	Kolestasis intrahep	CMV	Sirosis hepatis		
77	Referral	Prolonged fever	ISK			
79	Self adm	KDS ec faringitis	ISK	HAP		
75	Referral	HIV st III				
55	Referral	SBP	Kolestasis Intra	Acites	PJB Asianotik	Sirosis hepatis c

160	Self adm	Neuroritinitis	CMV			
106	Referral	CMV	ODS nistagmus	Toxoplasmosis kongenital		
56,5	Referral	Hepatoblastoma	Diare kronis	Phymosis		
46	Referral	Epilepsi	Diabetes insipic	ME		
78	Referral	CMV	CP tetraparse s	Sepsis	Epilepsi	Decubitus
109	Referral	B20	Pneumonia	Bronchiectasis		
100	Referral	DHF gr I	Efusi Pleura	Acites		
55	Self adm	Kolestasis	CMV			
154	Referral	Thypoid Fever	Stomatitis			
165	Self adm	Epilepsi	Hidrocephalus	Halusinasi orga	RFA	
52	Self adm	Pneumonia	PJB asianotik			
51,5	Referral	Pneumonia	PJB asianotik	Anemia	Konjungtivitis	Kolestasis ekstr
51,8	Referral	PJB asianotik	Pneumonia	Down syndrom		
49	Self adm	CMV	Congenital rubella sndrm			
55	Referral	Hidrocepalus	DCA tanpa dehi	Pneumonia	Sepsis	ISK
120	Referral	Syok hipofolemik	Diare cair akut	Utikaria		
118	Referral	DSS	Git bleeding			
51	Self adm	Kolestasis	Kista duktus	ISK		
51	Referral	DCA dehidrasi tak I	riw post op volvulus			
66,5	Self adm	Epilepsi	DCA tanpa dehi	RFA		
140	Referral	DHF gr I				
133	Referral	DSS hr-6				
71	Referral	Pneumonia	PJB asianotik			
131	Referral	DHF gr II	Efusi Pleura			
61	Referral	HF	PJB sianotik	Truncus Art tp I	PH	CAP
150	Referral	Encephalopaty der	DSS	Efusi Pleura		
148	Referral	DSS	Efusi Pleura	Ascites	DIC	
125	Self adm	PJB sianotik				
76	Self adm	Dehidrasi pd gizi bu	Kwarsiorkor	Hirschprung	Syok hipovolemik	
116	Referral	Abses Cerebri	PJB sianotik	Pulmonal Atresia		
153	Referral	Tetraparesis spasti	UMN	Myelitis Transversa		
52	Referral	Down syndrom klir	PJB asianotik			
79	Self adm	AV Blok Total	PJB asianotik			
50	Self adm	Hidronefrosis bilat	HT st II	Wheezing infan	Pneumonia	
98	Self adm	Nistagmus kongen	Pareses N IV	GDD		
119	Referral	KAD	DM tp I			
84	Referral	CKD	HT st II	Hidronefrosis B	Cystitis Kronis	Polidactily
141	Referral	Encephalitis	Upper Gastroin	Edema cerebri		
87	Self adm	PJB asianotik	CRS			
50	Self adm	Pneumonia	PJB asianotik			
67	Self adm	Pneumonia	PJB asianotik			
124	Self adm	PJB asianotik				
62	Self adm	PJB asianotik				
	Self adm	Meduloblastoma	Tumor Intracer	HT st II	Hidrocepalus	
	Self adm	Dehidrasi tak berat	Hidrosefalus			
159	Self adm	Epilepsi gtc belum teratasi				
93	Referral	Hematuria ec susp	ISK			
	Referral	Gastritis erosif	Duodenitis	Vomitus Profuse dg dehidrasi tak berat		

133	Self adm	PJB asianotik				
150	Referral	Kista duktus koledukus				
160	Self adm	Nefritis lupus	Anemia mikrositik hipokromik			
156	Self adm	SLE	Sirosis hepatik	HT st II	Sindrome nefrotik	
96	Self adm	Retensio Urine	Anemia normo:	Nefritis HSP	Hidrocepalus	Cystitis
44	Referral	Edward syndrome	Asfiksia neonat	Mild HVE		
144,5	Self adm	Sindrom nefrotik	Dependent ster	HT st I		
154	Self adm	Vomitus profuse ta	gastroduc denit	epilepsi abdomen		
69	Self adm	PJB asianotik				
	Self adm	SLE nefritis				
85	Self adm	Abses Cerebri mult	Ventrikularis	Hemiparesis		
137	Referral	Tetraparesis flaccic	CMV	Lobs susp dd myelitis transversa		
143,5	Referral	Gastritis				
54	Self adm	Effusi pericard				
	Referral	Probable dengue				
67	Self adm	PJB asianotik	Down syndrome			
89	Self adm	PJB asianotik				
148	Self adm	SLE				
109	Self adm	PJB sianotik				
66	Self adm	Kolestasis ekstrahe	Hernia umbilikalis			
130	Referral	PJB asianotik				
98	Self adm	Nefropati				
131	Self adm	Varises esofagus gı	HT			
92	Self adm	SNDS relaps				
135	Self adm	Hematemesis ec va	HT portal			
152	Self adm	Hepatitis Akut	HT gr II	Glaukoma sekunder		
156	Self adm	HT urgensi	SNRS dalam protokol mg III-IV			
126	Referral	Sindrom nefrotik	HT gr II			
	Self adm	Vomitus dg dehidr	Cerebral palsy			
	Transfer	Shock sepsis overc	post LE	Anemia		
54	Referral	Susp PDVK	Kolestasis intrahepatal			
142	Referral	HT gr II belum terk	Hydronfrosis bi	Retinopati HT ODS		
49	Transfer	Neonatal Seizure	PVL grade IV Ve	Sepsis	Neonatal Jaundice	
149	Self adm	PJB asianotik				
146	Referral	PJB asianotik				
101	Self adm	PJB sianotik				
139	Self adm	Varises esofagus gr III ec HT Porta Ec Non sirosis hepatis				
78	Self adm	ISK kompleks ec pc	Bardet biedl syı	CKD st V		
147	Referral	PJB asianotik	Infective endoc	Anemia gravis		
122	Referral	Melena	Varises Esofagu	Banti's Syndron	Flebitis	
130	Referral	SN	AKI Fase remisi	HT ST II		
	Self adm	SLE	Anemia Severe			
83	Referral	SN	AKI	Efusi pleura	HT st II	
132	Self adm	DMD				
158	Referral	PJB asianotik				
86	Self adm	PJB asianotik				
126	Self adm	PJB asianotik				
120	Referral	Kolik abdomen susp nefroktiasis				

54	Transfer	Pneumonia berat	ARDS	Hernia Umbilik	Diare cair akut tanpa dehidrasi		
87	Self adm	Pneumonia	Enchepalitis HS	Tetraparesis Spastik			
	Referral	Koma ec encephal	Acute hepatic f	GIT Bleeding			
145	Referral	HF	PJB asianotik				
143	Self adm	SN resisten steroid	Acites	Edema Anasarka ed hypoalbumin			
63	Self adm	Kolestasis Intrahep	CMV	Susp sirosis hepatis			
147	Referral	Nefritis HSP					
52	Referral	ME bakterial	Anemia mikrositik hipokromik				
58	Referral	Kolestasis Intrahep	DCA tanpa dehi	RFA			
103	Referral	PJR					
125	Self adm	Vomitus profuse d	Pangastritis dgn	asma			
124	Self adm	PJB sianotik	Down syndrome				
114	Self adm	DMD					
82	Self adm	ISK	SNHL AS				
103	Referral	AHP ec GBS dd mipelitis transversa					
154	Referral	SLE					
148	Self adm	CP tetraparesis spa	Lesi LMN	HT st I			
130	Referral	CKD st V	Efusi pleura	HT st I	ISK		
113	Self adm	SN dependent ster	Pneumonia				
	Self adm	Urtikaria	Angioedema	Nefritis	Tonsilofaringitis acute		
105	Referral	ISK	SN dependent s	Katarak ec steroid			
54,5	Referral	Kolestasis ekstrahe	Hernia umbilikalis				
163	Self adm	SLE	HT terkontrol				
56	Referral	PJB asianotik					
55	Self adm	PJB sianotik	Atresia pulmonal dg intact ventriculas septum				
139	Self adm	PJB asianotik	HT st II				
74	Self adm	IEM	Fatty liver	Hernia Umbilikalis			
116	Self adm	PJB sianotik	Rinitis alergika				
107	Referral	Space occupy lesion					
114	Self adm	SNRS	HT st II	Glaukoma ec ES	RFA		
147	Self adm	Obs nyeri perut epigastrik					
124	Referral	HT emergency	Encephalopath	GNA non Streptokokus DD SN SLE			
153	Self adm	Gastritis	ISK	dehidrasi tidak berat ec low intake n fomit			
130	Referral	Decomp cordis	Cardiomyopathy dilatasi				
123	Referral	Acute kidney injur	Acute nefritis s	Anemia mikrosi	HT		
164	Self adm	PJB asianotik					
	Referral	PJB asianotik					
157	Self adm	PJB asianotik					
122	Self adm	PJB sianotik					
146	Referral	PJB asianotik	hepatorenal sy	Kolestasis	Cardiac sirosis	Cauti	
	Referral	PJB sianotik	RFA				
142	Referral	Hiperglikemia pd D	Common cold				
112,5	Referral	Perdarahan Saluran Cerna Bawah dd Polip					
132	Referral	CKD	Anemia on ren	Susp clabsi			
118	Referral	PJR					
166	Self adm	PJB asianotik					
69	Referral	PJB asianotik					
65	Referral	Spasmeinfantil dd Myoclonic epilepsi					

157	Self adm	PJB asianotik	RFA			
125	Referral	Vomitus dg dehidr	Pangastritis	Ulcus peptikum	Asma persisten ringan	
86	Self adm	Vomitus dg dehidrasi tak berat ec susp pancreatitis akut				
117	Referral	GIT bleeding ec sus	HT portal	Sirosis Hepatis		
152	Self adm	Gastritis Akut				
74	Self adm	Enteritis dg dehidrasi tak berat dd ISK				
84	Self adm	PJB Asianotik				
145	Referral	Kejang pd pasien g	Hidrosefalus			
53	Referral	ME	Celulitis dd abs	Pneumonia		
114	Transfer	Syok septik syndro	Sepsis	ME bacterial	CNS relaps pd post limfoma burk	
65	Self adm	Probable dengue	Tetraparesis Sp	CMV		
124	Referral	DF hr V				
105	Referral	DHF	Efusi pleura	DIC		
71	Referral	Typoid Fever	Anemia mikrosi	Prolonged Feve	PJB Asianotik	
111	Self adm	Typoid Fever dd ISI	Pneumonia			
121	Self adm	GEFS	RFA	Probable Dengue		
75	Referral	DHF gr II	Efusi pleura			
74	Referral	Susp Viral Cromp				
67	Referral	DF hr III				
48	Self adm	PJB asianotik	Pneumonia	Syok septic	Sepsis Berat	
123	Referral	DSS hr VI	Efusi pleura	Edema Pulmo		
74	Transfer	Post thoracotomy,	Asidosis resp	ARDS berat	Pneumonia bera	Git bleeding
	Transfer	Atresia Bilier tp 2 p	CMV	Atrofi cerebral		
74	Referral	Tetraparesis flaccid	CMV	Susp Guillain Ba	Diare cair akut ta	Rhinofaringitis a
45	Referral	Fistula tracheosofa	PJB asianotik	Pneumonia	Sepsis neonatorum	
107	Referral	DSS hr V	GIT bleeding	Asidosis metabolik		
86	Referral	Severe sepsis	Syok septik	Pneumonia	ISK	Infeksi Intracrar
109	Referral	DSS Overcome	Efusi pleura			
	Referral	ISK	DSS overcome	GIT Bleeding ec	Ensefalopati den	Sepsis
141	Referral	DSS Overcome				
115	Referral	DSS Overcome	Efusi pleura	Git Bleeding		
145	Referral	DRA	ISK			
142	Referral	SLE	Efusi pleura	Nefritis Lupus	CKD ST IV	
130	Referral	Me bacterial dd vir	CP tetraparese	Sepsis	Shock septic	Hidrosephalus
162	Referral	Syok Septic	Sepsis	Encephalopaty	DIC	Git bleeding
110	Referral	DSS not overcome	Oedema pulmo			
56,2	Transfer	Tumor Intracerebr	Hidrosefalus	mixed asidosis	Sepsis	Syok Septic
51	Referral	PJB	Syndrome rube	Pneumonia	DCA	
113	Referral	DSS overcome				
66,5	Referral	Atresia Bilier	Prolonged Feve	CMV	Susp spontaneus	Sepsis
100	Transfer	PCP	All SRFS Mainte	Syok kardiogen	CAP	PCP
	Transfer	Syok septik	Sepsis	Demam neutropenia		
56	Referral	Edema cerebri	SAH	Kolestasis extra	Cushing Syndrome	
56	Referral	Syok septik	Sepsis	CAP	Syok hipovolemi	HAP
61	Self adm	Kolestasis ec inf CN	Infeksi luka ope	Post Kasai pro a	Sepsis berat	DIC
140	Referral	Resp distress	Sepsis	Demam neutro	Abses mamae	Kolestasis intral
80	Referral	Reccurent pneumo	Anemia	Obesitas	KIPI post campal	DCA
	Transfer	Sepsis	Pneumonia ber	ARDS ringan	Distress resp	

57	Referral	Syok septic overco	Severe sepsis	Pneumonia	Seizure ec Hypog	Git bleeding
125	Referral	Expanded dengue :	DSS overcome	GIT bleeding	ARDS moderate	DCA
124	Self adm	Anemia on CKD	CKD on CAPD	Inpatensi CAPD		
	Self adm	Kejang GTC epileps	Tetraparesis Spastik		ME dd abses cerebri	
155	Referral	Malignancy	VCS syndrome	ISK	Infark cerebri ec	Susp renal emb
64	Referral	DCA dehidrasi tak l	Epilepsi	Holoproenchep	ADS severe SNHI	Agenesis Corpu
132	Self adm	CKD st V	Susp Edema pu	HT st II	Impatensi HD cath	
150	Self adm	Susp ensefalitis via	sepsis	HT	ISK	Tonsilitis kronis
79	Referral	Pneumonia	GDD	KIPI	DCA	
89	Self adm	PJB asianotik	Kabuki Syndron	GDD		
153,5	Self adm	PJB asianotik				
102	Referral	PJB asianotik				
68	Referral	Pneumonia	PJB asianotik	GDD		
108	Referral	DSS	Efusi pleura			
107	Self adm	PJB sianotik				
161	Referral	SLE	HT gr I	Neuropsikiatri l	GMO	
68	Referral	DHF gr II	RFA	Kejang demam	DCA tanpa dehic	ISK dg pnymosis
68	Self adm	PJB asianotik	DS			
67	Self adm	SCID	TB	Pneumonia	Abses hepar	FTT
	Referral	Intoksikasi				
104	Referral	Intoksikasi	Seizure ec bradifacum toxicity dd intracranial bnleeding			
60	Self adm	Dengue fever hr V dg warning sign				
45	Referral	PJB asianotik				
75	Self adm	PJB asianotik				
73	Referral	PJB asianotik	Pulmonary hyp	Epilepsi terkont	Hernia scrotalis	Hernia umbilika
117	Self adm	ISK	B20 dlm tx ARV	Anemia Micro-hypo		
54	Self adm	Status epileptikus	Encephalitis dd	Pneumona	West syndrome	
102	Self adm	Hematosezia ec susp colitis dd polip				
58	Referral	Pneumonia	PJB asianotik	Sepsis	HAP	
65	Referral	CAP	DCA dehidrasi t	CMV		
53	Referral	Broncolitis dd Pneu	Anemia normositik normokromik			
108	Self adm	Ketoasidosis diabe	Severe sepsis	DM tp I	GIT bleeding	
	Transfer	Hydranchephaly post VP shunt				
54	Referral	CMV	Holoproenceph	Riw hipernatrei	Spina bifida	
58	Self adm	Pneumonia				
106,5	Referral	PJB asianotik				
84	Self adm	PJB asianotik				
115	Self adm	SNRS	HT st II	Hipoalbuminen	Claukoma ec ESO	
146	Self adm	Organik mentol dis	Depresi berat tanpa psikotik dd Afektif organik			
87,5	Referral	PJB asianotik				
163	Self adm	Neuropsikiatri lupu	SLE berat	Gangguan mental organik		
156	Transfer	Nefritis lupus	HT st II	Probable Dengue		
85	Referral	Angioedem ec drug allergy (amoxicilin)				
52	Referral	PJB sianotik	Pneumonia			
131	Referral	DHF gr II	Efusi pleura	GIT bleeding		
82	Self adm	Uncontrolled epile	Laringomalacia	Pneumonia	GDD	
122,5	Referral	DHF gr III				
72	Referral	Obs Vomitus profu	DCA tanpa dehi	VSD	Hypotyroid	Down Syndrom

60	Referral	Pneumonia	Epilepsy uncon	Cerebral palsy	CMV	
124	Self adm	Severe SLE	Nefritis Lupus			
128	Self adm	Vomitus Profuse dg dehidrasi tak berat				
58	Self adm	Vomitus Profuse d	Congenital Abn	Epilepsi terkon	Severly Stunted	
144	Referral	DHF	Riw ALL-SR			
98,5	Referral	SN resisten steroid	Pneumonia	HT gr II	TB paru	ISK
62	Self adm	Pneumonia				
49	Self adm	Kolestasis Intrahep	Down syndrom	PJB sianotik		
61	Referral	CMV	Kolestasis intra	Hernia Umbilikalis		
114	Referral	TB abdomen dd lin	Efusi pleura bilateral			
48	Referral	Pneumonia	Hydrochepali s	DNR	CTEV bilateral	
133	Self adm	CKD st V on HD	Hemophisis ec	HT st II	Edema paru	
100	Self adm	PJB sianotik				
96	Self adm	PJB asianotik				
155	Referral	DF	MVP			
98	Referral	RFA dd vira infecti	ITP			
155,5	Self adm	Nefritis HSP mg ke	HT st II	ESO streroid susp		
51,5	Referral	Congenital adrenal	Hiponatremia berat			
150	Referral	DHF gr II				
56	Self adm	Pneumonia berat	Sepsis	ARDS moderate	Asidosis respirat	DCA tanpa dehi
97	Referral	PJB asianotik				
	Self adm	SN kongenital	Hipotiroid kong	ISK	Fimosis	
70	Referral	Pneumonia	Multiple konge	GDD		
	Referral	DSS	GIT bleeding ec	Efusi pleura	Edema pulmo	
153	Self adm	Dyspepsia pro endoskopi				
132	Referral	DSS	Efusi pleura			
72	Self adm	Hipoalbuminemia	Kolestasis intra	CMV	Acites	
62	Referral	Kolestasis ekstrahe	Anemia hemoli	RFA	Pneumonia	DIC
50	Referral	Pneumonia	Sepsis	ME dd encephalopaty sepsis		
	Referral	DSS hr 7	DIC	Sepsis	GIT bleeding	Asidosis metab
136	Referral	Intracranial bleedi	HT st II	GIT Bleeding ec	Anemia gravis e	Dehidrasi tak be
134	Referral	DHF gr I	Gizi buruk tp m	Anemia		
134	Referral	DSS hr 5	GIT Bleeding	Efusi pleura		
82	Self adm	PJB asianotik				
127	Referral	CKD st V	Anemia mikrosi	HT st II	Encephalopathy	Edema Intracer
159	Referral	GNAPS dd SN	HT st II			
115	Referral	Uncontrolled epile	Cerebral palsy tetraparese spastik			
	Referral	Pneumonia	DM tp I			
	Referral	CAP	Kolestasis	Tonsilitis kronis	Riw urtikaria	
88	Self adm	Nyeri abdomen ec	Pankreatitis akut			
60	Referral	PJB sianotik	Pneumonia	GDD		
136	Self adm	DSS overcome hr 5	Efusi pleura			
108	Referral	DSS not overcome	Efusi pleura	Ascites		
132	Self adm	Sindrom nefrotik d	ODS glaukoma	Riw HSP		
157	Referral	DSS overcome	GIT bleeding	Efusi pleura		
	Referral	PJB sianotik	Sepsis	Pneumonia	Aki fase failure	Diare
134	Self adm	Typoid Fever	DF			
168	Transfer	Hemophisis	PJB asianotik			

159	Referral	Subdural hematom	Edema cerebri	Hemiparesis	Meningoenceph	Torticollis colli c
87	Referral	PJB asianotik				
48	Referral	Kolestasis ekstrahe	CMV	FTT		
152	Referral	Meningitis viral sus	edema cerebri	Stomatitis	Phlebitis	
	Referral	DHF gr I	HT st II	Hypoalbumin	Myocarditis dengue	
78	Referral	Vomitus tanpa tan	Post eksisi encephalocele			
149	Referral	Hemangioma infar	PCP			
67	Referral	Meningoencephali	DCA tanpa dehi	GDD		
69	Self adm	Pneumonia	CMV	ISK		
91,5	Self adm	Massa pons ec glioma dd abses cerebri				
114	Self adm	SNRS	Hypoalbumin			
82	Self adm	B20	Pneumonia	Diarrhea	ISK	Flebitis
49	Referral	PJB sianotik	Pneumonia			
115	Self adm	Epilepticus	Tonsilofaringitis			
100	Self adm	Pneumonia	PJB asianotik			
113	Referral	HSP	Nefritis HSP			
92,5	Self adm	DCA tanpa dehidra	Hydrocephali p	Hernia scrotalis		
113	Self adm	Shock hipovolemik	Diabetic Ketoasidosis			
133	Self adm	PJB asianotik				
82	Self adm	PJB asianotik				
92	Self adm	PJB asianotik				
59	Self adm	Kolestasis ekstrahepatal dd intrahepatal				
58	Referral	Prolonged fever	Down syndrome			
157	Referral	HT emergency	Susp edema pa	CKD st V on CAPD		
64	Referral	Kolestasis ekstrahepatal dd intrahepatal				
112	Self adm	ISK kompleks	Neurogenic bla	RTA		
121	Referral	SNRS				
81	Referral	CMV	Pneumonia	DCA dg dehidra	Prolonged Fever	
87	Referral	Resp distress	Pneumonia	pper GIT bleedi	sidosis respirato	Atelektasi Paru
68	Referral	Prolonged Syok	Epilepticus	GIT bleeding	Sepsis	DCA
135	Referral	DSS not overcome	Efusi Pleura	Edema Pulmo		
151	Referral	Susp adhesi post la	Polypgaster susp			
143	Self adm	Severe SLE	demam neuropenia			
82	Referral	Pneumonia	Sepsis	Prolonged Feve	Cerebral palsy tp	West syndrome
96	Referral	Dehidrasi tak berat	Gastritis dd abc	ISK		
94	Self adm	PJB asianotik	Epilepsi GTC terkontrol			
114	Self adm	SNRS				
108	Self adm	PJB asianotik	Thalasemia mayor			
46	Referral	Pneumonia				
112	Self adm	Morbili	Pneumonia			
55	Self adm	Sepsis	Phymosis	ISK	PJB asianotik	
110	Referral	PJB asianotik				
142	Self adm	Hematemesis mele	HT portal	Anemia mikrosi	PJB asianotik	
	Referral	PJB sianotik	Riw shock kardiogenik			
102	Referral	PJB sianotik	Pneumonia	Shock kardiogenik		
137	Self adm	HT	CKD on CAPD	Seizure ec susp HT		
120	Referral	DSS				
125	Self adm	PJB asianotik				

103	Referral	Ensefalitis viral dd	DCA tanpa dehi	GDD	Riw status epileptikus	
96	Self adm	PJB sianotik				
85	Self adm	PJB asianotik				
61	Referral	Kolestasis Intrahepatal dd ekstrahepatal				
83	Self adm	DCA ec susp rotavi	Epilepsi GTC			
80	Referral	CP spastik tetra pa	Prolonged feve	West syndrome	Pneumonia	
57	Self adm	DF hr V	PJB asianotik			
122	Self adm	Severe SLE	Nefritis Lupus	HT terkontrol		
71	Referral	DHF gr III hr V				
76	Referral	Pneumonia	Cerebritis	CMV	Tetraparesis spastik	
137	Referral	HT emergency	Retinopathy HT	Acute on chron	Obs kejang ec H	Anemia post tra
60	Referral	Pneumonia	DS dd hypotiroid			
133	Transfer	Chepalgia kronis	hidrocepalik ob	Shock neurogenik		
153	Referral	HT emergency	CKD st V	Obs retinopati	anemia on CKD	
98	Self adm	Hipoalbuminemia	SNRS	Anemia of chro	Pioderma susp	
160	Referral	Multipel athralgia dan myalgia dd juvenile rheamatoid arthritis				
133	Self adm	Ketoasidosis diabe	Nefritis			
83	Self adm	Pneumonia	DCA tanpa deh	ppert Syndrom	Milliaria Rubra	
114	Referral	DF dgn warning sig	Efusi pleura			
154	Referral	Nefritis HSP susp	DCA tanpa dehi	HT gr I		
100	Self adm	PJB asianotik				
91	Referral	Foreign body in GI	Soft tissue infection due to foreign body			
65	Self adm	PJB sianotik				
171	Transfer	PJB asianotik				
70	Referral	Pneumonia	Blepharitis	DS	Hipotiroid konge	PFO
126	Self adm	DF	Palato schizis	Caries dentist		
179	Referral	DHF	Shock hipovole	Efusi pleura		
64	Referral	DHF gr II dg overload cairan				
115	Referral	ME	DCA			
53	Self adm	Hemiperesis dextra	Enchephalitis s	Hydrocephalus	IVH	Edema cerebri
118	Referral	Ensefalitis vial dd	Otitis media su	Epilepsi GTC belum terkontrol		
157	Self adm	SNRS	Hiperkalemia	AKI fase injury	HT st II	
130	Self adm	PJB asianotik				
154	Referral	Varises esofagus gr III				
67	Referral	Urtikaria	Hidrocephalus	GDD		
100	Self adm	PJB asianotik				
74	Self adm	PJB asianotik				
70	Self adm	Sirosis hepatis	Ascites permag	Sepsis		
	Transfer	Appendicitis perfo	Sepsis	Upper GIT blee	Hematuria es susp traumatic	
85	Referral	Pneumonia	Shock septic	DCA	Asidosis metabolik	
137	Self adm	Hipersplenisme	Varises esofagu	GIT bleeeding		
	Referral	Shock sepsis overc	Sepsis	Me bacterial	DCA tanpa dehic	Asidosis metab
	Referral	Spontaneos tensio	Pneumonia sev	DCA tanpa dehidrasi		
120	Referral	PJB sianotik				
56	Referral	ME	Pneumonia			
73	Referral	SE overcome	ME bacterial dc	Pneumonia	Shock septic	GDD
86	Self adm	CMV	GDD	Mikrocepal	PJB asianotik	
64	Referral	PJB asianotik				

90	Referral	PJB asianotik				
90	Self adm	Asma serangan berat				
166	Self adm	DF hr 3				
126	Referral	Typoid Fever				
52	Transfer	Sepsis	Hidrocephalus	Exposed VP shunt		
166	Self adm	SLE nefritis	Neuropsikiatris	Hyperglikemia eceso steroid ulkus diabetes		
92	Self adm	Riw status epilepti	Susp glioma dd	Upper GIT bleeding		
	Self adm	PJB asianotik				
112	Self adm	PJB sianotik				
105	Referral	PJB asianotik				
137	Self adm	Severe SLE	Nefritis SLE	AIHA	Riw Efusi pleura	
	Self adm	GIT bleeding ec ru	Sirosis hepatis	HT pulmonal		
61	Referral	ISK	Larsen syndron	PJB asianotik	Pneumonia	Phymosis
54	Self adm	PJB asianotik				
149	Transfer	Pneumonia	PJR			
64	Referral	ISK kompleks	Hydronephrosis			
176	Referral	Shock hipovolemik	DCA dehidrasi b	Sepsis	Ensefalitis viral c	Pneumonia
160	Self adm	Ruptur varises eso	Gizi buruk tp m	Marfan Snyndrome		
146	Referral	DSS overcome	GIT bleeding	Efusi pleura	Expanded Dengu	Asidosis metab
133	Referral	CKD st V	Anemia on CKD			
115	Referral	DSS	GIT bleeding ec	Encephalopati I	Edema pulmo	Efusi Pleura
94	Self adm	PJB asianotik				
102	Referral	DHF gr I	Febris H5	PJB asianotik		
53	Referral	Pneumonia	PJB asianotik			
51	Referral	PJB asianotik	usp ODS Kataral	Hydrocele	ISK	Diaper rush
131	Referral	Me bacterial dd viral				
122	Referral	DMD				
95	Self adm	PJB asianotik				
94	Referral	PJB asianotik				
100	Self adm	Pneumonia dg SN	ten Steroid Scal	HT		
130	Referral	DHF gr II				
46	Transfer	Hischprung diseas	oilier type II A p	DS	ɔspadia dgn phym	PJB
52	Referral	Spasme infantile dd epilepsi				
64	Self adm	Pneumonia	buruk tp maras	Phymosis	ɔCA dgn dehidrasi	
71	Referral	Kejang demam kor	buruk tp maras	ɔ gross matur delay		
6	Referral	laringomalacia	palsy tetrapares	Pneumonia	Mikrocepal	
55,5	Referral	Kolestasis ekstrahepatal dd intrahepatal				
58	Referral	Kolestasis intrahap	A tanpa dehidr	ormositik norm	i buruk tp marasmik	
98	Self adm	Vomitus Profuse d	ISK			
110	Self adm	B20	ere sekundum, cor pulmonale chronic			
142	Referral	Ig A Nefropathy				
118	Referral	DMD				
59,5	Self adm	Sirosis dg ascites permagna				
150	Referral	PJB asianotik	ISK			
83,5	Self adm	CMV	Tetraparesis			
	Referral	Upper GIT bleedin	fusi pleura dextr	Hipofolsemia	CA tanpa dehidra	Pediculosis
119	Referral	PJB asianotik	Gizi buruk tp marasmik			
81	Referral	DSS	Efusi pleura	eeding ec stress ulcer		

49,5	Referral	DCA dehidrasi tak l	Anemia normo:	Susp Hypertropic pyloric stenosis		
80	Self adm	PJB asianotik	nital rubella syn	Mikrocepal		
73,5	Self adm	PJB asianotik				
	Referral	Peritonitis generalist				
57	Self adm	PJB sianotik	CTEV	CRS	Hernia umbilikalis	
125	Referral	DSS overcome hr 5	ding ec trombositopenia			
163	Referral	SNRS	Rhinofaringitis akut			
	Referral	Sepsis berat	GIT bleeding Su	Efusi pleura	Shock septic	nemia micro-hyp
	Self adm	Pneumonia				
122	Self adm	Severe SLE dalam p	Nefritis SLE	HT		
82	Self adm	PJB asianotik				
64	Self adm	Sirosis hepatis	HT portal	nemia pro transfusi albumin		
90	Transfer	Pneumonia	Hydrocephalus	Flebitis	i buruk tp marasmik	
76	Self adm	CMV	GDD	buruk tp marasmik		
72	Referral	Laringomalacia	buruk tp maras	tetraparese spa:	GDD	Pneumonia
63	Self adm	Pneumonia	PJB asianotik			
169	Referral	PJR				
122,5	Self adm	epilepsy	LBP	Meduloblastoma	Hidrocefalus	ISK
155	Referral	Kista choledocal	Kolestasis intrahepatal			
65	Referral	Upper airways obs	buruk tp maras	HAP		
84	Self adm	Edema cerebri	Vomit profuse (	B20	Flebitis	
69	Self adm	Pneumonia	Cerebral Palsy			
154	Referral	Hemiperesis sinistı	PJB asianotik	Sepsis		
97	Self adm	PJB asianotik	HT pulmonal	HAP		
149	Self adm	Gastritis	TB paru	Atrofi cerebral	SLE	
76	Self adm	PJB asianotik				
74	Self adm	Vomitus Profuse ta	Stomatitis	TB paru	B20	PCP drug induc
156	Referral	DHF gr III	Efusi pleura	Ascites		
83	Self adm	Ascites ec susp chr	Hypoalbumin	Kolestasis extra	DCA	ISK
134,5	Referral	SLE				
64,5	Referral	Encephalitis baktei	Sepsis	DCA tanpa dehi	Riw status epilep	Bronkiolitis
145	Self adm	SNRS	Hipoalbumin	a ec susp steroid induced		
84	Self adm	Kejang demam kompkleks ec RFA				
143	Self adm	PJB asianotik				
83	Self adm	PJB asianotik	epsi GTC terkontrol			
155	Self adm	CKD st V on CAPD	Edema pulmo	Anemia	HT st II	Kardiomyopath
80	Referral	Prolonged fever	DCA	nemia mikro-Hipo		
132	Referral	DHF gr III	Thalasemia			
	Referral	Me bacterial dd vi	inusitis maxilaris			
143	Self adm	PJB asianotik				
145	Self adm	PJB asianotik	HT pulmonal			
152	Referral	Leptospirosis	AKI	Trombositopen	Anemia mikro-hi	Skabies
73	Self adm	PJB asianotik				
	Transfer	FR angulus mandib	edema cerebri	Ruptur diafragr	Atelektasis pulmo sinistra	
127	Self adm	PJB sianotik				
154	Referral	SNRS	Dengue fever	HT st I	Hepatitis	
74	Referral	Kolestasis ekstrahe	s dg hidronefro	Thalasemia		
80	Self adm	PJB asianotik	HT pulmonal	DS		

120	Self adm	Paraparese flaccid	e muscular dysthropy			
75	Self adm	Pneumonia	BPD			
69	Self adm	CMV	Common cold	GDD		
145	Self adm	Hyperviskositas sy	SNRS			
146	Self adm	PJR	Miliaria			
123	Self adm	PJB asianotik				
103	Self adm	PJB sianotik				
	Transfer	Shock sepsis	Probable dengu	KDS		
80	Self adm	Dengue fever	Anemia	CP dd pneumonia		
99	Self adm	PJB asianotik	HAP			
104	Self adm	Anemia gravit on C	CKD st V			
111	Referral	Status epileptikus	Encephalitis			
130	Referral	DSS	GIT bleeding	Efusi pleura		
140	Self adm	Varises esofagus g	portal non sirosis			
	Transfer	Encephalitis	ute Infark Cere	Phlebitis		
59	Self adm	Pneumonia	CMV	Encephalitis	Edema cerebri	
124	Self adm	Hematoschezia ec	Varises esofagus			
175	Referral	DF				
62	Self adm	Atresia bilier susp	SBP	Post Kasai		
65	Self adm	Atresia bilier type I	SBP			
70	Referral	Bronchitis dd pneumonia				
62	Referral	Epilepsy tak terkor	Meningocele	Sepsis	Palatoschisis	
145	Self adm	Nefritis lupus	HT gr I			
136	Self adm	Severe SLE	Retinopathy SLE	Susp CMV		
110	Self adm	PJB asianotik				
79	Self adm	PJB asianotik				
111	Transfer	Corpal kacang bcronkus dex				
121	Self adm	Melena ec ruptur	Gastritis	anths syndrome		
145	Referral	Foreign body (jarum pentul)				
125	Self adm	Candidiasis	ISK			
155	Self adm	ME viral dd bakter	buruk tp marasmik			
150	Self adm	VES bigemini	Severe SLE	Nefritis SLE		
140	Transfer	AKI fase failure	Sepsis membaik	Shock Septic	CA tanpa dehidra	ISK
76	Transfer	Encephalitis viral	A dehidrasi ber	Sepsis	sidosis metabolik	
73	Referral	DHF gr II	usp typoid feve	Edema Pulmo	Efusi pleura	
85	Referral	PJB asianotik				
57	Referral	Pneumonia	Sepsis	Pertusis		
86,5	Self adm	Pneumonia berat				
139	Referral	Probable dengue				
55	Referral	PJB sianotik	Pneumonia			
55	Transfer	Pneumonia				
69	Referral	Impetigo Bulosa	Miliaria			
59	Referral	PJB asianotik				
92	Referral	Kolestasis ec intrahepatal dd ekstrahepatal				
87	Self adm	PJB asianotik				
82	Self adm	PJB asianotik				
55	Referral	Shock kardiogenik	HAP	PJB asianotik	Disentri	emia normo-nor
142	Self adm	SNRS	Hypoalbumin			

	Self adm	PJB asianotik	DS			
102	Self adm	DHF gr II	AKI			
134,5	Referral	SLE	HT gr I			
130	Referral	Kejang demam kor	ISK			
57	Transfer	Pneumonia	Sepsis	Laringomalacia	Hidrocefalus	oplasma konger
127	Referral	CKD st V	Anemia on CKD	HT st II	Severe Asidosis Metabolic	
135	Self adm	DMD				
139	Referral	DHF				
51	Self adm	Pneumonia	Miliaria	CLD		
	Self adm	Asthma Bronciale	Pneumonia			
53	Self adm	PJB asianotik	lypoplasia pulm	ypotiroid subklir	DS	Pneumonia
48	Referral	Pneumonia				
106	Self adm	B20	Pneumonia			
114	Self adm	CKD st I	SN relaps jaran	Hipokalemia		
137	Self adm	PJR				
132	Self adm	DHF gr I				
97	Self adm	Viral exantina	buruk tp marasmik			
126	Self adm	Gastritis				
147	Self adm	PJB asianotik				
82	Transfer	Vomitus Profuse d	pilepsi terkontr	sefalus post vp	Edema cerebri	
67	Referral	Kolestasis ekstrahe	Susp sirosis hep	Susp ailagile syndrome		
	Self adm	DCA dehidrasi tak berat				
156	Self adm	HT st II	CKD st III	Anemia on CKD	Retinopati	
123	Self adm	Vomitus Profuse ta	Asma persisten	Ulkus Peptikum		
156	Referral	DSS overcome	Upper GIT blee	Efusi pleura	Ascites	
84	Self adm	Pneumonia	Ensefalitis HSV I			
157	Self adm	PJB asianotik				
64	Self adm	PJB asianotik	eksi rubella kongenital			
87	Self adm	PJB asianotik				
112,5	Transfer	Dengue Fever				
58	Referral	PJB asianotik				
139	Self adm	Severe SLE				
149	Self adm	DF dg warning sign				
71	Self adm	CMV	S esotropia infa	GDD		
60	Referral	PJB asianotik				
136	Self adm	HT	CKD st V	Anemia on CKD	efalopati hipertensi	
104	Referral	DHF gr I	Efusi pleura			
145	Referral	Nefritis SLE				
114	Referral	Ensefalitis bakteria	DCA dehidrasi tak berat			
92	Self adm	SN frequent relaps	Edema anasarka	HT st I		
	Referral	DSS overcome				
66	Self adm	ISK kompleks Ecoli	Spina Bifida	eurogenic bladder		
64	Self adm	Pneumonia	DS	GDD		
126	Self adm	Recurrent Bula Pul	ra superior pulmo sinistra			
94	Self adm	PJB asianotik				
102	Referral	PJB asianotik				
133	Self adm	TB Mesenterika				
65	Referral	Kolestasis Intrahep	Hypoalbumin			

50	Referral	DHF gr I	Ileus functional	HCRS dd imbala	Prolonged diare ec intol lemak d	
51	Referral	PJB sianotik				
	Self adm	PJB asianotik				
83	Self adm	PJB asianotik				
123	Self adm	Varises esofagus gı	Gastritis	Banths syndrome		
140	Referral	Upper GIT bleedin	emia ec blood loss			
154	Self adm	Gastro duedenitis				
102	Referral	CKS	edema cerebri	eeding ec stress	nemia mikro-hip	Rhinitis Acute
145	Self adm	CLABSI	Faringitis	CKD st V on HD	anemia on CKD	Fraktur femur
	Transfer	Shock hipovolemik	rises esofagus gı	HT portal		
58,5	Referral	Kolestasis Intrahepatal dd ekstrahepatal				
120	Referral	Recurrent abdomiı	Common cold			
81	Self adm	PJB sianotik				
103	Self adm	PJB asianotik				
96	Self adm	PJB asianotik				
126	Self adm	Dengue severe				
65	Self adm	ME viral dd bakteri	DHF	Encephalopati I	Trombositopeni	DCA tanpa dehi
113	Self adm	Hypoalbuminemia	SNRS	Glaukoma Ods		
144,5	Self adm	PJB asianotik				
	Self adm	PJB sianotik				
98	Self adm	ISK kompleks	Neurogenik bla	CTEV	Tetraparese spastik	
145	Self adm	Nefritis lupus	HT st II			
73,5	Referral	Pneumonia	Anemia micro-hipo			
118	Referral	DHF gr II	Efusi pleura			
113	Self adm	PJB sianotik	Atresia pulmonal post BT shunt			
	Referral	PJB asianotik				
129,5	Referral	DHF gr II				
158	Self adm	Dengue fever hr ke	AML dlm remisi			
125	Referral	Probable dengue	Thalasemia	Hemosiderosis		
130	Referral	DHF gr I	A tanpa dehidrasi			
63	Referral	Diare disentriform				
155	Referral	DHF gr I				
117	Referral	SIRS	Dehidrasi ec low intake			
68	Referral	Shock septic	Sepsis	Encephalopaty	SE overcome	ME viral
148	Referral	AKI FS failure ec tr	Gross hematuri	HT st II	Upper GIT Bleed	Asidosis metab
60	Referral	Pneumonia	ernia diafragm	A tanpa dehidr	GDD	
82	Self adm	Epilepsi	CMV	Cerebral palsy	GDD	
119	Self adm	Hemibalismus dext	Susp KIPI			
59	Self adm	Laringomalacia tp	Pneumonia			
149	Referral	GNAPS	PJB asianotik	HT st I		
90	Self adm	B20 on terapi ARV	idrasi ec low int	Susp pneumonia		
106	Referral	Gullian Bare Syndrome				
139	Referral	Pneumonia				
73	Self adm	Pneumonia	A tanpa dehidr	Epilepsi	Riw inf CMV	
70	Referral	DCA ec rotavirus dg dehidrasi tak berat				
53	Transfer	Pneumonia	Sepsis	Edema Pulmo	PJB sianotik	Anemia normo-
61	Self adm	Anemia ec susp he	is hepatis dg as	Riw SBP		
140	Referral	Acute abdomen ec susp peritonitis dd colic renal sp dd HSP				

70	Transfer	DCA dg dehidrasi tak berat ec susp rotavirus				
107	Self adm	SN relaps	RFA	Ascites		
122	Self adm	Hidrosefalus post vp shunt ec massa intrakranial				
81	Self adm	Hipoalbuminemia	HT portal	Kolestasis		
173	Referral	Hematoschezia				
135	Self adm	Severe SLE on prot	AIHA			
64	Referral	Prolonged fever	Synd larsen			
153	Referral	HSP	Nefritis HSP			
	Referral	PJB asianotik	DS			
5,5	Referral	Hypertiroid pyloric stenosis				
63	Referral	DHF gr II	Efusi Pleura			
111	Referral	DHF gr I	Efusi Pleura dextra			
97,5	Referral	Functional konstipation				
67	Self adm	PJB asianotik				
109	Self adm	DF with warning si	Varises Esofagus	Faringitis akut		
132	Referral	SVT				
82	Self adm	PJB asianotik				
121	Self adm	PJB asianotik				
100	Referral	SNDS	palbuminemia b	Hipokalemia	HT	
126	Self adm	Massa sella tursica	Prolaktinoma			
158	Self adm	Probable dengue	SLE berat	HT st II	is hepatis dg HT portal	
133	Referral	Nefritis HSP dlm protokol				
105	Self adm	PJB asianotik				
128	Self adm	SNH	Gangguan mental organik dd psikosis akut			
127	Referral	Epilepsi				
67	Referral	DCA dehidrasi tak berat				
105	Self adm	ME viral dd bakterial				
126	Referral	DSS	Efusi pleura	GIT bleeding	SN	
151	Self adm	Dengue fever	HT st II	SN		
158	Self adm	Anemia	HT st II			
117	Self adm	PJB asianotik				
103	Self adm	PJB asianotik				
58	Referral	Pneumonia	PJB asianotik	DS	Anemia	:A tanpa dehidra
133	Self adm	Respiratory distres	CKD st V	HT st II	Asidosis metabo	ARDS moderate
152	Referral	PJR				
57	Referral	Kejang tanpa dema	Phimosis	ISK		
94	Self adm	PJB asianotik				
86	Self adm	Epilepsi	Cerebral Palsy	GDD		
53,5	Referral	Obstruksi portal du	Pneumonia	lg dehidrasi tak	ISK	
158	Referral	CKD st V	HT st II	Edema Pulmo	Efusi pleura	
57	Referral	Pneumonia	PJB asianotik	Edema Pulmo	CMV	Plebitis gr III
65	Referral	Kolestasis ekstrahepatal dd intrahepatal				
58	Transfer	Kolestasis ekstrah	ernia umbilikalis			
88	Transfer	Peritonitis generali	Pneumonia			
51	Self adm	Shock sepsis	Sepsis	andibula bilater	ap parotitis bilate	Plebitis gr Iv
40	Referral	Pneumonia	DCA tanpa dehi	CTEV bilateral	PJB asianotik	
124	Self adm	SNRS	HT st II			
133	Self adm	PJB sianotik				

59	Referral	PJB sianotik	nemia mikro-hipo			
118	Referral	ME bacterial dd vir	Upper GIT bleeding			
98	Referral	ICH	palsy tetrapares	Hidrancepali		
74,5	Referral	Pneumonia				
68	Referral	DHF gr I				
110	Referral	KDK	Faringitis akut e	ISK suspicious	Phymosis	
145	Self adm	Parese nervus cran	Edema cerebri			
110	Self adm	PJB asianotik				
152	Self adm	SLE	Nefritis lupus	ISK		
157	Self adm	PJB asianotik				
95	Self adm	PJB asianotik				
55	Referral	Pneumonia	B dd infeksi kongenital			
127	Referral	GNAPS	Ht st I			
123	Self adm	SLE berat	Nefritis SLE	HT		
157	Self adm	CKD st V	HT	Anemia on CKD		
165	Referral	Dyspepsia ec susp	pro endoskopi			
136	Referral	DHF gr I	fusi Pleura dextra			
89	Self adm	PJB sianotik				
	Referral	PJB asianotik				
67	Self adm	PJB asianotik				
114	Self adm	Bronchiestasis dg i	Pneumonia	uis bar syndrome		
108	Self adm	DMD	ablitias intelektual			
82	Self adm	PJB asianotik				
56,5	Referral	Pneumonia	A tanpa dehidrasi			
56	Referral	ME	Pneumonia	DCA tanpa dehidrasi		
81	Referral	ME	pper GIT bleedir	ISK		
110	Referral	DSS overcome	Efusi Pleura	GIT bleeding	emia normo-normo	
60	Self adm	Pneumonia	PJB asianotik	CMV		
64	Referral	DHF gr I				
115	Self adm	PJB asianotik				
91	Referral	SN				
76	Referral	Kejang demam kompleks				
157	Referral	DSS	Efusi Pleura	Upper GIT bleeding		
66	Referral	CMV				
145	Referral	Multiple fracture	HT			
67	Self adm	CMV	TB paru on OAT			
60	Self adm	CMV				
124	Referral	PJB sianotik				
158	Self adm	Miliaria Krestalina	Anemia ec susp autoimun			
47,5	Self adm	IEM				
135	Self adm	Severe SLE	AIHA	Nefritis SLE		
80	Referral	Kejang demam kompleks				
156	Referral	DSS Reversal	Efusi pleura	GIT Bleeding		
98	Self adm	PJB asianotik				
125	Self adm	DSS hr 7	Upper GIT blee	Efusi Pleura dextra		
142	Referral	Efusi pleura sinistr	SLE			
112	Referral	Peutz jagger syndr	Ascites	Ileus mekanik e	Flebitis	Sepsis
87	Referral	GIT bleeding	emia normo-normo			

88	Self adm	PJB asianotik				
111	Self adm	PJB asianotik				
95	Self adm	PJB asianotik				
60	Self adm	Epilepsi blm terkor/entrikulomegali				
117	Referral	CKD st V	HT st II	Hypertensive heart disease		
67	Referral	Kolestasis Intrahepatal dd ekstrahepatal				
139	Self adm	CKD st V	Pro HD cath	SN	Anemia on CKD	
67	Self adm	PJB sianotik				
178	Self adm	DF H-4				
86	Self adm	B20	emia normo-nor	A tanpa dehidra	Hiponatremia	
146	Referral	SN	HT st II	AKI fase failure ec RPGN susp		
52	Self adm	Kolestasis ekstrah	ISK dalam terapi			
95	Referral	Pertusis	Pneumonia			
108	Self adm	PJB asianotik				
	Self adm	Pertusis				
109	Self adm	PJB sianotik	Hemophisis ec susp rupture of colateral artery pd pasien TOF			
	Self adm	PJB asianotik				
154	Self adm	Empiema				
65	Referral	DM tp I	Riw KAD	Sepsis	Pneumonia	
148	Self adm	AKI	Krisis HT			
138	Referral	GNADS susp dd SN				
166	Referral	CKD st IV	SN dd GNAPS	Obesitas	ISK	
80	Referral	DSS overcome	pper GIT bleeding			
105	Referral	DSS Reversal	Efusi pleura	Upper GIT bleeding		
67	Self adm	Pneumonia				
112	Self adm	CKD	neral bone disorder			
165	Self adm	Neuromyositis	Chepalgia			
130	Referral	Impatensi HD cath	CKD st V	Anemia on CKD	HT gr II	Epilepsi
117	Self adm	CKD st V	ISK			
69	Referral	ME viral dd bakteri	SE	CAP	leeding ec stress ulcer	
47	Self adm	DS	PJB asianotik	Kolestasis intra	FTT	
79	Self adm	Pneumonia	Diare persisten			
115	Self adm	Snake bite	Encephalopaty	GIT Bleeding		
63	Referral	ME viral dd bakteri	ma Subdural Frontal			
64	Self adm	Pneumonia	CMV	DS	PJB asianotik	
146	Referral	PJR				
133	Referral	Deuchen muscular distrophy susp				
123	Self adm	SLE berat	Nefritis SLE	Ht terkontrol		
149	Referral	GNAPS	HT gr I			
156	Self adm	Varices esofagus g	rises cardia gaster			
80	Referral	Drawning	Pneumonia			
63	Referral	Kolestasis ekstrahepatal ec atresia bilier				
79	Self adm	Hidrosefalus	CMV			
101	Self adm	Oedem anasarka e	A ec viral infection			
62	Referral	Varises esofagus				
63	Self adm	Sirosis hepatis	Anemia gravis	Impending decomp cordis		
148	Self adm	DF hr IV dg hemofilia				
54	Self adm	Kolestasis ekstrahe	PJB asianotik	emia normo-normo		

155	Self adm	AKI	HT	Anemia	Trombositopenia	
79	Self adm	PJB asianotik	lernia umbilikalis			
113	Self adm	KAD	DM tp I	Hiponatremia	GIT bleeding ec stress ulcer	
142	Self adm	Varises esofagus	PJB asianotik	portal non sirotik		
144	Self adm	Severe SLE protokc	ISK	Nefritis SLE		
138	Self adm	Ensefalopati Tifosa	Demam Tifoid			
60	Referral	Me bacterial dd vir	Epilepsi			
70	Referral	Kolestasis Ekstrahepatal ec atresia bilier				
	Referral	Enchephalitis viral	CAP ec e coli	Shock septic	Sepsis severe	:A tanpa dehidra
57	Self adm	TB paru	Pneumonia	nia dg hepatomegali		
	Transfer	Miokarditis	AKI			
76	Self adm	Pneumonia	PJB sianotik	DS		
74	Referral	Abses hepar				
119	Self adm	CKD st V	PJR			
100	Self adm	PJB sianotik				
110	Referral	CKD st V	Riw SN	Edema Pulmo	HT st II	Anemia on CKD
70	Transfer	Nefroblastoma				
151	Referral	PJR				
81	Self adm	PJB asianotik				
121	Referral	Hemiparesis	HT st II			
165	Referral	GBD dd myelitis	Post trauma occuli			
136	Self adm	SLE	Nefritis SLE	HT st II		
128	Referral	Hepatitis typosa dd	Typoid fever			
77	Self adm	B20	TB paru	Parotis		
82	Referral	KDK	GDD			
62,5	Self adm	Prolonged fever	Pneumonia	PJB asianotik		
57	Referral	Kolestasis Ekstrahe	CMV			
47	Transfer	Kolestasis ekstrahe	Konjungtivitis o	DS		
67	Self adm	Pneumonia				
96	Self adm	PJB sianotik	DS			
117	Referral	Penkes ec kista cav	GNA	HT st II	ISK	Upper GIT blee(
141	Referral	Enchephalitis dd ei	Hipocalsemia			
73	Referral	SN	Hipoalbuminemia berat			
148	Referral	Neuropsikiatri lupu	GIT bleeding ec	Nefritis lupus		
111	Self adm	Nefritis HSP	Ods steroid ind	lg dehidrasi tak	AKI fase injury	
85	Self adm	PJB sianotik				
159	Self adm	PJB sianotik				
121	Self adm	DCA dehidrasi tak l	AKI			
50	Referral	Dehidrasi tak berat	Fistel peristoma			
130	Referral	Post external venti	Hydrocephalus	Edema Cerebri		
128	Referral	PJB sianotik				
127	Referral	DHF gr III	Efusi pleura bilateral			
112	Referral	Enchepalitis dengu	Sepsis	Shock septic	leeding ec stress	Edema paru
140	Referral	PJB asianotik	Scabies			
94	Self adm	Vomitus Profuse d	Epilepsi dd impatensi vp shunt dd ISK			
82,5	Self adm	Illeus obstruktif ec	HAEC			
113,5	Self adm	Pneumonia	Sepsis	Shock septic		
52	Transfer	Spinal muscular ati	Pneumonia ec HAP membaik			

59	Referral	HAP	Prolonged feve	DS	irschprung diseas	PJB asianotik
155	Self adm	Chepalgia kronik intermiten ec neurocysticercosis				
70	Referral	Prolonged fever ec	Riw kejang demam sederhana			
70,5	Self adm	PJB asianotik				
120	Self adm	Nefritis HSP relaps	DCA dehidrasi tak berat			
155	Self adm	HT	:KD st V on CAPI	Edema pulmo		
102	Self adm	GBS tp AIDP sensorimotor				
69	Self adm	ISK	Faringitis akut			
57	Referral	DSS overcome	DCA dehidrasi tak berat			
113,5	Self adm	KAD	DM tp I			
164	Referral	Recurrent hipoglikemia ec hiperinsulinemia dd diabates melitus tp I dd tumor pitui				
58	Self adm	Spontaneos bacter	Anemia gravis kasai pd atresia		is hepatis dg HT portal	
137	Self adm	Ig A Nefropati				
62	Referral	Pneumonia				
60	Self adm	Tetraparese spasti	Susp infeksi intrauterine			
142	Self adm	CRS	ISK kompleks	idronefrosis st II		
66	Self adm	PJB sianotik				
136	Self adm	Gastritis erosif	bdominal epilepsi			
126	Self adm	Varises esofagus	HT portal			
111	Referral	Oedem cerebri				
113	Self adm	Vomitus berulang :	Hidrocefalus obs dg vp shunt			
99	Self adm	SN dependent ster	HT st II	Hipokalemia	ukoma induce steroid	
91	Self adm	PJB asianotik	Steiner syndrome			
84	Self adm	PJB sianotik				
86	Self adm	PJB asianotik				
122	Self adm	PJB asianotik	p Steiner syndrome			
79	Self adm	Epilepsi	CMV			
116	Referral	Upper GIT bleedin	Anemia normo-hipo			
87	Self adm	DF dg expanded dengue				
51	Transfer	Sepsis	Ulkus decubitu	Ventrikulo megali bilateral		
103	Self adm	Pneumonia	Epilepsi	pospadia hipofisis		
52	Referral	PJB sianotik				
95	Referral	DMD				
150	Self adm	Muscle spam ec hi	Riw SN			
146	Self adm	Severe SLE	ISK	PJR		
68	Self adm	Pneumonia	PJB asianotik			
116	Referral	LMN				
101	Self adm	PJB asianotik				
106	Self adm	PJB asianotik				
135	Self adm	Severe SLE	Nefritis SLE	Riw AIHA		
90	Self adm	Bronchiestasis	ieumonia bilater	PJB sianotik	i buruk tp marasmik	
60	Self adm	DCA ec rotavirus	Dehidrasi pd gizi buruk			
67	Referral	Pneumonia	onged fever ec (	DCA		
129	Self adm	Kolestasis ec stero	Riw trauma abdomen			
62	Referral	CMV	DS	Mikrosefal		
64	Self adm	DCA	Vomitus profus	PJB asianotik		
79	Self adm	Pneumonia	Ricket disease			
134	Referral	PJB sianotik				

127,5	Self adm	SN dependent ste	albuminemia b	Efusi Pleura		
70,5	Self adm	CMV	TB paru	GDD	ADS SNHL	
141	Self adm	PJB asianotik				
56	Referral	Pneumonia				
75	Self adm	Pneumonia	PJB asianotik			
57	Referral	Pneumonia	Laringomalacia			
51	Referral	PJB asianotik				
77	Referral	Pneumonia				
152	Self adm	Severe SLE	Nefritis SLE	ISK		
102	Self adm	PJB sianotik				
155	Self adm	Gastroduodenitis c	Cyclic vomitus syndrome			
49	Transfer	PJB sianotik				
128	Self adm	Anemia on CKD	CKD st V	Varises esofagus ec hipertensi porta		
160	Referral	PJR				
	Referral	ME bacterial dd vir	GIT bleeding	Candidiasis oral		
158	Referral	SLE	Kardiodilatasi	fusi Pleura dextra		
82	Referral	DCA tanpa dehidra	Phimosis	ISK	Ileus fungsional	
80	Referral	Epilepsi GTC	tetraparese spa	D SNHL profoun	TORCH infect	GDD
146	Self adm	PJR	Edema pulmo			
160	Referral	SN dependent steroid				
58	Referral	PJB sianotik				
125	Referral	Epilepsi blm terko	ilepsi dd konvulsi disosiasi			
69	Self adm	Kolestasis ekstrahe	Sirosis hepatis (	Hypoalbuminer	Anemia normo-normo	
134	Self adm	JIAS				
96	Self adm	PJB asianotik				
105,5	Referral	CSWS	iw trauma kepala			
56	Transfer	Atresia bilier tp III	emia normo-normo			
53	Self adm	Pneumonia	Spinal muscular atrophy tp I			
	Referral	DSS overcome	Efusi pleura	pper GIT bleeding		
148	Self adm	PJB asianotik	HT pulmonal			
69	Referral	Pneumonia	PJB asianotik			
	Referral	PJB asianotik	DS			
85,5	Self adm	PJB asianotik				
137	Self adm	DCA tanpa dehidra	Thalasemia	Anemia on Thalasemia		
	Referral	ME viral dd bakter	itatus epileptiku	Sepsis	CP	DCA
56	Self adm	Pneumonia	PJB asianotik			
62	Self adm	Kolestasis ekstrahepatal ec atresia bilier dd kolestasis intrahepatal				
158	Referral	PJB asianotik	Asma	Dyspepsia	Faringitis akut	
59	Self adm	CMV	CRS	PJB asianotik		
65	Referral	CMV				
75	Self adm	Pneumonia	PJB asianotik	DS		
69	Self adm	Pneumonia	PJB asianotik			
130	Self adm	CKD st V	HT gr II			
124	Referral	DSS overcome H-5	fusi pleura dextra			
61	Referral	Pneumonia	PJB asianotik			
79	Referral	DCA dengan dehid	yndrome dg gizi	CMV		
68	Self adm	Wheezy Infant dd bronchiolitis				
62	Referral	Me bacterial dd viral				

105	Referral	DCA tanpa dehidrasi				
59	Referral	CAP				
100	Self adm	SNDS	Gastritis			
156	Self adm	PJB sianotik				
80	Referral	CAP	paru dl terapi bl V			
129	Self adm	Hematoshezia	Hidrosefalus	Astrosytoma dl radioterapi		
100	Self adm	PJB asianotik				
68	Referral	Prolonged fever	Pneumonia			
70	Referral	Pneumonia dd PCF	HIV dlm terapi			
130	Referral	DM tp I				
69	Referral	PJB sianotik				
50	Self adm	Hidronefrosis Sinistra ec pelvicoureteric junction obstruction				
140	Self adm	Supraventricular ta	Dyspepsia			
115	Referral	Fever of unknown	A tanpa dehidrasi			
147	Self adm	Severe SLE	PJR			
143,5	Self adm	SNRS	CKD st III	ipoalbumin berat		
86,5	Self adm	CAP dd TB infection				
68	Self adm	PJB sianotik				
151	Self adm	PJB asianotik				
	Referral	DM tp I	Hipertiroid	PJB asianotik		
87	Self adm	Epilepsi	CMV	Rubella	Cerebral palsy	
162	Referral	Hidrosefalus post vp shunt				
158	Referral	Severe SLE dl protc	HT st II	Nefritis SLE	Efusi pericardium	
152	Self adm	Severe SLE dl protc	Nefritis SLE	Neuropsikiatrik lupus		
110	Referral	Anemia on CKD	st V ec SN on C	HT st II		
54	Referral	Epilepsi	Hidrosefalus	ntrahepatal dd extrahepatal		
76	Referral	PJB asianotik	DS	GDD		
52	Referral	Laringomalacia dd laryngeal web				
107,5	Referral	PJB asianotik				
59	Self adm	Kolestasis ekestrak	PJB sianotik	A tanpa dehidra	Gizi buruk	ARDS severe
69	Referral	Pneumonia	Efusi pleura	Edema anasarka		
153	Self adm	PJB sianotik	HT st I tak berat			
69	Self adm	Pneumonia	PJB sianotik	Mikrosefal	andy walker variant	
74	Referral	Cerebral CMV dise	tetraparese spa	GDD		
57	Referral	Riwayat SE	Edema Cerebri	SDH	DCA	buruk tp maras
72	Self adm	Infeksi CMV				
75	Self adm	Cerebral CMV disease				
56	Referral	Pneumonia	Hernia umbilikalis			
59	Referral	Pneumonia				
100	Referral	DCA dg dehidrasi tak berat				
126	Referral	DSS	Encephalopaty	Efusi pleura	GIT bleeding	
104	Referral	PJB asianotik	s tanpa tanda dehidrasi			
	Referral	Asma intermitten	Rhinofaringitis akut			
70	Referral	CAP	Anemia Mikro-I	Sepsis	Dehidrasi tak be	Aki fase failure
	Referral	SE	ME viral	Pneumonia	GIT bleeding	
70,5	Referral	CAP	tetraparese spa	GDD		
149	Self adm	SLE	Nefritis SLE	HT st II	Common cold	Hipoalbuminem
132	Referral	PJB asianotik				

110	Self adm	Proloned fever ec	Typoid fever			
90	Self adm	Pneumonia	tetraparese spa	Mikrosefal	Epilepsi	
117	Referral	PJB asianotik				
151	Self adm	PJR				
84,5	Self adm	PJB sianotik				
55	Referral	DCA dg dehidrasi t	otiroid kongeni	DS	PJB asianotik	
69,5	Self adm	Cholestasis intrahe	Mikrosefali			
112	Referral	Nefritis HSP				
110	Referral	CKD st V	SN	HT st II		
114	Referral	Hematemesis mel	Varises esofagu	emia ec blood loss		
140	Self adm	Hematheresis et h	Gastritis erosif			
124	Self adm	Severe SLE	Nefritis SLE	HT terkontrol		
55	Self adm	DCA dg dehidrasi t	DS klinis	Hipotiroid	Diaper rush	PJB asianotik
140	Referral	Enchephalopaty de	DHF gr I			
53	Self adm	PJB asianotik				
147	Self adm	HSP	c pain ec HSP dd gastritis			
80	Self adm	PJB asianotik	Endocarditis			
61	Self adm	PJB asianotik				
69	Self adm	Paraparesis spasti	per motor neur	yelitis transversa		
118	Referral	Edema anasarka e	nefrotik resisten steroid			
47	Transfer	Kolestasis ec atres	Sindrom Alagille	Caroli disease	PJB asianotik	emia normo-nor
108	Self adm	PJB sianotik				
53	Referral	Kolestasis ekstrahe	Atresia bilier	emia normo-normo		
69	Referral	Meningoencephali	A tanpa dehidrasi			
44	Referral	Pneumonia	Palatoscisis	Spinal muscular atrofi tp I		
65	Self adm	CMV disease				
70	Self adm	Cerebral CMV disease				
55	Referral	Pneumonia				
140	Referral	SN resisten steroid	Vomitus tanpa tanda dehidrasi			
134	Referral	DMD				
128	Self adm	B20	Ektima	PCP	Gizi Buruk	
66	Referral	Pneumonia				
70	Self adm	Pericarditis TB	Feeding problem			
91	Referral	PJB asianotik				
57	Self adm	Gizi buruk tipe kwa	Hipoalbuminem	Anemia mikro-hipo		
103	Referral	Hypoalbuminemia	Hiponatremia	SNRS		
120	Referral	SE	PJB asianotik			
160	Referral	Epilepsi atonik				
111	Referral	SN				
80	Transfer	Massa intrabdome	Kolestasis	Hepatitis		
95	Self adm	PJB sianotik				
157	Self adm	Severe SLE	Nefritis SLE	HT st II	fusi pericardium	
152	Self adm	Nefritis SLE	Neuropsikiatri Lupus			
131	Referral	Dyspepsia ec gastr	Hematoschezia			
130	Self adm	Nefritis SLE	ISK			
157	Referral	Paraparesis inferio	Lesi LMN	ISK		
135	Transfer	Anemia gravis	GIT bleeding ec trombositopenia			
105	Referral	Pneumonia				

162	Self adm	Severe SLE	Nefritis lupus	Neuropsikiatrik	Hipoalbuminemi	Hipokalemia
60	Referral	Epilepsi	asidosis diabeti	DM tp I		
137	Referral	PJB asianotik				
132	Referral	PJB asianotik				
112	Referral	PJB asianotik				
150	Referral	PJB sianotik				
135	Referral	SLE	Nefritis SLE	ISK		
138	Referral	Epilepsi	Involuntary mo	Paraparese flaccid		
102	Self adm	PJB asianotik				
102	Self adm	Epilepsi	Syndrome lenn	Severe Conjuctive Imparrevent		
84	Self adm	PJB asianotik				
115	Referral	GNAPS	HT st II	CHF dg bradiaritmia		
59	Referral	PJB asianotik				
110	Referral	HT	KD st V on CAPI	SNRS		
152	Referral	SLE berat	Hipoalbuminemia			
169	Referral	PJB sianotik				
52	Referral	Infantile spasme ec congenital infection				
137	Referral	Severe SLE	PJR	ISK		
71	Referral	SN dependent steroid				
140	Referral	Hematemesis ec g	chezia ec kolitis dd polip			
153	Referral	PJB asianotik				
62	Referral	Ileus Paralitik	Sepsis	rolonged dengue		
	Referral	Pneumonia	DS	PJB asianotik	Sepsis	A tanpa dehidra
69	Referral	ME viral dd bakteri	Pneumonia	Anemia	Oedem pulmo	PJB sianotik
116	Referral	Tetanus	Caries dentist	Oral Trush		
80	Referral	Pneumonia	CP			
72	Referral	DHF gr III	Phymosis			
57	Referral	Pneumonia	PJB asianotik	DCA tanpa dehidrasi		
148	Referral	SLE	Dengue fever			
102	Self adm	PJB sianotik	Pneumonia			
73	Referral	Hidrosefalus post v	CMV			
136	Referral	Vomitus profuse	Hepatitis akut			
120	Self adm	B20	nia ec hematosh	Hematoshezia ec hemoroid		
109	Referral	PJB sianotik				
76	Referral	Shock sepsis	ISK	Pneumonia		
153	Transfer	HSP nefritis	ISK	drasi pada gizi buruk		
63	Referral	Osteogenesis impe	Commond cold			
112	Referral	Vomitus Profuse d	Hipoglikemia	Glaukoma		
65	Referral	Corpal jepit rambut gaster				
129	Referral	Varises esofagus	antys syndrome			
106	Referral	DSS	Efusi pleura			
73	Referral	DSS	Efusi pleura	Acites		
79	Referral	Shock sepsis	Sepsis	Ensefalitis viral	akut tanpa dehi	TB dlm terapi
120	Referral	Edema pulmo	DSS	Efusi pleura	Acites	Cardiomyopaty
74	Self adm	Penkes ec susp hidtatus epileptiku	Kipi Campak			
	Referral	Enchephalitis viral	Sepsis	Shock septic	ca tanpa dehidra	HAP
123	Referral	DSS	usi pleura bilate	Acites	GIT bleeding	
121	Transfer	DSS overcome				

134	Referral	DSS overcome	Efusi pleura			
160	Referral	HT st II	y glomeronefrin	AKI free failure	ailure ec edema		Efusi pleura
55	Self adm	Sepsis	Ht st II	PJB asianotik	Pneumonia	
68	Referral	ME	DCA	Syok Septic	Pneumonia	Anemia
133	Referral	Severe sepsis	Shock septic	DSS overcome	Edeme pulmo	Efusi pleura
	Referral	DSS overcome	Efusi pleura	GIT bleeding		
78	Referral	DSS overcome	ephalopathy der	Sepsis	Hipoalbuminemia	Efusi pleura
167	Referral	DSS overcome	Efusi pleura	Obesitas		
112	Referral	DSS overcome	usi pleura bilat	epper GIT bleeding		
100	Referral	DSS overcome	fusi pleura dextra			
75	Referral	Dengue fever				
65	Transfer	Pneumonia	Edema pulmo	PJB asianotik	DS	:A tanpa dehidra
152	Referral	Hematoshezia ec n	Anemia gravis			
110	Referral	Hemolitis Uremic S	cute renal failur	Encephalopaty	iponatremia bera	ISK
129	Referral	Encephalitis viral	ilepsi uncontrol	3antis Syndrome		
149	Referral	PJB sianotik	s tanpa tanda dehidrasi			
143	Self adm	Chest pain ec gastr	PJB asianotik	Varices esofagus	HT portal	
46	Referral	Hidrosefalus obstru	Apneu			
80	Self adm	PJB asianotik				
131	Self adm	Vomitus profuse ta	pilepsi abdominal			
69	Self adm	PJB asianotik				
66	Referral	PJB sianotik				
152	Self adm	Nefritis SLE	europsikiatri Lup	ISK		
158	Self adm	SLE berat	Nefritis SLE	HT st II	Efusi pericardium	
125	Referral	Gastritis	s tanpa tanda dehidrasi			
112	Referral	DMD				
122	Referral	DMD				
122	Self adm	PJB asianotik	DS	GDD		
139	Self adm	JIA	PJR	Prolonged fever	MAS	
147	Self adm	HSV				
75	Referral	Diare Kronis				
70	Referral	Pneumonia	CMV			
107	Referral	Measles	Pneumonia			
121	Referral	Enshefalitis	Edema cerebri			
45	Referral	Sepsis	Pneumonia	E bacterial dd viral		
	Referral	Pneumonia	Morbili			
62,5	Referral	Pneumonia				
57,5	Self adm	Ileus fungsional ec	Laringomalacia			
47	Self adm	PJB asianotik				
116	Referral	Barther syndrome	Hipomagnesiur	Cerebral palsy	Epilepsi	Dermatitis atro
150	Referral	Tetraparese spasti	Lesi LMN	Abses cerebri	PJB sianotik	nusitis kronis m
104	Referral	Neuroblastoma dg	bone metastase			
128	Referral	PJB asianotik	buruk tp marasmik			
85	Referral	PJB asianotik				
167	Referral	PJB asianotik				
147	Referral	Encephalopathy	HT emergency	SNRS	Common cold	
150	Self adm	Vomitus	RFA	PJB sianotik	Abses cerebri	
155	Referral	Nefritis SLE	ISK	Anemia normo	CKD	

148	Referral	PJB asianotik					
69	Referral	PJB asianotik					
158	Referral	PJB asianotik					
58	Referral	Kolestasis Intrahep	Commond cold	Miliaria			
157	Referral	PJB asianotik					
134	Referral	CKD st V	DCA tanpa dehi	HT St I	Renal Osteoclasi	Dispepsia	
93	Referral	Epilepsi	palsy tetrapares	Microcephaly	ıs tanpa tanda dehidrasi		
99	Referral	Asma intermitten	Epilepsi	GDD			
158	Referral	SLE berat	Nefritis SLE	HT st II	ͰEfusi pericardium		
49	Referral	Laringomalacia tp I	CTEV billateral	Shelldown hall	Hiperkalemia		
110	Referral	PJB sianotik					
64	Referral	Hipoalbuminemia	Sirosis hepatis	Gizi buruk tp m	CMV		
97	Referral	SN dependent ster	Commond cold	Iipoalbuminemi	HT St I		
134	Referral	Periodic paralytic	Hipocalemia				
152	Referral	Severe SLE	Nefritis SLE				
132	Referral	Epilepsi	Mikrocephal	Disabilitas	Oral candidiasis	Gizi buruk tp ma	
140	Self adm	Total AV block	t pemasangan PPM				
70	Referral	PJB asianotik					
130	Referral	PJB sianotik					
110	Referral	CKD st V	Ht st II	Anemia on CKD			
	Referral	SNRS	HT st I	Iipoalbuminemi	Atrofi Cerebri	buruk tp marası	
	Referral	Sepsis	emia heart dise	Edema pulmo	a enterocutan pc	Cardiomyopaty	
130	Referral	PJB sianotik	Commond cold	Gizi buruk tp marasmik			
158	Referral	ME viral dd bakteri	ydrocephalus ob	Edema Cerebri	Cauti	rointestinal blee	
75	Referral	Hipotiroid subklinis	Bisitopenia	GDD			
70	Referral	SNHL ADS	CMV	PJB asianotik			
	Referral	ME viral dd bakteri	Edema cerebri	Status epileptik	DCA dehidrasi ta	SIRS	
67	Referral	Prolonged fever	CMV	Anemia	GDD	ISK	
126	Referral	AKI FS failure	HT st I	Epitaksis anteri	Bradi Aritmia	SN	
146	Referral	Massa intracranial	abilitas intelektual				
	Referral	ME viral dd bakteri	Edema cerebri	Sepsis	Myelomeningitis	DCA tanpa dehi	
109	Referral	PJB asianotik	buruk tp marasmik				
57	Referral	Pneumonia	Hemangioma	PJB asianotik			
68	Referral	PJB sianotik	Pneumonia	Gizi buruk tp marasmik			
84	Referral	PJB sianotik	Gizi kurang				
64	Referral	Hipoalbuminemia	Kolestasis intra	Anemia mikro-ı	Hernia umbilikal	Gizi buruk tp m	
135	Referral	Ensefalopati hipert	Acute Glomeru	HT emergency	Status epileptikus		
70	Referral	Hipoalbuminemia	Ascites	HT portal	PJB asianotik	Gizi buruk tp m	
96	Referral	Asma intermitten	Rhinofaringitis	Gizi kurang			
73	Referral	Kolestasis ekstrahe	Sirosis hepatis	Epistaksis	Gizi buruk tp ma	Hipoalbuminem	
121	Referral	PJB sianotik					
103	Referral	PJB asianotik					
151	Referral	Nefritis SLE					
147	Referral	Upper GIT bleeding ec sirosis hepatis					
80	Transfer	Pneumonia	CMV	B20	Oral candidiasis	GDD	
75	Referral	Seizure with fever	SNHL	Phimosis	Epilepsi	PJB asianotik	
69	Referral	Me bacterial dd vir	DCA	Sepsis	Mikrocephal	GDD	
137	Referral	CIDP	Gizi buruk tp marasmik				

93	Referral	PJB sianotik					
77	Referral	PJB asianotik					
100	Referral	PJB sianotik	buruk tp marasmik				
70	Referral	Pneumonia	DS	PJB asianotik	Hipotiroid		
79	Referral	SNRS	Edema anasark	Epilepsi	Mikrocephal		GDD
141	Referral	SLE	SNRS	Hypokalemia			
107	Referral	Hematoshezia ec h	Prolonged diare				
110	Referral	CKD st V on CAPD	Encephalopath	HT emergency	SNRS		
57	Referral	PJB asianotik	Pneumonia	DS	Hernia umbilikalis		
127	Referral	Vomitus profuse					
141	Self adm	AV block	Post pemasangan PPM				
147	Referral	PJB asianotik					
71	Referral	PJB sianotik	DCA tanpa dehidrasi				
57	Referral	Kolestasis ekstrahe	PJB asianotik	CMV	Anemia hemoliti	Commoncold	
52	Referral	PJB asianotik	Pneumonia	DS	Gizi buruk tp ma	Hipotiroid subk	
85	Referral	Ensefalitis HSV	CP tetraparese	Epilepsi	Intoksikasi as valproat kronis		
93	Referral	Probable dengue	Hepatomegali				
152	Referral	Peritonitis induced	CKD st V on CA	HT st II	Hipokalemia	Anemia on CKD	
133	Referral	Peritoneal dyalsica	CKD st V on CA	HT st II	ISK		
142	Referral	PJB asianotik	PJR	Dyspepsia			
72	Transfer	Cerebral CMV dise	GDD				
	Referral	CMV	Headache				
77	Referral	PJB sianotik	DS				
70	Referral	CAP					
77	Referral	SN kongenital	Hipoalbumin				
110	Referral	B20	PH severe	buruk tp maras	Bronkiestasis		
	Referral	Asma intermitten serangan berat					
	Referral	PJB sianotik					
140	Referral	PJB asianotik					
115	Referral	PJB asianotik					
157	Referral	Varises esofagus g	HT portal	Gizi kurang	Anemia		
67	Referral	Hematemesis ec r	palsy tetrapares	Epilepsi			
124	Referral	Severe SLE	HT gr I				
97	Referral	Epilepsi	CP tetraparese	GIT bleeding			
147	Referral	Massa intracranial					
71	Referral	PJB asianotik					
160	Referral	PJB asianotik	Scabies				
137	Referral	PJB asianotik					
	Referral	PJB asianotik					
144	Referral	Iskemia ensefalopa	Epilepsi	Upper GIT blee	GDD		
147	Referral	Melena pd varises	HT				
117	Referral	DMD	GDD				
168	Referral	Upper GIT bleeding					
156	Referral	Varises esofagus g	Varises cardiac gaster				
78	Referral	Ruptur varises eso	Epilepsi	CP tetraparese spastik			
72	Referral	CMV	Hidrocefalus ob	Anemia			
152	Self adm	Neuropsikiatri SLE					
	Referral	SLE berat					

159	Referral	Abdominal pain dd	Obesitas			
68	Referral	Prolonged fever	Pneumonia	Laringo malacia	Cerebral CMV	Gizi buruk tp m
80	Referral	CMV	Epilepsi	West Syndrome		
	Referral	PJB sianotik	Scabies			
106	Referral	PJB sianotik				
46	Referral	Prolonged fever	DCA dehidrasi t	Appert Syndrom	PJB asianotik	ISK
108	Referral	Cardiomyopati dila	Oedem anasark	Gizi buruk tp marasmik		
76	Referral	Epilepsi	CP tetraparese	E bacterial dd viral		
58	Referral	Pneumonia	Hydrochele	DCA tanpa dehidrasi		
134	Referral	PJR				
	Referral	Melena ec colitis	Ulceratif dd chrone disease			
162	Referral	Nefritis SLE	DCA	ISK	HT st II	Hiponatremia
124	Referral	ME bacterial dd viral				
114	Referral	Epilepsi	Rhinofaringitis	GEFS		
	Referral	Bronchopneumonia				
86	Referral	PJB sianotik				
57	Referral	PJB asianotik	Gizi buruk tp marasmik			
65	Referral	ISK	ensi selang nefro	Commoncold		
61	Referral	Vomitus profuse ta	Abdominal epil	Gastritis erosif		
76	Referral	PJB asianotik				
126	Referral	Hematochezia ec colitis dd divertikel				
59	Referral	Hidronefrosis bilat	HT st I			
120	Referral	AKI FS failure	HT st II	Bradiarytmia	Gizi buruk tp ma	Cystitis
73	Referral	Sirosis hepatis	HT portal	oalbuminemia bi	buruk tp marasmik	
146	Referral	HT emergency	GNAPS	ISK	Enchepalopathy	
129	Referral	Vomitus profuse	Asma			
	Referral	Severe SLE	Neuropsikiatri S	Nefritis SLE	ISK	JIA
120	Referral	PJB sianotik	Gangren gigi			
138	Referral	KAD	DM tp I	ISK	Gizi kurang	
96	Referral	JIA	ISK			
116	Referral	Vomitus profuse	PJB sianotik	Abses cerebri		
87	Referral	Epilepsi	Cerebral palsy t	DS	Mikrocephal	Hipotiroid
75	Referral	SN kongenital	Rhinofaringitis	Hipokalsemia		
112	Referral	SN relaps	CKD st V	Anemia	Hipocalcemia	Hipokalemia
101	Referral	PJB asianotik				
129	Referral	JIA	Defisiensi vit D			
123	Referral	SLE berat	Nefritis SLE	ISK	Epilepsi	Anemia
85	Referral	GBS	KIPI			
130	Referral	DMD	Sirosis hepatis	Efusi pleura	Edeme pulmo	GIT bleeding
53	Referral	Atresia esofagus	Gizi buruk tp m	Mikrocephali		
	Referral	Kolestasis Intrahep	Anemia normo	Hernia inguinal	Hipoalbuminemia	
114	Referral	PJB asianotik	Rhinitis akut			
67.5	Referral	Edema cerebri				
53	Referral	Kolestasis intrahep	CMV	Anemia hemoliti	HIL	Defisiensi vit B
85	Referral	Hemiparesis sinistra akut				
153	Self adm	Gastritis	Varises esofagu	PJB asianotik	CA tanpa dehidrasi	
	Referral	Myelitis transfersa	AFP			
52	Referral	Pneumonia	Atresia esofagu	zi buruk marasm	Hiponatremia	Hipokalemia

74	Referral	DCA dehidrasi tak	Atresia ani	Upper GIT bleeding		
57	Referral	ME viral	Anemia normo	ernia Umbilikal	iestasis Intrahepatal	
67	Referral	GEFS Plus	KIPI	UTI		
65	Referral	Diare disentriform				
75	Referral	Sirosis hepatis	Hipoalbuminen	Gizi buruk tipe	HT portal	CMV
127	Self adm	PJB asianotik				
126	Self adm	PJB asianotik				
124	Referral	Anemia mikro-hipc	B20	Hematoshezia		
68	Referral	PJB sianotik	CAP			
69	Self adm	PJB sianotik				
75	Self adm	PJB asianotik	Rhinofaringitis	Gizi buruk tp marasmik		
84	Referral	Hidrosefalus obstr	mor fossa posterior			
121	Referral	GBS				
72	Self adm	PJB asianotik	GDD			
121	Referral	Prolonged fever				
161	Referral	Vomitus profuse t	bdominal epilep	Gastroduodenitis		
54	Referral	CAH	FTT			
88	Self adm	PJB asianotik				
	Self adm	PJB asianotik				
81	Self adm	PJB sianotik	Rhinofaringitis akut			
98	Referral	Vomitus Profuse d	Hidrosefalus	Edema Cerebri	Gizi buruk	Mikrosefali
	Self adm	PJB asianotik				
135	Referral	Epilepsi	Hemiparesis	Disabilitas Intel	Mikrocephal	Flebitis
132	Transfer	PJB sianotik				
150	Self adm	Nefritis SLE	HT			
152	Self adm	PJB asianotik				
115	Referral	MD dd becker dd c	Anemia mikro-l	Gizi kurang		
127,5	Self adm	PJB sianotik	Diffuse parench	ISK	Hipoalbuminemi	Gizi kurang
133	Referral	Encephalopaty	Dehidrasi pd gi	Epilepsi	Neurosklerosis	Hipertrofi gingg
144	Referral	Kolitis no spesifik				
132	Self adm	PJB asianotik				
90	Self adm	Atresia bilier post	Anemia normo	Hipoalbuminemia		
142	Self adm	PJB asianotik				
140	Referral	SLE berat	Nefritis SLE	Severly stunted	ISK	
100	Self adm	PJB asianotik				
62	Self adm	PJB asianotik				
	Referral	SN Kongenital	CKD st IV	Hipotiroid	Hipoalbuminemi	Anemia on CKD
127	Self adm	PJB asianotik	TB paru dalam	Gizi buruk tp marasmik		
151	Referral	Severe SLE on prot	ISK	Nefritis SLE		
130	Referral	PJB sianotik	HT st II	Gizi kurang		
125	Referral	SN	GNAPS			
162	Referral	SNRS	HT st I	Obesitas	Dispepsia	Hipoalbuminem
129	Referral	SLE berat	Neuropsikiatri l	Epilepsi		
68	Self adm	PJB asianotik				
131	Referral	Nefritis				
73	Self adm	PJB sianotik	Vomitus profus	dehidrasi tak berat		
88	Self adm	PJB asianotik				
	Self adm	PJB asianotik				

127	Referral	Abdominal pain dd gastritis				
148	Self adm	Epilepsi	GMO			
	Transfer	GBS post				
83	Referral	Pneumonia	B20	Laringitis candi	Gizi buruk tp ma	Anemia makros
115	Referral	Hipokalemik perio	Epilepsi	KIPI MR	Varicella dg infeksi sekunder	
105	Referral	ME viral	Pneumonia	Epilepsi	Shock septic tera	DCA tanpa dehi
	Referral	DCA dehidrasi bera	PJB asianotik	DS	AKI	
	Self adm	Pneumonia	CP tetraparese	Collaps orrofari	Gizi kurang	
109	Referral	Snake bite				
64	Referral	Pneumonia	PJB asianotik			
105	Referral	Hidrosefalus comn	Hipertiroid	Disabilitas Intel	SHL	Pneumonia
69	Referral	Epilepsi	Kejang demam	RFA		
133	Referral	PJR	Trapping pleura	Konsolidasi ple	Empiema pulmo	Gizi kurang
82	Referral	Cerebral CMV dise	Epilepsi	GDD		
67	Referral	Cerebral CMV dise	SNHL ADS seve	Gizi buruk tp m	GDD	
64	Self adm	PJB asianotik				
	Self adm	PJB asianotik				
142	Self adm	PJB asianotik				
111	Self adm	PJB asianotik				
86	Referral	PJB asianotik	CP tetraparese	GDD		
61	Referral	Dehidrasi tak bera	Commond cold	PJB asianotik		
111	Referral	B20	Muscle spasm	Bronchietasis d	Gizi buruk tp marasmik	
149,5	Referral	Dispepsia	SNDS	Shock Hipovolemik		
150	Referral	Gastritis				
172	Self adm	PJB asianotik				
97	Self adm	PJB asianotik	Gizi kurang			
103	Self adm	PJB asianotik				
	Self adm	PJB asianotik				
	Self adm	PJB asianotik				
	Referral	SN	Hipoalbuminemia			
91	Referral	SNRS	Hipoalbuminen	Sindrom Mallo	Disabilitas intelektual	
78	Self adm	PJB asianotik				
75	Self adm	PJB asianotik				
73	Referral	Pneumonia	CP tetraparese spastik			
76	Referral	SN Kongenital	Nefritis Intersti	Bronchitis akut	Gizi kurang	
71	Self adm	PJB asianotik				
100	Self adm	PJB asianotik				
77	Self adm	PJB asianotik				
97	Self adm	PJB asianotik				
	Self adm	PJB asianotik				
102	Self adm	SNRS	Hipoalbuminemia			
129	Referral	DMD				
	Referral	PJR	Edema Pulmo	Efusi pleura bila	Hipoalbuminemi	Anemia
163	Self adm	SLE berat	Nefritis SLE	Neuropsikiatrik	ISK	
52	Referral	Peritonitis	DCA dg dehidra	Prolapse stoma	Gizi buruk	Sepsis
	Self adm	Pneumonia	Corpal kacang di bronkus kanan			
	Referral	Drowning	Asidosis resp b	ARDS	AKI	
125	Self adm	PJB asianotik				

97	Referral	Shock hipovolemik	GIT bleeding			
95	Self adm	SNRS				
101	Referral	DMD				
72	Self adm	PJB sianotik				
	Referral	PJB asianotik	GDD			
150	Referral	Nefritis SLE	HT st II			
135	Self adm	PJB sianotik				
75	Self adm	SN Kongenital				
117	Referral	Vomitus dg dehidr	Gastritis	ISK		
68	Referral	DF dd viral	Overweight			
92	Referral	PJB asianotik				
142,5	Self adm	SLE berat				
88,5	Self adm	PJB asianotik				
63	Referral	Prolonged fever	PJB asianotik	Hernia Umbilik	Endocarditis infe	Pneumonia
48	Referral	AKI	Hidronefrosis b	ISK	PJB asianotik	CMV cerebral i
47	Referral	Pneumonia	Hipoglikemia	Stunted	Ateletaxis lobus supol pulmo de	
45	Transfer	Pneumonia	FTT	Laringomalacia		
97	Self adm	Varicella Zoster				
87	Referral	Guillan Bare Syndr	Alkalosis respir	Pneumonia		
116	Transfer	Septic atritis viral c	AIHA			
151	Self adm	Severe SLE dl protokol bl ke IV				
83	Referral	Kejang Demam	Faringitis Akut	B20 dl terapi		
135	Referral	Me bacterial dd vir	Sepsis	AKI	Epilepsi	Hematuria ec t
73	Self adm	PJB asianotik				
105	Self adm	PJB asianotik				
57	Referral	Kolestasis intrahep	CMV	nemia hemoliti	Pneumonia	Sepsis
89	Referral	Prolonged fever	CMV cerebral	Phimosis pro ci	Dca tanpa dehid	Diaper rush
	Transfer	Fistula enterokuta	UTI	Peutz Jagher Sy	Hipokalemia	Gizi buruk tp m
65	Self adm	Pneumonia	PJB sianotik	DCA tanpa dehi	Eritema nodasur	Gizi buruk tp m
	Self adm	CMV disease	Epilepsi	GDD		
80	Transfer	CMV cerebral	GDD	Gizi kurang		
66	Transfer	Pneumonia	CMV disease	tetraparese spa	ca tanpa dehidra	buruk tp maras
58	Referral	Kolestasis ekstrahepatal dd intrahepatal				
76	Referral	GERD	idrasi pd gizi bu	er Russel Syndrome		
58	Referral	Me bacterial dd vir	Epilepsi	Katarak Konger	CMV	Gizi buruk tp m
53	Referral	Kejang Demam	Laringomalacia	Epilepsi	ISK	
105	Self adm	Varicella	Epilepsi			
151	Self adm	Abdominal pain dd	Abdominal epilepsi			
89	Referral	Scarlet Fever				
87	Self adm	PJB asianotik				
	Self adm	PJB asianotik				
60	Referral	Pneumonia	GDD			
130	Referral	SLE berat				
88	Referral	Stroke iskemik	Parese N VI ec	Seizure ec TIK	Infaret cerebri	Atrofi Cerebri
155	Self adm	GBS tp bulbar				
102	Self adm	PJB sianotik				
55	Referral	Kolestasis Intrahep	Post kasai	Bronkitis akut		
	Referral	GBS	sidosis metabolik			

	Referral	Resp Failure crisis	CAUTI ec jamur				
147	Self adm	Nefritis SLE	Severe SLE	Arthritis	1yelitis transvers	Stomatitis	
131	Self adm	GNAPS	HT st II				
135	Self adm	PJB asianotik					
153	Self adm	Peritonitis	HT st II	CKD on CAPD			
111	Self adm	PJB asianotik					
144	Self adm	Gastritis	Asma				
119	Self adm	PJB asianotik					
104	Self adm	SNRS	Hipoalbuminemia				
	Self adm	PJB sianotik					
71	Self adm	Prolonged fever ec	Epilepsi	GDD			
91	Referral	PJB Asianotik					
52	Referral	Vomitus profuse ta	CMV	buruk tp maras	Pneumonia	astroentritis Aku	
	Referral	Pneumonia	Kardiomyopati	CMV disease	Severe SNHL	Gizi buruk tp m	
	Referral	Sepsis	Shock septic	ISK	Phimosis	Hipokalemia	
136	Referral	Autoimmune Ench	Stenosis Arteri	Gizi Buruk	HT St II		
66	Referral	Prolonged Fever	ebral CMV dise	SNHL moderate	KDK		
71	Referral	Peritonitis	Sirosis hepatis	PJB asianotik	i buruk tp marasmik		
51	Referral	Atresia coana bilateral pro repair					
149	Referral	CKD st V	HT st II	Edema Pulmo	ISK	SLE	
134	Self adm	Peritonitis on CAPD	CKD st V on CAPI	HT st I	Gizi buruk		
	Referral	Goldenhar Syndroı	PJB asianotik	olostomy pd atre	ISK	buruk tp marası	
107	Referral	DMD					
108	Referral	DMD					
154	Self adm	PJB asianotik	Gizi buruk				
53	Referral	SAH	SDH	Edema difus	Anemia Gravis	A tanpa dehidra	
92	Self adm	PJB asianotik					
90	Referral	PJB asianotik					
90	Self adm	PJB asianotik	al on permanen	GDD			
47	Referral	PJB asianotik	Pneumonia	FTT			
85	Referral	PJB asianotik	Gizi kurang				
132	Self adm	Varises esofagus p	lantys syndrome				
103	Self adm	SNRS	oalbuminemia b	HT st I	CC	Conjunctivitis	
151	Self adm	Severe SLE					
142	Self adm	Severe SLE	Nefritis SLE	Severly stunted			
68	Self adm	PJB asianotik	DS				
148	Self adm	PJB asianotik					
156	Self adm	PJB asianotik					
95	Self adm	PJB asianotik					
130	Transfer	Severe SLE	Vomitus profuse tanpa tanda dehidrasi				
133	Transfer	Hipertensif heart d	HT	GNAPS			
74	Self adm	PJB asianotik					
133	Self adm	PJB asianotik					
48	Referral	Neonatal Ascites	Gizi buruk tp marasmik				
148	Self adm	PJB asianotik					
155	Self adm	JIA					
86	Self adm	PJB asianotik					
115	Self adm	PJB sianotik	ISK	Abses cerebri	Sepsis	ephalopati hipo	

160	Referral	AKI FS failure	HT st II	ISK		
64	Self adm	PJB asianotik	buruk tp marasmik			
55	Referral	Epilepsi Uncontroll	CMV disease	GDD		
77	Self adm	DCA dengan dehid	SN kongenital	CKD st V	potiroid kongenit	buruk tp maras
61	Self adm	Cholongitis	Atresia bilier pc	Gizi buruk tp marasmik		
92	Self adm	SNDS	Pneumonia	lipoalbuminemi	buruk tp marasmik	
162	Self adm	DF	Gastritis	Abdominal Epilepsi		
156	Transfer	PJB asianotik				
150	Referral	HT emergency	SLE Nefritis	GAC	ISK	CKD st V
100	Self adm	Vomitus Profuse	Hidrosefalus			
91	Self adm	Galloway Mawat S	SNRS	CMV	Disabilitas intelektual	
59	Self adm	Kolestasis ekstrahepatal dd intrahepatal				
159	Self adm	SN	HT gr II			
87	Self adm	PJB Asianotik				
135	Self adm	PJB asianotik				
102	Referral	Resp Failure ec air	ost Tracheotom	Pneumonia		
67	Referral	PJB asianotik	Disgenesis Cere	Hidrosefalus		
138	Referral	Encephalitis	Epilepsi	GMO	i buruk tp marasmik	
59	Referral	Bronkholitis dd ser	Mild ARDS	Hipoglikemia	VAP	
61	Referral	Prolonged fever	Pneumonia	ncephalopaty Ur	CMV	Stunted
	Referral	Resp Failure ec cor	Pneumonia			
77	Referral	Ensefalitis HSV	Cerebral Palsy	Diare persisten	Laringomalacia	TB paru
162	Self adm	DCA	SLE Nefritis	SLE	ISK	HT gr II
107	Referral	Abses submandibu	tis externa diffu	ME		
55	Referral	Pneumonia				
104	Referral	Impatensi Vp shun	Hunter synd	SNHL moderate	SDH kronis	Edema difus
74	Self adm	Hepatomegali	ISK	Phimosis	i buruk tp marasmik	
52	Self adm	ISK kompleks	stra post nefros	sefalus SN kong	PJB sianotik	
117	Self adm	PJB asianotik				
95	Self adm	SN				
	Self adm	PJB asianotik				
149	Self adm	SLE	CKD st V	ral Cerebri Arthrophy		
83	Self adm	PJB asianotik				
125	Self adm	PJB asianotik				
161	Referral	SLE				
128	Transfer	Dyspepsia dd gastritis akut				
130	Self adm	Severe SLE	Efusi pleura bilateral			
168	Referral	Edema cerebri	sefalus post vp	ntensi vp shunt tanpa dehindrasi		
83	Self adm	ISK	Phimosis gr III	CP tetraparese	Epilepsi	Gizi kurang
115	Self adm	Anemia on CKD	CKD st V	Faringitis akut	SNRS	Gizi kurang
	Transfer	PJB sianotik	Caries dentist			
103	Self adm	SNRS dlm protokol	HT gr I	lipoalbuminemia		
151	Self adm	Severe SLE				
142	Self adm	Severe SLE				
126	Self adm	PJB asianotik				
	Self adm	PJB asianotik				
61	Referral	PJB asianotik	ISK kompleks	DS	ndokarditis infekt	Phimosis
13,4	Self adm	Peritonitis on CAPI	CKD st V	Dyspepsia	Dca tanpa dehid	PJB asianotik

48	Referral	Me bacterial dd vir	Hidrocephalus	FTT		
10,7	Transfer	Hidrocephalus	Pneumonia	Cerebral palsy	Epilepsi	STomatitis
72	Self adm	DCA tanpa dehidra	PJB sianotik	Pneumonia	Diaper rush	Gizi buruk
111	Self adm	Myelitis transfersa	Hipokalemia	Hidrocele		
129	Referral	Epilepsi	Edema cerebri	idosis Respiratorik		
55	Referral	Pneumonia	PJB sianotik	Gizi kurang		
137	Self adm	Efusi Pleura	TB abdomen	Gizi buruk		
137	Referral	DHF gr I				
165	Transfer	Leptospirosis				
134	Self adm	PJB asianotik	Epilepsi	Hemiparese dextra		
130	Referral	SN Relaps Frequen	lipoalbuminemia			
60	Referral	Kolestasis Intrahep	ISK	Mikrosefali	ADS Fever	
128	Self adm	Severe SLE	Epilepsi			
56	Referral	Pneumonia	ernia Umbilikali	Gizi kurang		
77	Self adm	SN Kongenital	Hipotiroid	Gangguan Perkembangan Otak		
137	Referral	Shock hipovolemik	AKI Fase failure	lkalosi metaboli	Hipoatremia	Edema pulmo
143	Self adm	PJB sianotik	isenmeyer synd			
155	Self adm	CKD on CAPD	HT gr II			
	Referral	Tonsilopharingitis	metastase akut			
99	Self adm	PJB sianotik				
87	Self adm	PJB asianotik				
131	Self adm	Vomitus Profuse d	Asma persisten	crastosa pedis bilateral		
51	Transfer	Vomitus Profuse	DCA tanpa dehi	Hirsphrung pos	PJB asianotik	
	Self adm	PJB sianotik				
96	Self adm	PJB asianotik				
	Self adm	PJB asianotik				
96	Self adm	PJB asianotik				
145	Self adm	Dyspepsia dd gastr	Gizi kurang			
154	Self adm	Apendicitis akut	ISK			
158	Self adm	PJB sianotik				
	Self adm	PJB sianotik				
82	Self adm	PJB asianotik				
162	Self adm	Severe anemia on	CKD st IV	HT st II	Hiperparatiroid sekunder	
112	Transfer	Campak	Balanitis	emorhage post c	Hemofilia B	Alergi makanan
122	Self adm	Uncontrolled epile	CMV	Atrofi cerebri	Hemiparese	
138	Referral	SE teratasi	HT st III	AKI fase failure	GIT bleeding	
116	Referral	PJB asianotik				
131	Self adm	CKD st V	Anemia on CKD	inofaringitis ak	rdiomyopati dilat	HT
117	Self adm	CMV disease	Epilepsi	CP tetraparese	GDD	
143	Transfer	Apendicitis				
61	Referral	Sepsis membaik	Atresia esofagus	Pneumonia	GDD	Gizi kurang
80	Self adm	Epilepsi	osefalus dg Vp S	inofaringitis ak	rnia scrotalis sinis	GDD
112	Referral	CKD st V	Anemia on CKD	ISK	HT st II	dehidrasi tak be
59	Self adm	CMV cerebral	Mikrocephal	sphrung post TEPT		
93	Referral	Kejang demam kon	inofaringitis ak	GDD	Post Vp shunt	
	Referral	DCA ec rotavirus ta	Hidrocephalus	Dandy Walker	stagmus kongenit	GDD
113	Self adm	PJB asianotik				
	Referral	Pneumonia				

160	Self adm	Dyspepsia ec gastr	bdominal epilepsi			
160	Referral	AKI FS failure	ISK	Gastritis	Nefritis Interstitial dd GNA	
143	Referral	SLE severe protokol bl I				
126	Self adm	PJB sianotik				
61	Self adm	PJB asianotik				
115	Self adm	PJB asianotik				
150	Transfer	Gastritis akut				
135	Referral	DM tp I	Riwayat KAD			
144	Self adm	CKD st V	SNRS	Anemia on CKD	Hipoalbuminemia	
119	Referral	SLE				
58	Self adm	CAP	Floopy infant	PJB asianotik		
130	Self adm	Gastritis erosif	Ashma persisten			
131	Referral	SE teratasi				
131	Transfer	SLE berat	Pneumonia	Gizi buruk tp m	ADS otitis	Efusi Pleura
131	Self adm	DMD				
142	Referral	Crohn Disease	Gastroduodeni	GIT Bleeding membaik		
92	Self adm	SNRS	Gallowaymowa	Gizi buruk tp m	GDD	Hipoalbuminem
65	Self adm	Kolestasis ekstrahepatal ec atresia bilier dd kolestasis intrahepatal				
60	Self adm	Congenital cystic a	Pneumonia	Gizi kurang	Anemia normo-normo	
152	Self adm	PJB asianotik				
154	Self adm	PJB asianotik				
108	Self adm	PJB sianotik				
113	Referral	Bronkiektasis ekstl	Ataxia	Gizi buruk mara	GDD	
107	Self adm	PJB asianotik				
104	Self adm	PJB asianotik				
163	Self adm	PJB asianotik	Autisme spectrum disorder			
148	Self adm	Severe SLE bl II	CKD st V	Neuropsikiatrik lupus		
142	Self adm	Severe SLE	Nefritis SLE	Severly stunted		
129	Self adm	Severe SLE bl 6	Epilepsi dlm terapi			
154	Self adm	SLE protokol bl I				
	Referral	Resp failure	Kardiomiopati dilatasi			
130	Referral	Kardiomiopati dilatasi				
	Transfer	Expanded dengue :	DF	DIC	Encephalitis	pper GIT bleedir
49	Referral	Kolestasis Intrahep	Perdarahan intı	CMV	ISK membaik	DCA
124	Self adm	SE teratasi	Impatensi Vp sł	Hidrocephalus		CP tetraparese s	Anemia mikro-l
	Referral	SE teratasi	Epilepsi tidak terkontrol			
110	Referral	SN	Kardiomiopati dilatasi			
56	Self adm	Holoproensefali	Cerebral CMV c	GDD	Labiognathopalatoskisis	
139	Self adm	CKD st V on CAPD	Impatensi CAPI	Glomerulonefritis kronik		
	Referral	SE teratasi	Encephalitis			
51	Transfer	Epilepsi	PJB sianotik			
155	Self adm	Susp CAPD periton	HT urgensi	:KD st V on CAPD		
64	Referral	CAP	PJB asianotik			
130	Self adm	PJB asianotik	Disab intelektu	Overweight		
118	Self adm	SLE berat protokol	Drug induced hepatitis			
60	Self adm	PJB asianotik				
85	Self adm	PJB asianotik				
50	Referral	PJB asianotik				

151	Transfer	Gastroduodenitis					
126	Self adm	PJB asianotik					
79	Self adm	PJB sianotik					
110	Self adm	PJB asianotik					
94	Self adm	PJB asianotik					
107	Self adm	PJB sianotik					
111	Self adm	CKD st V on CAPD	HT st II	SN	Anemia		
112	Self adm	PJB sianotik					
161	Self adm	PJB sianotik					
	Transfer	CKB	Pneumonia	Edema Cerebri	ISK	:A tanpa dehidra	
160	Transfer	Superficial dermal burn injury					
133	Self adm	PJB sianotik					
143	Self adm	Hemiparese dextra					
133	Self adm	PJB sianotik	RFA				
50	Referral	Hidrocephalus					
160	Referral	SLE	HT st II				
105	Self adm	PJB asianotik					
56	Referral	Kolestasis Intrahep	Mikrocephal	Gizi kurang	Atresia bilier		
162	Self adm	Vomitus profuse	Abdominal epile	hidrasi tak berat			
82	Transfer	AKI FS failure	idrosephalus ob	traparese spast	Sepsis	Syok Sepsis	
	Referral	Riwayat GBS	ısi pleura bilate	Disstres resp	Dyspepsia	Gizi kurang	
	Referral	DSS	ısi pleura bilateral				
	Referral	ME	Pneumonia	Epilepsi	CMV disease	GIT bleeding	
	Transfer	Post Op Adhenolitis					
175	Referral	SDH	Edema cerebri	GIT Bleeding	Multiple VE		
	Self adm	DSS	fusi pleura dextı	Git bleeding			
	Referral	ICH contusio lobus	Edeme cerebri	Multiple interc	Multiple fraktur		
165	Transfer	Fraktur Basic Cran	Edeme cerebri	SAH	ıeumonia bilateral		
75,5	Referral	Syok	DCA	SE teratasi	Intoleransi lemak		
100	Referral	Post craniotomy e\	CKB	EDH	Hipertensi		
	Referral	Post craniotomy e\	EDH				
81	Referral	Post laparotomy	Sepsis				
156	Referral	EDH frontal	ASDH	TSAH	Post craniotomy	Flebitis	
	Transfer	Tumor	ıemia mikro-hip	buruk tp maras	Hypoalbumin		
	Transfer	Total colonic aganglion post op					
	Transfer	Post LE ec ileus me	Hiperglikemi				
	Referral	CKB	yok Hipovolemi	VES Bigemini			
	Transfer	Ileus mekanik	Post LE	HT	Gizi kurang	Syok hemoraghi	
	Transfer	GBS	Milliaria rubra	Resp failure			
	Transfer	Atresia bilier tp III	Peritonitis				
	Referral	Resp distress	ıeumonia Severe				
54,5	Referral	CAP	FTT	Gizi buruk	Hyponatremia	ARDS mild	
65	Referral	Pneumonia	E bacterial dd vi	dehidrasi tak b	Severe sepsis	Gizi buruk	
55	Referral	Kolestasis ekstrahesia Bilier Post k		Sepsis	Hypoalbumin	DIC	
64	Referral	DSS overcome	KDK	Hyponatremia	fusi Pleura Dextra		
	Self adm	Pneumonia	PJB asianotik	CRS	Hidrocele	ɘmia normo-nor	
	Referral	Pneumonia	PJB asianotik	Resp failure	i buruk tp marasmik		
104	Referral	DHF gr II	Edema pulmo	fusi pleura dextra			

67	Referral	Sepsis severe	Syok Sepsis	DSS	pper GIT bleedin	usi Pleura Dextr
167	Transfer	Post LE	Syok Sepsis	usi pleura bilate	emia normo-normo	
	Referral	CP tetraparese Spa	SE			
	Referral	KAD dg Syok	sidosis metabolik			
	Referral	Post LE	Syok Sepsis	Sepsis	Atelektasis	
	Transfer	Atresia ani post PS	Post laparatomy			
	Transfer	Hirsprung	Post op	Kista ovarium	Hypoalbumin	ISK
	Referral	Resp failure	yok Hipovolemi	AKI fase failure	DCA	Pneumonia
	Referral	Atresia bilier tp II	neumonia bilate	DIC		
142	Referral	Myelitis transfersa	Syok Sepsis	ieumonia bilateral		
129	Referral	HF	AIHA	Common Cold		
	Transfer	Cholangitis	esia Bilier Post k	PJB asianotik	Stress ulcer	
	Referral	Resp failure	yok Hipovolemi	dehidrasi tak b	ISK	
	Referral	SE teratasi	E bacterial dd v	ipper GIT bleedir	SNHL	HT st II
	Referral	Trauma abdomen	Ruptur ileus gr II			
	Referral	HF	Pancytopenia			
	Referral	CAP	pper GIT bleedir	Alkalosis resp	DCA	Hyponatremia
	Referral	Ileus mekanik	Post LE	DCA	Syok hipovolemik	Hypoglikemia
115	Transfer	PDA, PH post cath	Gizi kurang	DS	GIT bleeding	
	Transfer	Pneumonia	ARDS mild	Osteoporosis	Dextrocardia	GDD
	Referral	Syok septic	Sepsis	CAP	ARDS moderate	Hypoglikemia
76	Transfer	Hydrocephalus	tetraparese spa	Sepsis membaik	ME bacterial	
99	Transfer	Cyst common bile	Post laparatomy	eumonia bilatei	Edema larynx	
	Referral	Spell, HF	PJB sianotik	Pneumonia	AKI	
55	Referral	HF	PJB sianotik	Edema Pulmo		
88	Referral	Anemia aplastik	HF	RFA	Hypoalbumin	
	Referral	HF	Anemia gravis	pper GIT bleedir	MDS	
80	Referral	AFA	Riw SVT	anpa tanda deh	CAP	
	Referral	Spell	PJB sianotik	Pneumonia	Resp failure	
	Referral	Ileus mekanik ec intususepsi post LE				
165	Referral	Encephalopaty	Pneumonia	HT emergency	Severe SLE	AKI
	Referral	PS critical post PTB	Syok kardiogenik			
	Transfer	Post laparotomy				
	Transfer	Resp failure	ARDS	Sepsis	HT portal	Hypoalbumin
105	Self adm	Ulkus palatum	Pneumonia aspirasi			
86	Referral	Post Vp Shunt	GIT bleeding	Gizi kurang	Illeus paralitis	Syok sepsis
99	Referral	CKB	Syok hemorrag	Odeme cerebri	IVH bilateral	Hypoalbumin
161	Referral	Post LE ec internal	yok hemorraghi	Anemia		
154	Referral	Reye synd	Pneumonia			
57	Transfer	Pneumonia aspiras	Resp failure	Neuroblastoma	Hidronefrosis	ISK
98	Self adm	Encephalitis viral	Sepsis	Edeme cerebri	Dca tanpa dehid	Hipernatremia
99	Referral	KDK				
54	Referral	CAP	Anemia mikro-hipo			
69	Self adm	PJB sianotik	Pneumonia			
127	Self adm	Diare persisten tar	PCP dd bronkie	B20	Gizi buruk tp ma	Candidiasis oral
5,5	Self adm	CAP	Edward synd	Epilepsi	Laringomalacia	PJB asianotik
75	Self adm	CMV disease	GDD			
80	Referral	DCA tanpa dehidrasi				

140	Referral	Myelitis transfersa	Resp disstres	ARDS mild	Atelektasis	ıeumonia bilateı
115	Self adm	GNAPS	HT gr II	AKI fase failure	ıemia mikro-normo	
53	Referral	PJB asianotik	Gizi kurang			
123	Self adm	Steven Johnson Sy	Epilepsi	ovement disorder		
132	Self adm	PJB asianotik				
162	Self adm	Polip Recti	Overweight			
93	Self adm	SN Kongenital	Gallmowat synd			
65	Referral	PJB asianotik	Gizi buruk tp marasmik			
92	Referral	PJB asianotik				
103	Self adm	PJB sianotik				
137	Self adm	PJB asianotik	Gizi kurang			
	Referral	Sepsis	E bacterial dd vi	uctus Coleducu	DIC	GIT bleeding
	Self adm	DCA dg dehidrasi	Atresia Bilier Pc	AKI fase failure	Sepsis	
	Transfer	Post op	TTA			
106	Referral	Encephalitis viral d	Riw SE	DCA tanpa dehi	GIT bleeding	Edema cerebri
	Referral	Intususepsi ileocolica post LE				
	Referral	Severe pneumonia	Severe ARDS	TB	Sepsis	Nefritis
82	Referral	SDH Regio front-te	Edema cerebri	IVH	Edema Intracerebelli	
133	Self adm	SDH Regio Paretal	Hidrosephalus (	Hemofilia		
	Transfer	Post kolangografi	Atresia Bilier	PJB asianotik	Gizi buruk tp ma	Anemia grafis
	Referral	ME viral dd bacteri	Pneumonia bilateral			
69	Transfer	SE	Epilepsi	Mikrocepal	CMV disease	
	Self adm	GBS relaps	Gizi buruk	Alkalosis metabolik		
64	Referral	Post LE	Hipoalbuminen	Peritonitis	Sepsis	
95	Transfer	Sepsis	Syok Sepsis	Spinal musculaı	Bronkopneumor	TB
	Self adm	Resp failure	Pneumonia bila	Riw GBS	Cystitits	
	Transfer	CKD st V on CAPD	Anemia on CKD	ISK	Pneumonia	Edeme pulmo
55	Referral	SDH Frontal temp	Pneumonia	Anemia ec bloo	SE	AKI
	Referral	PJB asianotik	Post ADO	LE		
52	Referral	General peritonitis ec NEC gr III B				
55	Referral	Edema pulmo	PJB asianotik	DS	Pneumonia bilat	FTT
123	Referral	SE	Me viral dd bac	Edema Cerebri	GIT bleeding	Alkalosis metab
	Transfer	Resp failure	Encephalitis vir	SE	CAP	Edema cerebri
	Transfer	ALL HR on protoco	Hiperglikemi	Sepsis	Syok Sepsis	DCA
	Referral	ME	SE overcome	Obesity		
79	Referral	DSS overcome	Efusi pleura dextra			
166	Referral	PJR	Asma bronkiale			
148	Self adm	PJR	Gizi buruk tp m	Complete RBB		
150	Self adm	Severe SLE bl II	CKD st V	Neuropsikiatri S	Anemia normo-r	DCA tanpa dehi
133	Self adm	SLE severe	Pneumonia	Gizi buruk tp marasmik		
144	Self adm	CKD st V on hemoc	SNRS			
	Self adm	Ensefalitis viral dd	Oedem cerebri	Gangguan Men	Epilepsi	
95	Self adm	Vomitus profuse ta	Hidrocephal ob	CMV kongenita	Gizi kurang	
88	Self adm	PJB asianotik				
115	Self adm	PJB asianotik				
128	Referral	Involuntary moven	DRA			
152	Self adm	PJB asianotik				
	Self adm	High output stoma	rsprung post TE	Common Cold		

117	Self adm	Severe SLE	Nefritis SLE			
139	Transfer	PJR				
132	Referral	Kolestasis	Gizi kurang			
66	Self adm	PJB asianotik				
128	Self adm	PJB asianotik				
	Self adm	PJB sianotik				
147	Self adm	PJB asianotik				
148	Self adm	SLE	Anemia	Dispepsia		
54	Self adm	PJB sianotik				
159	Self adm	SLE on protokol bl	HT st II	Nefritis SLE	nemia normo-hip	lipoalbuminemi
54	Self adm	Congenital adrenal	Gizi kurang			
77	Self adm	PJB asianotik				
94	Self adm	PJB asianotik	DS			
154	Self adm	JIA Oligointra artikuler				
160	Self adm	SE teratasi	Disabilitas intel	punctum pedis	evere Malnutritic	Epilepsi
123	Self adm	Oedem cerebri	Chepalgia			
93	Self adm	SN Kongenital	CKD st V	CP tetraparese	Gizi kurang	
110	Self adm	Anemia on CKD	CKD st V	HT st II	merulonefritis kr	lipoalbuminemi
56	Self adm	PJB asianotik	Anemia mikro-r	Gizi buruk tp marasmik		
77	Self adm	ME viral dd bacteri	Kejang demam	pilepsi dlm tera	tetraparese spas	Stomatitis
151	Self adm	SLE	Probable dengue			
78	Referral	Cerebral CMV dise	GDD	ephalus post vp shunt		
94	Referral	PCP	TB paru	Gizi buruk mara	Candidiasis oral	
102	Self adm	HIV st IV	TB paru	Pneumonia	CMV cerebral	A tanpa dehidra
102	Referral	Kejang demam kor	RFA	Purotitis	Fhimosis	
59	Self adm	Uncontrolled epile	ebral CMV dise	Mikrocepali		
61	Referral	Pneumonia	buruk tp maras	CMV disease		
63	Self adm	Pneumonia berat	Prolonged fever	A tanpa dehidr	Piere robin syn	CMV
132	Referral	DSS overcome	xpanded dengu	Gizi kurang		
161	Self adm	Abdominal pain ec	dlm protokol bl	ISK		
110	Self adm	PJB asianotik				
149	Self adm	SLE				
128	Self adm	PJB asianotik				
61	Self adm	PJB asianotik	CRS	Gizi buruk marasmik		
72	Self adm	PJB asianotik				
116	Referral	PJR				

15,97%	61	79,92%
72,50%	29	32,52%
70,33%	64	38,56%
75,29%	64	40,67%
46,27%	62	87,58%
74,16%	66	49,72%
58,03%	112	57,10%
72,92%	35	97,96%
80,00%	8	58,82%
50,00%	2	57,14%
91,89%	34	92,50%

88,89%	8	34,62%

Underlying disease6	Underlying disease7	Classified Diagnosis	Invasive devices				
			'ICU/war	CVL	Leght of CVC use (days)	ventilator	Leght of ventilator use (days)
		Respiratory	M2	N		N	
		Infectious	M2	N		N	
		Infectious	M2	N		N	
		Infectious	M2	N		N	
		Gastrohepatology	M2	N		N	
		Infectious	PICU	N		N	
		Neurology	M2	N		N	
		Surgery	M2	N		N	
		Cardiovascular	M2	N		N	
		Infectious	M2	N		N	
		Infectious	M2	N		N	
		Cardiovascular	M4	N		N	
		Cardiovascular	M4	N		N	
		Respiratory	M2	N		N	
		Infectious	M2	N		N	
		Infectious	M2	N		N	
		Respiratory	M2	N		N	
		Infectious	M2	N		N	
		Infectious	M2	N		N	
		Infectious	PICU	N		N	
		Neurology	M2	N		N	
		Neurology	M2	N		N	
		Infectious	PICU	N		N	
		Infectious	PICU	N		N	
		Infectious	M2	N		N	
		Infectious	M2	N		N	
		Infectious	PICU	N		N	
		Infectious	M2	N		N	
		Cardiovascular	M2	N		N	
		Gastrohepatology	M2	N		N	
		Neurology	M4	N		N	
		Infectious	M2	N		N	
		Infectious	M2	N		N	
		Neurology	M2	N		N	
		Infectious	M2	N		N	
		Infectious	M2	N		N	
		Neurology	M2	N		N	
		Infectious	M2	N		N	
		Cardiovascular	M2	N		N	
		Neurology	M2	N		N	
		Neurology	M2	N		N	
		Infectious	M2	N		N	
		Neurology	M2	N		N	

	Neurology	M2	N		N	
	Respiratory	M2	N		N	
	Gastrohepatology	M2	N		N	
	Neurology	M2	N		N	
	Infectious	M2	N		N	
	Respiratory	M2	N		N	
	Infectious	PICU	N		N	
	Infectious	PICU	N		N	
	Cardiovascular	M2	N		N	
	Neurology	PICU	N		Y	3
	Neurology	M2	N		N	
	Infectious	M2	N		N	
	Infectious	M2	N		N	
	Sepsis	PICU	N		Y	5
	Infectious	PICU	N		N	
	Respiratory	M2	N		N	
	Neurology	PICU	N		N	
	Infectious	PICU	N		N	
	Neurology	M2	N		N	
	Neurology	M2	N		N	
	Neurology	PICU	N		Y	2
	Malnutrition	M2	N		N	
	Neurology	M2	N		N	
	Infectious	M2	N		N	
	Neurology	M2	N		N	
	Infectious	M2	N		N	
	Neurology	M2	N		N	
	Respiratory	M2	Y	8	N	
	Respiratory	M2	N		N	
	Neurology	PICU	N		Y	13
	Infectious	M2	N		N	
	Sepsis	M2	N		N	
	Sepsis	PICU	N		Y	3
	Neurology	M2	N		N	
	Gastrohepatology	M4	N		N	
	Cardiovascular	M2	N		N	
	Respiratory	M2	N		N	
	Infectious	PICU	N		N	
	Neurology	PICU	N		N	
\ tanpa dehidrasi	Infectious	PICU	N		N	
	Nephrology	M2	N		N	
	Respiratory	M2	N		N	
	Neurology	PICU	N		Y	3
	Gastrohepatology	M2	N		N	
	Infectious	PICU	N		N	
	Neurology	M2	N		N	
	Infectious	M2	N		N	
sis hepatis dg HT portal	Gastrohepatology	M2	N		N	

	Neurology	M2	N	N	
	Neurology	M2	N	N	
	Gastrohepatology	M2	N	N	
	Endocrine	M2	N	N	
	Neurology	M2	N	N	
	Infectious	M2	N	N	
	Infectious	M2	N	N	
	Gastrohepatology	M2	N	N	
	Infectious	M2	N	N	
	Neurology	M4	N	N	
	Cardiovascular	M2	N	N	
estasis ekstrahepatal	Cardiovascular	M2	N	N	
	Cardiovascular	M2	N	N	
	Neurology	M2	N	N	
	Neurology	M2	N	N	
	Gastrohepatology	PICU	N	N	
	Infectious	PICU	N	N	
	Gastrohepatology	M2	N	N	
	Gastrohepatology	M2	N	N	
	Neurology	M2	N	N	
	Infectious	M2	N	N	
	Infectious	M2	N	N	
	Respiratory	M2	N	N	
	Infectious	M2	N	N	
	Cardiovascular	M2	N	N	
	Infectious	PICU	N	N	
	Infectious	PICU	N	N	
	Cardiovascular	M4	N	N	
	Malnutrition	M4	N	N	
	Cardiovascular	M4	N	N	
	Neurology	M4	N	N	
	Cardiovascular	M4	N	N	
	Cardiovascular	M4	N	N	
	Nephrology	M4	N	N	
	Neurology	M4	N	N	
	Endocrine	M4	N	N	
	Nephrology	M4	N	N	
	Neurology	PICU	N	Y	26
	Cardiovascular	M4	N	N	
	Cardiovascular	M4	N	N	
	Cardiovascular	M2	N	N	
	Cardiovascular	M4	N	N	
	Cardiovascular	M4	N	N	
	Hemato-oncology	M4	N	N	
	Neurology	M4	N	N	
	Neurology	M4	N	N	
	Nephrology	M4	N	N	
	Gastrohepatology	M4	N	N	

Cardiovascular	M4	N		N	
Gastrohepatology	M4	N		N	
Immunology	M4	N		N	
Immunology	M4	N		N	
Nephrology	M4	N		N	
Endocrine	M4	N		N	
Neurology	M4	N		N	
Gastrohepatology	M4	N		N	
Cardiovascular	M4	N		N	
Immunology	M4	N		N	
Neurology	M4	N		N	
Neurology	M4	N		N	
Gastrohepatology	M4	N		N	
Cardiovascular	M4	N		N	
Infectious	M4	N		N	
Cardiovascular	M4	N		N	
Cardiovascular	M4	N		N	
Immunology	M4	N		N	
Cardiovascular	M4	N		N	
Gastrohepatology	M4	Y	5	Y	3
Cardiovascular	M4	N		N	
Nephrology	M4	N		N	
Gastrohepatology	M4	N		N	
Nephrology	M4	N		N	
Gastrohepatology	M4	N		N	
Gastrohepatology	M4	N		N	
Nephrology	M4	N		N	
Nephrology	M4	N		N	
Neurology	M4	N		N	
Sepsis	PICU	Y	4	N	
Gastrohepatology	PICU	N		N	
Nephrology	M4	N		N	
Neurology	M4	N		N	
Cardiovascular	M4	N		N	
Cardiovascular	M4	N		N	
Cardiovascular	M4	N		N	
Gastrohepatology	M4	N		N	
Nephrology	M4	N		N	
Cardiovascular	M4	N		N	
Gastrohepatology	M4	N		N	
Nephrology	M4	N		N	
Nephrology	M4	N		N	
Nephrology	M4	N		N	
Neurology	M4	N		N	
Cardiovascular	M4	N		N	
Cardiovascular	M4	N		N	
Cardiovascular	M4	N		N	
Gastrohepatology	M4	N		N	

Respiratory	PICU	N	N
Respiratory	M2	N	N
Neurology	PICU	N	N
Cardiovascular	M4	N	N
Nephrology	M4	N	N
Gastrohepatology	M4	N	N
Nephrology	M4	N	N
Neurology	M2	N	N
Gastrohepatology	M2	N	N
Cardiovascular	M4	N	N
Gastrohepatology	M4	N	N
Cardiovascular	M4	N	N
Neurology	M4	N	N
Infectious	M4	N	N
Neurology	M4	N	N
Immunology	M4	N	N
Neurology	M4	N	N
Nephrology	M4	N	N
Nephrology	M4	N	N
Immunology	M4	N	N
Nephrology	M4	N	N
Gastrohepatology	M4	N	N
Immunology	M4	N	N
Cardiovascular	M4	N	N
Cardiovascular	M4	N	N
Cardiovascular	M4	N	N
Malnutrition	M4	N	N
Cardiovascular	M4	N	N
Neurology	M4	N	N
Nephrology	M4	N	N
Gastrohepatology	M4	N	N
Nephrology	M4	N	N
Gastrohepatology	M4	N	N
Cardiovascular	M4	N	N
Nephrology	M4	N	N
Cardiovascular	M4	N	N
Cardiovascular	M4	N	N
Cardiovascular	M4	N	N
Cardiovascular	M4	N	N
Cardiovascular	M4	N	N
Cardiovascular	M4	N	N
Endocrine	M4	N	N
Gastrohepatology	M4	N	N
Nephrology	M4	N	N
Cardiovascular	M4	N	N
Cardiovascular	M4	N	N
Cardiovascular	M4	N	N
Neurology	M4	N	N

		Cardiovascular	M4	N		N	
		Gastrohepatology	M4	N		N	
		Gastrohepatology	M4	N		N	
		Gastrohepatology	M4	N		N	
		Gastrohepatology	M4	N		N	
		Gastrohepatology	M4	N		N	
		Cardiovascular	M4	N		N	
		Neurology	M4	N		N	
		Neurology	M2	N		Y	4
mfoma burkit		Sepsis	M2	N		N	
		Neurology	M2	N		N	
		Infectious	M2	N		N	
		Infectious	M2	N		N	
		Infectious	M2	N		N	
		Infectious	M2	N		N	
		Neurology	M2	N		N	
		Infectious	M2	N		N	
		Respiratory	M2	N		N	
		Infectious	M2	N		N	
		Sepsis	PICU	N		Y	2
		Infectious	PICU	N		N	
		Surgery	PICU	Y	3	Y	1
		Gastrohepatology	PICU	Y	8	Y	7
nofaringitis akut		Neurology	PICU	Y	7	Y	15
		Sepsis	PICU	N		Y	4
		Infectious	PICU	Y	6	Y	6
ksi Intracranial		Sepsis	PICU	Y	6	N	
		Infectious	PICU	N		N	
		Infectious	PICU	N		N	
		Infectious	PICU	N		N	
		Infectious	PICU	N		N	
		Cardiovascular	PICU	N		Y	5
		Immunology	M4	N		Y	3
	VAP	Neurology	M2	N		Y	6
		Sepsis	PICU	N		Y	3
		Infectious	PICU	Y	3	Y	4
		Neurology	PICU	Y	11	Y	11
		Cardiovascular	PICU	N		Y	3
		Infectious	PICU	N		N	
		Gastrohepatology	M2	N		N	
		Hemato-oncology	PICU	Y	4	Y	3
		Sepsis	PICU	Y	2	Y	3
		Neurology	PICU	Y	3	Y	3
		Sepsis	PICU	Y	30	Y	15
		Surgery	M2	Y	19	Y	2
estasis intrahepatal	Alkalosis resp r	Respiratory	PICU	N		Y	6
		Respiratory	M2	N		Y	4
		Respiratory	PICU	N	17	Y	14

		Sepsis	PICU	Y	16	Y	9
		Infectious	PICU	N		Y	7
		Nephrology	M4	N		N	
		Neurology	M2	N		N	
o renal emboli		Hemato-oncology	M4	N		N	
	Schizenechepal GDD	Neurology	M2	N		N	
		Nephrology	M4	Y		N	
silitis kronis		Neurology	PICU	N		N	
		Respiratory	M2	N		N	
		Cardiovascular	M4	N		N	
		Cardiovascular	M4	N		N	
		Cardiovascular	M4	N		N	
		Cardiovascular	M2	N		N	
		Infectious	PICU	N		N	
		Cardiovascular	M4	N		N	
		Immunology	M4	N		N	
dg pnymosis		Infectious	M2	N		N	
		Cardiovascular	M4	N		N	
		Respiratory	M4	N		N	
		Gastrohepatology	M4	N		N	
		Gastrohepatology	M4	N		N	
		Infectious	M2	N		N	
		Cardiovascular	M4	N		N	
		Cardiovascular	M4	N		N	
nia umbilikalis		Cardiovascular	M2	N		N	
		Infectious	M2	N		N	
		Neurology	M2	N		N	
		Gastrohepatology	M4	N		N	
		Cardiovascular	M2	N		Y	4
		Respiratory	M2	N		N	
		Respiratory	M2	Y	10	Y	14
		Endocrine	PICU	N		Y	2
		Neurology	M2	N		N	
		Neurology	M2	N		N	
		Respiratory	M2	N		N	
		Cardiovascular	M4	N		N	
		Cardiovascular	M4	N		N	
		Neurology	M4	N		N	
		Neurology	M4	N		N	
		Cardiovascular	M4	N		N	
		Immunology	M2	N		N	
		Immunology	M4	N		N	
		Immunology	M4	N		N	
		Cardiovascular	M4	N		N	
		Infectious	M2	N		N	
		Neurology	M4	N		N	
		Infectious	M2	N		N	
wn Syndrome		Gastrohepatology	M2	N		N	

	Respiratory	M2	N		N	
	Immunology	M4	N		N	
	Gastrohepatology	M4	N		N	
	Neurology	M4	N		N	
	Infectious	M2	N		N	
	Nephrology	M4	N		N	
	Respiratory	M2	N		N	
	Gastrohepatology	M2	N		N	
	Neurology	M2	N		N	
	Gastrohepatology	M2	N		N	
	Respiratory	M2	N		N	
	Nephrology	M4	N		N	
	Cardiovascular	M4	N		N	
	Cardiovascular	M4	N		N	
	Infectious	M2	N		N	
	Hemato-oncology	M2	N		N	
	Immunology	M4	N		N	
	Nephrology	M4	N		N	
	Infectious	M4	N		N	
tanpa dehidrasi	Sepsis	PICU	Y	12	Y	12
	Cardiovascular	M4	N		N	
	Nephrology	M4	N		N	
	Respiratory	M2	N		N	
	Infectious	PICU	N		N	
	Gastrohepatology	M4	N		N	
	Infectious	PICU	N		N	
	Neurology	M2	N		N	
	Gastrohepatology	PICU	Y	20	Y	21
	Sepsis	PICU	N		Y	6
dosis metabolik berat	Infectious	PICU	Y	8	Y	8
idrasi tak berat	Neurology	PICU	N		Y	5
	Infectious	M2	N		N	
	Infectious	PICU	N		N	
	Cardiovascular	M4	N		N	
ma Intracerebri	Nephrology	M4	N		N	
	Nephrology	M4	N		N	
	Neurology	M4	N		N	
	Endocrine	M4	N		N	
	Respiratory	M2	N		N	
	Gastrohepatology	M4	N		N	
	Cardiovascular	M2	N		N	
	Infectious	PICU	N		N	
	Infectious	PICU	N		N	
	Nephrology	M4	N		N	
	Infectious	PICU	N		N	
	Cardiovascular	PICU	N		Y	3
	Infectious	M4	N		N	
	Cardiovascular	M4	N		N	

ticollis colli dextra	Neurology	PICU	N		N	
	Cardiovascular	M4	N		N	
	Gastrohepatology	M4	N		N	
	Nephrology	PICU	N		N	
	Infectious	PICU	N		N	
	Gastrohepatology	M4	N		N	
	Hemato-oncology	PICU	Y	7	N	
	Neurology	M2	N		N	
	Respiratory	M2	N		N	
	Neurology	M4	N		N	
	Nephrology	M4	N		N	
	Infectious	M2	N		N	
	Cardiovascular	M2	N		N	
	Neurology	M2	N		N	
	Cardiovascular	M2	N		N	
	Immunology	M4	N		N	
	Gastrohepatology	M2	N		N	
	Endocrine	M4	N		N	
	Cardiovascular	M4	N		N	
	Cardiovascular	M4	N		N	
	Cardiovascular	M4	N		N	
	Gastrohepatology	M4	N		N	
	Infectious	M2	N		N	
	Nephrology	M4	N		N	
	Gastrohepatology	M4	N		N	
	Nephrology	M4	N		N	
	Nephrology	M4	N		N	
	Neurology	M2	Y	20	N	
	Respiratory	PICU	N		Y	9
	Infectious	PICU	N		Y	6
	Infectious	PICU	N		N	
	Surgery	M4	N		N	
	Immunology	M4	N		N	
PJB asianotik	Infectious	PICU	Y	15	Y	14
	Gastrohepatology	M4	N		N	
	Cardiovascular	M4	N		N	
	Nephrology	M4	N		N	
	Cardiovascular	M4	N		N	
	Respiratory	M2	N		N	
	Respiratory	M2	N		N	
	Sepsis	M2	N		N	
	Cardiovascular	M4	N		N	
	Gastrohepatology	M4	N		N	
	Cardiovascular	M4	N		N	
	Cardiovascular	PICU	N		N	
	Nephrology	M4	N		N	
	Infectious	PICU	N		N	
	Cardiovascular	M4	N		N	

	Neurology	M2	N		N	
	Cardiovascular	M4	N		N	
	Cardiovascular	M4	N		N	
	Gastrohepatology	M4	N		N	
	Gastrohepatology	M2	N		N	
	Neurology	PICU	N		N	
	Cardiovascular	M2	N		N	
	Immunology	M4	N		N	
	Infectious	M2	N		N	
	Respiratory	M2	N		N	
mia post transfusi	Nephrology	PICU	N		N	
	Respiratory	M2	N		N	
	Neurology	PICU	Y	7	Y	7
	Nephrology	M4	N		N	
	Nephrology	M4	N		N	
	Immunology	M4	N		N	
	Endocrine	M4	N		N	
	Respiratory	M2	N		N	
	Infectious	M2	N		N	
	Immunology	M4	N		N	
	Cardiovascular	M4	N		N	
	Gastrohepatology	M4	N		N	
	Cardiovascular	M4	N		N	
	Cardiovascular	M4	N		N	
SNHL	Respiratory	M2	N		N	
	Infectious	M2	N		N	
	Infectious	M2	N		N	
	Infectious	M2	N		N	
	Neurology	M2	N		N	
	Neurology	M2	N		N	
	Neurology	M2	N		N	
	Nephrology	M4	N		N	
	Cardiovascular	M4	N		N	
	Gastrohepatology	M4	N		N	
	Neurology	M2	N		N	
	Cardiovascular	M4	N		N	
	Cardiovascular	M4	N		N	
	Gastrohepatology	M4	N		N	
	Surgery	PICU	Y	21	N	
	Sepsis	PICU	Y	16	Y	3
	Gastrohepatology	M4	N		Y	6
ISK	Sepsis	PICU	Y	16	Y	2
	Respiratory	PICU	N		N	
	Cardiovascular	M4	N		N	
	Neurology	M2	N		N	
	Neurology	PICU	Y	11	Y	2
	Neurology	M2	N		N	
	Cardiovascular	M4	N		N	

	Cardiovascular	M4	N		N	
	Respiratory	M4	N		N	
	Infectious	M2	N		N	
	Infectious	M2	N		N	
	Sepsis	M2	N		N	
	Immunology	M4	N		N	
	Neurology	M2	N		N	
	Cardiovascular	M4	N		N	
	Cardiovascular	M4	N		N	
	Cardiovascular	M4	N		N	
	Immunology	M4	N		N	
	Gastrohepatology	M4	N		N	
	Cardiovascular	M2	N		N	
	Cardiovascular	M4	N		N	
	Cardiovascular	M2	N		N	
	Nephrology	M4	N		N	
	Sepsis	PICU	N		Y	6
	Gastrohepatology	M4	Y	13	N	
Sepsis	Infectious	PICU	Y	9	Y	8
	Nephrology	M4	N		N	
	Infectious	PICU	N		N	
	Cardiovascular	M4	N		N	
	Infectious	M2	N		N	
	Cardiovascular	M2	N		N	
	Cardiovascular	M2	N		Y	23
	Neurology	M2	N		N	
	Neurology	M4	N		N	
	Cardiovascular	M4	N		N	
	Cardiovascular	M4	N		N	
	Nephrology	M2	N		N	
	Infectious	M2	N		N	
	Surgery	PICU	Y	5	Y	5
	Neurology	M4	N		N	
	Respiratory	M2	N		N	
	Neurology	M2	N		N	
	Neurology	M2	N		N	
	Gastrohepatology	M4	N		N	
	Gastrohepatology	M2	N		N	
	Gastrohepatology	M2	N		N	
	Infectious	M2	N		N	
	Immunology	M4	N		N	
	Neurology	M4	N		N	
	Gastrohepatology	M4	N		N	
	Cardiovascular	M4	N		N	
	Neurology	M2	N		N	
	Gastrohepatology	M2	Y	9	N	
	Cardiovascular	M4	N		N	
	Infectious	PICU	N		N	

	Infectious	M2	N		N	
	Cardiovascular	M4	N		N	
	Cardiovascular	M4	N		N	
	Surgery	PICU	Y	23	N	
	Cardiovascular	M4	N		N	
	Infectious	M2	N		N	
	Nephrology	M4	N		N	
DSS	Sepsis	M2	N		Y	4
	Respiratory	M2	N		N	
	Immunology	M4	N		N	
	Cardiovascular	M4	N		N	
	Gastrohepatology	M4	N		N	
	Respiratory	M2	N		N	
	Neurology	M2	N		N	
	Neurology	M4	N		N	
	Cardiovascular	M4	N		N	
	Cardiovascular	M4	N		N	
	Neurology	M4	N		N	
	Gastrohepatology	M4	Y	10	N	
	Respiratory	M4	N		N	
	Infectious	M2	N		N	
	Respiratory	M2	N		N	
	Neurology	M4	N		N	
	Infectious	M4	N		N	
	Immunology	M4	N		N	
	Cardiovascular	M4	N		N	
DCA	Infectious	M2	N		N	
	Infectious	M2	N		N	
Efusi pleura	Gastrohepatology	M4	N		N	
	Immunology	M2	N		N	
	Neurology	M2	N		N	
	Nephrology	M4	N		N	
	Neurology	M2	N		N	
	Cardiovascular	M4	N		N	
	Cardiovascular	M4	N		N	
Pneumonia	Nephrology	M4	N		N	
	Infectious	M2	N		N	
	Infectious	M2	N		N	
	Neurology	M2	N		N	
	Cardiovascular	M4	N		N	
	Cardiovascular	M4	N		N	
	Infectious	M2	N		N	
	Cardiovascular	M4	N		N	
	Surgery	M4	N		N	
	Cardiovascular	M4	N		N	
	Nephrology	M2	N		N	
	Gastrohepatology	M2	N		N	
	Cardiovascular	M4	N		N	

	Neurology	M4	N	N	
	Respiratory	M2	N	N	
	Neurology	M2	N	N	
	Nephrology	M4	N	N	
	Cardiovascular	M4	N	N	
	Cardiovascular	M4	N	N	
	Cardiovascular	M4	N	N	
	Sepsis	M2	N	N	
	Infectious	M2	N	N	
	Cardiovascular	M4	N	N	
	Nephrology	M4	N	N	
	Neurology	M4	N	N	
	Infectious	M2	N	N	
	Gastrohepatology	M4	N	N	
	Neurology	M2	N	N	
	Neurology	M2	N	N	
	Gastrohepatology	M4	N	N	
	Infectious	M2	N	N	
	Gastrohepatology	M2	N	N	
	Gastrohepatology	M2	N	N	
	Respiratory	M2	N	N	
	Neurology	M2	N	N	
	Immunology	M4	N	N	
	Immunology	M4	N	N	
	Cardiovascular	M4	N	N	
	Cardiovascular	M4	N	N	
	Respiratory	M4	N	N	
	Gastrohepatology	M4	N	N	
	Gastrohepatology	M4	N	N	
	Infectious	M4	N	N	
	Neurology	M2	N	N	
	Immunology	M4	N	N	
GIT bleeding	Sepsis	M2	N	N	
	Neurology	M2	N	N	
	Infectious	M2	N	N	
	Cardiovascular	M4	N	N	
	Respiratory	M2	N	N	
	Respiratory	M2	N	N	
	Infectious	M2	N	N	
	Cardiovascular	M4	N	N	
	Respiratory	M2	N	N	
	Infectious	M2	N	N	
	Cardiovascular	M4	N	N	
	Gastrohepatology	M4	N	N	
	Cardiovascular	M4	N	N	
	Cardiovascular	M4	N	N	
mo	Cardiovascular	PICU	N	Y	7
	Nephrology	M4	N	N	

	Cardiovascular	M4	N		N	
	Infectious	M2	N		N	
	Immunology	M4	N		N	
	Neurology	M2	N		N	
Flebitis	Sepsis	M2	N		N	
	Nephrology	PICU	Y	8	Y	2
	Neurology	M4	N		N	
	Infectious	M2	N		N	
	Respiratory	M2	N		N	
	Respiratory	M4	N		N	
	Cardiovascular	M4	N		N	
	Respiratory	M2	N		N	
	Infectious	M2	N		N	
	Nephrology	M4	N		N	
	Cardiovascular	M4	N		N	
	Infectious	M2	N		N	
	Malnutrition	M2	N		N	
	Gastrohepatology	M4	N		N	
	Cardiovascular	M4	N		N	
	Nephrology	M4	N		N	
	Gastrohepatology	M2	N		N	
	Gastrohepatology	M2	N		N	
	Nephrology	M4	N		N	
	Gastrohepatology	M4	N		N	
	Infectious	M2	N		N	
	Respiratory	M2	N		N	
	Cardiovascular	M4	N		N	
	Cardiovascular	M4	N		N	
	Cardiovascular	M4	N		N	
	Infectious	M2	N		N	
	Cardiovascular	M4	N		N	
	Immunology	M4	N		N	
	Infectious	M2	N		N	
	Neurology	M2	N		N	
	Cardiovascular	M4	N		N	
	Nephrology	M4	N		N	
	Infectious	M2	N		N	
	Immunology	M4	N		N	
	Neurology	M2	N		N	
	Nephrology	M4	N		N	
	Infectious	PICU	Y	4	Y	4
	Neurology	M4	N		N	
	Respiratory	M2	N		N	
	Respiratory	M2	N		N	
	Cardiovascular	M4	N		N	
	Cardiovascular	M4	N		N	
	Gastrohepatology	M4	N		N	
	Gastrohepatology	M2	N		N	

ntol lemak dan laktosa	Infectious	M2	N		N	
	Cardiovascular	M4	N		N	
	Cardiovascular	M4	N		N	
	Cardiovascular	M4	N		N	
	Gastrohepatology	M4	N		N	
	Gastrohepatology	M4	N		N	
	Gastrohepatology	M4	N		N	
	Neurology	PICU	N		N	
	Nephrology	M4	N		N	
	Gastrohepatology	PICU	N		N	
	Gastrohepatology	M4	N		N	
	Gastrohepatology	M4	N		N	
	Cardiovascular	M4	N		N	
	Cardiovascular	M4	N		N	
	Cardiovascular	M4	N		N	
	Infectious	M2	N		N	
\ tanpa dehidrasi	Neurology	M2	N		N	
	Nephrology	M4	N		N	
	Cardiovascular	M4	N		N	
	Cardiovascular	M4	N		N	
	Nephrology	M4	N		N	
	Immunology	M4	N		N	
	Respiratory	M2	N		N	
	Infectious	M2	N		N	
	Cardiovascular	M4	N		N	
	Cardiovascular	M4	N		N	
	Infectious	M2	N		N	
	Infectious	M2	N		N	
	Infectious	M2	N		N	
	Infectious	M2	N		N	
	Gastrohepatology	M2	N		N	
	Infectious	M2	N		N	
	Sepsis	M2	N		N	
:A tanpa dehidrasi	Sepsis	M2	Y	42	Y	7
dosis metabolik	Nephrology	M4	N		Y	6
	Respiratory	M2	N		Y	6
	Neurology	M2	N		N	
	Neurology	M4	N		N	
	Respiratory	M2	N		N	
	Neurology	M4	N		N	
	Infectious	M2	N		N	
	Neurology	PICU	N		N	
	Respiratory	M2	N		N	
	Respiratory	M2	N		N	
	Gastrohepatology	M2	N		N	
mia normo-normo	Cardiovascular	PICU	N		Y	13
	Gastrohepatology	M4	N		N	
	Gastrohepatology	M4	N		N	

		Gastrohepatology	M2	N		N	
		Nephrology	M4	N		N	
		Neurology	M4	N		N	
		Gastrohepatology	M4	N		N	
		Gastrohepatology	M4	N		N	
		Immunology	M4	N		N	
		Infectious	M2	N		N	
		Immunology	M4	N		N	
		Cardiovascular	M4	N		N	
		Gastrohepatology	M4	N		N	
		Infectious	M2	N		N	
		Infectious	M2	N		N	
		Gastrohepatology	M4	N		N	
		Cardiovascular	M4	N		N	
		Infectious	M2	N		N	
		Cardiovascular	M4	N		N	
		Cardiovascular	M4	N		N	
		Cardiovascular	M4	N		N	
		Nephrology	M4	N		N	
		Neurology	M4	N		N	
		Immunology	M2	N		N	
		Immunology	M4	N		N	
		Cardiovascular	M4	N		N	
		Neurology	M4	N		N	
		Neurology	M4	N		N	
		Gastrohepatology	M2	N		N	
		Neurology	M2	N		N	
		Infectious	M2	N		N	
		Infectious	M2	N		N	
		Nephrology	M4	N		N	
		Cardiovascular	M4	N		N	
		Cardiovascular	M4	N		N	
asi		Cardiovascular	M2	N		N	
S moderate		Nephrology	PICU	Y	36	Y	3
		Cardiovascular	M4	N		N	
		Neurology	M2	N		N	
		Cardiovascular	M4	N		Y	2
		Neurology	M4	N		N	
		Gastrohepatology	M2	N		N	
		Nephrology	M4	Y	17	Y	21
		Cardiovascular	PICU	Y	16	Y	19
		Gastrohepatology	M4	Y	6	Y	6
		Surgery	PICU	Y	11	Y	3
		Surgery	PICU	Y	10	Y	2
	ISK	Sepsis	PICU	Y	12	Y	9
		Respiratory	M2	N		N	
		Nephrology	M4	N		N	
		Cardiovascular	M4	N		N	

Cardiovascular	M2	N		N	
Neurology	PICU	N		Y	7
Neurology	M4	N		N	
Respiratory	M2	N		N	
Infectious	M2	N		N	
Neurology	M2	N		N	
Neurology	M4	N		N	
Cardiovascular	M4	N		N	
Immunology	M4	N		N	
Cardiovascular	M4	N		N	
Cardiovascular	M4	N		N	
Nephrology	M2	N		N	
Nephrology	M2	N		N	
Immunology	M4	N		N	
Nephrology	M4	N		N	
Gastrohepatology	M4	N		N	
Infectious	M2	N		N	
Cardiovascular	M4	N		N	
Cardiovascular	M4	N		N	
Cardiovascular	M4	N		N	
Respiratory	M2	N		N	
Neurology	M4	N		N	
Cardiovascular	M4	N		N	
Respiratory	M2	N		N	
Neurology	M2	N		N	
Neurology	PICU	N		Y	6
Infectious	PICU	N		N	
Respiratory	M2	N		N	
Infectious	M2	N		N	
Cardiovascular	M4	N		N	
Nephrology	M4	N		N	
Neurology	M2	N		N	
Infectious	PICU	Y	6	N	
Neurology	M2	N		N	
Surgery	M4	N		Y	6
Neurology	M2	N		N	
Neurology	M2	N		N	
Cardiovascular	M4	N		N	
Immunology	M4	N		N	
Gastrohepatology	M4	N		N	
Immunology	M4	N		N	
Neurology	M2	N		N	
Infectious	PICU	N		N	
Cardiovascular	M4	N		N	
Infectious	PICU	N		N	
Respiratory	M2	N		N	
Endocrine	M4	N		N	
Gastrohepatology	M4	N		N	

	Cardiovascular	M4	N		N	
	Cardiovascular	M4	N		N	
	Cardiovascular	M4	N		N	
	Neurology	M4	N		N	
	Nephrology	M4	N		N	
	Gastrohepatology	M2	N		N	
	Nephrology	M4	N		N	
	Cardiovascular	M4	N		N	
	Infectious	M2	N		N	
	Infectious	M2	N		N	
	Nephrology	M4	N		N	
	Gastrohepatology	M4	N		N	
	Respiratory	M2	N		N	
	Cardiovascular	M4	N		N	
	Respiratory	M2	N		N	
	Cardiovascular	M4	N		N	
	Cardiovascular	M4	N		N	
	Respiratory	M2	N		N	
	Endocrine	M4	N		N	
	Nephrology	M4	N		N	
	Nephrology	M4	N		N	
	Nephrology	M4	N		N	
	Infectious	PICU	N		N	
	Infectious	PICU	N		N	
	Respiratory	M2	N		N	
	Nephrology	M4	N		N	
	Neurology	M2	N		N	
ISK	Nephrology	M4	N		N	
	Nephrology	M2	N		N	
	Neurology	PICU	N		N	
	Cardiovascular	M4	N		N	
	Respiratory	M2	N		N	
	Neurology	PICU	N		N	
	Neurology	M2	N		N	
	Respiratory	M2	N		N	
	Cardiovascular	M4	N		N	
	Neurology	M4	N		N	
	Immunology	M4	N		N	
	Nephrology	M4	N		N	
	Gastrohepatology	M4	N		N	
	Respiratory	M2	N		N	
	Gastrohepatology	M4	N		N	
	Neurology	M2	N		N	
	Nephrology	M2	N		N	
	Gastrohepatology	M4	N		N	
	Gastrohepatology	M4	N		N	
	Infectious	M2	N		N	
	Gastrohepatology	PICU	Y	6	Y	3

	Nephrology	M4	N	N	
	Cardiovascular	M4	N	N	
	Endocrine	M4	N	N	
	Gastrohepatology	M4	N	N	
	Immunology	M4	N	N	
	Infectious	M2	N	N	
	Neurology	M2	N	N	
	Gastrohepatology	M2	N	N	
asi	Sepsis	PICU	N	Y	7
	Respiratory	M2	N	N	
	Cardiovascular	M4	N	N	
	Respiratory	M2	N	N	
	Gastrohepatology	M2	N	N	
	Neurology	M4	N	N	
	Cardiovascular	M4	N	N	
	Neurology	M4	N	N	
	Hemato-oncology	M4	N	N	
	Cardiovascular	M4	N	N	
	Cardiovascular	M4	N	N	
	Neurology	M4	N	N	
	Neurology	M4	N	N	
	Immunology	M4	N	N	
	Gastrohepatology	M2	N	N	
	Infectious	M2	N	N	
	Neurology	M4	N	N	
	Cardiovascular	M2	N	N	
	Gastrohepatology	M2	N	N	
	Gastrohepatology	M4	N	N	
	Respiratory	M2	N	N	
	Cardiovascular	M4	N	N	
per GIT bleeding	Hemato-oncology	PICU	N	N	
	Neurology	PICU	N	N	
	Nephrology	M4	N	N	
	Immunology	M4	N	N	
	Immunology	M4	N	N	
	Cardiovascular	M4	N	N	
	Cardiovascular	M4	N	N	
	Gastrohepatology	M2	N	N	
	Gastrohepatology	M4	N	N	
	Neurology	PICU	N	Y	8
	Cardiovascular	M4	N	N	
	Infectious	PICU	N	N	
Flebitis	Sepsis	PICU	N	Y	4
	Cardiovascular	M4	N	N	
	Neurology	M2	N	N	
	Gastrohepatology	M2	N	N	
	Sepsis	PICU	N	Y	3
	Neurology	M4	N	N	

	Respiratory	M2	N	N
	Neurology	M2	N	N
	Infectious	M2	N	N
	Cardiovascular	M4	N	N
	Immunology	M2	N	N
	Nephrology	M4	N	N
	Neurology	M4	N	N
	Infectious	M2	N	N
	Infectious	PICU	N	N
	Endocrine	M4	N	N
tumor pituitari	Endocrine	M4	N	N
	Gastrohepatology	M2	N	N
	Nephrology	M4	N	N
	Respiratory	M2	N	N
	Neurology	M4	N	N
	Nephrology	M4	N	N
	Cardiovascular	M4	N	N
	Gastrohepatology	M4	N	N
	Gastrohepatology	M4	N	N
	Neurology	M4	N	N
	Neurology	M4	N	N
	Nephrology	M4	N	N
	Cardiovascular	M4	N	N
	Cardiovascular	M4	N	N
	Cardiovascular	M4	N	N
	Cardiovascular	M4	N	N
	Neurology	M2	N	N
	Gastrohepatology	M4	N	N
	Infectious	M2	N	N
	Sepsis	M4	N	N
	Respiratory	M2	N	N
	Cardiovascular	M4	N	N
	Neurology	M4	N	N
	Neurology	M4	N	N
	Immunology	M4	N	N
	Cardiovascular	M2	N	N
	Neurology	M4	N	N
	Cardiovascular	M4	N	N
	Cardiovascular	M4	N	N
	Immunology	M4	N	N
	Respiratory	PICU	N	N
	Gastrohepatology	M2	N	N
	Neurology	M2	N	N
	Gastrohepatology	M2	N	N
	Neurology	M2	N	N
	Gastrohepatology	M2	N	N
	Respiratory	M2	N	N
	Cardiovascular	M4	N	N

	Nephrology	M4	N		N	
	Neurology	M2	N		N	
	Cardiovascular	M4	N		N	
	Respiratory	M2	N		N	
	Cardiovascular	M2	N		N	
	Respiratory	M2	N		N	
	Cardiovascular	M4	N		N	
	Respiratory	M2	N		N	
	Immunology	M4	N		N	
	Cardiovascular	M4	N		N	
	Gastrohepatology	M4	N		N	
	Cardiovascular	M4	N		N	
	Nephrology	M4	N		N	
	Cardiovascular	M4	N		N	
	Neurology	M2	N		N	
	Immunology	M4	N		N	
	Gastrohepatology	M2	N		N	
	Neurology	M2	N		N	
	Cardiovascular	M4	N		N	
	Nephrology	M4	N		N	
	Cardiovascular	M4	N		N	
	Neurology	M4	N		N	
	Gastrohepatology	M4	N		N	
	Immunology	M4	N		N	
	Cardiovascular	M4	N		N	
	Endocrine	M4	N		N	
	Gastrohepatology	PICU	Y	11	Y	4
	Neurology	M2	N		N	
	Infectious	PICU	N		N	
	Cardiovascular	M4	N		N	
	Respiratory	M2	N		N	
	Cardiovascular	M4	N		N	
	Cardiovascular	M4	N		N	
	Hemato-oncology	M2	N		N	
Pneumonia	Neurology	PICU	N		Y	10
	Respiratory	M2	N		N	
	Gastrohepatology	M4	N		N	
	Respiratory	M2	N		N	
	Neurology	M2	N		N	
	Neurology	M2	N		N	
	Respiratory	M2	N		N	
	Respiratory	M2	N		N	
	Nephrology	M4	N		N	
	Infectious	M2	N		N	
	Respiratory	M2	N		N	
	Gastrohepatology	M2	N		N	
	Respiratory	M4	N		N	
	Neurology	M2	N		N	

	Gastrohepatology	M2	N		N	
	Respiratory	M2	N		N	
	Nephrology	M4	N		N	
	Cardiovascular	M4	N		N	
	Respiratory	M2	N		N	
	Neurology	M4	N		N	
	Cardiovascular	M4	N		N	
	Infectious	M2	N		N	
	Infectious	M2	N		N	
	Endocrine	M4	N		N	
	Cardiovascular	M2	N		N	
	Nephrology	M4	N		N	
	Cardiovascular	M4	N		N	
	Infectious	M2	N		N	
	Immunology	M4	N		N	
	Nephrology	M4	N		N	
	Respiratory	M2	N		N	
	Cardiovascular	M4	N		N	
	Cardiovascular	M4	N		N	
	Endocrine	M4	N		N	
	Neurology	M4	N		N	
	Neurology	M4	N		N	
	Immunology	M4	N		N	
	Immunology	M4	N		N	
	Nephrology	M4	N		N	
	Neurology	M4	N		N	
	Cardiovascular	M4	N		N	
	Respiratory	M4	N		N	
	Cardiovascular	M4	N		N	
VAP	Gastrohepatology	M2	N		N	
	Respiratory	M2	N		N	
	Cardiovascular	M4	N		N	
	Respiratory	M2	N		N	
	Neurology	M2	N		N	
mik	Neurology	PICU	Y	7	N	
	Neurology	M2	N		N	
	Neurology	M2	N		N	
	Respiratory	M2	N		N	
	Respiratory	M2	N		N	
	Gastrohepatology	M2	N		N	
	Infectious	PICU	N		N	
	Cardiovascular	M4	N		N	
	Respiratory	M4	N		N	
	Respiratory	PICU	Y	20	Y	43
	Neurology	PICU	N		Y	4
	Neurology	PICU	N		N	
oalbuminemia	Immunology	M4	N		N	
	Cardiovascular	M4	N		N	

	Infectious	M2	N	N
	Neurology	M2	N	N
	Cardiovascular	M4	N	N
	Cardiovascular	M4	N	N
	Cardiovascular	M4	N	N
	Gastrohepatology	M2	N	N
	Gastrohepatology	M4	N	N
	Nephrology	M4	N	N
	Nephrology	M4	N	N
	Gastrohepatology	M4	N	N
	Gastrohepatology	M4	N	N
	Immunology	M4	N	N
	Gastrohepatology	M4	N	N
	Infectious	M2	N	N
	Cardiovascular	M4	N	N
	Immunology	M4	N	N
	Cardiovascular	M4	N	N
	Cardiovascular	M2	N	N
	Neurology	M4	N	N
	Nephrology	M4	N	N
DS	Gastrohepatology	M4	N	N
	Cardiovascular	M4	N	N
	Gastrohepatology	M4	N	N
	Neurology	M2	N	N
	Neurology	M2	N	N
	Neurology	M2	N	N
	Neurology	M2	N	N
	Respiratory	M2	N	N
	Nephrology	M2	N	N
	Neurology	M4	N	N
	Infectious	M2	N	N
	Respiratory	M2	N	N
	Cardiovascular	M2	N	N
	Cardiovascular	M4	N	N
	Malnutrition	M4	N	N
	Nephrology	M4	N	N
	Neurology	M4	N	N
	Neurology	M4	N	N
	Nephrology	M4	N	N
	Gastrohepatology	M4	N	N
	Cardiovascular	M4	N	N
	Immunology	M4	N	N
	Immunology	M4	N	N
	Gastrohepatology	M4	N	N
	Immunology	M4	N	N
	Neurology	M4	N	N
	Hemato-oncology	M4	N	N
	Respiratory	M4	N	N

	Immunology	M4	N		N	
	Endocrine	M4	N		N	
	Cardiovascular	M4	N		N	
	Cardiovascular	M4	N		N	
	Cardiovascular	M4	N		N	
	Cardiovascular	M4	N		N	
	Immunology	M4	N		N	
	Neurology	M4	N		N	
	Cardiovascular	M4	N		N	
	Neurology	M4	N		N	
	Cardiovascular	M4	N		N	
	Nephrology	M4	N		N	
	Cardiovascular	M4	N		N	
	Nephrology	M4	N		N	
	Immunology	M4	N		N	
	Cardiovascular	M4	N		N	
	Neurology	M4	N		N	
	Immunology	M4	N		N	
	Nephrology	M4	N		N	
	Gastrohepatology	M4	N		N	
	Cardiovascular	M4	N		N	
	Sepsis	PICU	Y	13	Y	7
asi	Sepsis	PICU	N		Y	9
	Neurology	PICU	N		N	
	Neurology	PICU	N		N	
	Respiratory	PICU	N		Y	5
	Infectious	PICU	N		N	
	Cardiovascular	M2	N		N	
	Immunology	M2	N		N	
	Respiratory	M2	N		N	
	Neurology	M2	N		N	
	Gastrohepatology	M2	N		N	
	Infectious	M2	N		N	
	Respiratory	M4	N		N	
	Sepsis	M2	N		Y	3
	Nephrology	M4	N		N	
	Endocrine	M4	N		N	
	Gastrohepatology	M4	N		N	
	Gastrohepatology	M4	N		N	
	Gastrohepatology	M4	N		N	
	Infectious	PICU	N		N	
	Infectious	PICU	N		N	
	Sepsis	PICU	Y	9	Y	1
hepalopaty dengue	Infectious	PICU	y	5	y	5
	Neurology	PICU	N		Y	4
	Neurology	PICU	N		Y	4
	Infectious	PICU	N		N	
	Infectious	PICU	N		N	

	Infectious	PICU	N		N	
Acites	Nephrology	M4	Y	7	Y	7
	Sepsis	PICU	Y	6	Y	19
	Sepsis	PICU	N		Y	3
MODS	Sepsis	PICU	Y	4	Y	5
	Infectious	PICU	N		N	
	Infectious	PICU	N		Y	3
	Infectious	PICU	N		N	
	Infectious	PICU	N		N	
	Infectious	PICU	N		N	
	Infectious	PICU	N		N	
Hipoglikemia	Cardiovascular	M4	N		N	
	Gastrohepatology	M4	N		N	
HAP	Nephrology	M4	Y	21	N	
	Neurology	PICU	Y	23	N	
	Cardiovascular	M4	N		N	
	Gastrohepatology	M4	N		N	
	Neurology	M4	N		N	
	Cardiovascular	M4	N		N	
	Gastrohepatology	M4	N		N	
	Cardiovascular	M4	N		N	
	Cardiovascular	M4	N		N	
	Immunology	M4	N		N	
	Immunology	M4	N		N	
	Gastrohepatology	M4	N		N	
	Neurology	M4	N		N	
	Neurology	M4	N		N	
	Cardiovascular	M2	N		N	
	Immunology	M2	N		N	
	Neurology	M2	N		N	
	Gastrohepatology	M2	N		N	
	Respiratory	M2	N		N	
	Infectious	M2	N		N	
	Neurology	M2	N		N	
	Sepsis	M2	N		N	
	Respiratory	M2	N		N	
	Respiratory	M2	N		N	
	Gastrohepatology	M4	N		N	
	Cardiovascular	M4	N		N	
Diaper rush	Neurology	M4	N		N	
Post EVD	Neurology	M4	N		N	
	Hemato-oncology	M4	N		N	
	Cardiovascular	M4	N		N	
	Cardiovascular	M4	N		N	
	Cardiovascular	M4	N		N	
	Nephrology	PICU	N		N	
	Cardiovascular	M4	N		N	
	Immunology	M4	N		N	

	Cardiovascular	M4	N		N	
	Cardiovascular	M4	N		N	
	Cardiovascular	M4	N		N	
	Gastrohepatology	M4	N		N	
	Cardiovascular	M4	N		N	
Oral trush	Nephrology	M4	N		N	
	Neurology	M4	N		N	
	Respiratory	M4	N		N	
	Immunology	M4	N		N	
	Respiratory	M4	N		N	
	Cardiovascular	M4	N		N	
	Gastrohepatology	M4	N		N	
	Nephrology	M4	N		N	
	Neurology	M4	N		N	
	Immunology	M4	N		N	
buruk tp marasmik	Neurology	M4	N		N	
	Cardiovascular	M4	N		N	
	Cardiovascular	M4	N		N	
	Cardiovascular	M4	N		N	
	Nephrology	M4	N		N	
mik	Nephrology	M4	N		N	
HAP	Sepsis	PICU	Y	19	N	
	Cardiovascular	M2	N		N	
ding	Neurology	PICU	Y	14	Y	5
	Endocrine	M2	N		N	
	Neurology	M2	Y		Y	
	Neurology	PICU	N		N	
	Neurology	M2	N		N	
ISK	Nephrology	M4	N		N	
	Neurology	M4	N		N	
GIT bleeding	Neurology	PICU	N		N	
	Cardiovascular	M4	N		N	
	Respiratory	M2	N		N	
	Cardiovascular	M2	N		N	
	Cardiovascular	M4	N		N	
buruk tp marasmik	Gastrohepatology	M4	N		N	
	Nephrology	PICU	N		N	
buruk tp marasmik	Gastrohepatology	M4	N		N	
	Respiratory	M4	N		N	
oalbuminemia	Gastrohepatology	M4	N		N	
	Cardiovascular	M4	N		N	
	Cardiovascular	M4	N		N	
	Immunology	M4	N		N	
	Immunology	M4	N		N	
Gizi buruk	Gastrohepatology	M2	N		N	
Syndrome sotos	Neurology	M2	N		N	
	Sepsis	M2	N		N	
	Neurology	M4	N		N	

	Cardiovascular	M4	N	N
	Cardiovascular	M4	N	N
	Cardiovascular	M4	N	N
	Respiratory	M2	N	N
	Nephrology	M4	N	N
	Immunology	M4	N	N
	Gastrohepatology	M4	N	N
	Nephrology	PICU	N	N
	Respiratory	M4	N	N
	Gastrohepatology	M4	N	N
	Cardiovascular	M4	N	N
	Cardiovascular	M4	N	N
	Cardiovascular	M4	N	N
	Gastrohepatology	M4	N	N
Mikrosefali	Cardiovascular	M4	N	N
	Neurology	M4	N	N
	Infectious	M2	N	N
Infektif endocarditis	Nephrology	M4	N	N
	Nephrology	M4	N	N
	Cardiovascular	M4	N	N
	Neurology	M2	N	N
	Neurology	M2	N	N
	Cardiovascular	M4	N	N
	Respiratory	M2	N	N
	Respiratory	M4	N	N
	Nephrology	M2	N	N
	Respiratory	M4	N	N
	Cardiovascular	M4	N	N
	Cardiovascular	M4	N	N
	Cardiovascular	M4	N	N
	Gastrohepatology	M4	N	N
	Gastrohepatology	M4	N	N
	Immunology	M4	N	N
	Neurology	M4	N	N
	Neurology	M4	N	N
	Cardiovascular	M4	N	N
	Cardiovascular	M4	N	N
	Cardiovascular	M4	N	N
	Cardiovascular	M4	N	N
	Neurology	M4	N	N
	Gastrohepatology	M4	N	N
	Neurology	M4	N	N
	Gastrohepatology	M4	N	N
	Gastrohepatology	M4	N	N
	Gastrohepatology	M4	N	N
	Neurology	M4	N	N
	Immunology	M4	N	N
	Immunology	M4	N	N

	Gastrohepatology	M4	N	N
GDD	Neurology	M2	N	N
	Neurology	M2	N	N
	Cardiovascular	M4	N	N
	Cardiovascular	M4	N	N
Mikrosefali	Infectious	M2	N	N
	Cardiovascular	M4	N	N
	Neurology	M2	N	N
	Respiratory	M4	N	N
	Cardiovascular	M4	N	N
	Gastrohepatology	M4	N	N
	Immunology	M4	N	N
	Neurology	M2	N	N
	Neurology	M2	N	N
	Respiratory	M2	N	N
	Cardiovascular	M4	N	N
	Cardiovascular	M4	N	N
	Nephrology	M4	N	N
	Gastrohepatology	M4	N	N
	Cardiovascular	M4	N	N
	Gastrohepatology	M4	N	N
	Nephrology	M4	N	N
	Nephrology	M4	N	N
	Gastrohepatology	M4	N	N
	Nephrology	PICU	N	N
	Gastrohepatology	M4	N	N
	Immunology	M4	N	N
	Cardiovascular	M4	N	N
	Endocrine	M4	N	N
	Immunology	M4	N	N
	Cardiovascular	M4	N	N
	Cardiovascular	M4	N	N
	Nephrology	M4	N	N
	Nephrology	M4	N	N
	Cardiovascular	M4	N	N
	Immunology	M4	N	N
ISPA	Immunology	M4	N	N
	Neurology	M4	N	N
ARH	Neurology	M4	N	N
	Gastrohepatology	M4	N	N
	Gastrohepatology	M4	N	N
	Cardiovascular	M4	N	N
	Neurology	M4	N	N
FTT	Gastrohepatology	M4	N	N
	Neurology	M4	N	N
	Gastrohepatology	M4	N	N
	Neurology	M4	N	N
	Gastrohepatology	M2	N	N

	Gastrohepatology	M2	N	N
	Neurology	M2	N	N
	Neurology	M2	N	N
	Gastrohepatology	M2	N	N
	Gastrohepatology	M2	N	N
	Cardiovascular	M4	N	N
	Cardiovascular	M4	N	N
	Infectious	M2	N	N
	Cardiovascular	M2	N	N
	Cardiovascular	M4	N	N
	Cardiovascular	M4	N	N
	Neurology	M4	N	N
	Neurology	M4	N	N
	Cardiovascular	M4	N	N
	Infectious	M2	N	N
	Gastrohepatology	M4	N	N
	Endocrine	M4	N	N
	Cardiovascular	M4	N	N
	Cardiovascular	M4	N	N
	Cardiovascular	M4	N	N
GDD	Neurology	M4	N	N
	Cardiovascular	M4	N	N
	Neurology	M4	N	N
	Cardiovascular	M4	N	N
	Immunology	M4	N	N
	Cardiovascular	M4	N	N
	Neurology	M4	N	N
	Cardiovascular	M4	N	N
DCA tanpa dehidrasi	Neurology	M4	N	N
	Gastrohepatology	M4	N	N
	Cardiovascular	M4	N	N
	Gastrohepatology	M4	N	N
	Cardiovascular	M4	N	N
	Immunology	M4	N	N
	Cardiovascular	M4	N	N
	Cardiovascular	M4	N	N
Pioderma	Nephrology	M4	N	N
	Cardiovascular	M4	N	N
	Immunology	M4	N	N
	Cardiovascular	M4	N	N
	Nephrology	M4	N	N
palbuminemia	Nephrology	M4	N	N
	Immunology	M4	N	N
	Cardiovascular	M4	N	N
	Nephrology	M4	N	N
	Gastrohepatology	M2	N	N
	Cardiovascular	M4	N	N
	Cardiovascular	M4	N	N

	Gastrohepatology	M4	N		N	
	Neurology	M4	N		N	
	Neurology	PICU	Y	7	N	
Upper GIT bleeding	Infectious	M2	N		N	
	Neurology	M4	N		N	
Miliaria Christalina	Neurology	PICU	N		Y	2
	Gastrohepatology	PICU	N		N	
	Neurology	M2	N		N	
	Neurology	M2	N		N	
	Respiratory	M2	N		N	
Anemia	Neurology	M4	N		N	
	Neurology	M2	N		N	
	Cardiovascular	M4	N		N	
	Neurology	M2	N		N	
	Neurology	M2	N		N	
	Cardiovascular	M4	N		N	
	Cardiovascular	M4	N		N	
	Cardiovascular	M4	N		N	
	Cardiovascular	M4	N		N	
	Cardiovascular	M4	N		N	
	Gastrohepatology	M4	N		N	
	Infectious	M2	N		N	
	Nephrology	M4	N		N	
	Gastrohepatology	M4	N		N	
	Cardiovascular	M4	N		N	
	Cardiovascular	M4	N		N	
	Cardiovascular	M4	N		N	
	Cardiovascular	M4	N		N	
	Cardiovascular	M4	N		N	
	Nephrology	M4	N		N	
	Nephrology	M4	N		N	
	Cardiovascular	M4	N		N	
	Cardiovascular	M4	N		N	
	Neurology	M2	N		N	
	Nephrology	M2	N		N	
	Cardiovascular	M4	N		N	
	Cardiovascular	M4	N		N	
	Cardiovascular	M4	N		N	
	Cardiovascular	M4	N		N	
	Cardiovascular	M4	N		N	
	Nephrology	M4	N		N	
	Neurology	M4	N		N	
Pneumonia	Cardiovascular	PICU	N		N	
	Immunology	M4	N		N	
Trombositopenia	Gastrohepatology	M4	N		N	
	Gastrohepatology	M2	N		N	
	Respiratory	PICU	N		Y	6
	Cardiovascular	M4	N		N	

	Gastrohepatology	PICU	N	Y	2
	Nephrology	M4	N	N	
	Neurology	M4	N	N	
	Cardiovascular	M4	N	N	
	Cardiovascular	M4	N	N	
	Immunology	M4	N	N	
	Cardiovascular	M4	N	N	
	Nephrology	M4	N	N	
	Gastrohepatology	M4	N	N	
	Infectious	M4	N	N	
	Cardiovascular	M4	N	N	
	Immunology	M4	N	N	
	Cardiovascular	M4	N	N	
AKI	Cardiovascular	M2	N	N	
Meatal Stenosis	Nephrology	M4	N	N	
ol pulmo dextra	Respiratory	M2	N	N	
	Respiratory	M2	N	N	
	Infectious	M2	N	N	
	Neurology	PICU	N	N	
	Sepsis	M4	N	N	
	Immunology	M4	N	N	
	Infectious	M2	N	N	
Kista arachnoid dd abses	Sepsis	M2	N	N	
	Cardiovascular	M4	N	N	
	Cardiovascular	M4	N	N	
Ascites	Gastrohepatology	M2	N	N	
GDD	Neurology	M2	N	N	
buruk tp marasmik	Gastrohepatology	M4	N	N	
buruk tp marasmik	Cardiovascular	M2	N	N	
	Neurology	M2	N	N	
	Neurology	M2	N	N	
mik	Neurology	M2	N	N	
	Gastrohepatology	M4	N	N	
	Gastrohepatology	M4	N	N	
buruk tp marasmik	Neurology	M2	N	N	
	Neurology	M4	N	N	
	Infectious	M2	N	N	
	Gastrohepatology	M4	N	N	
	Infectious	M2	N	N	
	Cardiovascular	M4	N	N	
	Cardiovascular	M4	N	N	
	Respiratory	M2	N	N	
	Immunology	M4	N	N	
	Neurology	M4	N	N	
	Neurology	M4	N	N	
	Cardiovascular	M4	N	N	
	Gastrohepatology	M4	N	Y	2
	Neurology	PICU	N	Y	4

	Respiratory	PICU	Y	13	Y	6
	Immunology	M4	N		N	
	Nephrology	M4	N		N	
	Cardiovascular	M4	N		N	
	Nephrology	M4	N		N	
	Cardiovascular	M4	N		N	
	Gastrohepatology	M4	N		N	
	Cardiovascular	M4	N		N	
	Nephrology	M4	N		N	
	Cardiovascular	M4	N		N	
	Infectious	M2	N		N	
	Cardiovascular	M4	N		N	
ut	Neurology	M2	N		N	
Hiperglikemia	Respiratory	PICU	N		N	
:A tanpa dehidrasi	Sepsis	PICU	N		N	
	Immunology	M4	N		N	
	Neurology	M2	N		N	
	Gastrohepatology	M2	N		N	
	Gastrohepatology	M2	N		N	
Efusi Pleura	Nephrology	M4	N		N	
	Nephrology	M4	N		N	
mik	Cardiovascular	M4	N		N	
	Neurology	M4	N		N	
	Neurology	M4	N		N	
	Cardiovascular	M4	N		N	
asi	Surgery	PICU	N		N	
	Cardiovascular	M4	N		N	
	Cardiovascular	M4	N		N	
	Cardiovascular	M4	N		N	
	Cardiovascular	M4	N		N	
	Cardiovascular	M4	N		N	
	Gastrohepatology	M2	N		N	
	Nephrology	M4	N		N	
	Immunology	M4	N		N	
	Immunology	M4	N		N	
	Cardiovascular	M4	N		N	
	Cardiovascular	M4	N		N	
	Cardiovascular	M4	N		N	
	Cardiovascular	M4	N		N	
	Immunology	M4	N		N	
	Nephrology	M4	N		N	
	Cardiovascular	M4	N		N	
	Cardiovascular	M4	N		N	
	Malnutrition	M4	N		N	
	Cardiovascular	M4	N		N	
	Immunology	M4	N		N	
	Cardiovascular	M4	N		N	
ksik	Cardiovascular	M4	N		N	

	Nephrology	M4	Y	25	N	
	Cardiovascular	M4	N		N	
	Neurology	M2	N		N	
lipoalbuminemia	Nephrology	M2	N		N	
	Gastrohepatology	M2	N		N	
	Nephrology	M4	N		N	
	Infectious	M2	N		N	
	Cardiovascular	M4	N		N	
	Immunology	M4	N		N	
	Neurology	M4	N		N	
	Nephrology	M4	N		N	
	Gastrohepatology	M4	N		N	
	Nephrology	M4	N		N	
	Cardiovascular	M4	N		N	
	Cardiovascular	M4	N		N	
	Respiratory	M4	N		N	
	Neurology	M4	N		N	
	Neurology	PICU	N		N	
	Respiratory	PICU	N		Y	20
	Neurology	M2	N		N	
	Respiratory	PICU	N		Y	9
Gizi buruk	Neurology	M2	N		N	
HAP	Immunology	M4	N		N	
	Neurology	M2	N		N	
	Respiratory	M2	N		N	
Gizi buruk	Neurology	M4	N		N	
	Malnutrition	M4	N		N	
	Nephrology	M4	N		N	
	Cardiovascular	M4	N		N	
	Nephrology	M4	N		N	
	Cardiovascular	M4	N		N	
	Immunology	M4	N		N	
	Cardiovascular	M4	N		N	
	Cardiovascular	M4	N		N	
	Immunology	M4	N		N	
	Gastrohepatology	M4	N		N	
	Immunology	M4	N		N	
	Neurology	M4	N		N	
GDD	Neurology	M4	N		N	
	Nephrology	M4	N		N	
	Cardiovascular	M4	N		N	
	Nephrology	M4	N		N	
	Immunology	M4	N		N	
	Immunology	M4	N		N	
	Cardiovascular	M4	N		N	
	Cardiovascular	M4	N		N	
HAP	Cardiovascular	M4	N		N	
Gizi buruk tp marasmik	Nephrology	M4	N		N	

	Neurology	M2	N		N	
Gizi buruk tp marasmik	Neurology	M2	N		N	
	Cardiovascular	M2	N		N	
	Neurology	M4	N		N	
	Neurology	PICU	N		Y	5
	Cardiovascular	M2	N		N	
	Respiratory	M2	N		N	
	Infectious	M2	N		N	
	Infectious	M4	N		N	
	Cardiovascular	M4	N		N	
	Nephrology	M4	N		N	
	Gastrohepatology	M4	N		N	
	Immunology	M4	N		N	
	Respiratory	M2	N		N	
	Nephrology	M4	N		N	
Efusi Pleura	Nephrology	PICU	N		N	
	Cardiovascular	M4	N		N	
	Nephrology	M4	N		N	
	Respiratory	M2	N		N	
	Cardiovascular	M4	N		N	
	Cardiovascular	M4	N		N	
	Respiratory	M4	N		N	
	Gastrohepatology	M2	N		N	
	Cardiovascular	M4	N		N	
	Cardiovascular	M4	N		N	
	Cardiovascular	M4	N		N	
	Cardiovascular	M4	N		N	
	Gastrohepatology	M4	N		N	
	Gastrohepatology	M4	N		N	
	Cardiovascular	M4	N		N	
	Cardiovascular	M4	N		N	
	Cardiovascular	M4	N		N	
	Nephrology	M4	N		N	
Sepsis	Infectious	M2	N		N	
	Neurology	M2	N		N	
	Nephrology	PICU	Y	30	Y	4
	Cardiovascular	M4	N		N	
Pneumonia	Nephrology	M2	N		N	
	Neurology	M2	N		N	
	Gastrohepatology	M4	N		N	
	Sepsis	M2	N		N	
	Neurology	M4	N		N	
erat	Nephrology	M4	N		N	
	Neurology	M2	N		N	
	Neurology	M2	N		N	
	Neurology	M2	N		N	
	Cardiovascular	M4	N		N	
	Respiratory	M2	N		N	

	Gastrohepatology	M4	N		N	
	Nephrology	M4	Y	15	N	
	Immunology	M4	N		N	
	Cardiovascular	M4	N		N	
	Cardiovascular	M4	N		N	
	Cardiovascular	M4	N		N	
	Gastrohepatology	M4	N		N	
	Endocrine	M4	N		N	
	Nephrology	M4	N		N	
	Immunology	M4	N		N	
	Respiratory	M2	N		N	
	Gastrohepatology	M4	N		N	
	Neurology	PICU	N		N	
	Immunology	M2	N		N	
	Nephrology	M4	N		N	
	Gastrohepatology	M4	N		N	
oalbuminemia	Nephrology	M4	N		N	
	Gastrohepatology	M4	N		N	
	Respiratory	M2	N		N	
	Cardiovascular	M4	N		N	
	Cardiovascular	M4	N		N	
	Cardiovascular	M4	N		N	
	Respiratory	M2	N		N	
	Cardiovascular	M4	N		N	
	Cardiovascular	M4	N		N	
	Cardiovascular	M4	N		N	
	Immunology	M4	N		N	
	Immunology	M4	N		N	
	Immunology	M4	N		N	
	Immunology	M4	N		N	
	Cardiovascular	PICU	N		Y	8
	Cardiovascular	M4	N		N	
SLE berat	Infectious	PICU	N		N	
Pneumonia	Gastrohepatology	M2	N		N	
mia mikro-hipo	Neurology	M2	N		N	
	Neurology	PICU	N		N	
	Nephrology	M4	N		N	
	Neurology	M2	N		N	
	Nephrology	M4	N		N	
	Neurology	PICU	N		N	
	Cardiovascular	M2	N		N	
	Nephrology	M4	N		N	
	Cardiovascular	M2	N		N	
	Cardiovascular	M4	N		N	
	Immunology	M4	N		N	
	Cardiovascular	M4	N		N	
	Cardiovascular	M4	N		N	
	Cardiovascular	M4	N		N	

			Gastrohepatology	M4	N		N	
			Cardiovascular	M4	N		N	
			Cardiovascular	M4	N		N	
			Cardiovascular	M4	N		N	
			Cardiovascular	M4	N		N	
			Cardiovascular	M4	N		N	
			Nephrology	M4	N		N	
			Cardiovascular	M4	N		N	
			Cardiovascular	M4	N		N	
asi			Neurology	PICU	Y	11	Y	26
			Gastrohepatology	M4	N		N	
			Cardiovascular	M4	N		N	
			Neurology	M4	N		N	
			Cardiovascular	M4	N		N	
			Neurology	M4	N		N	
			Immunology	M4	N		N	
			Cardiovascular	M4	N		N	
			Gastrohepatology	M4	N		N	
			Gastrohepatology	M4	N		N	
	DIC	ARDS mild	Neurology	PICU	Y	6	Y	7
			Neurology	PICU	N		Y	3
			Infectious	PICU	N		N	
tetraparese spasidosis metabol			Neurology	PICU	N		Y	16
			Surgery	PICU	N		N	
			Surgery	PICU	N		Y	4
			Infectious	PICU	N		N	
			Surgery	PICU	N		Y	6
			Surgery	PICU	N		N	
			Gastrohepatology	PICU	N		Y	8
			Surgery	PICU	N		Y	5
			Surgery	PICU	N		Y	8
			Surgery	PICU	N		N	
			Surgery	PICU	Y	11	Y	15
			Hemato-oncology	PICU	Y	4	N	
			Surgery	PICU	N		Y	2
			Surgery	PICU	N		N	
			Neurology	PICU	Y	11	Y	17
	ISK		Surgery	PICU	N		Y	2
			Neurology	PICU	Y	10	Y	1
			Surgery	PICU	Y	10	Y	13
			Respiratory	PICU	N		N	
	ISK		Respiratory	PICU	N		Y	13
	VAP		Respiratory	M2	Y	8	Y	8
			Surgery	M4	Y	36	Y	23
			Infectious	PICU	N		N	
AKI fase failure		Edema pulmo	Cardiovascular	PICU	N		Y	23
			Cardiovascular	PICU	N		Y	6
			Infectious	PICU	N		N	

ra		Sepsis	PICU	N		N	
		Surgery	PICU	Y	4	N	
		Neurology	PICU	N		N	
		Endocrine	PICU	N		N	
		Surgery	PICU	Y	4	Y	1
		Surgery	PICU	N		N	
		Surgery	PICU	N		N	
Hidrocephalus	Encephalopaty	Respiratory	PICU	N		Y	12
		Surgery	PICU	Y	20	Y	20
		Neurology	PICU	Y	26	Y	32
		Hemato-oncology	PICU	N		N	
		Gastrohepatology	PICU	N		N	
		Respiratory	PICU	N		Y	2
		Neurology	PICU	N		N	
		Surgery	PICU	N		N	
		Hemato-oncology	PICU	N		N	
		Respiratory	PICU	N		Y	4
		Surgery	PICU	Y	6	Y	7
		Cardiovascular	PICU	N		Y	3
		Cardiovascular	PICU	N		Y	6
alitis viral dd bacterial		Sepsis	PICU	N		Y	7
		Neurology	PICU	N		N	
		Surgery	PICU	Y	11	N	
		Cardiovascular	PICU	N		N	
		Cardiovascular	PICU	N		Y	11
		Hemato-oncology	PICU	N		N	
		Hemato-oncology	PICU	N		N	
		Cardiovascular	PICU	N		N	
		Cardiovascular	PICU	N		Y	3
		Surgery	PICU	N		N	
Sepsis	Edema pulmo	Immunology	PICU	N		Y	3
		Cardiovascular	PICU	Y	6	Y	6
		Surgery	PICU	N		N	
		Gastrohepatology	PICU	N		Y	10
		Respiratory	PICU	N		Y	19
		Sepsis	PICU	Y	11	Y	13
VAP		Neurology	PICU	N		N	
		Surgery	PICU	N		Y	9
		Endocrine	PICU	Y	3	N	
Anemia		Hemato-oncology	PICU	N		N	
Hipokalemia	Hipoalbuminen	Neurology	PICU	N		N	
		Neurology	M2	N		N	
		Respiratory	M2	N		N	
		Cardiovascular	M2	N		N	
didiasis oral		Infectious	M2	N		N	
CMV		Respiratory	M2	N		N	
		Neurology	M2	N		N	
		Gastrohepatology	M2	N		N	

HT gr I		Neurology	PICU	N		Y	13
		Nephrology	M4	N		N	
		Cardiovascular	M4	N		N	
		Endocrine	M4	N		N	
		Cardiovascular	M4	N		N	
		Gastrohepatology	M4	N		N	
		Nephrology	M4	N		N	
		Cardiovascular	M4	N		N	
		Cardiovascular	M4	N		N	
		Cardiovascular	M4	N		N	
		Cardiovascular	M4	N		N	
DCA		Sepsis	PICU	N		N	
		Surgery	PICU	N		N	
		Surgery	PICU	N		N	
Candidiasis oral		Neurology	PICU	N		Y	4
		Surgery	PICU	Y	4	N	
		Respiratory	PICU	N		Y	5
		Neurology	PICU	N		Y	3
		Neurology	PICU	N		N	
VAP		Gastrohepatology	PICU	Y	14	Y	14
		Neurology	PICU	N		Y	2
		Neurology	PICU	N		N	
		Neurology	PICU	N		N	
		Surgery	PICU	Y	17	Y	10
Gizi buruk tp marasmik		Sepsis	PICU	N		Y	101
		Respiratory	PICU	N		Y	10
Sepsis	DCA tanpa deh	Nephrology	PICU	Y	20	Y	10
		Neurology	PICU	N		Y	6
		Cardiovascular	PICU	N		Y	8
		Gastrohepatology	PICU	Y	20	N	
DCA tanpa dehidrasi		Cardiovascular	PICU	N		Y	20
DCA tanpa dehidrasi		Neurology	PICU	Y	35	Y	41
DCA tanpa dehidrasi		Neurology	PICU	Y	6	Y	7
Syok hipovolem	AKI	Hemato-oncology	PICU	N		N	
		Neurology	PICU	N		N	
		Infectious	PICU	N		N	
		Cardiovascular	M4	N		N	
		Cardiovascular	M4	N		N	
A tanpa dehidrasi		Immunology	M4	N		N	
		Immunology	M4	N		N	
		Nephrology	M4	N		N	
		Neurology	PICU	N		N	
		Neurology	M4	N		N	
		Cardiovascular	M4	N		N	
		Cardiovascular	M4	N		N	
		Neurology	M4	N		N	
		Cardiovascular	M4	N		N	
		Gastrohepatology	M4	N		N	

		Immunology		M4	N		N	
		Cardiovascular		M4	N		N	
		Gastrohepatology		M4	N		N	
		Cardiovascular		M4	N		N	
		Cardiovascular		M4	N		N	
		Cardiovascular		M4	N		N	
		Cardiovascular		M4	N		N	
		Immunology		M4	N		N	
		Cardiovascular		M4	N		N	
a		Immunology		M4	N		N	
		Endocrine		M4	N		N	
		Cardiovascular		M4	N		N	
		Cardiovascular		M4	N		N	
		Immunology		M4	N		N	
		Neurology		M4	N		N	
		Neurology		M4	N		N	
		Nephrology		M4	N		N	
a		Nephrology		M4	N		N	
		Cardiovascular		M4	N		N	
Pneumonia		Neurology		M2	N		N	
		Immunology		M2	N		N	
		Neurology		M2	N		N	
		Respiratory		M2	N		N	
asi		Infectious		M2	N		N	
		Neurology		M2	N		N	
		Neurology		M2	N		N	
		Respiratory		M2	N		N	
Laringomalacia	GDD	Respiratory		M2	N		N	
		Infectious		M2	N		N	
		Gastrohepatology		M4	N		N	
		Cardiovascular		M4	N		N	
		Immunology		M4	N		N	
		Cardiovascular		M4	N		N	
		Cardiovascular		M4	N		N	
		Cardiovascular		M4	N		N	
		Cardiovascular		M4	N		N	
382	Cardio		478	286	90	1121	159	1359
40	Immun		123	538				
91	Gastro		236	1029				96
85	Infect		209	1853				120
134	Respi		153					216
89	Nepro		179					
193	Neuro		338					10,301692
48	Sepsis		49					
10	Hemato		17					
4	Malnutrisi		7					
37	Surgery		40					

9	Endocrine	26					
1122		1855					
	PICU	286	219	76,6%	172	78,5%	162
	m2	538	365	67,8%	192	52,6%	173
	m4	1031	538	52,2%	181	33,6%	175
		1855	1122				

Jrine cathete	Leght of urin catheter use (days)	Outcome	Cause of death	INOS 1 Nosocomial	When inos	Organism 1	specimen 1	Day of cultu
N		survive		N				
N		survive		N				
N		survive		N				
N		survive		URTI	6			
N		survive		N				
Y	4	survive		N				
N		survive		N				
N		survive		N				
N		survive		N				
N		survive		N				
N		survive		N				
N		survive		N				
N		survive		N				
N		survive		N				
N		survive		N				
N		survive		N				
N		survive		N				
N		survive		N				
N		survive		N				
Y	1	survive		N				
N		survive		URTI	14			
N		survive		N				
y	3	survive		N				
Y	3	survive		N				
N		survive		N				
N		survive		N				
Y	2	survive		N				
Y	4	survive		N				
N		survive		N				
N		survive		N				
N		survive		N				
N		survive		N				
N		survive		N				
N		survive		N				
N		survive		N				
N		survive		N				
N		survive		N				
N		survive		N				
N		survive		N				
N		survive		N				
N		survive		N				
Y	4	survive		N				
Y	11	survive		N				

N		survive		N				
N		survive		N				
N		survive		N				
N		survive		N				
N		survive		GIT	5			
N		survive		N				
Y	4	survive		N				
Y	3	survive		N				
N		survive		N				
Y	3	survive		HAP	7			
N		survive		URTI	3			
N		died	Respiratory	N				
N		survive		N				
Y	6	survive		N				
Y	4	survive		N				
N		survive		N				
Y	4	survive		UTI	6	Kocuria rhi	Urine	8
Y	1	survive		N				
N		survive		Likely	27			
N		survive		N				
Y	17	survive		CAUTI	19	Klebsiella p	Urine	19
N		survive		N				
N		survive		N				
N		survive		N				
N		survive		N				
N		survive		N				
N		survive		GIT	26			
N		survive		GIT	5			
N		survive		HAP	10			
Y	18	survive		HAP	15			
N		survive		UTI	4			
N		survive		N				
Y	11	survive		HAP	4			
N		survive		URTI	16			
N		survive		N				
N		died	Syok Kardic	N				
N		survive		N				
Y	3	survive		N				
Y	3	survive		N				
Y	4	survive		N				
N		survive		N				
N		survive		GIT	12			
N	7	survive		N				
N		survive		URTI	10			
N		survive		N				
N		survive		UTI	4			
N		survive		N				
N		died	Syok	UTI	5			

N		survive		N				
N		survive		N				
N		survive		N				
N		survive		HAP	4			
N		survive		N				
N		survive		N				
N		survive		N				
N		survive		N				
N		survive		N				
N		survive		N				
N		survive		N				
N		died	Pneumonia	GIT	11			
N		survive		N				
N		survive		N				
N		survive		N				
Y	4	survive		N				
Y	4	survive		N				
N		survive		N				
N		survive		N				
N		survive		GIT	6			
N		survive		N				
N		survive		N				
N		survive		N				
N		survive		N				
N		survive		GIT	4			
Y	4	survive		N				
N		survive		N				
N		survive		N				
N		survive		N				
N		survive		URTI	16			
N		survive		N				
N		survive		N				
N		survive		N				
N		survive		N				
N		survive		N				
N		survive		N				
Y	10	survive		CAUTI	6	E. Cloacae	Urine	6
Y	25	survive		CAUTI	4	Negatif	Urine	4
N		survive		N				
N		survive		N				
N		survive		N				
N		survive		N				
N		survive		N				
N		survive		N				
N		survive		N				
Y	2	survive		N				
N		survive		N				
N		survive		N				

N		survive		N				
N		survive		N				
N		survive		N				
N		survive		N				
N		survive		N				
N		survive		N				
N		survive		N				
N		survive		N				
N		survive		N				
N		survive		N				
Y	3	survive		N				
N		survive		N				
N		survive		N				
N		survive		N				
N		survive		N				
N		survive		N				
N		survive		N				
N		survive		N				
N		survive		N				
Y	3	survive		N				
N		survive		N				
N		survive		N				
N		survive		N				
N		survive		N				
N		survive		N				
N		survive		N				
N		survive		N				
N		survive		N				
N		survive		N				
Y	9	survive		SSI	13	Negatif	PUS	13
N		survive		N				
N		died	Syok	GIT	7			
N		survive		N				
N		survive		N				
N		survive		GIT	3			
N		survive		Flebitis	5			
N		survive		N				
Y	8	survive		N				
N		survive		URTI	16			
N		survive		Flebitis	5			
N		survive		N				
N		survive		N				
N		survive		N				
N		survive		N				
N		survive		N				
N		survive		N				
N		survive		N				
N		survive		N				

N		survive		N				
N		survive		N				
Y	2	survive		N				
N		survive		N				
N		survive		N				
Y	10	survive		UTI	7	Negatif	Urine	11
N		survive		N				
N		survive		N				
N		survive		N				
N		survive		GIT	13			
N		survive		N				
N		survive		N				
N		survive		N				
Y	4	survive		N				
N		survive		URTI	12			
N		survive		N				
Y	13	survive		CAUTI	16	Enterococc	Urine	17
Y	5	survive		GIT	21			
N		survive		N				
N		survive		N				
Y	5	survive		N				
N	5	survive		N				
N		survive		N				
N		survive		N				
N		survive		N				
N		survive		N				
N		survive		Likely	17			
N		survive		N				
N		survive		N				
N		survive		URTI	4			
N		survive		N				
N		survive		N				
N		survive		N				
N		survive		N				
N		survive		N				
N		survive		N				
N		survive		N				
N		survive		N				
N		survive		N				
Y	6	died	Syok	CAUTI	4	Negatif	Urine	5
N		survive		N				
N		survive		N				
N		survive		N				
N		survive		N				
N		survive		N				
N		survive		N				
N		survive		N				
N		survive		N				

N		survive		N				
N		survive		URTI	4			
N		survive		N				
N		survive		N				
N		survive		N				
N		survive		N				
N		survive		N				
N		died	Syok	N				
Y	4	survive		N				
N		died	Syok	N				
N		survive		UTI	3	Enterococc	Urine	4
N		survive		N				
Y	5	survive		N				
N		survive		N				
N		survive		N				
N		survive		N				
N		survive		N				
N		survive		N				
N		survive		N				
Y	2	died	ARDS	N				
y	2	survive		N				
Y	3	died	ARDS	N				
Y	2	survive		N				
Y	9	died	Severe Sep	HAP	5	Stenotropo	ETT	5
N		died	Severe Sep	N				
Y	6	died	Severe Sep	HAP	5			
Y	6	died	Severe ARD	N				
N		survive		N				
Y	6	survive		N				
N		survive		N				
Y	2	survive		N				
Y	7	died	Cardiomyo	N				
Y	5	died	Oedem pul	Likely	5			
Y	6	died	Syok Sepsis	VAP	26			
Y	6	survive		N				
Y	4	survive		N				
Y	11	died	Syok	VAP	7			
Y	4	died	Syok Kardic	N				
N		survive		N				
N		died	Severe Sep	Likely	15			
Y	4	died	Syok Septic	N				
Y	3	died	Syok Septic	N				
Y	3	died	Respiratory	N				
Y	13	survive		GIT	14			
Y	19	died	MODS	Likely	23	Negatif	Blood	23
Y	6	died	Severe seps	N				
Y	8	survive		GIT	22			
Y	13	survive		CAUTI	4	Enterococc	Urine	4

Y	12	survive		N				
Y	9	survive		GIT	7			
N		died	Syok Sepsis	N				
N		survive		N				
Y	11	survive		N				
N		survive		URTI	11			
Y	3	survive		N				
Y	11	survive		N				
N		survive		N				
N		survive		N				
N		survive		N				
N		survive		N				
N		survive		N				
Y	4	survive		N				
N		survive		N				
N		survive		N				
N		survive		N				
N		survive		N				
N		survive		N				
N		survive		N				
N		survive		N				
N		survive		N				
N		survive		N				
N		survive		N				
N		survive		GIT	19			
N		survive		N				
N		survive		N				
N		survive		N				
Y	8	died	Respiratory	GIT	14			
N		survive		GIT	4			
Y	13	died	Syok Septic	Likely	12	Negatif	Blood	12
Y	7	survive		N				
N		survive		N				
N		survive		HAP	8			
N		survive		N				
N		survive		N				
N		survive		N				
N		survive		N				
N		survive		N				
N		survive		N				
N		survive		N				
N		survive		N				
N		survive		N				
N		survive		N				
N		survive		N				
N		survive		N				
N		survive		N				
N		survive		N				

N		survive		N				
N		survive		N				
N		survive		N				
N		survive		N				
N		survive		N				
N		survive		N				
N		survive		N				
N		survive		N				
N		survive		N				
N		died	Respiratory	Y	Flebitis	4		
N		died	Pneumonia	N				
N		survive		N				
N		survive		N				
N		survive		N				
N		survive		N				
N		survive		N				
N		survive		N				
N		survive		N				
N		survive		N				
N		died	ARDS	GIT	5			
N		survive		N				
N		survive		N				
N		survive		N				
Y	2	survive		N				
N		survive		N				
Y	2	survive		N				
N		survive		N				
Y	8	died	DIC	UTI	8	Serratia for	Urine	10
Y	5	survive		N				
Y	7	died	Reye Snync	VAP	4			
Y	5	died	Respiratory	N				
N		survive		N				
Y	4	survive		N				
N		survive		N				
Y	14	survive		N				
N		survive		N				
N		survive		N				
N		survive		N				
Y	5	survive		URTI	44			
N		survive		GIT	3			
N		died	Syok Kardic	N				
N		survive		N				
N		survive		N				
N		survive		N				
N		survive		N				
Y	3	died	Syok Kardic	N				
N		survive		N				
N		survive		N				

Y	10	survive		SSI	12	Staphyloco	PUS	12
Y	1	survive		HAP	3			
N		survive		URTI	7			
y	11	survive		Flebitis	8			
y	4	survive		N				
N		survive		N				
Y	8	died	Severe seps	N				
N		survive		GIT	3			
N		survive		N				
N		survive		N				
N		survive		N				
N		survive		Flebitis	13			
N		survive		N				
N		survive		N				
N		survive		N				
N		survive		N				
N		survive		N				
Y	3	survive		N				
N		survive		N				
N		survive		N				
N		survive		N				
N		survive		N				
N		survive		HAP	4			
N		survive		N				
N		survive		N				
N		survive		N				
N		survive		N				
N		died	Respiratory	HAP	15			
Y	11	survive		N				
y	13	survive		CAUTI	7	Negatif	Urine	9
Y	2	survive		N				
N		survive		N				
N		survive		N				
Y	14	died	Respiratory	N				
N		survive		N				
N		survive		N				
N		survive		N				
N		survive		N				
N		survive		N				
N		survive		N				
N		survive		N				
N		survive		N				
N		survive		N				
N		survive		N				
Y	8	survive		N				
N		survive		N				
N		survive		N				
N		survive		N				

N		survive		N				
N		survive		N				
N		survive		N				
N		survive		N				
N		survive		N				
N		survive		N				
N		survive		N				
N		survive		N				
N		survive		N				
N		survive		N				
Y	13	survive		HAP	3			
N		survive		N				
Y	7	died	Respiratory	N				
Y	3	survive		URTI	23			
N		survive		N				
N		survive		N				
N		survive		N				
N		survive		N				
N		survive		N				
N		survive		N				
N		survive		N				
N		survive		N				
N		survive		N				
N		survive		N				
N		survive		GIT	7			
N		survive		N				
N		survive		N				
N		survive		N				
N		survive		N				
N		survive		N				
Y	16	survive		N				
N		survive		Likely	3			
N		survive		N				
N		survive		N				
N		survive		N				
N		survive		N				
N		survive		N				
N		survive		N				
Y	11	survive		BSI	22	A baumanii	CVC	24
Y	6	survive		CAUTI	6			
Y	6	survive		VAP	12	P aerugino:	ETT	12
Y	2	survive		UTI	4	C albicans	Urine	4
Y	4	survive		N				
N		survive		N				
N		survive		N				
Y	11	survive		BSI	12	E Cloacae	LCS	12
N		survive		N				
N		survive		N				

N		survive		N				
N		survive		N				
N		survive		N				
N		survive		N				
N		survive		BSI	21	S Hominis	Blood	21
N		survive		UTI	9	E Faecium	Urine	13
Y	16	survive		N				
N		died	Syok Kardic	N				
N		survive		HAP	4			
N		survive		N				
N		survive		N				
N		survive		N				
N		survive		URTI	3			
N		survive		N				
N		survive		N				
Y	10	survive		HAP	4			
Y	5	survive		N				
N		survive		N				
Y	7	survive		N				
N		survive		N				
Y	3	survive		N				
N		survive		N				
N		survive		N				
N		survive		N				
Y	9	survive		HAP	5			
N		survive		N				
N		survive		N				
N		survive		N				
N		survive		N				
N		survive		N				
N		survive		N				
Y	5	died	Surgery	N				
N		survive		N				
N		survive		Likely	7			
N		survive		N				
N		survive		N				
N		survive		UTI	5	Enterococu	Urine	2
N		died	Respiratory	N				
N		survive		N				
N		survive		N				
N		survive		N				
N		survive		N				
N		survive		HAP	8			
Y	11	survive		N				
N		survive		N				
Y	2	survive		CAUTI	4			
N		survive		N				
Y	2	survive		N				

N		survive		N				
N		survive		N				
N		survive		N				
Y	4	survive		BSI	15	Candida Tr	Blood	16
N		died	Aspirasi	N				
N		survive		N				
N		survive		N				
Y	4	survive		N				
N		survive		N				
N		survive		N				
N		survive		N				
N		survive		N				
N		survive		UTI	16	Candida Tr	Urine	16
N		survive		URTI	18			
N		survive		GIT	14			
N		survive		N				
N		survive		N				
N		survive		UTI	3			
Y	4	survive		N				
N		survive		HAP	19			
N		survive		Flebitis	11			
N		survive		N				
N		survive		Likely	24			
N		survive		HAP	7			
N		survive		N				
N		survive		N				
N		survive		N				
N		survive		N				
N		survive		UTI	5			
N		survive		N				
N		survive		N				
N		survive		N				
N		survive		N				
N		survive		N				
N		survive		N				
N		survive		N				
N		survive		N				
N		survive		N				
N		survive		N				
N		survive		N				
N		survive		N				
N		survive		N				
N		survive		N				
N		survive		N				
N		survive		N				
N		survive		N				
N		survive		N				
N		survive		N				

N		survive		N				
N		survive		N				
N		survive		URTI	21			
N		survive		N				
N		survive		N				
N		survive		N				
N		survive		N				
N		survive		N				
N		survive		HAP	9	S Viridans	Sputum	9
N		survive		HAP	10			
N		survive		N				
N		survive		N				
N		survive		N				
N		survive		N				
N		survive		Flebitis	7			
N		survive		N				
N		survive		N				
N		survive		N				
N		survive		N				
N		survive		N				
N		survive		N				
N		survive		N				
N		survive		N				
N		survive		N				
N		survive		N				
N		survive		N				
N		survive		N				
N		survive		N				
N		survive		N				
N		survive		N				
N		survive		N				
N		survive		N				
N		survive		N				
N		survive		N				
N		survive		N				
N		survive		N				
N		survive		N				
N		survive		N				
N		survive		N				
N		survive		N				
N		survive		N				
N		survive		N				
N		survive		UTI	12			
N		survive		N				
N		survive		N				
Y	18	died	Respiratory	HAP	18			
N		survive		N				

N		died	Respiratory	HAP	3			
N		survive		N				
N		survive		N				
N		survive		N				
N		survive		Flebitis	11			
Y	7	survive		N				
N		survive		N				
N		survive		URTI	3			
N		survive		N				
N		survive		N				
N		died	Respiratory	Flebitis	6			
N		survive		N				
Y	6	survive		N				
N		survive		N				
N		survive		N				
N		survive		N				
N		survive		N				
N		survive		N				
N		survive		N				
N		survive		N				
N		survive		N				
N		survive		N				
N		survive		UTI	10	Negatif	Urine	10
N		survive		N				
Y	5	survive		N				
N		survive		N				
N		survive		N				
N		survive		N				
Y	6	survive		HAP	12			
N		survive		N				
N		survive		HAP	7			
N		survive		N				
N		survive		N				
N		survive		N				
N		survive		N				
N		survive		N				
N		survive		HAP	4			
N		survive		N				
N		survive		N				
N		survive		N				
Y	5	survive		URTI	8			
Y	5	survive		N				
N		survive		N				
N		survive		N				
N		survive		N				
N		survive		N				
N		survive		N				
N		survive		N				

N		survive	URTI	4
N		survive	N	
N		survive	Likely	3
N		survive	N	
N		survive	N	
N		survive	GIT	9
N		survive	N	
Y	6	survive	N	
N		survive	N	
Y	6	survive	N	
N		survive	N	
N		survive	N	
N		survive	N	
N		survive	N	
N		survive	N	
N		survive	N	
N		survive	N	
N		survive	N	
N		survive	N	
N		survive	N	
N		survive	N	
N		survive	N	
N		survive	N	
N		survive	N	
N		survive	N	
N		survive	N	
N		survive	N	
N		survive	N	
N		survive	N	
N		survive	N	
N		survive	N	
N		survive	N	
N		survive	N	
Y	9	survive	N	
Y	24	survive	Likely	10
Y	6	survive	N	
N		survive	N	
N		survive	N	
N		survive	N	
N		survive	N	
N		survive	GIT	15
N		survive	N	
N		survive	N	
N		survive	N	
N		survive	N	
Y	14	survive	N	
N		survive	N	
N		survive	N	

N		survive		UTI	4	E.coli, E.Ga	Urine	5
N		survive		N				
N		survive		GIT	14			
N		survive		N				
N		survive		N				
N		survive		GIT	7			
N		Died	Syok sepsis	N				
N		survive		N				
N		survive		N				
Y	3	survive		N				
N		survive		N				
N		survive		N				
N		survive		N				
N		survive		N				
N		survive		N				
N		survive		N				
N		survive		N				
N		survive		N				
N		survive		N				
N		survive		N				
N		survive		N				
N		survive		N				
N		survive		N				
N		survive		N				
N		survive		N				
N		survive		N				
N		survive		N				
N		survive		N				
N		survive		N				
N		survive		N				
N		survive		N				
N		survive		N				
N		Died	Respiratory	HAP	23	Staphyloco	Sputum	28
Y	4	Died	Edema pulr	N				
N		survive		N				
N		survive		GIT	9			
Y	2	survive		N				
N		survive		N				
N		Died	Cardio puln	GIT	7	Klebsiella p	Feces	11
Y	30	Died	Respiratory	CAUTI	5			
Y	14	Died	MODS	Flebitis	17			
Y	6	Died	MODS	UTI	15			
Y	11	Died	Sepsis	VAP	9			
Y	5	Died	MODS	N				
Y	3	survive		Flebitis	3			
N		survive		N				
N		survive		N				
N		survive		URTI	4			

N		survive		N				
Y	7	Died	Sepsis	N				
N		survive		GIT	5			
N		survive		N				
N		survive		N				
N		survive		N				
N		survive		N				
N		survive		N				
N		survive		N				
N		survive		N				
N		survive		N				
N		died	Respiratory	HAP	36			
N		survive		UTI	5	Klebsiella p	Urine	5
N		survive		N				
N		survive		N				
N		survive		N				
N		survive		N				
N		survive		N				
N		survive		URTI	3			
N		survive		N				
N		survive		N				
N		survive		N				
N		survive		N				
N		survive		N				
N		survive		N				
Y	8	survive		N				
Y	5	survive		N				
N		survive		N				
N		survive		N				
N		survive		N				
N		survive		N				
N		survive		N				
Y	3	survive		N				
Y	7	survive		GIT	11	Klebsiella p	Feces	14
Y	12	survive		CAUTI	7			
N		survive		GIT	5			
N		survive		N				
N		survive		URTI	14			
Y	7	survive		CAUTI	4	E.coli	Urine	4
N		survive		N				
N		survive		N				
N		survive		N				
Y	2	survive		N				
N		survive		N				
N		survive		N				
N		survive		UTI	8	Negatif	Urine	19
N		died	Sepsis	Flebitis	6			
N		survive		N				

N		survive		N	
N		survive		N	
N		survive		N	
Y	12	survive		N	
Y	8	survive		N	
N		died	Respiratory	URTI	6
N		survive		N	
N		survive		N	
N		survive		N	
N		survive		N	
N		survive		N	
N		survive		N	
N		survive		N	
N		survive		N	
N		survive		N	
N		survive		N	
N		survive		N	
N		survive		N	
N		survive		URTI	12
N		survive		N	
N		survive		N	
N		survive		N	
N		survive		N	
N		survive		N	
N		survive		N	
N		died	ARDS	N	
N		survive		N	
Y	9	survive		N	
N		survive		N	
Y	18	survive		GIT	30
N		died	Sepsis	GIT	26
N		survive		N	
N		survive		N	
N		survive		N	
N		survive		N	
N		survive		N	
N		survive		N	
N		survive		N	
N		survive		N	
N		survive		N	
N		survive		N	
N		survive		UTI	14
N		survive		N	
N		survive		N	
N		survive		N	
N		survive		N	
N		survive		N	
Y	4	survive		N	

N		survive	N				
N		survive	N				
Y	7	survive	N				
N		survive	N				
N		survive	N				
N		survive	GIT	4	Klebsiella p	Feces	5
N		survive	N				
N		survive	N				
Y	7	survive	CAUTI	7	Enterococu	Urine	7
N		survive	N				
N		survive	N				
N		survive	N				
N		survive	N				
N		survive	N				
N		survive	N				
Y	7	survive	UTI	4	E.Cloacae	Urine	5
Y	4	survive	N				
N		survive	URTI	13			
N		survive	N				
N		survive	N				
N		survive	N				
N		survive	N				
N		survive	GIT	6			
N		survive	N				
N		survive	N				
N		survive	GIT	19			
Y	5	survive	N				
N		survive	N				
N		survive	N				
N		survive	N				
Y	2	survive	N				
N		survive	N				
N		survive	N				
Y	3	survive	N				
N		survive	N				
N		survive	N				
N		survive	N				
N		survive	N				
N		survive	N				
Y	8	survive	N				
N		survive	N				
Y	3	survive	N				
Y	3	survive	Flebitis	5			
N		survive	N				
N		survive	N				
N		survive	N				
Y	4	survive	N				
N		survive	HAP	6			

N		survive		N				
N		survive		N				
N		survive		N				
N		survive		N				
N		survive		N				
N		survive		N				
N		survive		N				
N		survive		N				
Y	2	survive		N				
Y	3	survive		UTI	5			
N		survive		N				
N		survive		N				
N		survive		N				
N		survive		N				
N		survive		UTI	3	Negatif	Urine	3
N		survive		N				
N		died	Syok kardic	N				
N		survive		N				
N		survive		N				
N		survive		N				
N		survive		N				
N		survive		N				
N		survive		N				
N		survive		N				
N		survive		N				
N		survive		N				
N		survive		N				
N		survive		N				
N		survive		N				
N		survive		N				
N		survive		N				
Y	7	survive		N				
N		survive		N				
N		survive		GIT	4			
N		survive		N				
N		survive		N				
N		survive		N				
N		survive		N				
N		survive		N				
N		survive		N				
N		survive		URTI	17			
N		survive		N				
N		survive		GIT	14			
N		survive		UTI	7	Negatif	Urine	9
N		survive		N				
N		survive		GIT	5			
N		survive		N				
N		survive		N				

N		survive	N				
N		survive	N				
N		survive	N				
N		survive	N				
N		survive	N				
N		survive	N				
N		survive	N				
N		survive	N				
N		survive	N				
N		survive	N				
N		survive	N				
N		survive	N				
N		survive	N				
N		survive	N				
N		survive	N				
N		survive	Likely	6			
N		survive	N				
N		survive	N				
N		survive	N				
N		survive	N				
N		survive	N				
N		survive	N				
N		survive	N				
N		survive	N				
N		survive	URTI	7			
Y	5	survive	N				
Y	4	survive	N				
N		survive	GIT	3			
Y	2	survive	N				
N		survive	GIT	7			
N		survive	N				
N		survive	HAP	6			
N		survive	N				
N		survive	N				
Y	12	survive	N				
N		survive	UTI	11	Providencia	Urine	12
N		survive	Likely	33			
N		survive	URTI	8			
N		survive	URTI	5			
N		survive	N				
N		survive	N				
N		survive	N				
N		survive	N				
N		survive	N				
N		survive	N				
N		survive	HAP	4			
N		survive	N				
N		survive	N				

N		survive		N				
N		survive		N				
N		survive		N				
N		survive		N				
N		survive		N				
N		survive		N				
N		survive		N				
N		survive		N				
N		survive		GIT	4			
N		survive		N				
N		survive		N				
N		survive		N				
N		survive		N				
N		survive		N				
N		survive		N				
N		survive		N				
N		survive		N				
N		survive		N				
N		survive		N				
N		survive		N				
N		survive		N				
N		survive		N				
N		survive		N				
N		survive		N				
N		survive		N				
N		survive		N				
N		survive		N				
N		survive		N				
N		survive		N				
N		survive		Likely	18	Negatif	Blood	18
N		survive		Likely	7			
N		survive		N				
N		died	Sepsis	GIT	14			
N		survive		HAP	3			
Y	6	died	Sepsis	BSI	8	Klebsiella p	CVC	10
N		survive		N				
N		survive		N				
N		survive		N				
N		survive		N				
N		survive		N				
N		survive		N				
N		survive		N				
N		survive		N				
Y	31	survive		Likely	7			
Y	4	survive		N				
N		survive		N				
N		survive		N				
N		survive		URTI	9			

N		survive	N				
N		survive	N				
N		survive	N				
N		survive	N				
N		survive	N				
N		survive	N				
N		survive	N				
N		survive	N				
N		survive	UTI	4	Negatif	Urine	5
N		survive	N				
N		survive	N				
N		survive	N				
N		survive	N				
N		survive	N				
N		survive	N				
N		survive	N				
N		survive	HAP	12			
N		survive	GIT	13			
N		survive	N				
N		survive	N				
N		survive	N				
N		survive	N				
N		survive	N				
N		survive	UTI	18			
N		survive	N				
N		survive	N				
N		survive	N				
N		survive	N				
N		survive	N				
N		survive	N				
N		survive	N				
N		survive	N				
N		survive	N				
N		survive	URTI	5			
N		survive	GIT	6			
N		survive	N				
N		survive	N				
N		survive	N				
N		survive	N				
N		survive	N				
N		survive	N				
N		survive	N				
N		survive	N				
N		survive	N				
N		survive	N				
Y	19	survive	CAUTI	11	E Coli	Urine	11
N		survive	N				
N		survive	N				

N		survive		UTI	3	E.Coli	Urine	3
N		survive		N				
N		survive		N				
N		survive		N				
N		survive		N				
N		died	Shock kardi	N				
N		survive		GIT	15	Klebsiella p	Feces	15
N		survive		N				
N		survive		N				
N		survive		N				
N		survive		N				
N		survive		N				
N		survive		N				
N		survive		UTI	5			
N		survive		GIT	7			
N		survive		N				
N		survive		N				
N		survive		N				
N		survive		N				
N		survive		N				
N		survive		N				
Y	9	survive		VAP	5	Acinetobac	ETT	8
Y	9	survive		N				
Y	2	survive		N				
N		survive		N				
Y	6	survive		N				
N		survive		N				
N		died	Shock kardi	UTI	14	Klebsiella p	Urine	15
N		survive		N				
N		survive		N				
N		survive		N				
N		survive		N				
N		survive		N				
N		survive		URTI	5			
N		survive		N				
N		survive		N				
N		survive		URTI	5			
N		survive		N				
N		survive		N				
N		survive		N				
Y	4	survive		N				
Y	3	survive		N				
Y	5	survive		N				
Y	6	survive		N				
Y	4	died	Sepsis	N				
Y	9	survive		HAP	3			
Y	2	survive		N				
N		survive		N				

Y	3	survive		N	
Y	11	died	Cardio resp	Likely	7
N		died	Pneumonia	VAP	8
Y	5	survive		HAP	7
Y	5	died	MODS	N	
Y	4	survive		N	
Y	4	survive		N	
Y	4	survive		N	
Y	6	survive		N	
Y	4	survive		N	
Y	3	survive		N	
N		died	Oedem pul	Likely	10
N		survive		N	
Y	15	survive		HAP	8
Y	6	survive		Likely	14
N		died	Syok kardic	URTI	3
N		survive		N	
N		died	Apneu DNR	N	
N		survive		N	
N		survive		N	
N		survive		N	
N		survive		N	
N		survive		N	
N		survive		N	
N		survive		N	
N		survive		N	
N		survive		N	
N		survive		N	
N		survive		N	
N		survive		N	
N		survive		N	
N		survive		N	
N		survive		N	
N		survive		N	
N		survive		N	
N		survive		N	
N		survive		N	
N		survive		N	
N		survive		N	
N		survive		N	
Y	6	survive		CAUTI	47
N		survive		N	
N		survive		N	
N		survive		N	
N		survive		N	
Y	2	survive		N	
N		survive		N	
N		died	Oedem pul	N	

N		survive		N				
N		survive		URTI	4			
N		survive		N				
N		survive		N				
N		survive		N				
N		survive		Likely	9	Negatif	Bahan lainr	9
N		survive		N				
N		survive		N				
N		survive		N				
N		survive		N				
N		survive		N				
N		survive		N				
N		survive		N				
N		survive		N				
N		survive		UTI	5			
N		survive		N				
N		survive		N				
N		survive		N				
N		survive		N				
N		survive		N				
N		survive		N				
Y	6	survive		Flebitis	6			
N		survive		N				
Y	16	died	Herniasi	CAUTI	3	Enterococu	Urine	4
N		survive		N				
Y		survive		N				
Y	4	survive		N				
N		survive		N				
N		survive		N				
N		died	Herniasi	Likely	12			
Y	11	survive		N				
N		survive		N				
N		survive		N				
N		survive		N				
N		survive		N				
N		survive		N				
Y	5	survive		N				
N		survive		N				
N		survive		N				
N		survive		N				
N		survive		N				
N		survive		N				
N		survive		UTI	5			
N		survive		N				
Y	5	survive		N				
N		survive		N				
N		survive		N				
N		survive		UTI	6			

N		survive	N				
N		survive	N				
N		survive	N				
N		survive	N				
N		survive	N				
N		survive	N				
N		survive	N				
N		survive	N				
N		survive	N				
N		survive	N				
N		survive	N				
N		survive	N				
N		survive	N				
N		survive	N				
N		survive	Likely	19	Negatif	Blood	19
N		survive	N				
N		survive	N				
N		survive	N				
Y	3	survive	N				
N		survive	N				
N		survive	N				
N		survive	N				
N		survive	N				
N		survive	N				
N		survive	URTI	3			
N		survive	N				
N		survive	N				
N		survive	N				
N		survive	N				
N		survive	N				
N		survive	N				
N		survive	N				
N		survive	N				
Y	3	survive	N				
N		survive	N				
N		survive	N				
N		survive	N				
N		survive	N				
N		survive	N				
N		survive	N				
N		survive	N				
N		survive	N				
N		survive	N				
N		survive	N				
N		survive	N				
N		survive	N				
N		survive	N				
N		survive	N				

N		survive		N				
N		survive		N				
N		survive		N				
N		survive		N				
N		survive		N				
N		survive		N				
N		died	Cardiomyo	N				
N		survive		N				
N		survive		GIT	3			
N		survive		N				
N		survive		N				
N		survive		N				
N		survive		N				
Y	4	survive		N				
N		survive		N				
N		survive		URTI	6			
N		survive		N				
N		survive		N				
N		survive		N				
N		survive		N				
N		survive		N				
N		survive		UTI	5			
N		survive		N				
N		survive		N				
N		survive		N				
N		survive		UTI	3	Negatif	Urine	4
N		survive		N				
N		survive		N				
N		survive		N				
N		survive		N				
N		survive		N				
N		survive		N				
N		survive		N				
N		survive		N				
N		survive		N				
N		survive		N				
N		survive		N				
N		survive		N				
N		died	Syok Spetic	N				
N		survive		N				
N		survive		N				
N		survive		N				
N		survive		N				
N		survive		N				
N		survive		N				
N		survive		N				
N		survive		N				
N		survive		N				

N		survive	N				
N		survive	N				
N		survive	UTI	4			
N		survive	N				
N		survive	N				
N		survive	N				
N		survive	N				
N		survive	N				
N		survive	N				
N		survive	N				
N		survive	GIT	5			
N		survive	N				
N		survive	N				
N		survive	N				
N		survive	N				
N		survive	N				
N		survive	N				
N		survive	N				
N		survive	N				
N		survive	N				
N		survive	N				
N		survive	N				
N		survive	Flebitis	5			
N		survive	N				
N		survive	N				
Y	8	survive	N				
N		survive	N				
N		survive	UTI	4	Negatif	Urine	4
N		survive	N				
N		survive	N				
N		survive	N				
N		survive	N				
N		survive	N				
N		survive	N				
N		survive	N				
N		survive	N				
N		survive	N				
N		survive	N				
N		survive	N				
N		survive	N				
N		survive	N				
N		survive	N				
N		survive	N				
N		survive	N				
N		survive	N				
N		survive	N				
N		survive	N				
N		survive	N				

N		survive	N				
N		survive	N				
N		survive	N				
N		survive	N				
N		survive	UTI	4			
N		survive	N				
N		survive	N				
N		survive	UTI	14	Negatif	Urine	15
N		survive	N				
N		survive	N				
N		survive	HAP	18			
N		survive	N				
N		survive	N				
N		survive	GIT	9			
N		survive	N				
N		survive	N				
N		survive	N				
N		survive	N				
N		survive	N				
N		survive	N				
N		survive	N				
N		survive	N				
y	3	survive	N				
N		survive	N				
N		survive	N				
N		survive	N				
N		survive	N				
N		survive	URTI	3			
N		survive	N				
N		survive	N				
N		survive	N				
N		survive	N				
N		survive	N				
N		survive	N				
N		survive	N				
N		survive	N				
N		survive	N				
N		survive	N				
N		survive	N				
N		survive	N				
N		survive	URTI	3			
N		survive	N				
Y	6	survive	URTI	17			
N		survive	UTI	5	C.Glabrata	Urine	6
N		survive	likely	9			
N		survive	N				
Y	6	survive	N				
N		survive	N				

Y	4	survive		N				
N		survive		N				
N		survive		N				
N		survive		N				
N		survive		N				
N		survive		N				
N		survive		N				
N		survive		N				
N		survive		N				
N		survive		N				
N		survive		N				
N		survive		N				
N		survive		N				
N		died	Sepsis	HAP	8	Pseudomor	Sputum	8
Y	24	survive		CAUTI	9			
N		survive		N				
N		survive		N				
N		survive		URTI	7			
y	3	survive		N				
N		survive		N				
N		survive		N				
N		survive		N				
Y	12	survive		CAUTI	7	Klebsiella p	Urine	10
N		survive		N				
N		survive		N				
N		survive		HAP	13			
N		survive		N				
N		survive		N				
N		survive		GIT	6			
N		survive		N				
N		survive		N				
N		survive		GIT	7			
N		survive		N				
N		survive		N				
N		survive		N				
N		survive		UTI	5	E.Coli	Urine	6
N		survive		N				
N		survive		N				
N		survive		N				
N		survive		N				
N		survive		N				
N		survive		N				
N		survive		N				
N		survive		N				
N		survive		N				
N		survive		N				
Y	5	survive		N				
Y	4	survive		N				

Y	11	survive		N				
Y	9	survive		N				
N		survive		UTI	4			
N		survive		N				
N		survive		N				
N		survive		N				
N		survive		N				
N		survive		N				
N		survive		N				
N		survive		N				
N		survive		N				
Y	2	survive		HAP	5	Candida alb	Sputum	10
N		survive		N				
Y	4	survive		N				
Y	2	survive		N				
N		survive		N				
N		died	Respiratory	N				
Y	7	died	Sepsis	N				
N		survive		N				
N		survive		URTI	4			
N		survive		N				
N		survive		UTI	4			
N		survive		N				
N		survive		N				
N		survive		N				
N		survive		GIT	11			
N		survive		N				
N		survive		N				
N		survive		N				
N		survive		N				
N		survive		N				
N		survive		N				
N		survive		URTI	9			
N		survive		N				
N		survive		N				
N		survive		N				
N		survive		N				
N		survive		N				
N		survive		N				
N		survive		N				
N		survive		N				
N		survive		N				
N		survive		N				
N		survive		N				
N		survive		N				
N		survive		N				
N		survive		N				
N		survive		likely	29	Negatif	Blood	32

Y	6	survive		UTI	22			
N		survive		N				
N		survive		N				
N		survive		N				
N		survive		N				
N		survive		N				
N		survive		N				
N		survive		N				
N		survive		N				
N		survive		N				
N		survive		N				
N		survive		N				
N		survive		N				
N		survive		N				
N		survive		N				
N		survive		N				
N		survive		N				
Y	4	survive		N				
Y	5	survive		GIT	4			
N		survive		N				
Y	9	survive		N				
N		survive		GIT	15			
N		survive		UTI	10	Negatif	Urine	18
N		survive		N				
N		survive		N				
N		survive		N				
N		survive		UTI	6	Staphyloco	Urine	6
Y	6	survive		N				
N		survive		N				
N		survive		N				
N		survive		N				
N		survive		N				
N		survive		N				
N		survive		N				
N		survive		N				
N		survive		N				
N		survive		N				
N		survive		N				
N		survive		N				
N		survive		N				
N		survive		N				
N		survive		N				
N		survive		N				
N		survive		N				
N		survive		N				
N		died	DNR	N				
N		died	Kardio Resp	HAP	31			
N		died	Respiratory	HAP	23			

N		survive		N	
N		survive		N	
N		survive		N	
N		survive		N	
Y	8	survive		N	
N		survive		N	
N		survive		N	
N		survive		N	
N		survive		N	
N		survive		N	
N		survive		N	
N		died	Shock hipot	GIT	5
N		survive		N	
N		survive		N	
N		survive		N	
Y	8	survive		CAUTI	3
N		survive		N	
N		survive		URTI	4
N		survive		N	
N		survive		N	
N		survive		N	
N		survive		N	
N		survive		N	
N		survive		N	
N		survive		N	
N		survive		N	
N		survive		N	
N		survive		N	
N		survive		N	
N		survive		N	
N		survive		N	
N		survive		N	
N		survive		N	
N		survive		URTI	33
N		survive		N	
Y	8	survive		UTI	8
N		survive		N	
N		survive		GIT	4
N		survive		N	
N		survive		N	
N		survive		likely	15
N		survive		N	
N		survive		GIT	7
N		survive		N	
N		survive		N	
N		survive		N	
N		survive		N	
N		survive		N	

N		survive		N				
Y	3	survive		GIT	4			
N		survive		N				
N		survive		N				
N		survive		N				
N		survive		N				
N		survive		N				
N		survive		N				
N		survive		N				
N		survive		N				
N		survive		N				
N		survive		N				
N		survive		N				
N		survive		N				
N		survive		N				
N		survive		N				
N		survive		N				
N		survive		N				
N		survive		N				
N		survive		N				
N		survive		N				
N		survive		N				
N		survive		N				
N		survive		N				
N		survive		URTI	5			
N		survive		N				
N		survive		N				
N		survive		N				
N		survive		N				
N		survive		N				
Y	9	survive		N				
N		survive		likely	20	Negatif	Blood	20
N		survive		N				
N		survive		N				
N		survive		UTI	6			
N		survive		N				
N		died	Oedem par	likely	14			
N		survive		Flebitis	6			
N		survive		UTI	8	Negatif	Urine	8
N		survive		N				
N		survive		HAP	4			
N		survive		N				
N		survive		N				
N		survive		N				
N		survive		N				
N		survive		N				
N		survive		N				
N		survive		N				

N		survive		N				
N		survive		N				
N		survive		N				
N		survive		N				
N		survive		N				
N		survive		N				
N		survive		N				
N		survive		N				
N		survive		N				
Y	6	survive		UTI	6	Enterococu	Urine	9
N		survive		GIT	7			
N		survive		N				
N		survive		N				
N		survive		N				
N		survive		N				
N		survive		N				
N		survive		N				
N		survive		UTI	4			
N		survive		N				
Y	7	died	Cardiac ker	CAUTI	7			
N		survive		N				
Y	3	survive		N				
N		survive		N				
Y	4	survive		N				
Y	5	survive		N				
N		survive		N				
Y	6	survive		CAUTI	3			
Y	7	survive		N				
Y	7	survive		VAP	4	Klebsiella p	Aspirat trak	6
Y	5	survive		N				
Y	8	survive		VAP	8	Pseudomor	ETT	8
Y	10	survive		CAUTI	3			
Y	8	survive		Flebitis	3			
N		survive		N				
Y	4	survive		N				
Y	6	survive		N				
Y	17	survive		VAP	8	Klebsiella p	Sputum	8
Y	4	survive		N				
Y	2	survive		N				
Y	10	survive		N				
Y	3	survive		N				
Y	7	survive		N				
Y	7	survive		GIT	3			
Y	14	died	Respiratory	BSI	30	Sphigomon	Blood	30
Y	3	survive		N				
Y	21	died	Oedem pul	CAUTI	22			
N		died	Pneumonia	N				
Y	3	survive		N				

Y	7	survive		N				
Y	4	survive		N				
N		survive		N				
N		died	Asidosis me	N				
Y	3	survive		N				
N		survive		N				
Y	6	survive		N				
Y	12	died	Sepsis	likely	9			
Y	20	died	Sepsis	GIT	8			
Y	30	died	AKI	CAUTI	14	Negatif	Urine	15
N		survive		N				
Y	4	survive		N				
Y	5	died	Surgery	URTI	3			
Y	7	survive		N				
N		survive		N				
N		survive		N				
Y	4	survive		N				
Y	7	survive		N				
N		survive		N				
Y	6	survive		N				
Y	8	died	Pneumonia	N				
N		survive		N				
Y	11	survive		N				
Y	4	died	AKI	N				
N		died	Respiratory	N				
N		survive		N				
N		survive		N				
N		survive		N				
N		died	Kardio Resp	N				
N		survive		N				
Y	19	survive		N				
Y	6	died	Syok kardic	N				
Y	3	survive		N				
Y	10	died	ARDS	N				
Y	14	survive		N				
Y	11	died	Syok septic	VAP	3			
Y	4	survive		VAP	4			
Y	9	survive		HAP	3	Klebsiella p	ETT	7
Y	4	died	Prolonged :	N				
N		died	Syok	likely	11	Negatif	Blood	11
Y	5	survive		URTI	8			
N		survive		N				
N		survive		N				
N		survive		N				
N		survive		N				
N		survive		UTI	5	Negatif	Urine	6
N		survive		N				
N		survive		N				

Y	3	survive		N				
Y	5	survive		N				
N		survive		HAP	5	Klebsiella p	Sputum	9
N		survive		N				
N		survive		N				
N		survive		N				
N		survive		N				
N		survive		N				
N		survive		N				
N		survive		N				
N		survive		N				
Y	12	survive		Likely	6	Negatif	Blood	10
Y	5	survive		N				
N		survive		N				
Y	8	died	Syok septic	N				
Y	6	survive		N				
Y	5	died	Respiratory	N				
Y	3	died	Intra Cereb	N				
N		survive		N				
Y	3	died	Pneumonia	VAP	7	Staphyloco	Aspirat trak	13
Y	2	survive		N				
N		survive		N				
Y	11	survive		VAP	8			
Y	13	survive		SSI	17			
Y	23	survive		CAUTI	14			
Y	5	survive		HAP	11			
Y	11	died	Oedem pul	GIT	4			
Y	3	survive		N				
Y	8	died	Surgery	N				
Y	3	survive		Flebitis	6			
Y	8	died	Surgery	N				
Y	24	died	Respiratory	CAUTI	19			
Y	9	survive		N				
Y	7	survive		N				
Y	5	survive		N				
N		survive		N				
N		survive		N				
N		survive		N				
N		survive		UTI	11	E Cloacae, [illegible]	Urine	12
N		survive		N				
N		survive		N				
Y	2	survive		URTI	4	Pseudomor	Swab tengg	4
N		survive		N				
N		survive		N				
N		survive		N				
N		survive		N				
N		survive		N				
N		survive		N				

N		survive	N					
N		survive	N					
N		survive	N					
N		survive	N					
N		survive	N					
N		survive	URTI		9			
N		survive	N					
N		survive	N					
N		survive	N					
N		survive	UTI		4			
N		survive	N					
N		survive	N					
N		survive	N					
N		survive	N					
N		survive	N					
N		survive	GIT		5			
N		survive	N					
N		survive	N					
N		survive	N					
N		survive	N					
N		survive	UTI		4	Staphyloco	Urine	6
N		survive	URTI		8			
N		survive	N					
N		survive	GIT		5			
N		survive	N					
N		survive	N					
N		survive	N					
N		survive	N					
N		survive	UTI		4			
N		survive	URTI		3			
N		survive	N					
N		survive	N					
N		survive	N					
N		survive	N					
N		survive	URTI		8			
N		survive	N					
310	2179			6	5			
	7,029032			30	5			
	5,058365			45	10			
				14	12			
				59	22			

0,94186
0,901042
0,966851

INOS 2					INOS 3			
Nosocomia	When inos	Organism 2	specimen 2	Day of cultu	Nosocomia	When inos	Organism 3	specimen 3

GIT	6				
URTI	29			Flebitis	33
Likely	15	Negatif	Blood 15		
HAP	6				
GIT	8			Flebitis	13

VAP	4	Acinetobac	Aspirat trak	4

UTI	6	Likely	8	Negatif	Blood

URTI	21

UTI	5	E.Coli	Urine	5	Likely	7		
Likely	3							
Flebitis	20				CAUTI	20		
Flebitis	4							
HAP	23	E.Coli	ETT	25				
URTI	31				SSI	31	Klebsiella P	Cairan Ascit
HAP	22							

HAP	16

GIT	15	Pseudomor	Feces	23				
HAP	26	Pseudomor	ETT	26	SSI	60	Stenotroph	Dasar luka [illegible]
GIT	37	E Cloacae	Feces	37				
URTI	7							

CAUTI	8	Staphyloco	Urine	9
Likely	43			
VAP	9			

CAUTI	3

Likely	20	Negatif	Blood	22	CAUTI	24	Klebsiella p	Urine
UTI	10	E Coli, Ente	Urine	12	GIT	21		

Flebitis	55		
HAP	18		
BSI	10	Klebsiella p Blood	11
URTI	32		
GIT	5		

HAP	7
Flebitis	14

Flebitis	3

BSI	15	Klebsiella p	Blood	15	Likely	21	Negatif	Blood

GIT	45				Flebitis	9
UTI	11				Likely	49
HAP	17	Pseudomor	Sputum	27		

URTI	16
GIT	33

GIT	29

Flebitis	5

HAP	20
HAP	41

URTI	25
HAP	34
URTI	25

UTI	11		
VAP	21	Klebsiella p Aspirat Tral	19
VAP	22		

Likely	28	Negatif	Blood	30	URTI	44

Likely	17

CAUTI	10	E.Faecalis	Urine	16

HAP	12			
BSI	17	A Baumanii	CVC	17

URTI	8

URTI	6

URTI	21

UTI	12	Staphyloco	Urine	12
URTI	4			

VAP	6	Acinetobac Aspirat Tral	6		
HAP	22				
UTI	68	Acinetobac Urine	68	URTI	86
Flebitis	21				

HAP	8	Likely	27	Negatif	Blood
HAP	17				

GIT	7	Candida alk	Feces	11	Likely	18	Negatif	Blood
URTI	11							

URTI	6					
URTI	22					
GIT	3					
Flebitis	9					
VAP	8	Klebsiella p	Sputum	10		
CAUTI	41				HAP	43

URTI	10					
VAP	15				Likely	19
HAP	38	Acinetobac	Aspirat trak	38		
GIT	5					
UTI	11				HAP	22

HAP	8	CAUTI	12

GIT	4
UTI	3

5	7

	INOS 4					INOS 5		
Day of cultu	Nosocomia	When inos	Organism 4	specimen 4	Day of cultu	Nosocomia	When inos	Organism 5

GIT	34

9

31

70

24	HAP	51

21

27

18

0

		Other organism 1				Other organism 2		
specimen 5	Day of cultu	Other cultu	Other orga	Other speci	Day culture	Other orga	Other speci	Day culture
		N	N					
		N	N					
		Y	E.Coli	Feces	1			
		N	N					
		N	N					
		N	N					
		N	N					
		Y	Klebsiella P	Feces	1			
		N	N					
		N	N					
		Y	Negatif	Urine	2			
		N	N					
		N	N					
		N	N					
		N	N					
		N	N					
		Y	Streptococ	Sputum	2	Negatif	Sputum	5
		N	N					
		N	N					
		N	N					
		Y	Negatif	LCS	3			
		N	N					
		N	N					
		N	N					
		N	N					
		N	N					
		N	N					
		N	N					
		Y	Staphyloco	Cairan Peri	1	Negatif	Sputum	8
		N	N					
		N	N					
		N	N					
		N	N					
		Y	Negatif	Blood	1			
		N	N					
		N	N					
		N	N					
		N	N					
		Y	Klebsiella P	ETT	10			
		N	N					
		N	N					
		N	N					
		N	N					

N	N						
N	N						
Y	Negatif	Cairan ascit		5	Negatif	Blood	1
N	N						
N	N						
N	N						
N	N						
N	N						
N	N						
Y	Negatif	Blood		1	Negatif	Blood	1
Y	Negatif	Urine		8			
Y	Burkholder	Sputum		1	Negatif	Blood	1
N	N						
N	N						
N	N						
N	N						
Y	Negatif	LCS		2			
N	N						
Y	Negatif	Urine		26	Streptococ	Swab tengg	25
Y	Negatif	Blood		16	Enterococu	Cairan hidu	24
Y	Negatif	ETT		8			
Y	S Haemoliti	Blood		2	E. Coli	Feces	3
N	N						
N	N						
N	N						
N	N						
Y	Y	Pus		36			
Y	Negatif	Blood		1			
Y	E.Coli, Stap	Sputum		6			
Y	Negatif	LCS		6			
Y	Negatif	Blood		21			
N	N						
Y	Enterococu	Cairan lain-		3	Negatif	Blood	3
N	N						
N	N						
N	N						
Y	E. Coli	Swab tengg		2			
N	N						
N	N						
N	N						
N	N						
Y	Streptococ	Sputum		3	Enterococu	Sputum	4
Y	Coagulase r	Dasar luka		3	Serratia Ma	Aspirat trak	3
N	N						
Y	Negatif	Urine		9			
N	N						
Y	Klebsiella p	Sputum		3	Negatif	Sputum	4
N	N						

N	N					
N	N					
Y	E.Coli	Feces	2			
N	N					
Y	Negatif	LCS	3			
N	N					
N	N					
Y	Negatif	Blood	9			
N	N					
Y	Enterococu	Cairan hidu	4			
N	N					
N	N					
N	N					
N	N					
Y	Pseudomor	Swab teling	10			
Y	Klebsiella p	Feces	3			
N	N					
N	N					
N	N					
N	N					
N	N					
N	N					
N	N					
N	N					
N	N					
N	N					
N	N					
N	N					
N	N					
Y	Negatif	Blood	17			
N	N					
N	N					
N	N					
N	N					
N	N					
N	N					
N	N					
Y	Staphyloco	LCS	1	Negatif	Urine	12
N	N					
N	N					
N	N					
N	N					
N	N					
N	N					
Y	Negatif	LCS	8			
N	N					
Y	E.Coli	Urine	1			
N	N					

N	N					
N	N					
N	N					
N	N					
N	N					
N	N					
N	N					
N	N					
N	N					
N	N					
Y	Staphyloco	Abses cerel	28	Candida alb	Blood	28
N	N					
N	N					
N	N					
N	N					
N	N					
N	N					
N	N					
N	N					
Y	Enterococu	CVC	18	Klebsiella p	ETT	32
N	N					
N	N					
N	N					
N	N					
N	N					
N	N					
N	N					
N	N					
N	N					
N	N					
N	N					
N	N					
N	N					
N	N					
N	N					
N	N					
N	N					
Y	Negatif	Blood	1			
Y	Streptococ	Blood	1			
N	N					
N	N					
N	N					
Y	Negatif	Urine	5			
N	N					
N	N					
N	N					
N	N					
N	N					

Y	Staphyloco	Blood	2			
N	N					
N	N					
N	N					
N	N					
N	N					
N	N					
Y	Negatif	LCS	3			
N	N					
N	N					
N	N					
N	N					
N	N					
Y	Negatif	Urine	6			
N	N					
N	N					
N	N					
N	N					
N	N					
Y	Negatif	Swab faring	2			
N	N					
N	N					
N	N					
N	N					
N	N					
N	N					
N	N					
N	N					
N	N					
Y	Morganela	Urine	6			
N	N					
Y	Streptococ	Swab tengg	4	Negatif	Blood	13
N	N					
N	N					
Y	Negatif	Urine	18			
N	N					
N	N					
N	N					
N	N					
N	N					
Y	Negatif	Blood	11			
N	N					
N	N					
N	N					
N	N					
N	N					
N	N					
N	N					

N	N					
N	N					
N	N					
N	N					
Y	Negatif	Urine	2	Negatif	Urine	1
N	N					
N	N					
N	N					
Y	Negatif	Blood	2	Enterococu	ETT	3
N	N					
N	N					
N	N					
Y	Negatif	Blood	1			
N	N					
N	N					
N	N					
N	N					
N	N					
N	N					
N	N					
N	N					
Y	E.Coli	Aspirat Tral	4			
Y	Klebsiella P	Urine	1	E.Coli	Cairan Acit	2
Y	Stenotroph	ETT	15			
Y	Acinetobac	Aspirat Tral	1	Negatif	Blood	1
N	N					
Y	Cronobacte	Darah	1	Streptococ	Sputum	2
N	N					
Y	Negatif	Urine	2			
N	N					
N	N					
Y	Negatif	Blood	3	Streptococ	Bullae	6
N	N					
Y	Negatif	LCS	6	Klebsiella pneumonia, (		18
Y	Klebsiella P	ETT	4			
Y	Pseudomor	ETT	4			
N	N					
Y	Klebsiella p	ETT	3			
N	N					
Y	Negatif	Blood	1	Negatif	Cairan Ascit	11
Y	E.Coli, Stap	Blood	1	Staphyloco	Sputum	2
N	N					
N	N					
Y	Negatif	Blood	4	Pseudomor	Sputum	4
Y	Klebsiella p	ETT	24	Stenotropo	Cairan Ascit	38
N	N					
Y	Klebsiella p	Sputum	6	Negatif	Urine	22
Y	Neissera ca	ETT	2	Klebsiella p	Aspirat trak	9

Y	Kocuria rhi:	Aspirat trak	2	Staphyloco	Cairan lain-	2
Y	Acinetobac	ETT	9			
Y	Candida All	Cairan perit	4			
N	N					
Y	Streptococi	Swab tengg	7			
N	N					
N	N					
Y	Candida All	Urine	5			
N	N					
N	N					
N	N					
N	N					
N	N					
N	N					
N	N					
N	N					
Y	Negatif	Urine	8			
N	N					
N	N					
N	N					
N	N					
N	N					
N	N					
N	N					
Y	Negatif	Blood	10			
Y	Staphyloco	Urine	2			
N	N					
N	N					
Y	Staphyloco	ETT	15	Klebsiella p	Sputum	11
Y	Enterobact	Sputum	18	Staphyloco	Sputum	19
Y	Staphyloco	ETT	19	Acinetobac	Sputum	8
Y	E Coli, Canc	ETT	2	Negatif	Urine	12
N	N					
N	N					
N	N					
N	N					
N	N					
N	N					
N	N					
Y	Negatif	Blood	4			
Y	Salmonella	Urine	1			
Y	Negatif	Blood	3			
N	N					
N	N					
N	N					
N	N					
N	N					
N	N					

N	N						
N	N						
N	N						
N	N						
N	N						
Y	Negatif	Urine	43	Negatif	Urine	56
Y	Pseudomor	Sputum	6			
N	N					
N	N					
N	N					
N	N					
N	N					
N	N					
N	N					
N	N					
N	N					
N	N					
N	N					
N	N					
Y	Pseudomor	ETT	1	Stenotroph	ETT	17
N	N					
N	N					
N	N					
N	N					
N	N					
N	N					
N	N					
Y	Negatif	Blood	49	E faecalis, F	Aspirat trak	51
N	N					
Y	Pseudomor	ETT	2			
N	N					
N	N					
N	N					
N	N					
Y	Negatif	Urine	2	Enterococu	Urine	11
Y	Negatif	Sputum	4			
N	N					
N	N					
N	N					
N	N					
N	N					
N	N					
N	N					
N	N					
N	N					
Y	Routella pl:	ETT	2			
N	N					
N	N					

Y	Coagulase r	PUS	26			
Y	Negatif	Urine	15	Streptococc	Swab tengg	2
N	N					
N	N					
N	N					
N	N					
Y	Burkholder	Sputum	2			
N	N					
N	N					
N	N					
N	N					
Y	E.Coli	Feces	3			
N	N					
Y	Pseudomor	Swab tengg	3			
Y	Neissera ca	Swab tengg	4			
N	N					
N	N					
N	N					
N	N					
N	N					
N	N					
N	N					
N	N					
N	N					
N	N					
Y	Enterococu	Urine	7			
N	N					
Y	Citrobacter	Feces	2	Staphyloco	Urine	4
Y	Candida Alk	ETT	2			
Y	Staphyloco	ETT	7	Enterobact	Feces	2
N	N					
N	N					
N	N					
Y	Stenothrop	ETT	2			
Y	Negatif	Urine	3			
N	N					
N	N					
N	N					
N	N					
N	N					
N	N					
N	N					
N	N					
N	N					
N	N					
N	N					
N	N					
N	N					

Y	Negatif	Blood	1			
N	N					
N	N					
N	N					
N	N					
N	N					
N	N					
N	N					
N	N					
N	N					
N	N					
N	N					
N	N					
N	N					
N	N					
N	N					
N	N					
N	N					
N	N					
N	N					
N	N					
N	N					
N	N					
N	N					
Y	Streptococ	Sputum	6			
N	N					
N	N					
N	N					
N	N					
Y	E Coli	LCS	2	S Epidermi	Blood	6
Y	Pseudomor	Swab teling	2	Negatif	LCS	5
N	N					
N	N					
N	N					
N	N					
N	N					
N	N					
N	N					
Y	Burkholder	PUS	1	Staphyloco	Blood	3
Y	Staphyloco	ETT	3			
N	N					
Y	Stenothrop	ETT	3	Negatif	LCS	7
Y	Negatif	Blood	4			
N	N					
N	N					
Y	Klebsiella p	ETT	3			
N	N					
N	N					

N	N						
N	N						
N	N						
N	N						
N	N						
Y	E Coli	PUS	20				
N	N						
Y	Kluyvera As	Swab tengg	2				
N	N						
N	N						
N	N						
N	N						
Y	Negatif	Blood	3				
N	N						
Y	Negatif	Sputum	10	E.Coli	Sputum	14	
Y	Pseudomor	Urine	8	Enterococu	Urine	16	
Y	Negatif	Blood	1				
N	N						
Y	Acinetobac	Sputum	8				
N	N						
N	N						
N	N						
N	N						
N	N						
Y	Negatif	Blood	15	Klebsiella p	ETT	22	
Y	Negatif	LCS	1				
N	N						
N	N						
N	N						
N	N						
N	N						
Y	Streptococc	ETT	2				
N	N						
Y	Negatif	Sputum	7	E.Coli, cand	Sputum	9	
N	N						
N	N						
N	N						
N	N						
N	N						
Y	E Coli, Stap	Sputum	8				
N	N						
N	N						
Y	Negatif	Cairan ascit	22				
Y	Negatif	Blood	4	Negatif	Blood	8	
N	N						
Y	Stenotroph	Aspirat trak	2	Staphyloco	CVC	4	
N	N						
N	N						

Y	E Coli	Feces	2	Staphyloco	Blood	2
N	N					
N	N					
Y	Enterobact	Urine	16			
N	N					
N	N					
N	N					
Y	Pseudomor	ETT	2			
N	N					
N	N					
N	N					
N	N					
N	N					
N	N					
N	N					
N	N					
N	N					
N	N					
N	N					
Y	Negatif	Blood	10	Staphyloco	Blood	10
N	N					
Y	Negatif	Blood	2			
Y	Negatif	Blood	5			
N	N					
N	N					
N	N					
N	N					
N	N					
Y	Negatif	Blood	3			
N	N					
Y	Negatif	Blood	2			
N	N					
N	N					
N	N					
N	N					
N	N					
Y	Negatif	Urine	1	E.Coli	Feces	1
N	N					
Y	Negatif	Blood	1			
N	N					
N	N					
N	N					
N	N					
N	N					
N	N					
N	N					
Y	Kocuria kris	Urine	3	Negatif	Urine	17
N	N					

N	N					
N	N					
N	N					
N	N					
N	N					
N	N					
N	N					
N	N					
N	N					
N	N					
N	N					
N	N					
N	N					
N	N					
N	N					
Y	Negatif	Blood	2			
N	N					
N	N					
N	N					
N	N					
N	N					
Y	Negatif	PUS	4			
N	N					
N	N					
N	N					
N	N					
N	N					
N	N					
N	N					
N	N					
N	N					
N	N					
Y	Negatif	PUS	6			
N	N					
N	N					
N	N					
N	N					
N	N					
N	N					
N	N					
N	N					
N	N					
N	N					
N	N					
N	N					
N	N					
Y	Serratia mc	ETT	5	Klebsiella p	Feces	18
N	N					

N	N						
N	N						
N	N						
Y	Negatif	Urine	3	Negatif	Blood	6
Y	Klebsiella p	Cairan bula	11			
Y	Acinetobac	ETT	3			
N	N					
N	N					
N	N					
N	N					
N	N					
N	N					
N	N					
N	N					
N	N					
N	N					
N	N					
N	N					
N	N					
N	N					
N	N					
Y	E.Coli	feces	1			
N	N					
N	N					
N	N					
N	N					
N	N					
N	N					
N	N					
N	N					
N	N					
N	N					
N	N					
N	N					
N	N					
N	N					
N	N					
N	N					
N	N					
Y	Negatif	Urine	19			
N	N					
N	N					
Y	Negatif	Urine	11			
N	N					
N	N					
N	N					
N	N					
N	N					

N	N					
N	N					
N	N					
N	N					
N	N					
N	N					
N	N					
N	N					
N	N					
N	N					
N	N					
N	N					
N	N					
N	N					
N	N					
N	N					
N	N					
N	N					
N	N					
N	N					
N	N					
N	N					
N	N					
N	N					
N	N					
N	N					
N	N					
N	N					
N	N					
N	N					
N	N					
N	N					
N	N					
Y	Coagulative	Cairan bula	5	Klebsiella p	Blood	21
Y	Enterobact	PUS	10	Acinetobac	Urine	11
Y	Klebsiella p	Swab Tengg	17	Negatif	Urine	20
Y	Negatif	Blood	23	Candida alb	Swab tengg	25
N	N					
N	N					
N	N					
Y	Staphyloco	Telinga kiri	5	Serratia Fo	Swab teling	5
N	N					
N	N					
N	N					
N	N					
Y	Enterococu	ETT	12			
N	N					
N	N					

N	N					
N	N					
N	N					
N	N					
N	N					
N	N					
Y	Negatif	Blood	4	Negatif	Blood	8
Y	Negatif	Blood	3			
N	N					
N	N					
N	N					
N	N					
N	N					
N	N					
N	N					
N	N					
N	N					
N	N					
N	N					
N	N					
N	N					
N	N					
N	N					
N	N					
N	N					
N	N					
N	N					
N	N					
N	N					
N	N					
N	N					
N	N					
N	N					
N	N					
N	N					
N	N					
N	N					
N	N					
Y	Negatif	Blood	4			
Y	Negatif	Sputum	16	Negatif	Sputum	17
Y	Pseudomor	ETT	4	Pseudomor	Sputum	7
Y	Klebsiella P	ETT	31			
N	N					
Y	Klebsiella P	Sputum	1	Negatif	Blood	6
Y	Candida Alb	ETT	1	Negatif	Blood	6
Y	E.Coli, Ente	PUS	8			
Y	Negatif	Urine	4			
N	N					

N	N					
N	N					
N	N					
N	N					
N	N					
N	N					
N	N					
N	N					
N	N					
N	N					
N	N					
Y	Roultella O	Sputum	17			
Y	Negatif	Urine	1	Negatif	Urine	13
N	N					
N	N					
N	N					
N	N					
N	N					
N	N					
N	N					
N	N					
N	N					
N	N					
Y	Klebsiella P	Sputum	20			
Y	Klebsiella P	Sputum	10			
Y	Candida Alb	Swab Tengg	2	Pseudomor	ETT	5
N	N					
N	N					
N	N					
N	N					
N	N					
N	N					
N	N					
Y	Stenotroph	LCS	2	Klebsiella P	Feces	36
Y	Acinetobac	PUS	27			
Y	Negatif	Blood	12			
N	N					
N	N					
N	N					
N	N					
N	N					
N	N					
N	N					
N	N					
N	N					
Y	Negatif	Sputum	4	Negatif	Sputum	8
Y	E.Coli, Pseu	Cairan usus	6	Pseudomor	PUS	6
N	N					

	N	N					
	N	N					
	N	N					
	Y	Staphyloco	Blood	21	Staphyloco	CVC	21
	Y	Negatif	Urine	14			
	Y	Negatif	Blood	1			
	N	N					
	N	N					
	N	N					
	Y	E.Coli	Feces	3	Negatif	Sputum	18
	N	N					
	N	N					
	N	N					
	N	N					
	N	N					
	N	N					
	N	N					
	N	N					
	Y	Citrobacter	Urine	3	Negatif	Urine	25
	N	N					
	N	N					
	Y	Negatif	Urine	2	Negatif	Urine	4
	N	N					
	N	N					
	N	N					
	N	N					
	N	N					
	N	N					
	N	N					
	Y	Negatif	Blood	5	Serratia Ma	Sputum	16
	N	N					
	Y	E.Coli	Feces	2			
	Y	Negatif	Blood	6			
	N	N					
	N	N					
	N	N					
	N	N					
	N	N					
	N	N					
	N	N					
	N	N					
	N	N					
	N	N					
	N	N					
	N	N					
	N	N					
	N	N					
	Y	Negatif	Blood	19	Klebsiella P	ETT	21

N	N					
N	N					
N	N					
N	N					
Y	E Coli	Urine	3			
N	N					
N	N					
N	N					
Y	Klebsiella P	ETT	6			
Y	E Coli, Pseu	Sputum	3			
N	N					
N	N					
N	N					
N	N					
N	N					
N	N					
N	N					
N	N					
N	N					
N	N					
N	N					
N	N					
Y	Negatif	Blood	1	E Coli	Feces	3
Y	Negatif	Blood	2			
N	N					
Y	Staphyloco	Blood	1			
Y	Negatif	Aspirat trak	13			
N	N					
N	N					
N	N					
N	N					
N	N					
N	N					
Y	E Coli	Urine	5			
N	N					
N	N					
N	N					
N	N					
N	N					
Y	Klebsiella p	Aspirat trak	6			
N	N					
N	N					
Y	Acinetobac	ETT	4	Acinetobac	ETT	5
N	N					
N	N					
N	N					
Y	Candida Alb	Urine	2	Enterobact	Aspirat trak	3
N	N					

N	N					
N	N					
N	N					
N	N					
N	N					
N	N					
Y	Stapylococ	Blood	5			
Y	E Coli	Urine	1			
N	N					
N	N					
N	N					
N	N					
N	N					
N	N					
N	N					
N	N					
N	N					
N	N					
N	N					
N	N					
N	N					
N	N					
N	N					
N	N					
N	N					
N	N					
N	N					
N	N					
N	N					
N	N					
N	N					
Y	Klebsiella P	Aspirat Tral	5	Klebsiella P	Blood	10
N	N					
N	N					
N	N					
N	N					
N	N					
N	N					
N	N					
N	N					
Y	Serratia ma	Sputum	2			
Y	Klebsiella p	Feces	11	E Coli, E fae	Feces	50
Y	Staphyloco	Blood	19			
Y	Enterococu	Blood	1			
N	N					
Y	Negatif	Blood	2			
N	N					
N	N					

N	N					
N	N					
N	N					
N	N					
N	N					
N	N					
N	N					
N	N					
Y	Negatif	Urine	3			
N	N					
N	N					
N	N					
N	N					
N	N					
Y	Pseudomor	LCS	2			
N	N					
N	N					
N	N					
N	N					
N	N					
N	N					
N	N					
N	N					
N	N					
N	N					
N	N					
Y	Negatif	Blood	1	Staphyloco	ETT	4
N	N					
N	N					
N	N					
N	N					
N	N					
N	N					
N	N					
Y	Enterobact	Ujung ETT	10			
Y	Providena S	Urine	2	Staphyloco	Blood	6
N	N					
N	N					
N	N					
N	N					
Y	Candida Alb	Sputum	4			
N	N					
N	N					
N	N					
N	N					
N	N					
N	N					
N	N					

N	N					
N	N					
N	N					
N	N					
N	N					
N	N					
N	N					
N	N					
Y	Negatif	Sputum	2			
N	N					
Y	Negatif	Blood	6			
N	N					
N	N					
N	N					
Y	Negatif	urine	1			
N	N					
N	N					
N	N					
N	N					
Y	Staphyloco	Urine	2			
N	N					
N	N					
N	N					
Y	Kocuria Kris	Urine	2			
N	N					
Y	Staphyloco	LCS	7			
N	N					
N	N					
N	N					
Y	Negatif	Urine	2	Negatif	Blood	15
Y	Negatif	Efusi perica	8	Klebsiella p	Efusi perica	8
N	N					
N	N					
N	N					
N	N					
N	N					
N	N					
N	N					
N	N					
N	N					
N	N					
N	N					
N	N					
Y	Pseudomor	Aspirat trak	3	Candida pa	Urine	6
N	N					
Y	Streptomor	Blood	1			
N	N					
N	N					

N	N							
N	N							
N	N							
N	N							
N	N							
N	N							
N	N							
N	N							
N	N							
N	N							
N	N							
N	N							
N	N							
N	N							
N	N							
N	N							
Y	Negatif	Blood		13				
Y	Staphyloco	Blood		5	Negatif	Blood		4
N	N							
N	N							
N	N							
N	N							
N	N							
Y	Salmonella	LCS		2				
Y	Serratia for	Sputum		16				
N	N							
N	N							
Y	Negatif	Feces		4				
N	N							
N	N							
N	N							
N	N							
Y	Negatif	Blood		2	Negatif	Urine		3
N	N							
N	N							
N	N							
N	N							
N	N							
N	N							
N	N							
N	N							
N	N							
N	N							
N	N							
N	N							
N	N							
N	N							
N	N							

N	N					
N	N					
N	N					
N	N					
N	N					
N	N					
Y	Negatif	Urine	2	Negatif	Blood	4
N	N					
N	N					
N	N					
N	N					
N	N					
N	N					
N	N					
Y	Negatif	Urine	2			
N	N					
N	N					
N	N					
N	N					
N	N					
Y	Negatif	Urine	5	Negatif	Urine	13
Y	Klebsiella p	Aspirat trak	4	Staphyloco	CVC	5
Y	E Coli	ETT	1	Klebsiella p	ETT	9
Y	E Cloacae, S	Swab tengg	3			
Y	E Cloacae, S	Dasar luka	2			
Y	Streptococi	Aspirat trak	1	E Faecalis, I	ETT	4
N	N					
N	N					
Y	Negatif	Urine	8			
N	N					
N	N					
N	N					
N	N					
N	N					
Y	Streptococi	Urine	1			
N	N					
N	N					
N	N					
N	N					
N	N					
N	N					
N	N					
Y	Staphyloco	Aspirat trak	2	Candida alb	ETT	3
Y	Streptococi	ETT	3			
Y	Negatif	Blood	1			
Y	Negatif	Blood	1	Negatif	LCS	1
N	N					
N	N					

N	N					
Y	E.Coli, P aei	ETT	11			
Y	Pseudomor	ETT	2	E Coli	Cairan lain2	4
Y	E. Cloacae,	Aspirat Tral	2			
Y	Candida trc	Swab phary	4			
N	N					
Y	Negatif	Blood	3	E Coli	ETT	4
N	N					
N	N					
N	N					
N	N					
N	N					
N	N					
N	N					
N	N					
N	N					
N	N					
N	N					
N	N					
N	N					
N	N					
N	N					
Y	S Haemoliti	Urine	2			
N	N					
N	N					
N	N					
N	N					
N	N					
Y	Negatif	Blood	2			
N	N					
N	N					
N	N					
N	N					
Y	Negatif	Blood	1			
N	N					
N	N					
N	N					
N	N					
N	N					
N	N					
Y	Negatif	Blood	37	Negatif	PUS	43
N	N					
N	N					
N	N					
N	N					
N	N					
N	N					
Y	Negatif	Blood	1			

N	N					
N	N					
N	N					
N	N					
N	N					
Y	E Coli	Cairan perit	1			
N	N					
N	N					
Y	Negatif	Urine	4			
N	N					
N	N					
N	N					
N	N					
N	N					
N	N					
N	N					
Y	Negatif	PUS	29			
N	N					
N	N					
N	N					
N	N					
Y	Candida alk	Aspirat Tral	22			
N	N					
Y	Pseudomor	ETT	6	Negatif	Blood	10
N	N					
N	N					
N	N					
N	N					
Y	Staphyloco	Urine	5			
Y	Stenotropo	Blood	8			
Y	Negatif	Blood	2			
N	N					
N	N					
N	N					
N	N					
N	N					
N	N					
N	N					
N	N					
N	N					
N	N					
N	N					
N	N					
N	N					
Y	Negatif	Sputum	1			
N	N					
Y	Negatif	Blood	1			
N	N					

N	N					
N	N					
N	N					
Y	Negatif	Blood	3			
N	N					
Y	Negatif	Urine	5			
Y	Klebsiella p	Feces	2			
N	N					
N	N					
N	N					
Y	Negatif	Blood	2			
N	N					
N	N					
N	N					
N	N					
N	N					
N	N					
Y	Negatif	Cairan diali	1	Negatif	Blood	11
Y	Negatif	Cairan diali	1			
N	N					
N	N					
N	N					
N	N					
N	N					
N	N					
N	N					
N	N					
N	N					
N	N					
N	N					
N	N					
N	N					
N	N					
N	N					
N	N					
N	N					
N	N					
N	N					
N	N					
N	N					
N	N					
N	N					
N	N					
N	N					
N	N					
N	N					
N	N					
N	N					

N	N						
N	N						
N	N						
N	N						
N	N						
Y	Negatif	Blood	2	Klebsiella P	Feces		5
N	N						
N	N						
N	N						
N	N						
N	N						
N	N						
N	N						
N	N						
N	N						
N	N						
N	N						
N	N						
N	N						
N	N						
N	N						
N	N						
N	N						
N	N						
Y	Negatif	Urine	2				
N	N						
Y	Negatif	Urine	3				
N	N						
Y	Negatif	Urine	2				
N	N						
Y	Negatif	Blood	1				
N	N						
N	N						
N	N						
N	N						
N	N						
Y	Negatif	Urine	2				
Y	Negatif	LCS	2				
N	N						
N	N						
N	N						
N	N						
N	N						
N	N						
N	N						
N	N						
N	N						
Y	Negatif	Urine	18	Klebsiella P	Feces		18

N	N					
N	N					
N	N					
N	N					
N	N					
N	N					
N	N					
N	N					
N	N					
N	N					
N	N					
Y	Candida Al	Aspirat trak	1			
N	N					
N	N					
Y	Staphyloco	Urine	3	Acinetobac	Swab tengg	6
N	N					
N	N					
N	N					
N	N					
N	N					
N	N					
N	N					
N	N					
N	N					
N	N					
Y	Streptococ	Swab tengg	5			
N	N					
N	N					
N	N					
N	N					
N	N					
N	N					
N	N					
Y	Negatif	Urine	2			
N	N					
N	N					
N	N					
N	N					
Y	Negatif	Urine	2			
N	N					
N	N					
N	N					
N	N					
N	N					
N	N					
N	N					
N	N					
N	N					

N	N					
N	N					
N	N					
Y	Negatif	Sputum	4	Klebsiella P	Sputum	9
N	N					
Y	E.Coli	Sputum	2			
N	N					
N	N					
N	N					
N	N					
Y	Enterobact	Blood	8			
N	N					
N	Negatif	Sputum	9			
Y	N					
N	N					
N	N					
N	N					
N	N					
N	N					
N	N					
N	N					
N	N					
N	N					
N	N					
N	N					
N	N					
N	N					
N	N					
N	N					
N	N					
N	N					
N	N					
N	N					
N	N					
N	N					
N	N					
N	N					
N	N					
N	N					
N	N					
N	N					
N	N					
N	N					
Y	Negatif	Urine	6	Negatif	Urine	2
N	N					
N	N					
N	N					
N	N					

N	N					
N	N					
N	N					
N	N					
N	N					
N	N					
N	N					
N	N					
Y	Acinetobac	Urine	2			
N	N					
N	N					
N	N					
N	N					
Y	Negatif	Blood	3	Negatif	Blood	37
Y	Negatif	Urine	2	Negatif	Blood	5
N	N					
N	N					
Y	Negatif	Blood	11			
Y	Negatif	Blood	2	Staphyloco	LCS	2
N	N					
N	N					
N	N					
Y	Negatif	Blood	7			
N	N					
N	N					
Y	Negatif	Blood	4	Negatif	Blood	12
Y	Klebsiella p	Feces	6	Negatif	Urine	15
Y	Candida alk	Urine	2	Candida All	Urine	8
N	N					
N	N					
N	N					
N	N					
N	N					
N	N					
N	N					
N	N					
N	N					
N	N					
N	N					
N	N					
N	N					
N	N					
N	N					
N	N					
N	N					
N	N					
Y	E.Coli	Aspirat Tral	11	Candida All	Aspirat Tral	11
Y	Negatif	Blood	2	Staphyloco	LCS	2

Y	Negatif	Blood	2	Negatif	ET Sekret	2
Y	Negatif	Urine	2			
N	N					
N	N					
N	N					
N	N					
N	N					
N	N					
N	N					
N	N					
Y	Negatif	Urine	5			
N	N					
Y	Candida tro	Swab tengg	19			
Y	Negatif	Blood	1	Klebsiella p	Aspirat Tra	3
Y	Negatif	Blood	1	Negatif	Blood	4
N	N					
Y	Enterococu	Swab tengg	2	Negatif	Blood	2
Y	Elizabethki	Blood	1			
N	N					
Y	E faecalis	Urine	19	Klebsiella pneumonia		28
Y	Negatif	Cairan CAP	2			
N	N					
N	N					
N	N					
N	N					
Y	Staphyloco	Blood	1			
N	N					
N	N					
N	N					
N	N					
N	N					
N	N					
N	N					
Y	Negatif	Urine	5			
N	N					
N	N					
N	N					
N	N					
N	N					
N	N					
Y	Brevundim	Urine	5			
N	N					
N	N					
Y	Negatif	Cairan ascit	4			
N	N					
N	N					
N	N					
Y	Negatif	Blood	8			

Y	Candida pa	Urine	7			
N	N					
N	N					
N	N					
Y	Staphyloco	Blood	1			
N	N					
N	N					
N	N					
N	N					
N	N					
N	N					
N	N					
N	N					
N	N					
N	N					
N	N					
N	N					
Y	Enterobact	Blood	1	Negatif	LCS	4
Y	Klebsiella P	Aspirat Tral	20			
Y	Negatif	Blood	5	Negatif	Blood	19
Y	Negatif	Blood	11	Negatif	Urine	3
Y	Negatif	Sputum	1			
Y	Serratia Far	Urine	2	Negatif	Urine	18
Y	Negatif	Blood	1			
N	N					
N	N					
Y	Klebsiella P	Urine	9			
Y	E Cloacae	Urine	2			
N	N					
N	N					
N	N					
Y	E Coli	PUS	3			
N	N					
N	N					
N	N					
N	N					
N	N					
N	N					
Y	Staphyloco	Urine	2			
N	N					
N	N					
N	N					
N	N					
N	N					
N	N					
N	N					
Y	Negatif	Blood	8	Negatif	Blood	23
Y	Negatif	Blood	1	Negatif	Cairan CAPI	14

N	N					
N	N					
N	N					
N	N					
Y	Negatif	Blood	1	Negatif	Sputum	3
N	N					
N	N					
N	N					
N	N					
N	N					
N	N					
N	N					
N	N					
N	N					
N	N					
Y	Negatif	Blood	3			
N	N					
N	N					
Y	Klebsiella p	Swab tengg	1			
N	N					
N	N					
N	N					
N	N					
N	N					
N	N					
N	N					
N	N					
N	N					
N	N					
N	N					
N	N					
N	N					
N	N					
Y	Staphyloco	Blood	2	Negatif	Blood	16
N	N					
N	N					
Y	Streptococ	Blood	2			
N	N					
N	N					
N	N					
N	N					
N	N					
Y	Negatif	Urine	14			
N	N					
N	N					
N	N					
N	N					
N	N					

N	N					
N	N					
N	N					
N	N					
N	N					
N	N					
N	N					
N	N					
N	N					
N	N					
Y	Negatif	Blood	4			
N	N					
N	N					
N	N					
N	N					
N	N					
N	N					
N	N					
N	N					
N	N					
N	N					
N	N					
N	N					
N	N					
N	N					
N	N					
N	N					
N	N					
N	N					
N	N					
Y	Enterobact	ETT	2	Negatif	Blood	3
Y	Negatif	Sputum	7	E Coli, Strep	Feces	40
Y	Coagulase	ETT	3			
N	N					
Y	Negatif	Blood	3			
N	N					
N	N					
N	N					
N	N					
N	N					
N	N					
Y	Negatif	Blood	2			
N	N					
N	N					
N	N					
N	N					
N	N					
N	N					

N	N					
N	N					
N	N					
N	N					
N	N					
N	N					
N	N					
N	N					
N	N					
Y	Enterococu	Urine	5	Pseudomor	Aspirat trak	17
N	N					
N	N					
N	N					
N	N					
N	N					
N	N					
N	N					
N	N					
N	N					
N	N					
N	N					
Y	Negatif	Blood	2			
Y	Enterococu	Cairan lain	1	Pseudomor	ETT	7
N	N					
Y	Enterococu	Aspirat Tral	2			
N	N					
Y	Negatif	Blood	2	Acinetobac	ETT	4
N	N					
Y	Negatif	Blood	1	Negatif	Urine	3
Y	Negatif	Aspirat Tral	1			
Y	Negatif	Blood	1	Enterococu	Aspirat trak	6
N	N					
Y	Candida du	Aspirat Tral	2	Pseudomor	ETT	8
Y	Negatif	Blood	1			
Y	Brevibacter	Blood	1			
N	N					
Y	Negatif	Blood	1			
Y	Pseudomor	ETT	1	Enterococu	Urine	3
N	N					
Y	Negatif	Blood	2	Streptococ	ETT	4
Y	Sphingomo	Blood	1	Candida All	Aspirat trak	2
Y	Klebsiella p	ETT	1	Enterococu	Cairan lain-	7
Y	Negatif	Blood	1	Negatif	Blood	3
Y	E Coli	Cairan Drai	29	Negatif	Urine	25
N	N					
Y	Negatif	Blood	1	Strenophor	ETT	11
N	N					
N	N					

Y	Pseudomor	ETT	2			
Y	Negatif	Blood	1			
N	N					
N	N					
N	N					
N	N					
Y	Negatif	Blood	1	Negatif	Urine	2
Y	Negatif	Blood	2	Candida Tr	Aspirat trak	3
Y	Staphyloco	Blood	1	Candida alb	Sputum	2
Y	E Coli, Stap	ETT	1	Negatif	Blood	1
N	N					
N	N					
Y	Negatif	Urine	2			
Y	E Coli	ETT	7			
N	N					
N	N					
Y	Staphyloco	Blood	2			
Y	Klebsiella P	Feces	2			
N	N					
Y	Negatif	Blood	1			
N	N					
Y	Negatif	Blood	2	E.Coli, Stap	ETT	4
N	N					
N	N					
Y	Negatif	Blood	1	Pseudomor	Aspirat Tral	3
N	N					
N	N					
N	N					
Y	Klebsiella p	ETT	2			
N	N					
N	N					
Y	Clonobacte	Blood	3	Enterobact	Aspirat trak	7
N	N					
N	N					
Y	Pseudomor	Aspirat Tral	10			
Y	Negatif	Blood	1	Staphyloco	Aspirat trak	1
N	N					
Y	E.Coli, Strep	Cairan lain	2			
N	N					
N	N					
N	N					
N	N					
N	N					
N	N					
Y	Negatif	Blood	1	Klebsiella p	Feces	2
Y	Negatif	Urine	6			
N	N					
N	N					

Y	Streptococı	Aspirat Tral	2	Staphyloco	LCS	3
N	N					
Y	Negatif	Blood	8			
N	N					
N	N					
N	N					
N	N					
N	N					
N	N					
N	N					
N	N					
N	N					
Y	E. Coli, Cryp	Feces	1	E. Coli	Feces	2
N	N					
Y	Enterobact	ETT	4	Staphyloco	ETT	7
Y	Staphyloco	Blood	1			
N	N					
N	N					
N	N					
Y	Klebsiella p	ETT	3	S Maltophil	Cairan lain-	10
Y	Negatif	Urine	3			
N	N					
Y	Acinetobac	Cairan lain	3	Enterobact	ETT	5
Y	Enterobact	Aspirat Tral	3	Negatif	ETT	6
Y	Enterobact	Aspirat Tral	1	Negatif	Urine	9
Y	Negatif	Blood	1	Enterobact	Sputum	3
N	N					
N	N					
Y	Negatif	Blood	2	Negatif	Blood	7
Y	Negatif	Blood	2	E Coli, E Fae	Endotracea	3
Y	Negatif	Aspirat Tral	5	Pseudomor	ETT	26
Y	Pseudomor	Aspirat Tral	30	Negatif	Blood	40
Y	Enterococu	Aspirat Tral	1	Klebsiella p	ETT	6
Y	Klebsiella p	Bloos	7			
Y	E coli	Feces	3			
N	N					
N	N					
N	N					
N	N					
N	N					
N	N					
N	N					
N	N					
N	N					
N	N					
N	N					
N	N					
N	N					

N	N					
N	N					
N	N					
N	N					
N	N					
N	N					
N	N					
N	N					
N	N					
N	N					
N	N					
N	N					
N	N					
N	N					
N	N					
N	N					
N	N					
N	N					
N	N					
Y	Negatif	Blood	1	Negatif	Urine	2
N	N					
N	N					
N	N					
Y	Candida trc	Sputum	14			
N	N					
N	N					
N	N					
Y	Negatif	Blood	1	klebsiella p	Sputum	2
Y	Negatif	Blood	3			
N	N					
N	N					
N	N					
N	N					
N	N					
N	N					
N	N					

Other organism 3			Other organism 4			Other organism 5		
Other orga	Other speci	Day culture	Other orga	Other speci	Day culture	Other orga	Other speci	Day culture

Streptococ	Cairan ascit	11			
Negatif	Sputum	2	Streptococ	ETT	3
Negatif	Sputum	2	Negatif	Sputum	3
Klebsiella p	Feces	12			
Negatif	Blood	15			

Negatif	Cairan drai	4			
Negatif	Urine	2			
E Coli	Kateter uri	6	Candida alk	Urine	9
Negatif	Blood	15			
P Aerugino:	Aspirat Tral	3	Klebsiella P	ETT	34
Staphyloco	Cairan lain	24			
Staphyloco	ETT	16			

Negatif	Sputum	19	Negatif	Sputum	21	Stenotroph	Endotrakea	10
Candida Alb	Urine	7						

E Cloacae	Cairan drai	54	Negatif	PUS	71

Klebsiella p Feces	22

Pseudomor	Swab teling	5
Negatif	Blood	8

Negatif	Sputum	18	Klebsiella P	ETT	18	Acinetobac	ETT	24
Stenotroph	ETT	19						
Staphyloco	ETT	9	E Coli	Kateter	3			

Negatif	Urine	17
Klebsiellla F	Sputum	6

Negatif	Aspirat Tral	25
Serratea m	Sputum	20

Staphyloco	ETT	18			
Pseudomor	ETT	24	Negatif	Blood	30
Negatif	Efusi perica	12	Staphyloco	Sputum	16
Staphyloco	ETT	18			

Negatif	Blood	46

E Coli	Urine	4	Negatif	Blood	8	Negatif	Blood	15
Negatif	Blood	1	Negatif	Blood	22	Negatif	Blood	36
Klebsiella P	Feces	7						

Staphyloco	LCS	12	E Faecalis, (	Urine	10

Negatif	Cairan lain	9
Negatif	Urine	4

Negatif		5	16						
Streptococ	ET		4						
Negatif	Blood		22						
Candida alb	Urine		9	Klebsiella p	PUS	9	Klebsiella p	Feces	12
Negatif	Darah		19						

Candida Pa Urine	7

Candida lus	ETT	9	Negatif	Sputum	16			
Negatif	Blood	56	Negatif	Blood	66	Negatif	Blood	26
Rhudotorul	Cairan CAP	14	Negatif	Blood	34	Negatif	Cairan diasi	35

Pseudomor	Swab tengg	20	Negatif	Urine	28	Negatif	Blood	28

Streptococı	ETT	8

Negatif	Blood	17	Negatif	Urine	17	Negatif	Blood	24
Pseudomor	ETT	11						
Acinetobac	ETT	15						
Klebsiella p	ETT	6						
Serratia ma	Blood	10	Streptococ	Sputum	11	Streptococ	ETT	12
Klebsiella p	Aspirat trak	5	Klebsiella p	Sputum	10			
S Maltophil	ETT	10	Strenophor	Aspirat trak	17	Negatif	Blood	22

Enterococu	Feces	4			
E Coli, Ente	Feces	19			
Negatif	LCS	5			
Negatif	Sputum	3	Streptococ	Sputum	3

Negatif	Urine	3	Negatif	Urine	4			
Negatif	Blood	4	Negatif	Urine	4			
Staphyloco	Aspirat trak	13						
Klebsiella p	Cairan lain-	14						
Enterobact	Cairan lain-	10	Candida alb	Blood	11			
Stenothrop	Sputum	16	Staphyloco	Blood	57	Enterococu	Aspirat trak	57
Negatif	Urine	4	Pseudomor	ETT	10	Negatif	LCS	19
E Coli	Swab luka	11	Negatif	Blood	16			
Negatif	Urine	46	Enterococu	Aspirat trak	1	Negatif	Blood	1

Negatif	Blood	3	Negatif	Blood	14
Negatif	Sputum	3			

Other organism 6			Other organism 7			Other organism 8		
Other orga	Other speci	Day culture	Other orga	Other speci	Day culture	Other orga	Other speci	Day culture

E cloacae, e	Swab tengg	17
Enterobact	Swab tengg	49

Negatif	Blood	19

Negatif	Cairan lain	28	Negatif	Blood	37

Negatif	Aspirat trak	24

Pseudomor	Aspirat trak	127

LOS			Antibiotics			
LOS	LOS in wards	overall LOS	Antibiotic	Irrationality	Type Irrationali	Type of first antibiot
5		5	Y	N		Ampicilin
3		3	N	N		
11		11	Y	Y	Spektrum	Cefotaxime
10		10	Y	Y	Spektrum	Amoxicilin
7		7	Y	N		OAT FOC
6		6	N	N		
6		6	Y	Y	Spektrum	Ceftriaxone
6		6	Y	N		Ampicilin
3		3	Y	N		Ampicilin
3		3	Y	Y	Spektrum	Cefotaxime
8		8	Y	Y	Spektrum	Ceftriaxone
12		12	N	N		
7		7	N	N		
16		16	Y	Y	Dose	Ampicilin
3		3	N	N		
6		6	N	N		
5		5	N	N		
4		4	N	N		
5		5	N	N		
4		4	Y	Y	Spektrum	Cefotaxime
21		21	Y	N		Ceftriaxone
4		4	Y	Y	Spektrum	Cefixime
5		5	Y	Y	Spektrum	Cefotaxime
4		4	Y	Y	Spektrum	Ampicilin
5		5	N	N		
5		5	N	N		
4		4	N	N		
4		4	N	N		
11		11	Y	Y	Spektrum	Cloxacilin
8		8	N	N		
13		13	Y	N		Ceftriaxone
11		11	Y	N		Ampicilin
9		9	Y	Y	Spektrum	Cefotaxime
8		8	Y	N		Cefotaxime
4		4	N	N		
4		4	N	N		
3		3	N	N		
6		6	Y	Y	Spektrum	Cefotaxime
10		10	Y	N		Ampicilin
13		13	Y	N		Cefotaxime
19		19	Y	N		Ampicilin
6		6	Y	Y	Spektrum	Cefotaxime
14		14	Y	Y	Spektrum	Cefotaxime

6		6	Y	Y	Spektrum	Amoxcilin
13		13	Y	Y	Spektrum	Ampicilin
20		20	Y	Y	Spektrum	Cefotaxime
6		6	Y	Y	Spektrum	Amoxcilin
7		7	Y	Y	Spektrum	Cefotaxime
6		6	Y	N		Ampicilin
5		5	N	N		
6		6	N	N		
6		6	Y	N		Ampicilin
15		15	Y	Y	Spektrum	Cefotaxime
18		18	Y	Y	Spektrum	Ampicilin
6		6	Y	Y	Spektrum	Cefotaxime
4		4	N	N		
7		7	Y	Y	Spektrum	Ceftriaxone
7		7	N	N		
6		6	Y	N		Ampicilin
18		18	Y	Y	Spektrum	Rifampicin
6		6	N	N		
44		44	Y	Y	Spektrum	Cefixime
27		27	Y	N		Ceftriaxone
38		38	Y	N		Cefotaxime
31		31	Y	Y	Spektrum	Ciprofloxacin
33		33	Y	Y	Spektrum	Cefotaxime
3		3	N	N		
6		6	Y	Y	Spektrum	Cefixime
4		4	N	N		
51		51	Y	Y	Spektrum	Cefotaxime
43		43	Y	N		Ampicilin
32		32	Y	Y	Spektrum	Metronidazole
24	24	48	Y	Y	Spektrum	Cefotaxime
70		70	Y	Y	Spektrum	Cefotaxime
8		8	Y	Y	Spektrum	Cefotaxime
40		40	Y	Y	Spektrum	Cefotaxime
18		18	N	N		
24		24	N	N		
20		20	Y	N		Ampicilin
9		9	Y	Y	Spektrum	Ceftriaxone
6		6	Y	Y	Spektrum	Cefotaxime
14		14	Y	N		Cefotaxime
5		5	Y	Y	Spektrum	Cefotaxime
7		7	Y	Y	Spektrum	Ceftriaxone
13		13	Y	Y	Spektrum	Ampicilin
23		23	Y	N		Ampicilin
25		25	Y	N		Cefotaxime
22		22	Y	Y	Spektrum	Cefotaxime
12		12	Y	N		Amoxicilin
9		9	Y	N		Ampicilin
19		19	Y	Y	Spektrum	Cefotaxime

18		18	N	N		
16		16	N	N		
15		15	N	N		
19		19	Y	N		Cefotaxime
13		13	Y	Y	Spektrum	Ceftriaxone
6		6	Y	N		Ampicilin
3		3	N	N		
18		18	N	N		
3		3	Y	N		Chlorampenicol
15		15	Y	Y	Spektrum	Cefotaxime
11		11	Y	Y	Dose	Ampicilin
11		11	Y	Y	Spektrum	Cefotaxime
23		23	Y	Y	Spektrum	Ceftriaxone
19		19	Y	N		Ampicilin
18		18	Y	N		Ampicilin
6		6	Y	Y	Spektrum	Cefotaxime
5		5	N	N		
6		6	Y	N		Amoxicilin
3		3	N	N		
6		6	N	N		
4		4	N	N		
3		3	N	N		
3		3	Y	Y	Spektrum	Azithromisin
5		5	N	N		
9		9	Y	N		Ampicilin
6		6	N	N		
4		4	N	N		
10		10	Y	N		Ampicilin
37		37	Y	N		Ampicilin
37		37	Y	Y	Spektrum	Meropenem
14		14	N	N		
11		11	Y	Y	Spektrum	Amoxicilin
12		12	Y	Y	Spektrum	Ceftriaxone
8		8	N	N		
8		8	N	N		
18		18	N	N		
15		15	Y	N		Ceftriaxone
26	16	42	Y	N		Cefotaxime
10		10	Y	N		Ampicilin
14		14	Y	N		Ampicilin
16		16	Y	N		Ampicilin
7		7	N	N		
19		19	Y	N		Ampicilin
20		20	N	N		
27		27	Y	N		Cefotaxime
18		18	Y	Y	Spektrum	Cefotaxime
11		11	Y	Y	Dose	Ampicilin
6		6	N	N		

4		4	N	N		
10		10	N	N		
10		10	N	N		
5		5	N	N		
10		10	Y	N		Gentamisin
3		3	N	N		
3		3	N	N		
6		6	N	N		
4		4	N	N		
6		6	N	N		
43	3	46	Y	Y	Spektrum	Cefotaxime
13		13	N	N		
4		4	N	N		
10		10	N	N		
3		3	N	N		
3		3	Y	N		Ampicilin
4		4	Y	N		Ampicilin
6		6	N	N		
5		5	Y	N		Ampicilin
47	4	51	Y	Y	Spektrum	Cefotaxime
3		3	Y	N		Ampicilin
4		4	N	N		
9		9	N	N		
3		3	N	N		
10		10	N	N		
10		10	N	N		
6		6	N	N		
6		6	N	N		
5		5	N	N		
21		21	Y	N		Ampicilin
25		25	Y	Y	Spektrum	Cefotaxime
7		7	N	N		
11		11	Y	N		
4		4	Y	N		Ampicilin
4		4	Y	N		Ampicilin
8		8	Y	N		Ampicilin
10		10	N	N		
12		12	Y	Y	Spektrum	Amikasin
22		22	Y	N		Ampicilin
25		25	Y	Y	Spektrum	Ampicilin Sulbact
8		8	Y	Y	Spektrum	Amoxicilin
17		17	Y	Y	Spektrum	Amoxicilin
16		16	Y	Y	Spektrum	Ceftriaxone
5		5	Y	Y	Spektrum	Amoxicilin
5		5	Y	N		Ampicilin
4		4	Y	N		
4		4	Y	N		Ampicilin
6		6	N	N		

21	21	Y	Y	Spektrum	Ceftriaxone
20	20	Y	Y	Spektrum	Ceftriaxone
14	14	Y	Y	Spektrum	Cefotaxime
6	6	N	N		
5	5	N	N		
25	25	Y	N		Cefotaxime
8	8	N	N		
14	14	Y	N		Cefotaxime
11	11	N	N		
15	15	Y	N		Penoxymethyl Penic
4	4	N	N		
4	4	Y	N		Ampicilin
4	4	Y	Y	Spektrum	Cefixime
6	6	Y	Y	Spektrum	Amikasin
14	14	Y	Y	Spektrum	Amoxicilin
8	8	N	N		
20	20	Y	N		Cefotaxime
25	25	Y	Y	Spektrum	Ampicilin
8	8	Y	N		Ampicilin
6	6	Y	N		Eritromisin
6	6	Y	N		Ampicilin
10	10	N	N		
6	6	N	N		
15	15	Y	Y	Spektrum	Cefixime
11	11	Y	N		Ampicilin
4	4	Y	N		Ampicilin
18	18	Y	N		Ampicilin
4	4	Y	N		Ampicilin
6	6	N	N		
14	14	Y	Y	Spektrum	Amoxycilin
4	4	N	N		
19	19	Y	N		Ampicilin
5	5	N	N		
21	21	N	N		
19	19	Y	Y	Spektrum	Ampicilin
3	3	N	N		
4	4	N	N		
3	3	N	N		
3	3	Y	N		Ampicilin
25	25	Y	Y	Spektrum	Ceftriaxone
15	15	Y	Y	Spektrum	Cefotaxime
3	3	N	N		
6	6	N	N		
8	8	Y	Y	Spektrum	Ceftriaxone
14	14	Y	N		Ospen
5	5	Y	N		Ampicilin
5	5	N	N		
4	4	N	N		

8		8	Y	N		Ampicilin
8		8	N	N		
7		7	N	N		
9		9	N	N		
7		7	Y	Y	Spektrum	Ceftriaxone
3		3	N	N		
3		3	Y	N		Ampicilin
3		3	N	N		
20		20	Y	N		Cefotaxime
4		4	Y	N		Cefotaxime
8		8	Y	Y	Spektrum	Cefotaxime
6		6	N	N		
6		6	Y	Y	Spektrum	Cefotaxime
4		4	Y	Y	Spektrum	Cefotaxime
8		8	N	N		
7		7	N	N		
3		3	N	N		
5		5	N	N		
3		3	N	N		
7		7	Y	N		Ampicilin
3		3	N	N		
4		4	Y	Y	Spektrum	Cefotaxime
9		9	Y	Y	Spektrum	Cefotaxime
15		15	Y	N		Ampicillin
5		5	Y	Y	Dose	Ampicillin
6		6	Y	Y	Spektrum	Cefotaxime
7		7	Y	Y	Spektrum	Meropenem
4		4	N	N		
7		7	Y	Y	Spektrum	Cefotaxime
4		4	N	N		
3		3	N	N		
9		9	Y	Y	Spektrum	Ceftriaxone
29		29	Y	N		Ampicilin
14	14	28	Y	N		Cefotaxime
8		8	Y	Y	Spektrum	Cefotaxime
4		4	Y	Y	Spektrum	Cefotaxime
12		12	Y	Y	Spektrum	Ceftazidime
11		11	Y	Y	Spektrum	
4		4	Y	N		Ceftriaxone
22		22	Y	Y	Spektrum	Cefotaxime
4		4	Y	Y	Spektrum	Cefotaxime
4		4	Y	Y	Spektrum	Cefotaxime
3		3	Y	N		
6		6	Y	Y	Spektrum	Azithromisin
29	9	38	Y	Y	Spektrum	Cotrimoxasole
41		41	Y	Y	Spektrum	Cotrimoxasole
6		6	Y	Y	Spektrum	Ceftazidime
30		30	Y	Y	Spektrum	Ceftriaxone

29		29	Y	Y	Spektrum	Ampicilin
21		21	Y	Y	Spektrum	Imipenem
13		13	Y	Y	Spektrum	Cefotaxime
21		21	Y	N		Cefotaxime
18		18	Y	Y	Spektrum	Ceftriaxone
15		15	Y	Y	Spektrum	Cefotaxime
13		13	N	N		
12		12	Y	Y	Spektrum	Cefotaxime
5		5	Y	N		Ampicilin
11		11	Y	N		Ampicilin
4		4	Y	Y	Spektrum	Ampicilin Sulbactam
4		4	Y	Y	Spektrum	Ampicilin Sulbactam
6		6	Y	N		Ampicilin
6		6	N	N		
5		5	N	N		
4		4	N	N		
8		8	Y	Y	Spektrum	Cefixime
7		7	Y	Y	Spektrum	Amoxicilin
7		7	Y	N		INH
5		5	N	N		
5		5	N	N		
4		4	N	N		
3		3	N	N		
3		3	Y	N		Ampicilin
26		26	Y	N		Ampicilin
16		16	Y	N		Cotrimoxasole
15		15	Y	Y	Spektrum	Cefotaxime
5		5	N	N		
37		37	Y	Y	Spektrum	Cefotaxime
37		37	Y	N		Ampicilin
25		25	Y	Y	Spektrum	Cefixime
4	15	19	Y	Y	Spektrum	Cefotaxime
3		3	Y	Y	Spektrum	Ceftriaxone
16		16	Y	N		Cefotaxime
12		12	Y	Y	Spektrum	Cefotaxime
10		10	N	N		
10		10	Y	N		Ampicilin
6		6	N	N		
9		9	N	N		
9		9	Y	N		Ampicilin
7		7	Y	Y	Spektrum	Cefotaxime
9		9	Y	N		Ampicilin
9		9	N	N		
7		7	Y	N		Ampicilin
3		3	N	N		
3		3	Y	N		Ampicilin
3		3	N	N		
3		3	N	N		

4		4	Y	N		Ampicilin
6		6	N	N		
6		6	N	N		
3		3	N	N		
4		4	N	N		
75		75	Y	Y	Dose	Ceftriaxone
35		35	Y	N		Ampicilin
27		27	Y	Y	Spektrum	Ceftazidime
18		18	N	N		
11		11	Y	N		OAT
10		10	Y	N		Ampicilin
10		10	N	N		
9		9	Y	N		Ampicilin
9		9	Y	N		Ampicilin
6		6	N	N		
5		5	N	N		
5		5	N	N		
3		3	N	N		
3		3	N	N		
33		33	Y	Y	Spektrum	Ampicilin sulbactam
18		18	N	N		
8		8	Y	Y	Spektrum	Cefixime
6		6	Y	N		Ampicilin
4		4	N	N		
5		5	N	N		
5		5	N	N		
4		4	N	N		
69		69	Y	Y	Spektrum	Amoxicilin
10	8	18	Y	Y	Spektrum	Meropenem
8		8	Y	Y	Spektrum	Meropenem
5		5	Y	Y	Spektrum	Cefotaxime
9		9	N	N		
5		5	N	N		
5		5	N	N		
29		29	Y	Y	Spektrum	Ceftriaxone
9		9	N	N		
8		8	N	N		
11		11	Y	Y	Spektrum	Cefotaxime
46		46	Y	N		Ampicilin
15		15	Y	N		Ampicilin
13		13	Y	Y	Spektrum	Ceftriaxone
3		3	N	N		
3		3	N	N		
4		4	N	N		
3		3	N	N		
3		3	Y	Y	Spektrum	Cefotaxime
8		8	Y	Y	Spektrum	Ceftriaxone
7		7	N	N		

8	47	55	Y	Y	Dose	Ceftriaxone
22	2	23	Y	N		Ampicilin
7	28	35	N	N		
4	23	27	Y	N		Ceftriaxone
4		4	N	N		
19		19	Y	N		Cefotaxime
10		10	Y	Y	Spektrum	Meropenem
10		10	Y	N		Cefotaxime
7		7	Y	Y	Spektrum	Ceftriaxone
8		8	N	N		
6		6	N	N		
18		18	Y	N		Ampicilin
14		14	Y	N		Ampicilin
12		12	Y	N		Amoxicilin
11		11	Y	N		Ampicilin
10		10	Y	Y	Spektrum	Cefixime
7		7	Y	N		Amoxicilin
5		5	N	N		
4		4	Y	N		Ampicilin
4		4	Y	N		Ampicilin
3		3	Y	N		Ampicilin
25		25	N	N		
15		15	Y	Y	Spektrum	Ciprofloxacin
11		11	N	N		
9		9	N	N		
13		13	Y	Y	Spektrum	Gentamisin
5		5	N	N		
40	3	43	Y	Y	Spektrum	Ampicilin
9	5	14	Y	Y	Spektrum	Ceftriaxone
14	6	20	Y	Y	Spektrum	Meropenem
3		3	N	N		
25		25	Y	Y	Spektrum	Amoxicilin
7		7	Y	Y	Spektrum	Ceftazidime
15		15	Y	Y	Spektrum	Meropenem
12		12	Y	Y	Spektrum	Ceftriaxone
8		8	Y	N		Ampicilin
8		8	N	N		
8		8	Y	N		Ampicilin
6		6	Y	N		Ampicilin
6		6	N	N		
5		5	Y	N		Ampicilin
5		5	Y	N		Ampicilin
24		24	N	N		
20		20	Y	Y	Spektrum	Ceftriaxone
15		15	Y	Y	Spektrum	Ceftraxone
7		7	N	N		
5		5	N	N		
5		5	Y	N		Ampicilin

7		7	Y	N		Cefotaxime
6		6	Y	N		Ampicilin
5		5	N	N		
12		12	N	N		
11		11	Y	Y	Spektrum	Amoxicilin
9		9	Y	N		Ampicilin
5		5	N	N		
6		6	N	N		
3		3	N	N		
23		23	Y	N		Ampicilin
5	8	13	Y	Y	Spektrum	Ampicilin sulbactam
9		9	Y	N		Ampicilin
7		7	Y	Y	Spektrum	Cefotaxime
26		26	N	N		
8		8	N	N		
8		8	N	N		
5		5	N	N		
6		6	Y	N		Ampicilin
5		5	N	N		
4		4	N	N		
4		4	N	N		
4		4	N	N		
8		8	Y	N		Ampicilin
5		5	Y	N		Ampicilin
12		12	Y	Y	Spektrum	Ceftazidime
5		5	N	N		
3		3	N	N		
4		4	N	N		
4		4	Y	Y	Spektrum	Cefotaxime
38		38	Y	Y	Dose	Cefotaxime
32		32	Y	Y	Spektrum	Ceftriaxone
22		22	N	N		
9		9	Y	N		Ampicilin
6		6	Y	Y	Spektrum	Amoxicilin
4		4	N	N		
4		4	N	N		
4		4	Y	N		Ampicilin
12		12	Y	N		Ampicilin
33		33	Y	Y	Spektrum	Cefotaxime
17		17	Y	Y	Spektrum	Meropenem
16		16	Y	Y	Spektrum	Ceftriaxone
16		16	Y	N		Ceftriaxone
11		11	Y	N		Ceftriaxone
7		7	Y	Y	Spektrum	Ampicilin
3		3	Y	N		Ampicilin
27		27	Y	N		Ampicilin
22		22	N	N		
8		8	Y	N		Ampicilin

6		6	Y	N		Ampicilin
3		3	N	N		
4		4	N	N		
3		3	Y	Y	Spektrum	Ceftriaxone
55		55	Y	Y	Spektrum	Ceftriaxone
35		35	Y	N		Ceftriaxone
26		26	Y	Y	Spektrum	Cefotaxime
14		14	N	N		
10		10	Y	N		Ampicilin
10		10	Y	N		Ampicilin
7		7	N	N		
4		4	N	N		
4		4	N	N		
4		4	N	N		
27		27	Y	Y	Spektrum	Ampicilin
24		24	Y	Y	Spektrum	Cefepime
4	12	16	Y	Y	Spektrum	Cefotaxime
13		13	N	N		
10	1	11	Y	Y	Spektrum	Cefotaxime
13		13	N	N		
5		5	N	N		
7		7	Y	N		Ampicilin
4		4	N	N		
4		4	Y	N		Ampicilin
53		53	Y	N		Ampicilin
8		8	Y	Y	Dose	Cefotaxime
8		8	Y	Y	Spektrum	Cefotaxime
6		6	Y	N		Ampicilin
5		5	Y	N		Ampicilin
6		6	Y	N		Ampicilin
4		4	N	N		
5		5	Y	Y	Spektrum	Cefotaxime
6		6	N	N		
23		23	Y	Y	Dose	Ampicilin
11		11	Y	Y	Spektrum	Cefixime
9		9	N	N		
9		9	Y	Y	Spektrum	Amoxiclav
9		9	Y	Y	Spektrum	Amoxicilin
5		5	Y	Y	Spektrum	Ceftriaxone
9		9	Y	N		Cotrimoxasole
4		4	N	N		
5		5	Y	Y	Spektrum	Cefotaxime
33		33	Y	Y	Spektrum	cefotaxime
20		20	Y	Y	Spektrum	Ceftriaxone
19		19	N	N		
9		9	N	N		
8		8	Y	N		Ampicilin
6		6	N	N		

4		4	N	N		
4		4	Y	N		Ampicilin
4		4	Y	N		Ampicilin
26		26	Y	Y	Spektrum	Cefotaxime
19		19	Y	Y	Spektrum	Amoxicilin
5		5	N	N		
5		5	N	N		
7		7	Y	Y	Spektrum	Cefotaxime
3		3	Y	N		Ampicilin
5		5	N	N		
4		4	Y	N		Ampicilin
4		4	N	N		
61		61	Y	Y	Spektrum	Cefotaxime
45		45	N	N		
21		21	Y	Y	Dose	Ampicilin
21		21	Y	N		Ampicilin
21		21	N	N		
24		24	Y	Y	Spektrum	Amoxicilin
39		39	Y	Y	Spektrum	Ceftazidime
24		24	Y	Y	Spektrum	Amoxiclav
23		23	Y	Y	Spektrum	Kotrimoxasole
8		8	Y	Y	Spektrum	Ceftazidime
40		40	Y	N		Cefotaxime
11		11	Y	N		Ampicilin
16		16	Y	N		INH
5		5	Y	N		Ampicilin
26		26	Y	Y	Spektrum	INH
7		7	N	N		
19	13	32	Y	N		Cotrimoxasole
13		13	N	N		
11		11	Y	Y	Spektrum	Ceftriaxone
7		7	N	N		
9		9	Y	Y	Spektrum	Amoxicilin
8		8	Y	N		Ampicilin
4		4	Y	N		Ampicilin
8		8	Y	Y	Spektrum	Ceftriaxone
18		18	Y	Y	Spektrum	Ciprofloxacin
5		5	N	N		
13		13	Y	Y	Dose	Cefotaxime
4		4	Y	N		Ampicilin
4		4	Y	N		Ampicilin
14		14	Y	Y	Spektrum	Ceftriaxone
5		5	Y	N		Ampicilin
4		4	N	N		
4		4	Y	N		Ampicilin
5		5	Y	Y	Spektrum	Cefotaxime
22		22	Y	Y	Dose	Ampicilin
7		7	Y	Y	Dose	Ampicilin

5	5	Y	Y	Spektrum	Cefotaxime
7	7	Y	N		Ampicilin
49	49	N	N		
9	9	N	N		
12	12	Y	Y	Spektrum	Eritromisin
7	7	Y	N		Ampicilin
5	5	Y	N		Ampicilin
3	3	Y	Y	Spektrum	Cefotaxime
44	44	Y	Y	Spektrum	Ampicilin
27	27	Y	N		Ceftazidime
3	3	N	N		
6	6	Y	Y	Spektrum	Cefotaxime
5	5	N	N		
9	9	N	N		
13	13	Y	Y	Spektrum	Ciprofloxacin
63	63	Y	Y	Spektrum	Cefotaxime
8	8	N	N		
4	4	N	N		
9	9	Y	N		Cefotaxime
9	9	Y	Y	Spektrum	Cefotaxime
6	6	Y	N		Ampicilin
16	16	Y	Y	Spektrum	Cefotaxime
6	6	N	N		
6	6	N	N		
4	4	Y	N		Ampicilin
4	4	Y	N		Ampicilin
9	9	Y	Y	Spektrum	Cefotaxime
4	4	N	N		
6	6	N	N		
26	26	Y	Y	Spektrum	Eritromisin
14	14	Y	N		Cefotaxime
5	5	N	N		
33	33	Y	Y	Spektrum	Meropenem
12	12	Y	Y	Spektrum	Cefotaxime
7	7	N	N		
3	3	Y	N		Ampicilin
13	13	Y	Y	Spektrum	Ceftriaxone
7	7	Y	N		Ampicilin
6	6	N	N		
11	11	Y	Y	Spektrum	Gentamisin
3	3	Y	Y	Spektrum	Cefotaxime
8	8	Y	Y	Spektrum	Cloxacilin
15	15	N	N		
33	33	Y	N		Ampicilin
11	11	Y	Y	Spektrum	Ampicilin
5	5	Y	N		Ampicilin
19	19	Y	Y	Spektrum	Ceftazidime
5	5	N	N		

5	5	Y	Y	Spektrum	Ampicilin
13	13	N	N		
9	9	N	N		
13	13	Y	N		Ampicilin
15	15	Y	N		Gentamisin
8	8	Y	Y	Spektrum	Cefotaxime
9	9	Y	Y	Spektrum	Cefotaxime
7	7	N	N		
4	4	Y	N		Ampicilin
4	4	Y	N		Ampicilin
19	19	N	N		
12	12	y	N		Ampicilin
24	24	Y	N		Ampicilin
6	6	Y	Y	Spektrum	Amoxicilin
14	14	Y	N		Phenoxy penicilin
5	5	N	N		
4	4	N	N		
5	5	N	N		
5	5	Y	N		Ampicilin
5	5	N	N		
5	5	Y	Y	Spektrum	Cefotaxime
11	11	Y	Y	Spektrum	Cefixime
11	11	Y	N		Ceftriaxone
4	4	N	N		
7	7	Y	Y	Spektrum	Cefotaxime
9	9	Y	Y	Spektrum	Ceftriaxone
4	4	Y	N		Ampicilin
5	5	Y	Y	Spektrum	Ampicilin
17	17	Y	Y	Spektrum	Ceftriaxone
3	3	N	N		
22	22	Y	Y	Spektrum	Ampicilin
3	3	N	N		
4	4	N	N		
20	20	N	N		
6	6	Y	Y	Spektrum	Ampicilin
8	8	N	N		
7	7	Y	Y	Spektrum	Ceftazidime
3	3	N	N		
13	13	Y	Y	Spektrum	Cefotaxime
20	20	Y	Y	Spektrum	Cefixime
11	11	Y	Y	Spektrum	Cefotaxime
5	5	Y	Y	Spektrum	Gentamisin
25	25	Y	Y	Spektrum	Cefotaxime
25	25	Y	Y	Spektrum	Ampicilin
4	4	Y	N		Ampicilin
4	4	N	N		
6	6	N	N		
24	24	Y	Y	Spektrum	Cefotaxime

15		15	Y	Y	Spektrum	Cefotaxime
13		13	N	N		
7		7	Y	N		Ampicilin
5		5	Y	N		Ampicilin
10		10	N	N		
11		11	Y	Y	Spektrum	Ampicilin
6		6	N	N		
6	4	10	Y	Y	Spektrum	Ampicilin
5		5	Y	Y	Spektrum	Ceftazidime
10		10	Y	Y	Spektrum	Cefotaxime
14		14	N	N		
7		7	N	N		
4		4	Y	N		Ampicilin
4		4	Y	N		Ampicilin
6		6	Y	N		Ampicilin
6		6	N	N		
6		6	Y	Y	Spektrum	Cefotaxime
4		4	N	N		
4		4	Y	N		Ampicilin
4		4	Y	N		Ampicilin
6		6	Y	Y	Spektrum	Amikasin
3		3	N	N		
7		7	Y	N		Ampicilin
3		3	N	N		
5		5	Y	N		Ampicilin
7		7	N	N		
4		4	N	N		
3		3	N	N		
9		9	N	N		
5		5	N	N		
5		5	Y	Y	Dose	Ciprofloxacin
5		5	N	N		
7		7	Y	N		Ampicilin
30	14	44	Y	N		Cefotaxime
7	26	33	Y	Y	Spektrum	Ceftriaxone
17	7	24	Y	Y	Spektrum	Cefotaxime
25		25	N	N		
20		20	Y	N		Metronidazole
19		19	Y	N		Ampicilin
18		18	Y	Y	Spektrum	Amoxiclav
16		16	Y	Y	Spektrum	Eritromisin
14		14	N	N		
8		8	Y	N		Ampicilin
11		11	Y	Y	Dose	Ceftriaxone
11		11	N	N		
13	6	19	Y	N		Ampicilin
7		7	N	N		
7		7	Y	Y	Spektrum	Cefotaxime

6		6	Y	N		Cefixime
7		7	N	N		
35		35	Y	Y	Spektrum	Cefotaxime
3		3	N	N		
3		3	N	N		
7		7	N	N		
9		9	Y	Y	Dose	Ampicilin
9		9	Y	Y	Spektrum	Cefixime
8		8	Y	N		Ampicilin
15		15	N	Y	Spektrum	Ceftazidime
4		4	N	N		
4		4	N	N		
3		3	N	N		
5		5	Y	N		Ampicilin
4		4	N	N		
4		4	N	N		
4		4	Y	N		Ampicilin
8		8	Y	N		Ampicilin
5		5	Y	Y	Spektrum	Cefixime
4		4	N	N		
4		4	N	N		
4		4	N	N		
4		4	Y	N		Ampicilin
11		11	N	N		
5		5	Y	Y	Spektrum	Ampicilin
5		5	N	N		
5		5	Y	Y	Spektrum	Cefotaxime
5		5	N	N		
3		3	N	N		
3		3	N	N		
3		3	Y	N		Ampicilin
3		3	Y	N		Ampicilin
13	40	53	Y	Y	Spektrum	Ciprofloxacin
36		36	Y	Y	Spektrum	Ceftazidime
20		20	N	N		
16		16	Y	N		Ampicilin
3		3	Y	N		Ampicilin
3		3	N	N		
77		77	Y	N		Ceftriaxone
12	25	37	Y	N		
21		21	Y	N		Ceftriaxone
32		32	Y	N		Cefixime
11		11	Y	Y	Spektrum	Ceftriaxone
13		13	Y	Y	Spektrum	Cefotaxime
16		16	Y	Y	Spektrum	Cefotaxime
24		24	Y	N		Ampicilin
14		14	Y	Y	Spektrum	Cefixime
10		10	N	N		

7		7	N	N		
7		7	Y	Y	Spektrum	Cefotaxime
6		6	N	N		
5		5	Y	Y	Spektrum	Cefotaxime
6		6	N	N		
4		4	Y	Y	Dose	Ampicilin
4		4	Y	N		
5		5	Y	N		Ampicilin
4		4	Y	Y	Spektrum	Cefixime
4		4	Y	N		Ampicilin
5		5	Y	N		Ampicilin
42		42	Y	N		Ceftriaxone
23		23	Y	Y	Spektrum	Ampicilin
6		6	N	N		
4		4	N	N		
6		6	N	N		
5		5	Y	N		Amoxicilin
4		4	Y	N		Ampicilin
4		4	Y	N		Amoxicilin
3		3	Y	N		Ampicilin
15		15	Y	Y	Spektrum	Ceftazidime
5		5	Y	Y	Spektrum	Cefixime
4		4	Y	N		Ampicilin
25		25	Y	N		Ampicilin
12		12	Y	N		Cefotaxime
8		8	Y	Y	Dose	Cefotaxime
8		8	Y	Y	Spektrum	Amoxicilin
4		4	Y	N		Ampicilin
4		4	N	N		
9		9	Y	N		Ampicilin
5		5	N	N		
4		4	N	N		
6		6	Y	Y	Spektrum	Cefotaxime
39		39	Y	N		Cotrimoxasole
40		40	Y	N		Cefixime
21		21	N	N		
17		17	N	N		
20		20	Y	N		Cefotaxime
13		13	Y	N		Cefixime
9		9	N	N		
6		6	N	N		
7		7	N	N		
2	4	6	N	N		
4		4	N	N		
2	2	4	N	N		
41	13	54	Y	N		Fluconazole
15		15	Y	Y	Spektrum	Cefotaxime
10		10	N	N		

8		8	Y	N		Ampicilin
4		4	Y	N		Ampicilin
3		3	Y	N		Ampicilin
26		26	N	N		
19		19	Y	N		Cefixime
8		8	Y	Y	Spektrum	Cefotaxime
5		5	N	N		
5		5	Y	N		Ampicilin
3		3	N	N		
24		24	Y	N		Cotrimoxasole
11		11	N	N		
8		8	N	N		
5		5	Y	Y	Spektrum	Azithromisin
7		7	Y	N		Ampicilin
4		4	Y	Y	Spektrum	Azithromisin
4		4	N	N		
5		5	N	N		
3		3	Y	Y	Spektrum	Cloxacilin
24	13	37	Y	Y	Spektrum	Cefixime
9		9	Y	Y	Spektrum	Pentoxycilin
7		7	Y	Y	Spektrum	Amoxicilin
6		6	Y	Y	Spektrum	Ceftriaxone
9		9	N	N		
5		5	N	N		
6		6	Y	N		Ampicilin
9		9	Y	N		Ampicilin
9		9	N	N		
8		8	Y	Y	Spektrum	Amoxiclav
5		5	Y	Y	Spektrum	Cefixime
44		44	Y	N		Cefotaxime
30		30	Y	Y	Spektrum	Amoxicilin
18		18	Y	N		Ampicilin
14		14	Y	Y	Spektrum	Cefotaxime
6		6	Y	Y	Spektrum	Cefotaxime
8		8	Y	Y	Spektrum	Cefotaxime
13		13	N	N		
6		6	N	N		
6		6	N	N		
5		5	Y	N		Ampicilin
4		4	N	N		
5		5	N	N		
18		18	Y	N		Cefixime
18		18	N	N		
11		11	N	N		
7		7	N	N		
8		8	N	N		
4		4	N	N		
33		33	Y	N		Cotrimoxasole

20		20	Y	Y	Spektrum	Ampicilin
13		13	Y	N		Ampicilin
13		13	N	N		
8		8	N	N		
6		6	Y	Y	Spektrum	Cefixime
12		12	Y	N		Ampicilin
9		9	Y	N		Cefotaxime
4		4	Y	Y	Spektrum	Cefotaxime
26		26	Y	Y	Spektrum	Cefotaxime
17		17	Y	Y	Spektrum	Ceftazidime
8		8	Y	Y	Spektrum	Fluconazole
11		11	Y	Y	Spektrum	Cefotaxime
11		11	Y	Y	Spektrum	Amoxicilin
5		5	Y	Y	Spektrum	Ceftriaxone
4		4	Y	N		Ampicilin
50	9	59	Y	N		Cefixime
22		22	Y	Y	Spektrum	Ceftriaxone
26		26	Y	N		Eritromisin
13		13	N	N		
10		10	N	N		
8		8	N	N		
6		6	N	N		
30		30	Y	Y	Spektrum	Ciprofloxacin
22		22	Y	N		Cotrimoxasole
5		5	N	N		
54		54	Y	Y	Spektrum	Ceftriaxone
29		29	Y	N		Cefotaxime
19		19	N	N		
14		14	Y	Y	Spektrum	Ceftriaxone
8		8	Y	N		Ampicilin
10		10	Y	N		Ampicilin
5		5	Y	N		Ampicilin
10		10	N	N		
10		10	N	N		
4		4	Y	Y	Spektrum	Ampicilin
6		6	Y	N		Ampicilin
6		6	Y	N		Ampicilin
4		4	N	N		
16		16	Y	Y	Spektrum	Ampicilin
10		10	Y	Y	Spektrum	Ampicilin Sulbactam
23		23	Y	N		Ampicilin
5		5	N	N		
7		7	Y	Y	Spektrum	Cefotaxime
21		21	N	N		
16		16	N	N		
10		10	Y	Y	Spektrum	cefotaxime
8		8	Y	Y	Spektrum	Cefotaxime
12		12	N	N		

15		15	Y	Y	Spektrum	Ceftazidime
11		11	N	N		
5		5	Y	Y	Spektrum	Ceftazidime
8		8	Y	N		Ampicilin
14		14	N	N		
7		7	Y	N		Ampicilin
13		13	N	N		
7		7	Y	Y	Spektrum	Amikasin
4		4	N	N		
7		7	N	N		
12		12	N	N		
3		3	Y	Y	Spektrum	Cotrimoxasole
3		3	N	N		
6		6	Y	N		Ampicilin
7		7	Y	N		Amoxycilin
7		7	Y	Y	Spektrum	Ceftazidime
5		5	N	N		
6		6	N	N		
6		6	N	N		
8		8	N	N		
7		7	N	N		
5		5	N	N		
3		3	N	N		
4		4	N	N		
4		4	N	N		
3		3	N	N		
21		21	N	N		
10		10	N	N		
6		6	N	N		
6		6	N	N		
3		3	Y	N		Ampicilin
20		20	Y	Y	Spektrum	Cefotaxime
10		10	N	N		
8		8	Y	Y	Spektrum	Ceftriaxone
6		6	Y	Y	Spektrum	Cefixime
14		14	Y	N		Ampicilin
10		10	N	N		
5		5	N	N		
4		4	Y	N		Ampicilin
6		6	N	N		
5	54	59	Y	N		Ampicilin
57		57	Y	N		Ampicilin
34		34	Y	Y	Spektrum	cefotaxime
20		20	Y	Y	Spektrum	cefotaxime
24		24	N	N		
11		11	Y	N		Ampicilin
9		9	Y	N		Ampicilin
8		8	Y	N		Ampicilin

5	5	N	N		
16	16	N	N		
9	9	Y	N		Ampicilin
7	7	Y	Y	Spektrum	Ceftriaoxne
8	8	Y	N		Ampicilin
7	7	Y	Y	Spektrum	Ceftriaxone
7	7	N	N		
5	5	Y	N		Ampicilin
6	6	Y	Y	Spektrum	Ceftriaxone
7	7	Y	N		Cotrimoxasole
4	4	N	N		
5	5	N	N		
5	5	N	N		
23	23	N	N		
19	19	Y	N		Ceftriaxone
14	14	Y	N		Ampicilin
7	7	Y	Y	Spektrum	Metronidazole
9	9	N	N		
9	9	N	N		
9	9	N	N		
7	7	N	N		
6	6	N	N		
4	4	N	N		
13	13	N	N		
10	10	Y	N		Ampicilin
6	6	N	N		
11	11	Y	Y	Spektrum	Cefotaxime
26	26	Y	N		Ampicilin
5	5	N	N		
11	11	N	N		
7	7	Y	N		Ampicilin
9	9	Y	N		Ampicilin
6	6	N	N		
3	3	Y	Y	Spektrum	Cefixime
18	18	Y	Y	Spektrum	Cefotaxime
30	30	Y	Y	Spektrum	Cefotaxime
33	33	N	N		
23	23	N	N		
18	18	N	N		
18	18	N	N		
15	15	Y	Y	Spektrum	Ceftriaxone
9	9	Y	Y	Spektrum	Ceftriaxone
20	20	N	N		
11	11	N	N		
9	9	Y	N		Ampicilin
9	9	N	N		
5	5	N	N		
4	4	Y	N		Cefotaxime

7	7	Y	Y	Spektrum	Cefotaxime
6	6	Y	N		Ampicilin
5	5	N	N		
4	4	N	N		
6	6	Y	Y	Spektrum	Ceftriaxone
15	15	N	N		
11	11	Y	N		Ampicilin
10	10	Y	Y	Spektrum	Meropenem
13	13	Y	N		Cotrimoxasole
10	10	N	N		
12	12	Y	N		Ampicilin
11	11	N	N		
4	4	N	N		
5	5	Y	Y	Spektrum	Cefotaxime
5	5	Y	Y	Spektrum	Cefixime
8	8	Y	N		Ospen
4	4	Y	N		Ampicilin
11	11	N	N		
7	7	Y	N		Ampicilin
8	8	Y	Y	Spektrum	Cefixime
7	7	N	N		
7	7	Y	Y	Spektrum	Cefotaxime
6	6	N	N		
6	6	Y	Y	Spektrum	Cefixime
9	9	N	N		
37	37	Y	Y	Spektrum	Cefotaxime
6	6	N	N		
4	4	N	N		
4	4	N	N		
38	38	Y	N		Cotrimoxasole
38	38	Y	Y	Dose	Ampicilin
6	6	N	N		
21	21	Y	N		Ampicilin
20	20	Y	N		Ampicilin
9	9	Y	Y	Spektrum	Cefixime
16	16	N	N		
15	15	N	N		
7	7	Y	N		Ampicilin
8	8	Y	N		Ampicilin
4	4	N	N		
5	5	N	N		
9	9	N	N		
3	3	Y	N		Amoxicilin
54	54	Y	Y	Spektrum	Cefotaxime
7	7	Y	N		Cefotaxime
15	15	Y	Y	Spektrum	Ceftriaxone
13	13	N	N		
14	14	N	N		

5	5	N	N		
7	7	Y	N		Ampicilin
9	9	Y	N		Ampicilin
9	9	Y	N		Ospen
9	9	Y	N		Ampicilin
6	6	N	N		
9	9	N	N		
8	8	N	N		
10	10	Y	N		Cefixime
4	4	N	N		
4	4	N	N		
3	3	N	N		
3	3	N	N		
3	3	N	N		
5	5	Y	N		Ampicilin
5	5	N	N		
56	56	Y	N		Ampicilin
53	53	Y	N		Ampicilin
13	13	N	N		
5	5	N	N		
4	4	N	N		
4	4	N	N		
21	21	N	N		
21	21	Y	Y	Dose	Cefotaxime
16	16	Y	N		Ceftriaxone
19	19	N	N		
17	17	N	N		
19	19	Y	N		Ampicilin
7	7	N	N		
14	14	Y	Y	Spektrum	Cefixime
7	7	Y	Y	Spektrum	Amoxiclav
7	7	Y	N		Ampicilin
8	8	Y	Y	Spektrum	Ceftazidime
11	11	Y	Y	Spektrum	Cefixime
9	9	Y	Y	Spektrum	Amoxicilin
6	6	N	N		
7	7	N	N		
8	8	N	N		
10	10	N	N		
5	5	N	N		
7	7	N	N		
6	6	N	N		
6	6	N	N		
6	6	N	N		
6	6	Y	Y	Spektrum	Cefixime
23	23	Y	N		Ceftriaxone
10	10	Y	Y	Spektrum	Cotrimoxasole
12	12	N	N		

14		14	Y	N		Cefixime
7		7	N	N		
3		3	N	N		
4		4	Y	N		Ampicilinj
4		4	N	N		
4		4	Y	Y	Spektrum	Cefotaxime
44		44	Y	Y	Spektrum	Ceftazidime
13		13	N	N		
24		24	Y	N		Ampicilin
12		12	N	N		
11		11	N	N		
6		6	Y	Y	Spektrum	Amoxycilin
8		8	Y	N		Ampicilin
6		6	N	N		
10		10	Y	N		Fluconazole
4		4	Y	N		Ampicilin
7		7	N	N		
5		5	N	N		
5		5	N	N		
5		5	N	N		
66	3	69	Y	N		Ampicilin
12	1	13	Y	Y	Spektrum	Cefotaxime
12	1	13	Y	Y	Spektrum	Cefotaxime
3	12	15	Y	N		Cefotaxime
7	6	13	Y	Y	Spektrum	Ampicilin sulbactam
16	6	22	N	N		
3	1	4	Y	Y	Spektrum	Cefotaxime
20		20	Y	N		Ampicilin
8		8	Y	Y	Spektrum	Cefotaxime
8		8	N	N		
11		11	Y	Y	Spektrum	Amoxicilin
8		8	N	N		
8		8	N	N		
21		21	Y	N		Ospen
6	3	9	Y	N		Cefotaxime
13		13	Y	Y	Spektrum	Cefixime
8		8	N	N		
6		6	N	N		
6		6	N	N		
4		4	N	N		
4		4	N	N		
3	2	5	N	N		
4	9	13	Y	Y	Spektrum	Cefotaxime
7	2	9	Y	Y	Spektrum	Cefotaxime
4		4	Y	N		Cefotaxime
10	19	29	Y	Y	Spektrum	Cefotaxime
3	3	6	Y	Y	Spektrum	Cefotaxime
2	1	3	N	N		

3		3	N	N		
12		12	Y	Y	Spektrum	Ceftriaxone
20		20	Y	N		Ampicilin
5	6	11	Y	N		Cefotaxime
5		5	Y	Y	Spektrum	Cefotaxime
4		4	Y	Y	Spektrum	Cefotaxime
7		7	Y	Y	Spektrum	Cefotaxime
4		4	N	N		
7		7	Y	Y	Spektrum	Cefotaxime
5		5	N	N		
3	2	5	N	N		
14		14	Y	N		Clindamisin
17		17	N	N		
24		24	Y	Y	Spektrum	Cefotaxime
25		25	Y	N		Cefotaxime
15		15	Y	Y	Spektrum	Cefotaxime
9		9	N	N		
7		7	N	N		
4		4	Y	N		Ampicilin
7		7	N	N		
6		6	Y	N		
12		12	Y	N		Ampicilin
5		5	Y	Y	Spektrum	Cefixime
5		5	N	N		
5		5	N	N		
5		5	N	N		
7		7	N	N		
5		5	Y	N		Ampicilin
26		26	Y	Y	Spektrum	Cefotaxime
11		11	N	N		
7		7	Y	Y	Spektrum	Cefotaxime
7		7	Y	N		Ampicilin
8		8	Y	N		Ampicilin
6		6	Y	N		Ceftriaxone
12		12	Y	N		Ampicilin
7		7	Y	N		Ampicilin
13		13	Y	N		Ampicilin
19		19	N	N		
5		5	Y	N		Ampicilin
7		7	N	N		
48		48	Y	Y	Spektrum	Cefotaxime
4		4	N	N		
4		4	Y	N		Ampicilin
4		4	Y	N		Ampicilin
8		8	Y	N		Ampicilin
3	4	7	N	N		
4		4	N	N		
11		11	Y	Y	Spektrum	Ceftriaxone

4		4	N	N		
6		6	Y	N		Ampicilin
6		6	N	N		
12		12	N	N		
8		8	Y	N		Ampicilin
14		14	Y	Y	Spektrum	Cefazolin
4		4	N	N		
3		3	N	N		
6		6	N	N		
7		7	N	N		
8		8	Y	N		Ampicilin
6		6	N	N		
4		4	N	N		
5		5	N	N		
6		6	N	N		
8		8	N	N		
31		31	Y	N		Ampicilin
7		7	Y	N		Ampicilin
5		5	Y	N		Ampicilin
4		4	N	N		
6		6	N	N		
21	5	26	Y	Y	Spektrum	Metronidazole
13		13	N	N		
7	15	22	Y	Y	Spektrum	Ceftriaxone
11		11	Y	Y	Spektrum	Ampicilin
10		10	N	N		
5	4	9	Y	N		Cefotaxime
6		6	Y	N		Ampicilin
8		8	Y	N		Ceftriaxone
12		12	Y	Y	Spektrum	Ampicilin
11		11	Y	N		Cefotaxime
5		5	N	N		
4		4	Y	N		Ampicilin
4		4	Y	N		Ampicilin
3		3	N	N		
9		9	N	N		
2	5	7	Y	Y	Spektrum	Ampicilin
6		6	N	N		
5		5	N	N		
3		3	N	N		
7		7	Y	N		Ampicilin
6		6	Y	N		Ampicilin
5		5	N	N		
4		4	N	N		
16		16	Y	Y	Spektrum	Ceftriaxone
7		7	Y	Y	Spektrum	Ampicilin
10		10	Y	Y	Spektrum	Cefotaxime
15		15	N	N		

10	10	Y	N		Ampicilin
7	7	Y	N		Ampicilin
9	9	Y	N		Ampicilin
5	5	Y	N		Ampicilin
6	6	N	N		
6	6	N	N		
7	7	N	N		
8	8	N	N		
5	5	Y	N		Ampicilin
4	4	N	N		
8	8	Y	Y	Spektrum	Ampi sulbactam
10	10	N	N		
11	11	N	N		
30	30	N	N		
31	31	Y	N		Ampicilin
20	20	N	N		
15	15	N	N		
18	18	Y	Y	Spektrum	Ceftazidime
26	26	Y	Y	Spektrum	Ceftriaxone
17	17	Y	N		Ospen
16	16	N	N		
5	5	N	N		
6	6	Y	N		Ampicilin
4	4	Y	N		Ampicilin
4	4	Y	N		Amoxicilin
5	5	Y	N		Cotrimoxasole
3	3	N	N		
4	4	N	N		
9	9	N	N		
9	9	Y	N		Ampicilin
7	7	N	N		
6	6	N	N		
3	3	N	N		
5	5	N	N		
5	5	N	N		
6	6	Y	N		Ampicilin
10	10	Y	N		Ampicilin
9	9	Y	N		Ampicilin
4	4	Y	N		Ampicilin
5	5	N	N		
8	8	N	N		
12	12	N	N		
4	4	N	N		
5	5	N	N		
5	5	N	N		
10	10	N	N		
6	6	N	N		
7	7	N	N		

5	5	N	N		
21	21	Y	N		Gentamisin
16	16	N	N		
17	17	N	N		
11	11	Y	N		Ampicilin
9	9	Y	N		Ampicilin
10	10	N	N		
9	9	Y	Y	Spektrum	Cefotaxime
12	12	Y	Y	Spektrum	Amoxycilin
15	15	Y	N		Ospen
6	6	N	N		
11	11	Y	Y	Spektrum	Eritromisin
7	7	Y	N		Cefotaxime
5	5	Y	N		Cefotaxime
5	5	Y	N		Ampicilin
19	19	Y	N		Ampicilin
13	13	Y	Y	Spektrum	Amoxycilin
4	4	Y	Y	Spektrum	Ceftriaxone
4	4	N	N		
11	11	Y	N		Ampicilin
12	12	N	N		
6	6	N	N		
11	11	Y	Y	Spektrum	Ceftriaxone
8	8	N	N		
5	5	Y	Y	Spektrum	Ampicilin
5	5	N	N		
4	4	N	N		
12	12	Y	N		Ampicilin
4	4	Y	Y	Spektrum	Cefixime
3	3	Y	Y	Spektrum	Cefixime
12	12	Y	Y	Spektrum	Metronidazole
12	12	N	N		
9	9	N	N		
6	6	N	N		
10	10	Y	Y	Spektrum	Ampicilin
11	11	N	N		
15	15	Y	Y	Spektrum	Cefotaxime
10	10	N	N		
4	4	Y	Y	Spektrum	Ampicilin
10	10	N	N		
8	8	N	N		
4	4	Y	N		Ampicilin
7	7	N	N		
35	35	Y	Y	Spektrum	Cefixime
26	26	N	N		
15	15	Y	Y	Spektrum	Ciprofloxacin
12	12	Y	N		Ceftriaxone
25	25	Y	N		Ampicilin

5		5	N	N		
9		9	Y	Y	Spektrum	Ceftazidime
6		6	Y	N		Ampicilin
7		7	Y	Y	Spektrum	Ciprofloxacin
10		10	N	N		
9		9	Y	N		Ampicilin
6		6	Y	N		Ampicilin
10		10	Y	N		Cotrimoxsasole
7		7	Y	N		Ampicilin
7		7	Y	N		Ampicilin
13		13	Y	N		Ampicilin
6		6	Y	N		Cefotaxime
8		8	N	N		
3		3	Y	N		Ampicilin
8		8	Y	Y	Spektrum	Eritromisin
4		4	N	N		
10		10	N	N		
4		4	Y	N		Ampicilin
4		4	Y	N		Ampicilin
4		4	Y	N		Ampicilin
10		10	N	N		
6		6	Y	N		Ampicilin
6		6	N	N		
37		37	N	N		
3		3	N	N		
20	4	24	Y	N		Cefotaxime
8		8	N	N		
13		13	Y	N		Cefixime
15		15	N	N		
8		8	N	N		
8		8	N	N		
9		9	N	N		
6		6	Y	N		Ampicilin
5		5	Y	Y	Spektrum	Cefixime
4		4	Y	N		Ampicilin
6		6	Y	N		Ampicilin
3		3	N	N		
3		3	Y	N		Ampicilin
6		6	N	N		
7		7	N	N		
4		4	N	N		
5		5	N	N		
6		6	N	N		
4		4	N	N		
3		3	N	N		
5		5	N	N		
4		4	Y	N		Ampicilin
4		4	Y	N		Ampicilin

4		4	N	N		
4		4	N	N		
4	3	7	N	N		
25		25	Y	N		Ampicilin
12	10	22	N	N		
4	13	17	Y	N		Cefotaxime
5	2	7	Y	Y	Spektrum	Cefotaxime
7	11	18	Y	N		Ampicilin
3		3	N	N		
10		10	Y	N		Ampicilin
11	6	17	Y	Y	Spektrum	Cefotaxime
7		7	Y	N		Ampicilin
10		10	Y	Y	Spektrum	Cloxacilin
15		15	N	N		
16		16	N	N		
8		8	Y	N		Ampicilin
7		7	Y	N		Ampicilin
6		6	Y	N		Ampicilin
5		5	Y	N		Ampicilin
5		5	Y	N		Ampicilin
13		13	N	N		
8		8	Y	N		Cotrimoxasole
7		7	N	N		
4		4	N	N		
4		4	Y	N		Ampicilin
4		4	Y	N		Ampicilin
4		4	Y	N		Ampicilin
11		11	Y	N		Ampicilin
5		5	Y	N		Ampicilin
4		4	N	N		
6		6	N	N		
6		6	Y	N		Ampicilin
5		5	Y	N		Ampicilin
6		6	N	N		
8		8	N	N		
4		4	Y	N		Ampicilin
4		4	Y	N		Ampicilin
4		4	Y	N		Ampicilin
4		4	Y	N		Ampicilin
4		4	Y	N		Ampicilin
5		5	N	N		
6		6	N	N		
5	22	27	Y	N		Ospen
24		24	Y	N		Cefixime
13		13	Y	Y	Spektrum	Cefotaxime
7	3	10	Y	N		Ampicilin
5	7	12	Y	Y	Spektrum	Cefotaxime
6		6	Y	N		Ampicilin

3	6	9	Y	Y	Spektrum	Cefotaxime
5		5	N	N		
8		8	Y	Y	Spektrum	Cefixime
5		5	Y	N		Ampicilin
8		8	N	N		
4		4	N	N		
4		4	Y	N		Ampicilin
5		5	N	N		
4		4	N	N		
4		4	N	N		
3		3	Y	N		Ampicilin
5		5	N	N		
5		5	Y	N		Ampicilin
33	10	43	Y	Y	Spektrum	Ceftazidime
13	21	34	Y	Y	Spektrum	Meropenem
18		18	Y	N		Ampicilin
13		13	Y	Y	Spektrum	Ceftriaxone
11		11	N	N		
5	7	12	N	N		
4		4	Y	Y	Spektrum	Ceftriaxone
7		7	N	N		
6		6	N	N		
24		24	Y	Y	Dose	Cefotaxime
4		4	Y	N		Ampicilin
4		4	Y	N		Ampicilin
33		33	Y	Y	Spektrum	Cefotaxime
30		30	Y	Y	Spektrum	Ceftazidime
21		21	Y	Y	Spektrum	Meropenem
21	6	27	Y	N		Ampicilin
18		18	N	N		
13		13	N	N		
10		10	Y	Y	Spektrum	Ceftriaxone
18		18	N	N		
13		13	N	N		
10		10	N	N		
12		12	Y	Y	Spektrum	Ampicilin
9		9	Y	Y	Spektrum	Kotrimoxasole
13		13	N	N		
5		5	N	N		
6		6	Y	N		Ampicilin
5		5	Y	N		Ampicilin
7		7	Y	N		Ampicilin
6		6	N	N		
7		7	N	N		
6		6	N	N		
4		4	y	N		Ampicilin
16	9	25	Y	Y	Spektrum	Cefotaxime
5	7	12	N	N		

11	6	17	Y	Y	Spektrum	Ceftriaxone
13		13	N	N		
10		10	Y	Y	Spektrum	Ampicilin
10		10	Y	N		Ampicilin
14		14	Y	Y	Spektrum	Ceftazidime
4		4	Y	N		Ampicilin
5		5	N	N		
3		3	Y	N		Ampicilin
9		9	N	N		
5		5	Y	N		Ampicilin
27		27	Y	Y	Spektrum	Cefotaxime
14	2	16	N	N		
21		21	Y	N		Ampicilin
4	17	21	Y	N		Ceftriaxone
6	15	21	Y	Y	Spektrum	Cefotaxime
28		28	N	N		
9		9	Y	Y	Spektrum	Ceftazidime
8		8	Y	Y	Spektrum	Cefotaxime
21		21	Y	Y	Spektrum	Amoxicilin
29		29	Y	Y	Spektrum	Ceftriaxone
14		14	Y	Y	Spektrum	Ceftazidime
18		18	Y	N		Ceftriaxone
8		8	N	N		
13		13	N	N		
3		3	Y	N		Ampicilin
2	16	16	Y	Y	Spektrum	Cefotaxime
6		6	Y	N		Ampicilin
5		5	Y	N		Ampicilin
8		8	Y	N		Ampicilin
7		7	N	N		
4		4	Y	N		Ampicilin
8		8	N	N		
13		13	Y	N		Gentamisin
6		6	N	N		
5		5	N	N		
3		3	Y	N		Ampicilin
3		3	Y	N		Ampicilin
5		5	Y	N		Ampicilin
5		5	Y	N		Ampicilin
4		4	Y	Y	Spektrum	Cefotaxime
8		8	Y	N		Ampicilin
4		4	Y	N		Ampicilin
4		4	Y	N		Ampicilin
5		5	N	N		
3		3	Y	N		Ampicilin
3		3	N	N		
5		5	Y	N		Ampicilin
49		49	Y	N		Ampicilin

29		29	Y	Y	Spektrum	Ceftriaxone
15		15	Y	N		Ampicilin
15		15	N	N		
6		6	N	N		
5		5	Y	N		Cefotaxime
8		8	Y	Y	Spektrum	Amoxycilin
5		5	N	N		
6		6	Y	N		Ampicilin
10		10	Y	Y	Spektrum	Cotromoxasole
9		9	N	N		
6		6	N	N		
8		8	N	N		
5		5	N	N		
3		3	Y	N		Ampicilin
3		3	Y	N		Ampicilin
5		5	N	N		
5		5	N	N		
5	5	10	N	N		
24	3	27	Y	Y	Spektrum	Cefotaxime
33		33	Y	N		Ampicilin
14	3	17	Y	Y	Spektrum	Ceftriaxone
21		21	Y	Y	Spektrum	Azithromisin
23		23	Y	Y	Spektrum	Ampicilin
13		13	Y	Y	Spektrum	Cefotaxime
7		7	Y	N		Ampicilin
15		15	Y	N		Cefotaxime
10		10	N	N		
9		9	Y	Y	Spektrum	Ceftriaxone
9		9	N	N		
5		5	N	N		
5		5	Y	N		Ampicilin
11		11	Y	Y	Spektrum	Ceftriaxone
3		3	Y	N		Ampicilin
3		3	Y	N		Ampicilin
6		6	N	N		
4		4	N	N		
6		6	N	N		
6		6	N	N		
5		5	Y	Y	Dose	Ampicilin
5		5	Y	Y	Spektrum	Ceftriaxone
3		3	Y	N		Ampicilin
5		5	N	N		
6		6	N	N		
4		4	N	N		
4		4	Y	N		Ampicilin
3		3	N	N		
86		86	Y	N		Gentamisin
29		29	Y	Y	Spektrum	Cefazolin

23		23	Y	N		Ceftriaxone
12		12	Y	N		Ampicilin
13		13	Y	Y	Spektrum	Ceftriaxone
12		12	N	N		
6	4	10	N	N		
11		11	Y	Y	Spektrum	Ceftriaxone
7		7	N	N		
4		4	N	N		
7		7	Y	N		Ampicilin
6		6	Y	N		Ampicilin
4		4	N	N		
5		5	N	N		
6		6	N	N		
9		9	Y	N		Ampicilin
5		5	N	N		
5	7	12	Y	Y	Spektrum	Cefotaxime
4		4	Y	N		Ampicilin
5		5	N	N		
5		5	Y	N		Eritromisin
3		3	Y	N		Ampicilin
3		3	Y	N		Ampicilin
7		7	N	N		
8		8	Y	Y	Spektrum	Cefotaxime
6		6	Y	N		Ampicilin
5		5	Y	N		Ampicilin
5		5	Y	N		Ampicilin
5		5	Y	N		Ampicilin
6		6	N	N		
3		3	Y	Y	Spektrum	Ciprofloxacin
4		4	Y	N		Ampicilin
6		6	Y	N		Ampicilin
6		6	Y	N		Ampicilin
5		5	N	N		
24	18	42	Y	Y	Spektrum	Azitromisin
11		11	N	N		
9	22	31	Y	Y	Spektrum	Ampicilin
43		43	Y	N		Ampicilin
20		20	Y	Y	Spektrum	Ceftriaxone
16		16	N	N		
10		10	Y	Y	Spektrum	Cefotaxime
27		27	Y	Y	Spektrum	Ceftazidime
14		14	Y	Y	Spektrum	Cefixime
21		21	Y	Y	Spektrum	Ceftriaxone
16		16	N	N		
22		22	Y	N		Cefotaxime
8		8	N	N		
12		12	N	N		
4		4	Y	Y	Spektrum	Amoxicilin

9		9	N		N		
16		16	Y		Y	Spektrum	Ceftriaxone
5		5	N		N		
4		4	Y		N		Ampicilin
4		4	Y		N		Ampicilin
4		4	Y		N		Ampicilin
5		5	N		N		
4		4	N		N		
16		16	N		N		
9		9	N		N		
8		8	Y		N		Ampicilin
4		4	N		N		
4	3	7	N		N		
9		9	Y		Y	Spektrum	Cefotaxime
6		6	N		N		
5		5	Y		Y	Spektrum	Ciprofloxacin
8		8	Y		Y	Spektrum	Ampicilin
12		12	N		N		
9		9	Y		N		Ampicilin
5		5	Y		N		Ampicilin
5		5	N		N		
8		8	Y		N		Ampicilin
8		8	N		N		
6		6	Y		N		Cefazolin
5		5	Y		N		Cefazolin
5		5	Y		N		Cefazolin
6		6	N		N		
5		5	N		N		
6		6	N		N		
3		3	N		N		
11	3	14	Y		Y	Spektrum	Cefotaxime
54		54	Y		N		Ospen
12	4	16	Y		Y	Spektrum	Cefotaxime
19		19	Y		N		Ampicilin
23		23	Y		Y	Spektrum	Cefotaxime
3	4	7	N		N		
20		20	N		N		
18		18	N		N		
14		14	Y		N		Ceftriaxone
3	2	5	Y		N		Ampicilin
7		7	Y		N		Ampicilin
9		9	Y		Y	Spektrum	Cefazoline
14		14	Y		Y	Spektrum	Cefotaxime
9		9	N		N		
5		5	N		N		
8		8	Y		N		Cefazoline
3		3	Y		N		Cefazoline
5		5	N		N		

5		5	Y	Y	Dose	Metronidazole
5		5	Y	N		Ampicilin
5		5	Y	N		Cefazoline
4		4	Y	N		Ampicilin
4		4	Y	N		Ampicilin
4		4	N	N		
7		7	N	N		
4		4	Y	N		Cefazoline
5		5	Y	N		Cefazoline
26	31	57	Y	Y	Dose	Ceftriaxone
26		26	Y	Y	Spektrum	Meropenem
11		11	Y	N		Cefazoline
11		11	N	N		
8		8	Y	N		Cefazoline
5		5	N	N		
4		4	N	N		
4		4	Y	N		Cefazoline
8		8	Y	N		Ampicilin
5		5	N	N		
7		7	Y	Y	Spektrum	Meropenem
10		10	Y	Y	Spektrum	Ceftriaxone
3		3	N	N		
19		19	Y	Y	Dose	Cefotaxime
4		4	N	N		
9		9	Y	Y	Spektrum	Cefotaxime
3		3	N	N		
6		6	Y	Y	Dose	Cefotaxime
10		10	Y	Y	Dose	Ceftriaxone
11		11	Y	Y	Spektrum	Cefotaxime
8		8	Y	N		Cefotaxime
20		20	Y	Y	Spektrum	Cefotaxime
12		12	Y	Y	Spektrum	Cefotaxime
20		20	Y	Y	Spektrum	Cefotaxime
5		5	Y	Y	Spektrum	Ceftriaxone
8		8	Y	Y	Spektrum	Ceftriaxone
7		7	Y	Y	Spektrum	Cefotaxime
17		17	Y	Y	Spektrum	Cefotaxime
11		11	Y	Y	Spektrum	Cefotaxime
12		12	Y	Y	Spektrum	Cefotaxime
29		29	Y	Y	Spektrum	Cefotaxime
7		7	Y	N		Ceftriaxone
22		22	Y	N		Ceftriaxone
14		14	Y	Y	Dose	Cefotaxime
22	36	58	Y	Y	Spektrum	Cefotaxime
3		3	N	N		
24		24	Y	Y	Spektrum	Cefotaxime
6		6	Y	N		Ampicilin
5		5	N	N		

7		7	Y	Y	Spektrum	Cefotaxime
11		11	Y	Y	Spektrum	Metronidazole
3		3	N	N		
4		4	N	N		
8		8	Y	Y	Spektrum	Cefotaxime
4		4	Y	Y	Spektrum	Cefotaxime
6		6	Y	Y	Spektrum	Cefotaxime
12		12	Y	Y	Spektrum	Cefotaxime
20		20	Y	Y	Spektrum	Cefotaxime
38		38	Y	Y	Spektrum	Ceftriaxone
4		4	N	N		
4		4	Y	Y	Spektrum	Metronidazole
5		5	Y	Y	Spektrum	Cefotaxime
10		10	Y	N		Cefotaxime
3		3	Y	Y	Spektrum	Cefotaxime
4		4	N	N		
6		6	Y	Y	Spektrum	Ceftriaxone
9		9	Y	Y	Spektrum	Meropenem
5		5	Y	N		Ampicilin
6		6	Y	Y	Spektrum	Ampicilin Sulbactam
8		8	Y	N		Ampicillin
7		7	Y	Y	Spektrum	Ampicilin Sulbactam
11		11	Y	Y	Spektrum	Meropenem
4		4	Y	Y	Spektrum	Ceftriaxone
11		11	Y	Y	Dose	Ceftriaxone
8		8	N	N		
4		4	N	N		
7		7	Y	Y	Spektrum	Cefotaxime
3		3	Y	Y	Dose	Ceftriaxone
4		4	N	N		
19		19	Y	Y	Spektrum	Cefotaxime
8		8	Y	Y	Spektrum	Cefotaxime
3		3	Y	Y	Spektrum	Cefotaxime
10		10	Y	Y	Spektrum	Cefotaxime
20		20	Y	Y	Spektrum	Ceftriaxone
13		13	Y	Y	Spektrum	Cefotaxime
16		16	Y	Y	Spektrum	Cefotaxime
10		10	Y	Y	Spektrum	Cefotaxime
4		4	Y	Y	Spektrum	Ceftriaxone
25		25	Y	N		Ampicilin
7	8	15	Y	Y	Spektrum	Meropenem
3		3	N	N		
6		6	Y	N		Ampicillin
4		4	Y	N		Ampicillin
5		5	Y	Y	Dose	Cotrimoxasole
13		13	Y	N		Ampicillin
20		20	N	N		
3		3	N	N		

29	18	47	Y	N		Ampicillin
27		27	Y	N		Ampicillin
14		14	Y	N		Ampicillin
11		11	N	N		
8		8	Y	N		Cefazolin
6		6	N	N		
6		6	N	N		
5		5	Y	N		Ampicillin
4		4	Y	N		Cefazolin
8		8	Y	N		Cefazolin
7		7	Y	N		Cefazolin
12		12	Y	Y	Spektrum	Meropenem
5		5	Y	Y	Spektrum	Cefotaxime
5		5	Y	Y	Spektrum	Metronidazole
8		8	Y	N		Cefotaxime
6		6	Y	Y	Spektrum	Cefotaxime
5		5	Y	Y	Spektrum	Ceftriaxone
3		3	N	N		
4		4	N	N		
14		14	Y	Y	Spektrum	Cefotaxime
7		7	Y	N		Cefotaxime
6		6	N	N		
14		14	Y	Y	Spektrum	Ceftriaxone
24		24	Y	Y	Spektrum	Cefotaxime
146		146	Y	Y	Spektrum	Cloxacilin
21		21	Y	Y	Spektrum	Cefotaxime
22		22	Y	Y	Spektrum	Ceftazidime
7		7	Y	Y	Spektrum	Ceftriaxone
8		8	Y	N		Ampicilin
22		22	Y	Y	Spektrum	Cefotaxime
27		27	Y	Y	Spektrum	Ceftriaxone
50		50	Y	N		Ceftriaxone
21		21	Y	Y	Spektrum	Cefotaxime
7		7	Y	Y	Spektrum	Ceftriaxone
5		5	Y	N		Ampicilin
4		4	N	N		
36		36	Y	N		Ospen
33		33	Y	N		Ospen
25		25	Y	Y	Spektrum	Ceftriaxone
10		10	Y	Y	Spektrum	Azithromisin
9		9	Y	Y	Spektrum	Cefadroxil
4	1	5	Y	Y	Dose	Cefotaxime
5		5	N	N		
4		4	Y	N		Cefazolin
4		4	Y	N		Cefazolin
15		15	Y	N		Ospen
4		4	Y	N		Cefazolin
3		3	N	N		

6	6	N	N		
31	31	Y	N		Ospen
10	10	N	N		
6	6	Y	N		Cefazolin
9	9	Y	N		Cefazolin
11	11	Y	N		Cefazolin
8	8	Y	N		Cefazolin
13	13	N	N		
8	8	Y	N		Cefazolin
9	9	N	N		
5	5	N	N		
4	4	Y	N		Cefazolin
4	4	Y	N		Cefazolin
3	3	N	N		
5	5	N	N		
6	6	N	N		
6	6	Y	N		Clindamisin
5	5	N	N		
4	4	Y	N		Cefazolin
34	34	Y	Y	Dose	Cefotaxime
14	14	Y	N		Cefixime
17	17	N	N		
13	13	Y	Y	Dose	Cotrimoxasole
17	17	Y	Y	Dose	Cotrimoxasole
7	7	N	N		
17	17	N	N		
9	9	Y	Y	Spektrum	Ceftriaxone
23	23	Y	N		Ampicilin
6	6	N	N		
12	12	Y	N		Ceftriaxone
8	8	Y	N		Cefazoline
6	6	N	N		
7	7	N	N		
8	8	Y	N		Cefazoline
9	9	Y	N		Cefazoline
3	3	N	N		
	20672			511	93,59%
	7	1122	546	35	6,41%
	5	59,52255	48,6631016	546	
	13				

Type of first antibiot	Type of first antibic	Type of first antib	Type of first ant	Type of firs	Type of firs	Type of firs
INH						
Gentamisin	Metronidazole					
Gentamisin						
Gentamisin						
Ciprofloxacin	Clindamisin					
OAT FOC						
Klorampenicol	Ceftriaxone					
Ampicilin						
Gentamisin						
INH	Rifampicin					

Gentamisin				
Amoxicilin	FDC	Etambutol		
Ceftazidime				
Gentamisin				
Ampicilin	Imipenem	Meropenem		
Cefotaxime				
Metronidazole				
Azithromisin				
Gentamisin				
Cefotaxime	INH	Etambutol		
Meropenem	Cloxacilin			
Cefotaxime				
Ciprofloxacin	Amikasin			
Ceftazidime	Metronidazole	Cefixime		
Ceftriaxone				
Metronidazole				
Gentamisin	Cefotaxime			
Ampicilin	Cloxacilin	Clindamisin	Ampicilin Sulba	Meropenem
Ampicilin	Imipenem	Amikasin		
INH	Ciprofloxacin	Ceftazidime	Gentamisin	Meropenem
Cefixime				
Metronidazole	Azithromisin	Amoxiclav	Ceftazidime	
Gentamisin	Ceftriaxone	Azithromisin		
Erytromisin				
Ceftazidime				
Amoxicilin				
Cefotaxime				
Ceftriaxone	Ampicilin sulbact	Gentamisin	Ciprofloxacin	Cefixime
Ampicilin	Gentamisin			
Gentamisin	Cotrimoxasole	Cefotaxime	Amikasin	INH

Ceftazidime	Clindamisin		
Ampicilin			
Gentamisin	Cotrimoxasole		
Gentamisin			
Ceftazidime	Azithromisin	Ampicilin	
Cefixime			
Gentamisin	Cefotaxime	Ceftriaxone	Azithromisin
Gentamisin	Amoxicilin		
Gentamisin	Cefotaxime		
Metronidazole	Ampicilin	Chlorampenicol	
Cefixime			
Ciprofloxacin			
Azhytromisin			
Gentamisin	Amoxicilin		
Gentamisin	Amoxicilin		
Ceftriaxone			
Metronidazole	Ampicilin	Meropenem	Amoxiclav
Ceftazidime	Gentamisin		

Metronidazole	Meropenem				
Metronidazole	Meropenem	Ceftazidime			
Gentamisin	Ceftriaxone	Metronidazole	Meropenem	Ampicilin S	Amikasin
Imipenem					
Gentamisin	Ceftriaxone				
Ceftriaxone					
Cefixime					

Meropenem	Azithromisin	Ciprofloxacin
Azithromisin	Ciprofloxacin	
Cefotaxime	Ciprofloxacin	
Ampicilin		
nethyl Penicilin		
Ampicilin	Amoxicilin	
Cefotaxime	Ceftriaxone	
Amoxcycilin		
Ceftazidime		
Ceftriaxone		
Cefotaxime		
Ampicilin		
Ampicilin		
Cefixime		

Ampicilin	Meropenem	Metronidazole		
Meropenem				
Cefixime				
Gentamisin				
Metronidazole				
Metronidazole	Ciprofloxacin			
Ciprofloxacin	Meropenem			
Chlorampenicol				
Azithromisin				
Cefotaxime	Ceftazidime			
Ceftriaxone	Meropenem			
Amikasin				
Azithromisin				
Ciprofloxacin	Metronidazole			
Cotrimoxasole	Azithromisin			
Cotrimoxasole	Azithromisin			
Cefepime	Ampicilin Sulbacta	Ceftriaxone		
Cefotaxime	Metronidazole	Imipenem	Meropenem	
Cefotaxime	Metronidazole	Imipenem	Meropenem	
Gentamisin	Metronidazole			
Azithromisin	Ceftazidime	Clindamisin	Cloxacilin	Ciprofloxacin

	Ceftriaxone	Imipenem	Azithromisin			
	Gentamisin	Azithromisin	Ceftriaxone	Ampicilin sulbactam		
	Ceftazidime	Metronidazole				
	Ampicilin					
	Gentamisin	Ceftriaxone				
n Sulbactam						
n Sulbactam						
	Gentamisin					
	Ampicilin					
	Gentamisin	Cefotaxime	Ceftriaxone	Eritromisin		
	Cefixime	Ciprofloxacin	Amoxiclav			
	Clindamisin	Azithromisin	Imipenem	Ampicilin Sulba	Vancomisin	
	Gentamisin	Ceftriaxone	Cefotaxime	Clindamisin	Cloxacilin	Ciprofloxacin
	Azithromisin	Ciprofloxacin	Clindamisin	Meropenem	Vancomysi	Amikasin
	Gentamisin					
	Cefixime					
	Eritromisin					
	Eritromisin	Gentamisin				
	Gentamisin					

Gentamisin					
Cefotaxime	Cefixime				
Gentamisin	Cefixime	Ceftriaxone	Azithromisin	Ceftriaxone	
Gentamisin	Cefixime				
Azithromisin	Ciprofloxacin	Meropenem	Gentamisin	Levofloxaci	Clindamisin
Gentamisin					
Ampisilin	Cefotaxime	Gentamisin	Cotrimoxasole	Metronidaz	Meropenem
Clindamisin					
Eritromisin					
Gentamisin	Cefixime				
Metronidazole	Cotrimoxasole				

Metronidazole	Meropenem		
Meropenem			
Imipenem	Cefixime		
Cotrimoxasole	Azithromisin	Cloxacilin	
Gentamisin	Cotrimoxasole	Ciprofloxacin	
Gentamisin	Cefotaxime		
Gentamisin	Ceftriaxone		
Ampicilin	Gentamisin		
Ceftazidime			
Cefotaxime	Ciprofloxacin	Amikasin	Ceftriaxone
Fluconazole			
Fluconazole			
Gentamisin			
Gentamisin			
Azithromisin	Cefixime		

Ceftriaxone

Gentamisin
n sulbactam

Chlorampenicol

Ampicilin	Ceftriaxone	Meropenem.
Cloxacilin	Metronidazole	

Metronidazole	Meropenem	Ciprofloxacin	Fluconazole
Ceftaxime			

Fluconazole
Cloxacilin

Cefotaxime
Cefotaxime

Cefepime	Levofloxacine	Cefixime				
Amoxiclav						
Gentamisin						
Gentamisin	Azithromisin	Ceftriaxone				
Gentamisin	Ceftazidime	Amikasin	Amoxiclav			
Fluconazole						
Gentamisin						
Gentamisin	Cefotaxime	Cloxacilin	Azithromisin	Fluconazol	Ciprofloxac	Amikasin
Metronidazole						
Gentamisin	Ceftriaxone	Ciprofloxacin				
Ampicilin	Gentamisin					
Ciprofloxacin						
Ceftazidime						

Metronidazole						
Ampicilin						
Azithromisin	Ampicilin	Meropenem	Cefepime	Ceftazidime	Micafungin	Amikasin
Clindamisin	Azithromisin	Ceftriaxone	Ciprofloxacin			
Gentamisin	Cefotaxim	Azithromisin				
Metronidazole						
Clindamisin	Ceftazidime					
Cefotaxime						
Metronidazole	Meropenem					
Gentamisin						
Rifampicin	Kotrimoxasole					
Ethambutol	Rifampicin	Kotrimoxasole	Cefotaxime	Ceftazidime	Gentamisin	Ofloxacin
Cefotaxime	Meropenem	Metronidazole				
Cefotaxime						
Ceftazidime	Azithromisin					
Meropenem	Metronidazole					

Gentamisin			
Amikasin	Chlorampenicol salep		
Ciprofloxacin	Cotrimoxasole	Ceftriaxone	
Cefixime			
Cefixime			
Cefixime			
Gentamisin			
Cefixime	Ciprofloxacin		
Cefixime			
Ceftriaxone	Ciprofloxacin		
Metronidazole	Ceftazidime	Cefotaxime	Amoxicilin
Ciprofloxacin			
Azithromisin			
Gentamisin			
Cefotaxime			
Ampicilin	Gentamisin		
Cefotaxime	Metronidazole		
Gentamisin	Azithromisin		

Ceftriaxone			
ceftriaxone	ceftazidime	Cefixime	Spiramycin
Gentamisin			
Getntamisin	Amoxycilin		
Cotrimoxasole	Azithromisin		
Metronidazole			
Metronidazole			
Ampicilin			
Cefixime			
Ceftriaxone			
Metronidazole	Ciprofloxacin		
Gentamisin	Ciprofloxacin		
Clindamisin	Cefepime	Ampicilin sulbact	Ciprofloxacin
Metronidazole	Cefixime		

Metronidazole	Cefixime				
Eritromisin	Amoxicilin				
Gentamisin	Amoxicilin				
Gentamisin					
Fluconazole	Meropenem				
Metronidazole	Gentamisin	Cefotaxime	Amikasin	Ampicilin	Imipenem
Azithromisin	Ceftriaxone	Fluconazole			
Gentamisin	Ceftazidime				
Ceftriaxone					
Cotrimoxasole	Ampicilin	Gentamisin	Azithromisin		
Gentamisin	Azithromisin				

Cotrimoxasole					
Gentamisin	Cefotaxime				
Gentamisin	Cefotaxime	Metronidazole			
Clindamisin	Ceftazidime	Ceftriaxone	Meropenem	Cloxacilin	
Ampicilin					
Cefotaxime					
Meropenem	Gentamisin	Gentamisin	Ampicilin Sulba	Ceftazidime	
Ampicilin	Gentamisin	Azithromisin	Ampicilin sulba	Cefepime	Ciprofloxacin
Cefotaxime	Metronidazole				
Metronidazole	Meropenem				
Metronidazole	Meropenem				
Cloxacilin	Meropenem	Fluconazole			
Gentamisin	Ceftazidime	Ciprofloxacin			

Metronidazole				
Azithromisin	Ceftazidime	Ciprofloxacin	Cotrimoxasole	
Cefixime	Ceftriaxone	Ciprofloxacin		
Ampicilin				
Gentamisin				
Gentamisin	Ceftriaxone	Cefotaxime	Ampicilin	Azithromisin
Gentamisin				
Ceftriaxone	Ceftazidime	Meropenem		
Ceftriaxone	Amikasin	Ceftazidime	Cotrimoxasole	
Ciprofloxacin				
Cefixime	Ceftriaxone	Ceftazidime	Gentamisin	
Ceftazidime	Metronidazole	Meropenem		

Ampicilin	Gentamisin	Ciprofloxacin	Ceftriaxone		
Cotrimoxasole	Ampicilin	Gentamisin	Ceftriaxone	Azithromisi	Ceftazidime
Ceftriaxone					
Meropenem	Azithromisin	Eritromisin	Ceftriaxone		
Cefotaxime					
Gentamisin	Cotrimoxasole	Ceftriaxone			
Gentamisin					
Ampicilin					
Cefotaxime	Metronidazole				

	Ciprofloxacin			
	Metronidazole			
	Amikasin	Ciprofloxacin	Fluconazole	Meropenem
	Meropenem	Ciprofloxacin		
	Ceftriaxone			
	Ceftriaxone			
	Cefotaxime			
	Ampicilin sulbactam	Ceftriaxone		
	Meropenem	Eritromisin		
	Metronidazole	Cotrimoxasole		
	Cefixime			
	Cefotaxime			
n Sulbactam				
	Metronidazole			

Azithromisin	Ceftriaxone			
Amikasin				
Cefotaxime				
Gentamisin				
Ciprofloxacin				
Gentamisin	Amoxycilin			
Gentamisin	Ceftriaxone	Eritromisin	Fluconazole	
Amoxicilin	Cotrimoxasole	Gentamisin	Ciprofloxacin	Cefotaxime
Amikasin	Ceftriaxone	Meropenem	Fluconazole	Vancomisin
Ciprofloxacin	Metronidazole			
Gentamisin	Cefotaxime			

Amikasin				
Cefotaxime				
Metronidazole				
Gentamisin	Cefotaxime	Cefixime	Eritomisin	
Gentamisin	Amoxicilin			
Amikasin				
Gentamisin	Ceftriaxone	Clindamisin	Cefepime	Ciprofloxacin
Eritromisin				
Gentamisin				

Gentamisin						
Cloxacilin	Gentamisin					
Ceftriaxone						
Amoxicilin syr	Cloxacilin					
Ampicilin	Gentamisin					
Gentamisin						
Cefotaxime	Metronidazole	Azithromisin	Meropenem			
Gentamisin	Cefotaxime	Ceftriaxone	Meropenem	Amikasin	Clindamisin	
Ceftriaxone	Eritromisin	Ceftazidime				
Gentamisin						
Gentamisin						
Metronidazole	Meropenem	Gentamisin	Fluconazole	Vancomisin	Cloxacilin	Cefepime

Gentamisin		
Gentamisin	Ceftriaxone	Cefazoline
Gentamisin	Ceftriaxone	Vancomisin
Ampicilin		
Cefixime		
Gentamisin	Cloxacilin	Clindamisin
Cotrimoxasole		
Gentamisin		
Ceftazidime	Fluconazole	
Cloxacilin		

Ciprofloxacin					
Gentamisin	Fluconazole	Meropenem	Ciprofloxacin	Amikasin	Cefepime
Ospen					
Gentamisin	Ceftriaxone	Cefazoline			
Meropenem					
Amikasin	Azithromisin				
Ceftriaxone					
Fluconazole					
Gentamisin					
Eritromisin					
Gentamisin					
Gentamisin					
Meropenem					
Gentamisin	Azithromisin	Ciprofloxacin	Meropenem	Chlorampe	Amikasin

Cefotaxime	Amoxicilin
Gentamisin	Amoxicilin
Meropenem	
Amikasin	
Ceftriaxone	
Ceftriaxone	
Eritromisin	Ceftriaxone
Gentamisin	Amoxicilin
Ceftriaxone	
Gentamisin	
Metronidazole	Meropenem

Ceftazidime	Amikasin	
Cefotaxime	Cloxacilin	Ampi Sulbactam
Cefotaxime	Meropenem	Amikasin
Gentamisin		
Gentamisin		
Fluconazole	Cotrimoxasole	

Gentamisin

Gentamisin	Ceftriaxone	Ceftazidime	Eritromisin	Cloxacilin
Cefazolin	Ampicilin			
Amikasin	Fluconazole			

Gentamisin

Azithromisin

Clindamisin	Eritromisin
Gentamisin	Eritromisin
Ceftriaxone	Fluconazole
Cefixime	
Gentamisin	
Ceftriaxone	
Cefixime	
Cefotaxime	
Ampicilin	
Cefotaxime	Meropenem

Gentamisin
Cefotaxime

Gentamisin

Gentamisin	Cotrimoxasole	Ceftriaxone	Azitromisin	Meropener	Cotrimoxas	Fluconazole
Ampicilin						
Cefixime						
Gentamisin						
Amoxicilin						
Cefotaxime						
Micafungin						
Metronidazole	Ampicilin					

Ampicilin	Gentamisin	Azithromisin	Cefixime	Clindamisin	Meropenen	Ciprofloxac
Micafungin	Cefotaxime	Chlorampenicol				
Gentamisin	Ceftriaxone	Ceftazidime	Cefixime			
Amikasin						
Meropenem	Ampi Sulb	Amikasin				
Cloxacilin						
Metronidazole	Amikasin	Fosfomisin				
Gentamisin	Ceftriaxone					
Amikasin						
Ceftazidime	Gentamisin					
Gentamisin						
Metronidazole						

Cefazoline

Ceftazidime

Chlorampenicole

Cotrimoxasole

Cefazoline

Cefixime

Ampicilin

Cefotaxime Metronidazole Ceftriaxone

Amoxycilin	Ceftriaxone			
Ciprofloxacin				
Gentamisin	Meropenem	Ciprofloxacin	Amikasin	Imipenem
Gentamisin	Ceftriaxone			
Azithromisin	Meropenem	Fluconazole		
Cefepime	Amikasin			
Gentamisin				
Ampicilin				
Ciprofloxacin				
Ceftriaxone				
Ceftriaxone	Ampicilin	Ciprofloxacin	Cefixime	
Amikasin	Imipenem	Meropenem		

Cloxacilin			
Gentamisin	Ceftriaxone		
Gentamisin			
Metronidazole			
Metronidazole			
Ampi Sulbactam	Cefotaxime	Meropenem	Ciprofloxacin
Ceftriaxone			
Gentamisin	Ceftriaxone		
Gentamisin	Metronidazole	Cefixime	Ceftriaxone
Meropenem			

Gentamisin

Ampicilin

Gentamisin

Ciprofloxacin

Cefotaxime	Cefixime	
Ampicilin	Ceftriaxone	Cefixime

Cefotaxime

Ceftazidime

Cefazoline

Amoxicilin				
Cefixime				
Cefotaxime	Amikasin	Netilmicin	Ciprofloxacin	
Ceftriaxone	Ciprofloxacin			
Amoxicilin				
Ampicilin	Azithromisin	Meropenem	Cloxacilin	
Meropenem				
Ampicilin				
Ampicilin sulbactam	Cefepime			
Metronidazole				
Cefepime				
Metronidazole				
Metronidazole				
Metronidazole	Meropenem			
Metronidazole	Meropenem			
Azithromisin				
Azithromisin	Meropenem			
Meropenem	Amikasin			
Metronidazole	Meropenem	Amikasin	Ciprofloxacin	Cloxacilin
Gentamisin				

	Meropenem		
	Metronidazole		
	Metronidazole		
	Metronidazole		
	Metronidazole	Meropenem	
	Amikasin	Ampicilin sulbactar	Meropenem
	Meropenem		
	Chloramphenicol		
	Azithromisin		
	Metronidazole		
	Ceftriaxone		
	Cefotaxime	Meropenem	
n Sulbactam			
	Azithromisin		
	Ceftazidime		
	Ciprofloxacin		
	Metronidazole		
	Metronidazole		
	Cotrimoxasole	Amikasin	
	Meropenem		
	Metronidazole	Ciprofloxacin	
	Gentamisin		
	Ceftriaxone		
	Gentamisin	Ceftriaxone	

Azithromisin				
Ceftriaxone	Clindamisin			
Gentamisin				
Metronodazole	Ciprofloxacin			
Cefotaxime				
Metronidazole				
Azithromisin				
Metronidazole	Meropenem			
Metronidazole	Meropenem	Amikasin		
Meropenem	Azithromisin	Amikasin	Ampisulbactam	Amoxiclav
Azithromisin	Ospen			
Gentamisin				
Metronidazole				
Amikasin	Azithromisin			
Meropenem	Vancomisin			
Ampi Sulbactam	Chlorampenicol			
Gentamisin				
Klorampenicol				

Meropenem	Fluconazole		
Ceftriaxone			
Ceftriaxone	Ciprofloxacin		
Gentamisin	Fluconazole	Ciprofloxacin	Meropenem

Culture tak contamination				Nosocomial Sign and symptoms 1
Culture tak	contaminat	type of con	organism c	New onset of fever1
N				N
N				N
Y				N
N				Y
N				N
N				N
N				N
Y				N
N				N
N				N
Y				N
N				N
N				N
N				N
N				N
N				N
Y				N
N				N
N				N
N				N
Y				Y
N				N
N				N
N				N
N				N
N				N
N				N
N				N
Y				N
N				N
N				N
N				N
N				N
Y				N
N				N
N				N
N				N
N				N
Y				N
N				N
N				
N				
N				

N	
N	
Y	
N	
N	
N	
N	
N	
N	
Y	Y
Y	
Y	
N	
Y	
N	
N	
Y	
N	
Y	
Y	
Y	
Y	
N	
N	
N	
N	
N	Y
Y	
Y	
Y	
Y	
N	
Y	
N	
N	
N	
Y	
N	
N	
N	
N	N
Y	
Y	
Y	Y
Y	N
N	
Y	
N	Y

N	
N	
Y	
N	Y
Y	
N	
Y	
N	
Y	
N	
N	
N	
N	
Y	
Y	
N	
N	
N	
N	
N	
N	
N	
N	
N	
N	
N	
N	N
N	N
Y	Y
N	N
N	N
N	N
N	N
N	N
N	N
Y	Y
Y	Y
N	N
N	N
N	N
N	N
N	Y
N	N
Y	N
N	Y
Y	N
N	N

N	N
N	N
N	N
N	N
N	N
N	N
N	N
N	N
N	N
N	N
Y	N
N	N
N	N
N	N
N	N
N	N
N	N
N	N
N	N
N	N
N	N
N	N
N	N
N	N
N	N
N	N
N	N
N	N
N	N
Y	Y
N	N
N	Y
N	N
N	N
N	Y
N	Y
N	N
Y	N
Y	Y
Y	Y
N	N
N	N
Y	N
N	N
N	N
N	N
N	N
N	N

N	N
N	N
N	N
N	N
N	N
Y	Y
N	N
Y	N
N	N
N	Y
N	N
N	N
N	N
Y	N
N	Y
N	N
N	Y
N	Y
N	N
Y	N
N	N
N	N
N	N
N	N
N	N
N	N
N	Y
N	N
N	N
Y	Y
N	N
N	N
N	N
N	
Y	
N	
N	
N	
N	
Y	
Y	
N	
N	
N	
N	
N	
N	
N	

N	
N	
N	
N	
Y	
N	
N	
N	
Y	
N	
Y	
N	
Y	
N	
N	
N	
N	
N	
N	
N	N
N	N
Y	N
Y	N
Y	Y
Y	N
N	Y
Y	N
N	N
Y	N
N	N
N	N
Y	N
N	Y
Y	Y
Y	N
Y	N
N	Y
Y	N
N	N
Y	Y
Y	N
N	N
N	N
Y	Y
Y	Y
N	N
Y	Y
Y	Y

Y	
Y	
Y	
N	
Y	
N	
N	
Y	
N	
N	
N	
N	
N	
N	
N	
N	
Y	
N	
N	
N	
N	
N	
N	
N	
Y	Y
Y	
N	
N	
Y	Y
Y	N
Y	Y
Y	
N	N
N	
N	
N	
N	
N	
N	
Y	
Y	
Y	
N	
N	
N	
N	
N	
N	

N	
N	
N	
N	
N	
Y	N
Y	N
N	
N	
N	Y
N	
N	
N	
N	
N	
N	
N	
N	
N	
Y	Y
N	
N	
N	
N	
N	
N	
N	
Y	
Y	N
Y	
Y	
N	
N	
N	
Y	N
Y	
N	
N	
Y	
N	
N	
N	
N	
N	
N	
Y	
N	N
N	

Y	
Y	
N	
Y	Y
N	
N	
Y	
N	
N	
N	
N	
Y	
Y	
Y	
Y	
N	
N	
N	
N	
N	
N	
N	
N	
N	
N	
N	
N	
Y	Y
Y	
Y	Y
N	
N	
N	
Y	
Y	
N	
N	
N	
N	
N	
N	N
N	
N	
N	
N	
N	
N	
N	

Y	
N	
N	
N	
N	
N	
N	
N	
N	
N	
N	
N	
N	
N	
N	
N	
N	
N	
N	
N	
N	
N	
N	
N	
Y	
N	
N	
N	
N	
Y	
Y	N
N	Y
N	
N	
N	
N	
N	
N	
Y	
Y	
Y	
Y	
Y	
N	
N	
Y	
N	
N	

N			
N			
N			
N			
Y			
Y			
N			N
Y			
N			
N			
N			
N			
Y			
N			
Y			
Y			
Y			
N			
Y			
N			
N			
N			
N			
N			
Y			Y
Y			N
N			
N			
N			
N			
N			
Y			N
N			
Y			Y
N			
Y	Urine	Enterococc	Y
N			
N			
Y	Sputum	E coli, Staphylococcus haemolyticus	
N			
N			
Y	Cairan Acites		
Y	Blood		
N			
Y	Aspirat trak	Stenotrophomonas maltophilia, Staphylococcus epidermidis	
N			
N			

Y		Feces, Bloo	E coli, Staphylococcus haemolyticus	
N				
N				
Y		Urine, Bloo	Enterobact	Y
N				
N				
N				
Y		ETT	Pseudomonas aeruginosa, Staphylococcus haemolyticus	
N				
N				
N				
N				
Y				Y
N				Y
Y				Y
N				N
N				N
Y	Y		Stapylococ	Y
N				N
N				Y
Y				Y
Y				N
Y				Y
N				Y
N				N
N				N
N				N
N				N
Y				Y
N				N
Y				N
N				N
N				N
N				N
N				N
N				N
Y				N
N				N
Y				N
N				N
N				N
N				N
N				N
N				N
N				N
N				Y
Y				N
N				N

N	N
N	N
N	Y
N	N
N	N
N	N
N	N
N	N
Y	Y
N	Y
N	N
N	N
N	N
N	N
N	Y
Y	N
N	N
N	N
N	N
N	N
N	N
Y	N
N	N
N	N
N	N
N	N
N	N
N	N
N	N
N	N
N	N
N	N
Y	N
N	N
N	N
N	N
N	N
N	N
N	N
N	N
N	N
N	N
N	N
N	Y
N	N
N	N
y	Y
N	N

N	Y
N	N
N	N
Y	N
Y	Y
Y	N
N	N
N	Y
N	N
N	N
N	Y
N	N
N	N
N	N
N	N
N	N
N	N
N	N
N	N
N	N
N	N
Y	N
Y	Y
N	N
N	N
N	N
N	N
N	N
N	Y
N	N
N	Y
N	N
N	N
N	N
N	N
N	N
N	Y
N	N
N	N
Y	N
N	Y
N	N
Y	N
N	N
N	N
N	N
N	N
N	N

N	Y
N	N
N	Y
N	N
N	N
N	Y
N	N
N	N
N	N
N	N
N	N
N	N
Y	Y
N	N
N	N
N	N
N	N
Y	N
N	N
N	N

Y	Y
N	N
N	Y
N	N
N	N
N	Y
N	N
N	N
N	N
N	N
N	N
N	N
N	N
N	N
N	N
N	N
N	N
N	N
N	N
N	N
N	N
N	N
N	N
N	N
N	N
N	N
N	N
N	N
N	N
N	N
N	N
N	N
N	Y
N	N
N	N
N	Y
N	N
N	N
Y	Y
Y	Y
Y	Y
Y	Y
N	Y
Y	N
Y	Y
Y	Y
Y	N
N	Y

N	N
N	N
N	Y
N	N
N	N
N	N
N	N
N	N
N	N
N	N
N	N
Y	Y
Y	Y
N	N
N	N
N	N
N	N
N	N
N	Y
N	N
N	N
N	N
N	N
Y	N
Y	N
Y	N
N	N
N	N
N	N
N	N
N	N
N	N
N	N
Y	Y
Y	Y
Y	Y
N	N
N	Y
Y	Y
N	N
N	N
N	N
N	N
N	N
N	N
Y	Y
Y	Y
N	N

N	N
N	N
N	N
Y	N
N	N
Y	Y
N	N
N	N
N	N
Y	N
N	N
N	N
N	N
N	N
N	N
N	N
N	N
N	N
N	Y
N	N
N	N
Y	N
N	N
N	N
N	N
N	N
N	N
N	N
N	N
N	Y
N	Y
Y	N
Y	N
N	N
N	N
N	N
N	N
N	N
N	N
N	N
N	N
N	Y
N	N
N	N
N	N
N	N
N	N
Y	N

N	N
N	N
N	N
N	N
Y	N
Y	Y
N	N
N	N
Y	Y
Y	N
Y	N
N	N
N	N
N	N
N	N
Y	Y
N	N
N	Y
N	N
N	N
N	N
N	N
Y	Y
Y	N
N	N
Y	Y
Y	N
N	N
N	N
N	N
N	N
N	N
N	N
Y	N
N	N
N	N
N	N
N	N
N	N
Y	N
N	N
N	N
Y	Y
N	N
N	N
N	N
Y	N
N	Y

N	N
N	N
N	N
N	N
N	N
N	N
Y	N
Y	N
N	N
N	Y
N	N
N	N
N	N
N	N
Y	Y
N	N
N	N
N	N
Y	N
Y	Y
Y	N
Y	Y
Y	Y
Y	Y

N Y

N Y

Y	Y
N	N
N	N
Y	Y
N	Y
Y	Y
Y	Y

Y	Y
Y	Y
Y	Y
Y	Y

Y	Y
Y	N
Y	Y

N	N
N	N
N	N
N	N
Y	N
N	N
N	N
N	Y
N	N
N	N
N	N
N	N
N	N
N	N
N	Y
N	N
N	N
N	N
N	N
N	N
N	Y
N	N
N	N
Y	N
Y	Y
Y	N
N	N
Y	N
N	N
Y	N
N	N
N	N
N	N
N	N
N	N
N	N
N	N
N	N
N	N
N	N
N	N
N	N
N	N
N	N
N	N
N	N
Y	N

N	N
N	N
N	Y
N	N
N	N
N	N
N	N
N	N
N	N
N	N
N	Y
Y	N
N	N
N	N
Y	N
N	N
N	N
N	N
N	N
N	N
N	N
N	N
N	Y
N	N
N	N
Y	Y
N	N
N	Y
N	N
N	N
N	N
N	N
N	N
N	N
N	N
N	N
N	N
N	N
Y	N
N	N
N	N
N	N
N	N
N	N
N	N
N	N
N	N
N	N

N	N
N	N
N	N
Y	N
N	Y
Y	N
N	N
Y	Y
N	N
N	N
Y	Y
N	N
Y	N
N	Y
N	N
N	N
N	N
N	N
N	N
N	N
N	N
N	N
N	N
N	N
N	N
N	N
N	N
N	Y
N	N
N	N
N	N
N	N
N	N
N	N
N	N
N	N
N	N
N	N
N	N
N	N
N	Y
N	N
N	Y
N	N

	N	N
Ciprofloxacin		
	Y	Y
	N	N
	Y	Y
	Y	N
	Y	N

Y	N
Y	N
N	Y
N	N
N	N
N	N
N	N
N	N
N	N
N	N
Y	N
Y	Y
Y	N
Y	N
Y	N
N	N
Y	N
Y	N
N	N
Y	Y
Y	N
N	Y
N	N
N	N
N	N
Y	Y
N	N
N	N
N	N
N	N
N	N
N	N
N	Y
Y	N
N	N
N	N
N	N
N	N
N	N
N	N
Y	N
N	N
N	N
Y	N
N	N
N	N
N	N
Y	Y

Y	Y
N	N
N	N
N	N
Y	N
N	N
N	N
N	N
N	N
N	N
N	N
N	N
N	N
N	N
N	N
N	N
N	N
Y	N
Y	Y
Y	N
Y	N
Y	Y
Y	Y
Y	N
N	N
N	N
Y	Y
Y	N
N	N
N	N
N	N
Y	N
N	N
N	N
N	N
N	N
N	N
N	N
Y	N
N	N
N	N
N	N
N	N
N	N
N	N
N	N
Y	Y
Y	Y

N	N
N	N
N	N
N	N
Y	N
N	N
N	N
N	N
N	N
N	N
N	N
N	Y
N	N
N	N
N	N
Y	Y
N	N
N	Y
Y	N
N	N
N	N
N	N
N	N
N	N
N	N
N	N
N	N
N	N
N	N
N	N
N	N
N	N
N	N
Y	Y
N	N
Y	Y
Y	N
N	Y
N	N
N	N
N	Y
N	N
Y	Y
N	N
N	N
N	N
N	N
N	N

N	N
N	Y
N	N
N	N
N	N
N	N
N	N
N	N
N	N
N	N
Y	N
N	N
N	N
N	N
N	N
N	N
N	N
N	N
N	N
N	N
N	N
N	N
N	N
N	N
N	Y
N	N
N	N
N	N
N	N
N	N
Y	N
Y	Y
Y	N
N	N
N	Y
N	N
N	Y
N	Y
Y	Y
N	N
N	Y
Y	N
N	N
N	N
N	N
N	N
N	N
N	N

N	N
N	N
N	N
N	N
N	N
N	N
N	N
N	N
N	N
Y	Y
N	Y
N	N
N	N
N	N
N	N
N	N
N	N
N	Y
N	N
N	Y
N	N
Y	N
Y	N
N	N
Y	N
N	N
Y	Y
N	N
Y	Y
Y	N
Y	Y
N	Y
Y	Y
Y	N
Y	N
N	N
Y	Y
Y	N
N	N
Y	N
Y	N
Y	N
Y	Y
Y	Y
N	N
Y	Y
N	N
N	N

Y	N
Y	N
N	N
N	N
N	N
N	N
Y	N
Y	Y
Y	Y
Y	Y
N	N
N	N
Y	Y
Y	N
N	N
N	N
Y	N
Y	N
N	N
Y	N
N	N
Y	N
N	N
N	N
Y	N
N	N
N	N
N	N
Y	N
N	N
N	N
Y	N
N	N
N	N
Y	N
Y	Y
N	Y
Y	Y
N	N
Y	Y
N	Y
N	N
N	N
N	N
Y	N
Y	Y
N	N
N	N

Y	N
N	N
Y	Y
N	N
N	N
N	N
N	N
N	N
N	N
N	N
N	N
Y	Y
Y	N
N	N
Y	N
Y	N
N	N
N	N
N	N
Y	Y
Y	N
N	N
Y	Y
Y	Y
Y	Y
Y	Y
N	Y
N	N
Y	N
Y	Y
Y	N
Y	Y
Y	N
Y	N
Y	N
N	N

1
1
1
1
2
2
2
2
2
2
2
2
2
2
2
2
2
2
2
2
3
3
3
3
3
3
3
3
3
3
3
3
3
3
3
3
3
3
3
3
3
3
3
3
4
4
4
4

4
4
4
4
4
4
4
4
4
4
4
4
4
5
5
5
5
5
5
5
5
5
5
5
6
6
6
6
6
6
6
6
6
6
6
6
6
6
6
6
6
6
6
7
7
7
7
7

7
7
7
7
7
7
7
7
8
8
8
8
8
8
8
8
9
9
9
9
9
9
10
10
10
10
10
11
11
12
12
13
13
13
13
13
13
14
14
14
14
15
15
15
16
17
19
19

19
20
20
20
21
21
23
23
23
26
26
32
41
43
101
7,0664598

1
1
1
2
2
2
2
2
2
2
2
2
2
2
2
2
2
2
2
2
2
2
2
2
2
2
2
2
2
3
3
3
3
3
3
3
3
3
3
3
3
3
3
3
3
3

3
3
3
3
3
3
3
3
3
3
3
3
3
3
3
3
3
3
3
3
3
3
3
3
3
4
4
4
4
4
4
4
4
4
4
4
4
4
4
4
4
4
4
4
4
4

4
4
4
4
4
4
4
4
4
4
4
4
4
4
4
4
4
4
4
4
4
4
4
4
4
4
4
4
5
5
5
5
5
5
5
5
5
5
5
5
5
5
5
5
5
5

5
5
5
5
5
5
5
5
5
5
5
5
5
5
5
6
6
6
6
6
6
6
6
6
6
6
6
6
6
6
6
6
6
6
6
6
6
6
6
6
6
6
6
6
6
6

6
6
6
7
7
7
7
7
7
7
7
7
7
7
7
7
7
7
7
7
7
7
7
7
7
7
7
8
8
8
8
8
8
8
8
8
8
8
8
8
8
8
8
8
8
8

9
9
9
9
9
9
9
9
9
9
9
9
9
9
10
10
10
10
10
10
10
11
11
11
11
11
11
11
11
11
11
11
11
11
11
11
11
11
11
12
12
12
12
12
12
12

13
13
13
13
13
13
13
14
14
14
14
14
14
15
16
16
16
17
17
18
18
18
19
19
19
20
21
23
24
24
24
25
30
30
31
5,058365

Y	Y	Dose	Ampicilin
Y	Y	Dose	Ampicilin
Y	Y	Dose	Ampicilin
Y	Y	Dose	Ampicillin
Y	Y	Dose	Ceftriaxone
Y	Y	Dose	Ceftriaxone
Y	Y	Dose	Cefotaxime
Y	Y	Dose	Cefotaxime
Y	Y	Dose	Ampicilin
Y	Y	Dose	Ampicilin
Y	Y	Dose	Cefotaxime
Y	Y	Dose	Ampicilin
Y	Y	Dose	Ampicilin
Y	Y	Dose	Ciprofloxacin
Y	Y	Dose	Ceftriaxone
Y	Y	Dose	Ampicilin
Y	Y	Dose	Ampicilin
Y	Y	Dose	Cefotaxime
Y	Y	Dose	Ampicilin
Y	Y	Dose	Cefotaxime
Y	Y	Dose	Cefotaxime
Y	Y	Dose	Ampicilin
Y	Y	Dose	Metronidazole
Y	Y	Dose	Ceftriaxone
Y	Y	Dose	Cefotaxime
Y	Y	Dose	Cefotaxime
Y	Y	Dose	Ceftriaxone
Y	Y	Dose	Cefotaxime
Y	Y	Dose	Ceftriaxone
Y	Y	Dose	Ceftriaxone
Y	Y	Dose	Cotrimoxasole
Y	Y	Dose	Cefotaxime
Y	Y	Dose	Cefotaxime
Y	Y	Dose	Cotrimoxasole
Y	Y	Dose	Cotrimoxasole
Y	Y	Spektrum	Cefotaxime
Y	Y	Spektrum	Amoxicilin
Y	Y	Spektrum	Ceftriaxone
Y	Y	Spektrum	Cefotaxime
Y	Y	Spektrum	Ceftriaxone
Y	Y	Spektrum	Cefotaxime
Y	Y	Spektrum	Cefixime
Y	Y	Spektrum	Cefotaxime
Y	Y	Spektrum	Ampicilin
Y	Y	Spektrum	Cloxacilin
Y	Y	Spektrum	Cefotaxime

Y	Y	Spektrum	Cefotaxime
Y	Y	Spektrum	Cefotaxime
Y	Y	Spektrum	Cefotaxime
Y	Y	Spektrum	Amoxcilin
Y	Y	Spektrum	Ampicilin
Y	Y	Spektrum	Cefotaxime
Y	Y	Spektrum	Amoxcilin
Y	Y	Spektrum	Cefotaxime
Y	Y	Spektrum	Cefotaxime
Y	Y	Spektrum	Ampicilin
Y	Y	Spektrum	Cefotaxime
Y	Y	Spektrum	Ceftriaxone
Y	Y	Spektrum	Rifampicin
Y	Y	Spektrum	Cefixime
Y	Y	Spektrum	Ciprofloxacin
Y	Y	Spektrum	Cefotaxime
Y	Y	Spektrum	Cefixime
Y	Y	Spektrum	Cefotaxime
Y	Y	Spektrum	Metronidazole
Y	Y	Spektrum	Cefotaxime
Y	Y	Spektrum	Cefotaxime
Y	Y	Spektrum	Cefotaxime
Y	Y	Spektrum	Cefotaxime
Y	Y	Spektrum	Ceftriaxone
Y	Y	Spektrum	Cefotaxime
Y	Y	Spektrum	Cefotaxime
Y	Y	Spektrum	Ceftriaxone
Y	Y	Spektrum	Ampicilin
Y	Y	Spektrum	Cefotaxime
Y	Y	Spektrum	Cefotaxime
Y	Y	Spektrum	Ceftriaxone
Y	Y	Spektrum	Cefotaxime
Y	Y	Spektrum	Cefotaxime
Y	Y	Spektrum	Ceftriaxone
Y	Y	Spektrum	Cefotaxime
Y	Y	Spektrum	Azithromisin
Y	Y	Spektrum	Meropenem
Y	Y	Spektrum	Amoxicilin
Y	Y	Spektrum	Ceftriaxone
Y	Y	Spektrum	Cefotaxime
Y	Y	Spektrum	Cefotaxime
Y	Y	Spektrum	Cefotaxime
Y	Y	Spektrum	Cefotaxime
Y	Y	Spektrum	Amikasin
Y	Y	Spektrum	Ampicilin Sulbact
Y	Y	Spektrum	Amoxicilin

Y	Y	Spektrum	Amoxicilin
Y	Y	Spektrum	Ceftriaxone
Y	Y	Spektrum	Amoxicilin
Y	Y	Spektrum	Ceftriaxone
Y	Y	Spektrum	Ceftriaxone
Y	Y	Spektrum	Cefotaxime
Y	Y	Spektrum	Cefixime
Y	Y	Spektrum	Amikasin
Y	Y	Spektrum	Amoxicilin
Y	Y	Spektrum	Ampicilin
Y	Y	Spektrum	Cefixime
Y	Y	Spektrum	Amoxycilin
Y	Y	Spektrum	Ampicilin
Y	Y	Spektrum	Ceftriaxone
Y	Y	Spektrum	Cefotaxime
Y	Y	Spektrum	Ceftriaxone
Y	Y	Spektrum	Ceftriaxone
Y	Y	Spektrum	Cefotaxime
Y	Y	Spektrum	Cefotaxime
Y	Y	Spektrum	Cefotaxime
Y	Y	Spektrum	Cefotaxime
Y	Y	Spektrum	Cefotaxime
Y	Y	Spektrum	Cefotaxime
Y	Y	Spektrum	Meropenem
Y	Y	Spektrum	Cefotaxime
Y	Y	Spektrum	Ceftriaxone
Y	Y	Spektrum	Ceftazidime
Y	Y	Spektrum	
Y	Y	Spektrum	Cefotaxime
Y	Y	Spektrum	Cefotaxime
Y	Y	Spektrum	Cefotaxime
Y	Y	Spektrum	Azithromisin
Y	Y	Spektrum	Cotrimoxasole
Y	Y	Spektrum	Cotrimoxasole
Y	Y	Spektrum	Ceftazidime
Y	Y	Spektrum	Ceftriaxone
Y	Y	Spektrum	Ampicilin
Y	Y	Spektrum	Imipenem
Y	Y	Spektrum	Cefotaxime
Y	Y	Spektrum	Ceftriaxone
Y	Y	Spektrum	Cefotaxime
Y	Y	Spektrum	Cefotaxime
Y	Y	Spektrum	Ampicilin Sulbactam
Y	Y	Spektrum	Ampicilin Sulbactam
Y	Y	Spektrum	Cefixime
Y	Y	Spektrum	Amoxicilin

Y	Y	Spektrum	Cefotaxime
Y	Y	Spektrum	Cefotaxime
Y	Y	Spektrum	Cefixime
Y	Y	Spektrum	Cefotaxime
Y	Y	Spektrum	Ceftriaxone
Y	Y	Spektrum	Cefotaxime
Y	Y	Spektrum	Cefotaxime
Y	Y	Spektrum	Ceftazidime
Y	Y	Spektrum	Ampicilin sulbactam
Y	Y	Spektrum	Cefixime
Y	Y	Spektrum	Amoxicilin
Y	Y	Spektrum	Meropenem
Y	Y	Spektrum	Meropenem
Y	Y	Spektrum	Cefotaxime
Y	Y	Spektrum	Ceftriaxone
Y	Y	Spektrum	Cefotaxime
Y	Y	Spektrum	Ceftriaxone
Y	Y	Spektrum	Cefotaxime
Y	Y	Spektrum	Ceftriaxone
Y	Y	Spektrum	Meropenem
Y	Y	Spektrum	Ceftriaxone
Y	Y	Spektrum	Cefixime
Y	Y	Spektrum	Ciprofloxacin
Y	Y	Spektrum	Gentamisin
Y	Y	Spektrum	Ampicilin
Y	Y	Spektrum	Ceftriaxone
Y	Y	Spektrum	Meropenem
Y	Y	Spektrum	Amoxicilin
Y	Y	Spektrum	Ceftazidime
Y	Y	Spektrum	Meropenem
Y	Y	Spektrum	Ceftriaxone
Y	Y	Spektrum	Ceftriaxone
Y	Y	Spektrum	Ceftraxone
Y	Y	Spektrum	Amoxicilin
Y	Y	Spektrum	Ampicilin sulbactam
Y	Y	Spektrum	Cefotaxime
Y	Y	Spektrum	Ceftazidime
Y	Y	Spektrum	Cefotaxime
Y	Y	Spektrum	Ceftriaxone
Y	Y	Spektrum	Amoxicilin
Y	Y	Spektrum	Cefotaxime
Y	Y	Spektrum	Meropenem
Y	Y	Spektrum	Ceftriaxone
Y	Y	Spektrum	Ampicilin
Y	Y	Spektrum	Ceftriaxone
Y	Y	Spektrum	Ceftriaxone

Y	Y	Spektrum	Cefotaxime
Y	Y	Spektrum	Ampicilin
Y	Y	Spektrum	Cefepime
Y	Y	Spektrum	Cefotaxime
Y	Y	Spektrum	Cefotaxime
Y	Y	Spektrum	Cefotaxime
Y	Y	Spektrum	Cefotaxime
Y	Y	Spektrum	Cefixime
Y	Y	Spektrum	Amoxiclav
Y	Y	Spektrum	Amoxicilin
Y	Y	Spektrum	Ceftriaxone
Y	Y	Spektrum	Cefotaxime
Y	Y	Spektrum	cefotaxime
Y	Y	Spektrum	Ceftriaxone
Y	Y	Spektrum	Cefotaxime
Y	Y	Spektrum	Amoxicilin
Y	Y	Spektrum	Cefotaxime
Y	Y	Spektrum	Cefotaxime
Y	Y	Spektrum	Amoxicilin
Y	Y	Spektrum	Ceftazidime
Y	Y	Spektrum	Amoxiclav
Y	Y	Spektrum	Kotrimoxasole
Y	Y	Spektrum	Ceftazidime
Y	Y	Spektrum	INH
Y	Y	Spektrum	Ceftriaxone
Y	Y	Spektrum	Amoxicilin
Y	Y	Spektrum	Ceftriaxone
Y	Y	Spektrum	Ciprofloxacin
Y	Y	Spektrum	Ceftriaxone
Y	Y	Spektrum	Cefotaxime
Y	Y	Spektrum	Cefotaxime
Y	Y	Spektrum	Eritromisin
Y	Y	Spektrum	Cefotaxime
Y	Y	Spektrum	Ampicilin
Y	Y	Spektrum	Cefotaxime
Y	Y	Spektrum	Ciprofloxacin
Y	Y	Spektrum	Cefotaxime
Y	Y	Spektrum	Cefotaxime
Y	Y	Spektrum	Cefotaxime
Y	Y	Spektrum	Cefotaxime
Y	Y	Spektrum	Eritromisin
Y	Y	Spektrum	Meropenem
Y	Y	Spektrum	Cefotaxime
Y	Y	Spektrum	Ceftriaxone
Y	Y	Spektrum	Gentamisin
Y	Y	Spektrum	Cefotaxime

Y	Y	Spektrum	Cloxacilin
Y	Y	Spektrum	Ampicilin
Y	Y	Spektrum	Ceftazidime
Y	Y	Spektrum	Ampicilin
Y	Y	Spektrum	Cefotaxime
Y	Y	Spektrum	Cefotaxime
Y	Y	Spektrum	Amoxicilin
Y	Y	Spektrum	Cefotaxime
Y	Y	Spektrum	Cefixime
Y	Y	Spektrum	Cefotaxime
Y	Y	Spektrum	Ceftriaxone
Y	Y	Spektrum	Ampicilin
Y	Y	Spektrum	Ceftriaxone
Y	Y	Spektrum	Ampicilin
Y	Y	Spektrum	Ampicilin
Y	Y	Spektrum	Ceftazidime
Y	Y	Spektrum	Cefotaxime
Y	Y	Spektrum	Cefixime
Y	Y	Spektrum	Cefotaxime
Y	Y	Spektrum	Gentamisin
Y	Y	Spektrum	Cefotaxime
Y	Y	Spektrum	Ampicilin
Y	Y	Spektrum	Cefotaxime
Y	Y	Spektrum	Cefotaxime
Y	Y	Spektrum	Ampicilin
Y	Y	Spektrum	Ampicilin
Y	Y	Spektrum	Ceftazidime
Y	Y	Spektrum	Cefotaxime
Y	Y	Spektrum	Cefotaxime
Y	Y	Spektrum	Amikasin
Y	Y	Spektrum	Ceftriaxone
Y	Y	Spektrum	Cefotaxime
Y	Y	Spektrum	Amoxiclav
Y	Y	Spektrum	Eritromisin
Y	Y	Spektrum	Cefotaxime
Y	Y	Spektrum	Cefotaxime
Y	Y	Spektrum	Cefixime
Y	Y	Spektrum	Cefixime
Y	Y	Spektrum	Ampicilin
Y	Y	Spektrum	Cefotaxime
Y	Y	Spektrum	Ciprofloxacin
Y	Y	Spektrum	Ceftazidime
Y	Y	Spektrum	Ceftriaxone
Y	Y	Spektrum	Cefotaxime
Y	Y	Spektrum	Cefotaxime
Y	Y	Spektrum	Cefixime

Y	Y	Spektrum	Cefotaxime
Y	Y	Spektrum	Cefotaxime
Y	Y	Spektrum	Cefixime
Y	Y	Spektrum	Ampicilin
Y	Y	Spektrum	Ceftazidime
Y	Y	Spektrum	Cefixime
Y	Y	Spektrum	Amoxicilin
Y	Y	Spektrum	Cefotaxime
Y	Y	Spektrum	Cefotaxime
Y	Y	Spektrum	Cefotaxime
Y	Y	Spektrum	Azithromisin
Y	Y	Spektrum	Azithromisin
Y	Y	Spektrum	Cloxacilin
Y	Y	Spektrum	Cefixime
Y	Y	Spektrum	Pentoxycilin
Y	Y	Spektrum	Amoxicilin
Y	Y	Spektrum	Ceftriaxone
Y	Y	Spektrum	Amoxiclav
Y	Y	Spektrum	Cefixime
Y	Y	Spektrum	Amoxicilin
Y	Y	Spektrum	Cefotaxime
Y	Y	Spektrum	Cefotaxime
Y	Y	Spektrum	Cefotaxime
Y	Y	Spektrum	Ampicilin
Y	Y	Spektrum	Cefixime
Y	Y	Spektrum	Cefotaxime
Y	Y	Spektrum	Cefotaxime
Y	Y	Spektrum	Ceftazidime
Y	Y	Spektrum	Fluconazole
Y	Y	Spektrum	Cefotaxime
Y	Y	Spektrum	Amoxicilin
Y	Y	Spektrum	Ceftriaxone
Y	Y	Spektrum	Ceftriaxone
Y	Y	Spektrum	Ciprofloxacin
Y	Y	Spektrum	Ceftriaxone
Y	Y	Spektrum	Ceftriaxone
Y	Y	Spektrum	Ampicilin
Y	Y	Spektrum	Ampicilin
Y	Y	Spektrum	Ampicilin Sulbactam
Y	Y	Spektrum	Cefotaxime
Y	Y	Spektrum	cefotaxime
Y	Y	Spektrum	Cefotaxime
Y	Y	Spektrum	Ceftazidime
Y	Y	Spektrum	Ceftazidime
Y	Y	Spektrum	Amikasin
Y	Y	Spektrum	Cotrimoxasole

Y	Y	Spektrum	Ceftazidime
Y	Y	Spektrum	Cefotaxime
Y	Y	Spektrum	Ceftriaxone
Y	Y	Spektrum	Cefixime
Y	Y	Spektrum	cefotaxime
Y	Y	Spektrum	cefotaxime
Y	Y	Spektrum	Ceftriaoxne
Y	Y	Spektrum	Ceftriaxone
Y	Y	Spektrum	Ceftriaxone
Y	Y	Spektrum	Metronidazole
Y	Y	Spektrum	Cefotaxime
Y	Y	Spektrum	Cefixime
Y	Y	Spektrum	Cefotaxime
Y	Y	Spektrum	Cefotaxime
Y	Y	Spektrum	Ceftriaxone
Y	Y	Spektrum	Ceftriaxone
Y	Y	Spektrum	Cefotaxime
Y	Y	Spektrum	Ceftriaxone
Y	Y	Spektrum	Meropenem
Y	Y	Spektrum	Cefotaxime
Y	Y	Spektrum	Cefixime
Y	Y	Spektrum	Cefixime
Y	Y	Spektrum	Cefotaxime
Y	Y	Spektrum	Cefixime
Y	Y	Spektrum	Cefotaxime
Y	Y	Spektrum	Cefixime
Y	Y	Spektrum	Cefotaxime
Y	Y	Spektrum	Ceftriaxone
Y	Y	Spektrum	Cefixime
Y	Y	Spektrum	Amoxiclav
Y	Y	Spektrum	Ceftazidime
Y	Y	Spektrum	Cefixime
Y	Y	Spektrum	Amoxicilin
Y	Y	Spektrum	Cefixime
Y	Y	Spektrum	Cotrimoxasole
Y	Y	Spektrum	Cefotaxime
Y	Y	Spektrum	Ceftazidime
Y	Y	Spektrum	Amoxycilin
Y	Y	Spektrum	Cefotaxime
Y	Y	Spektrum	Cefotaxime
Y	Y	Spektrum	Ampicilin sulbactam
Y	Y	Spektrum	Cefotaxime
Y	Y	Spektrum	Cefotaxime
Y	Y	Spektrum	Amoxicilin
Y	Y	Spektrum	Cefixime
Y	Y	Spektrum	Cefotaxime

Y	Y	Spektrum	Cefotaxime
Y	Y	Spektrum	Cefotaxime
Y	Y	Spektrum	Cefotaxime
Y	Y	Spektrum	Ceftriaxone
Y	Y	Spektrum	Cefotaxime
Y	Y	Spektrum	Cefotaxime
Y	Y	Spektrum	Cefotaxime
Y	Y	Spektrum	Cefotaxime
Y	Y	Spektrum	Cefotaxime
Y	Y	Spektrum	Cefotaxime
Y	Y	Spektrum	Cefixime
Y	Y	Spektrum	Cefotaxime
Y	Y	Spektrum	Cefotaxime
Y	Y	Spektrum	Cefotaxime
Y	Y	Spektrum	Ceftriaxone
Y	Y	Spektrum	Cefazolin
Y	Y	Spektrum	Metronidazole
Y	Y	Spektrum	Ceftriaxone
Y	Y	Spektrum	Ampicilin
Y	Y	Spektrum	Ampicilin
Y	Y	Spektrum	Ampicilin
Y	Y	Spektrum	Ceftriaxone
Y	Y	Spektrum	Ampicilin
Y	Y	Spektrum	Cefotaxime
Y	Y	Spektrum	Ampi sulbactam
Y	Y	Spektrum	Ceftazidime
Y	Y	Spektrum	Ceftriaxone
Y	Y	Spektrum	Cefotaxime
Y	Y	Spektrum	Amoxycilin
Y	Y	Spektrum	Eritromisin
Y	Y	Spektrum	Amoxycilin
Y	Y	Spektrum	Ceftriaxone
Y	Y	Spektrum	Ceftriaxone
Y	Y	Spektrum	Ampicilin
Y	Y	Spektrum	Cefixime
Y	Y	Spektrum	Cefixime
Y	Y	Spektrum	Metronidazole
Y	Y	Spektrum	Ampicilin
Y	Y	Spektrum	Cefotaxime
Y	Y	Spektrum	Ampicilin
Y	Y	Spektrum	Cefixime
Y	Y	Spektrum	Ciprofloxacin
Y	Y	Spektrum	Ceftazidime
Y	Y	Spektrum	Ciprofloxacin
Y	Y	Spektrum	Eritromisin
Y	Y	Spektrum	Cefixime

Y	Y	Spektrum	Cefotaxime
Y	Y	Spektrum	Cefotaxime
Y	N	Spektrum	Cloxacilin
Y	Y	Spektrum	Cefotaxime
Y	Y	Spektrum	Cefotaxime
Y	Y	Spektrum	Cefotaxime
Y	N	Spektrum	Cefixime
Y	Y	Spektrum	Ceftazidime
Y	Y	Spektrum	Meropenem
Y	Y	Spektrum	Ceftriaxone
Y	Y	Spektrum	Ceftriaxone
Y	Y	Spektrum	Cefotaxime
Y	Y	Spektrum	Ceftazidime
Y	Y	Spektrum	Meropenem
Y	Y	Spektrum	Ceftriaxone
Y	Y	Spektrum	Ampicilin
Y	Y	Spektrum	Kotrimoxasole
Y	Y	Spektrum	Cefotaxime
Y	Y	Spektrum	Ceftriaxone
Y	Y	Spektrum	Ampicilin
Y	Y	Spektrum	Ceftazidime
Y	Y	Spektrum	Cefotaxime
Y	Y	Spektrum	Cefotaxime
Y	Y	Spektrum	Ceftazidime
Y	Y	Spektrum	Cefotaxime
Y	Y	Spektrum	Amoxicilin
Y	Y	Spektrum	Ceftriaxone
Y	Y	Spektrum	Ceftazidime
Y	Y	Spektrum	Cefotaxime
Y	Y	Spektrum	Cefotaxime
Y	Y	Spektrum	Ceftriaxone
Y	Y	Spektrum	Amoxycilin
Y	Y	Spektrum	Cotromoxasole
Y	Y	Spektrum	Cefotaxime
Y	Y	Spektrum	Ceftriaxone
Y	Y	Spektrum	Azithromisin
Y	Y	Spektrum	Ampicilin
Y	Y	Spektrum	Cefotaxime
Y	Y	Spektrum	Ceftriaxone
Y	Y	Spektrum	Ceftriaxone
Y	Y	Spektrum	Ceftriaxone
Y	Y	Spektrum	Cefazolin
Y	Y	Spektrum	Ceftriaxone
Y	Y	Spektrum	Ceftriaxone
Y	Y	Spektrum	Cefotaxime
Y	Y	Spektrum	Cefotaxime

Y	Y	Spektrum	Ciprofloxacin
Y	Y	Spektrum	Azitromisin
Y	Y	Spektrum	Ampicilin
Y	Y	Spektrum	Ceftriaxone
Y	Y	Spektrum	Cefotaxime
Y	Y	Spektrum	Ceftazidime
Y	Y	Spektrum	Cefixime
Y	Y	Spektrum	Ceftriaxone
Y	Y	Spektrum	Amoxicilin
Y	Y	Spektrum	Ceftriaxone
Y	Y	Spektrum	Cefotaxime
Y	Y	Spektrum	Ciprofloxacin
Y	Y	Spektrum	Ampicilin
Y	Y	Spektrum	Cefotaxime
Y	Y	Spektrum	Cefotaxime
Y	Y	Spektrum	Cefotaxime
Y	Y	Spektrum	Cefazoline
Y	Y	Spektrum	Cefotaxime
Y	Y	Spektrum	Meropenem
Y	Y	Spektrum	Meropenem
Y	Y	Spektrum	Ceftriaxone
Y	Y	Spektrum	Cefotaxime
Y	Y	Spektrum	Cefotaxime
Y	Y	Spektrum	Cefotaxime
Y	Y	Spektrum	Cefotaxime
Y	Y	Spektrum	Cefotaxime
Y	Y	Spektrum	Ceftriaxone
Y	Y	Spektrum	Ceftriaxone
Y	Y	Spektrum	Cefotaxime
Y	Y	Spektrum	Cefotaxime
Y	Y	Spektrum	Cefotaxime
Y	Y	Spektrum	Cefotaxime
Y	Y	Spektrum	Cefotaxime
Y	Y	Spektrum	Cefotaxime
Y	Y	Spektrum	Cefotaxime
Y	Y	Spektrum	Cefotaxime
Y	Y	Spektrum	Metronidazole
Y	Y	Spektrum	Cefotaxime
Y	Y	Spektrum	Cefotaxime
Y	Y	Spektrum	Cefotaxime
Y	Y	Spektrum	Cefotaxime
Y	Y	Spektrum	Cefotaxime
Y	Y	Spektrum	Ceftriaxone
Y	Y	Spektrum	Metronidazole
Y	Y	Spektrum	Cefotaxime
Y	Y	Spektrum	Cefotaxime

Y	Y	Spektrum	Ceftriaxone
Y	Y	Spektrum	Meropenem
Y	Y	Spektrum	Ampicilin Sulbactam
Y	Y	Spektrum	Ampicilin Sulbactam
Y	Y	Spektrum	Meropenem
Y	Y	Spektrum	Ceftriaxone
Y	Y	Spektrum	Cefotaxime
Y	Y	Spektrum	Cefotaxime
Y	Y	Spektrum	Cefotaxime
Y	Y	Spektrum	Cefotaxime
Y	Y	Spektrum	Cefotaxime
Y	Y	Spektrum	Ceftriaxone
Y	Y	Spektrum	Cefotaxime
Y	Y	Spektrum	Cefotaxime
Y	Y	Spektrum	Cefotaxime
Y	Y	Spektrum	Ceftriaxone
Y	Y	Spektrum	Meropenem
Y	Y	Spektrum	Meropenem
Y	Y	Spektrum	Cefotaxime
Y	Y	Spektrum	Metronidazole
Y	Y	Spektrum	Cefotaxime
Y	Y	Spektrum	Ceftriaxone
Y	Y	Spektrum	Cefotaxime
Y	Y	Spektrum	Ceftriaxone
Y	Y	Spektrum	Cefotaxime
Y	Y	Spektrum	Cloxacilin
Y	Y	Spektrum	Cefotaxime
Y	Y	Spektrum	Ceftazidime
Y	Y	Spektrum	Ceftriaxone
Y	Y	Spektrum	Cefotaxime
Y	Y	Spektrum	Ceftriaxone
Y	Y	Spektrum	Cefotaxime
Y	Y	Spektrum	Ceftriaxone
Y	Y	Spektrum	Ceftriaxone
Y	Y	Spektrum	Azithromisin
Y	Y	Spektrum	Cefadroxil
Y	Y	Spektrum	Ceftriaxone
N	Y	Spektrum	Ceftazidime
Antibiotic	Irrationality	Type Irrationali	Type of first antibiotics 1
Y	N		Ampicilin
Y	N		OAT FOC
Y	N		Ampicilin
Y	N		Ampicilin
Y	N		Ceftriaxone
Y	N		Ceftriaxone
Y	N		Ampicilin

Y	N	Cefotaxime
Y	N	Ampicilin
Y	N	Cefotaxime
Y	N	Ampicilin
Y	N	Ampicilin
Y	N	Ampicilin
Y	N	Ampicilin
Y	N	Ceftriaxone
Y	N	Cefotaxime
Y	N	Ampicilin
Y	N	Ampicilin
Y	N	Cefotaxime
Y	N	Ampicilin
Y	N	Cefotaxime
Y	N	Amoxicilin
Y	N	Ampicilin
Y	N	Cefotaxime
Y	N	Ampicilin
Y	N	Chlorampenicol
Y	N	Ampicilin
Y	N	Ampicilin
Y	N	Amoxicilin
Y	N	Ampicilin
Y	N	Ampicilin
Y	N	Ampicilin
Y	N	Ceftriaxone
Y	N	Cefotaxime
Y	N	Ampicilin
Y	N	Ampicilin
Y	N	Ampicilin
Y	N	Ampicilin
Y	N	Cefotaxime
Y	N	Gentamisin
Y	N	Ampicilin
Y	N	Ampicilin
Y	N	Ampicilin
Y	N	Ampicilin
Y	N	Ampicilin
Y	N	
Y	N	Ampicilin
Y	N	Ampicilin
Y	N	Ampicilin
Y	N	Ampicilin
Y	N	Ampicilin
Y	N	
Y	N	Ampicilin

Y	N	Cefotaxime
Y	N	Cefotaxime
Y	N	Penoxymethyl Penicilin
Y	N	Ampicilin
Y	N	Cefotaxime
Y	N	Ampicilin
Y	N	Eritromisin
Y	N	Ampicilin
Y	N	Ampicilin
Y	N	Ampicilin
Y	N	Ampicilin
Y	N	Ampicilin
Y	N	Ampicilin
Y	N	Ampicilin
Y	N	Ospen
Y	N	Ampicilin
Y	N	Ampicilin
Y	N	Ampicilin
Y	N	Cefotaxime
Y	N	Cefotaxime
Y	N	Ampicilin
Y	N	Ampicillin
Y	N	Ampicilin
Y	N	Cefotaxime
Y	Y	Cefotaxime
Y	Y	Cefotaxime
Y	N	Ceftriaxone
Y	N	
Y	N	Cefotaxime
Y	N	Ampicilin
Y	N	Ampicilin
Y	N	Ampicilin
Y	N	INH
Y	N	Ampicilin
Y	N	Ampicilin
Y	N	Cotrimoxasole
Y	N	Ampicilin
Y	N	Cefotaxime
Y	N	Ampicilin
Y	N	Ampicilin
Y	N	Ampicilin
Y	N	Ampicilin
Y	N	Ampicilin
Y	N	Ampicilin
Y	N	Ampicilin
Y	N	OAT

Y	N	Ampicilin
Y	N	Ampicilin
Y	N	Ampicilin
Y	N	Ampicilin
Y	N	Ampicilin
Y	N	Ampicilin
Y	N	Ampicilin
Y	N	Ceftriaxone
Y	N	Cefotaxime
Y	N	Cefotaxime
Y	N	Ampicilin
Y	N	Ampicilin
Y	N	Amoxicilin
Y	N	Ampicilin
Y	N	Amoxicilin
Y	N	Ampicilin
Y	N	Ampicilin
Y	N	Ampicilin
Y	N	Ampicilin
Y	N	Ampicilin
Y	N	Ampicilin
Y	N	Ampicilin
Y	N	Ampicilin
Y	N	Ampicilin
Y	N	Cefotaxime
Y	N	Ampicilin
Y	N	Ampicilin
Y	N	Ampicilin
Y	N	Ampicilin
Y	N	Ampicilin
Y	N	Ampicilin
Y	N	Ampicilin
Y	N	Ampicilin
Y	N	Ampicilin
Y	N	Ampicilin
Y	N	Ceftriaxone
Y	N	Ceftriaxone
Y	N	Ampicilin
Y	N	Ampicilin
Y	N	Ampicilin
Y	N	Ampicilin
Y	N	Ceftriaxone
Y	N	Ampicilin
Y	N	Ampicilin
Y	N	Ampicilin
Y	N	Ampicilin

Y	N	Ampicilin
Y	N	Ampicilin
Y	N	Ampicilin
Y	N	Ampicilin
Y	N	Cotrimoxasole
Y	N	Ampicilin
Y	N	Ampicilin
Y	N	Ampicilin
Y	N	Ampicilin
Y	N	Ampicilin
Y	N	Ampicilin
Y	N	Cefotaxime
Y	N	Ampicilin
Y	N	INH
Y	N	Ampicilin
Y	N	Cotrimoxasole
Y	N	Ampicilin
Y	N	Ampicilin
Y	N	Ampicilin
Y	N	Ampicilin
Y	N	Ampicilin
Y	N	Ampicilin
Y	N	Ampicilin
Y	N	Ampicilin
Y	N	Ampicilin
Y	N	Ceftazidime
Y	N	Cefotaxime
Y	N	Ampicilin
Y	N	Ampicilin
Y	N	Ampicilin
Y	N	Cefotaxime
Y	N	Ampicilin
Y	N	Ampicilin
Y	N	Ampicilin
Y	N	Ampicilin
Y	N	Ampicilin
Y	N	Gentamisin
Y	N	Ampicilin
Y	N	Ampicilin
y	N	Ampicilin
Y	N	Ampicilin
Y	N	Phenoxy penicilin
Y	N	Ampicilin
Y	N	Ceftriaxone
Y	N	Ampicilin
Y	N	Ampicilin

Y	N	Ampicilin
Y	N	Ampicilin
Y	N	Ampicilin
Y	N	Ampicilin
Y	N	Ampicilin
Y	N	Ampicilin
Y	N	Ampicilin
Y	N	Ampicilin
Y	N	Ampicilin
Y	N	Ampicilin
Y	N	Cefotaxime
Y	N	Metronidazole
Y	N	Ampicilin
Y	N	Ampicilin
Y	N	Ampicilin
Y	N	Cefixime
Y	N	Ampicilin
Y	N	Ampicilin
Y	N	Ampicilin
Y	N	Ampicilin
Y	N	Ampicilin
Y	N	Ampicilin
Y	N	Ampicilin
Y	N	Ampicilin
Y	N	Ampicilin
Y	N	Ceftriaxone
Y	N	
Y	N	Ceftriaxone
Y	N	Cefixime
Y	N	Ampicilin
Y	N	
Y	N	Ampicilin
Y	N	Ampicilin
Y	N	Ampicilin
Y	N	Ceftriaxone
Y	N	Amoxicilin
Y	N	Ampicilin
Y	N	Amoxicilin
Y	N	Ampicilin
Y	N	Ampicilin
Y	N	Ampicilin
Y	N	Cefotaxime
Y	N	Ampicilin
Y	N	Ampicilin
Y	N	Cotrimoxasole
Y	N	Cefixime

Y	N	Cefotaxime
Y	N	Cefixime
Y	N	Fluconazole
Y	N	Ampicilin
Y	N	Ampicilin
Y	N	Ampicilin
Y	N	Cefixime
Y	N	Ampicilin
Y	N	Cotrimoxasole
Y	N	Ampicilin
Y	N	Ampicilin
Y	N	Ampicilin
Y	N	Cefotaxime
Y	N	Ampicilin
Y	N	Ampicilin
Y	N	Cefixime
Y	N	Cotrimoxasole
Y	N	Ampicilin
Y	N	Ampicilin
Y	N	Cefotaxime
Y	N	Ampicilin
Y	N	Cefixime
Y	N	Eritromisin
Y	N	Cotrimoxasole
Y	N	Cefotaxime
Y	N	Ampicilin
Y	N	Ampicilin
Y	N	Ampicilin
Y	N	Ampicilin
Y	N	Ampicilin
Y	N	Ampicilin
Y	N	Ampicilin
Y	N	Ampicilin
Y	N	Ampicilin
Y	N	Amoxycilin
Y	N	Ampicilin
Y	N	Ampicilin
Y	N	Ampicilin
Y	N	Ampicilin
Y	N	Ampicilin
Y	N	Ampicilin
Y	N	Ampicilin
Y	N	Ampicilin
Y	N	Ampicilin
Y	N	Ampicilin
Y	N	Ampicilin

Y	N	Cotrimoxasole
Y	N	Ceftriaxone
Y	N	Ampicilin
Y	N	Ampicilin
Y	N	Ampicilin
Y	N	Ampicilin
Y	N	Ampicilin
Y	N	Ampicilin
Y	N	Cefotaxime
Y	N	Ampicilin
Y	N	Ampicilin
Y	N	Cotrimoxasole
Y	N	Ampicilin
Y	N	Ospen
Y	N	Ampicilin
Y	N	Ampicilin
Y	N	Cotrimoxasole
Y	N	Ampicilin
Y	N	Ampicilin
Y	N	Ampicilin
Y	N	Ampicilin
Y	N	Amoxicilin
Y	N	Cefotaxime
Y	N	Ampicilin
Y	N	Ampicilin
Y	N	Ospen
Y	N	Ampicilin
Y	N	Cefixime
Y	N	Ampicilin
Y	N	Ampicilin
Y	N	Ampicilin
Y	N	Ceftriaxone
Y	N	Ampicilin
Y	N	Ampicilin
Y	N	Ceftriaxone
Y	N	Cefixime
Y	N	Ampicilinj
Y	N	Ampicilin
Y	N	Ampicilin
Y	N	Fluconazole
Y	N	Ampicilin
Y	N	Ampicilin
Y	N	Cefotaxime
Y	N	Ampicilin
Y	N	Ospen
Y	N	Cefotaxime

Y	N		Cefotaxime
Y	N		Ampicilin
Y	N		Cefotaxime
Y	N		Clindamisin
Y	N		Cefotaxime
Y	N		Ampicilin
Y	N		
Y	N		Ampicilin
Y	N		Ampicilin
Y	N		Ampicilin
Y	N		Ampicilin
Y	N		Ceftriaxone
Y	N		Ampicilin
Y	N		Ampicilin
Y	N		Ampicilin
Y	N		Ampicilin
Y	N		Ampicilin
Y	N		Ampicilin
Y	N		Ampicilin
Y	N		Ampicilin
Y	N		Ampicilin
Y	N		Ampicilin
Y	N		Ampicilin
Y	N		Ampicilin
Y	N		Ampicilin
Y	N		Cefotaxime
Y	N		Ampicilin
Y	N		Ceftriaxone
Y	N		Cefotaxime
Y	N		Ampicilin
Y	N		Ampicilin
Y	N		Ampicilin
Y	N		Ampicilin
Y	N		Ampicilin
Y	N		Ampicilin
Y	N		Ampicilin
Y	N		Ampicilin
Y	N		Ampicilin
Y	Y		Ampicilin
Y	N		Ospen
Y	N		Ampicilin
Y	N		Ampicilin
Y	N		Amoxicilin
Y	N		Cotrimoxasole
Y	N		Ampicilin
Y	N		Ampicilin

Y	N	Ampicilin
Y	N	Ampicilin
Y	N	Ampicilin
Y	N	Gentamisin
Y	N	Ampicilin
Y	N	Ampicilin
Y	N	Ospen
Y	N	Cefotaxime
Y	N	Cefotaxime
Y	N	Ampicilin
Y	N	Ampicilin
Y	N	Ampicilin
Y	N	Ampicilin
Y	N	Ampicilin
Y	N	Ceftriaxone
Y	N	Ampicilin
Y	N	Ampicilin
Y	N	Ampicilin
Y	N	Ampicilin
Y	N	Cotrimoxsasole
Y	N	Ampicilin
Y	N	Ampicilin
Y	N	Ampicilin
Y	N	Cefotaxime
Y	N	Ampicilin
Y	N	Ampicilin
Y	N	Ampicilin
Y	N	Ampicilin
Y	N	Ampicilin
Y	N	Cefotaxime
Y	N	Cefixime
Y	N	Ampicilin
Y	N	Ampicilin
Y	N	Ampicilin
Y	N	Ampicilin
Y	N	Ampicilin
Y	N	Ampicilin
Y	N	Ampicilin
Y	N	Cefotaxime
Y	N	Ampicilin
Y	N	Ampicilin
Y	N	Ampicilin
Y	N	Ampicilin
Y	N	Ampicilin
Y	N	Ampicilin
Y	N	Ampicilin

Y	N	Ampicilin
Y	N	Cotrimoxasole
Y	N	Ampicilin
Y	N	Ampicilin
Y	N	Ampicilin
Y	N	Ampicilin
Y	N	Ampicilin
Y	N	Ampicilin
Y	N	Ampicilin
Y	N	Ampicilin
Y	N	Ampicilin
Y	N	Ampicilin
Y	N	Ampicilin
Y	N	Ampicilin
Y	N	Ospen
Y	N	Cefixime
Y	N	Ampicilin
Y	N	Ampicilin
Y	N	Ampicilin
Y	N	Ampicilin
Y	N	Ampicilin
Y	N	Ampicilin
Y	N	Ampicilin
Y	N	Ampicilin
Y	N	Ampicilin
Y	N	Ampicilin
Y	N	Ampicilin
Y	N	Ampicilin
Y	N	Ampicilin
y	N	Ampicilin
Y	N	Ampicilin
Y	N	Ampicilin
Y	N	Ampicilin
Y	N	Ampicilin
Y	N	Ampicilin
Y	N	Ceftriaxone
Y	N	Ceftriaxone
Y	N	Ampicilin
Y	N	Ampicilin
Y	N	Ampicilin
Y	N	Ampicilin
Y	N	Ampicilin
Y	N	Gentamisin
Y	N	Ampicilin
Y	N	Ampicilin
Y	N	Ampicilin

Y	N	Ampicilin
Y	N	Ampicilin
Y	N	Ampicilin
Y	N	Ampicilin
Y	N	Ampicilin
Y	N	Ampicilin
Y	N	Ampicilin
Y	N	Ampicilin
Y	N	Cefotaxime
Y	N	Ampicilin
Y	N	Ampicilin
Y	N	Ampicilin
Y	N	Ampicilin
Y	N	Ampicilin
Y	N	Cefotaxime
Y	N	Ampicilin
Y	N	Ampicilin
Y	N	Ampicilin
Y	N	Ampicilin
Y	N	Ampicilin
Y	N	Gentamisin
Y	N	Ceftriaxone
Y	N	Ampicilin
Y	N	Ampicilin
Y	N	Ampicilin
Y	N	Ampicilin
Y	N	Ampicilin
Y	N	Eritromisin
Y	N	Ampicilin
Y	N	Ampicilin
Y	N	Ampicilin
Y	N	Ampicilin
Y	N	Ampicilin
Y	N	Ampicilin
Y	N	Ampicilin
Y	N	Ampicilin
Y	N	Ampicilin
Y	N	Ampicilin
Y	N	Cefotaxime
Y	N	Ampicilin
Y	N	Ampicilin
Y	N	Ampicilin
Y	N	Ampicilin
Y	N	Ampicilin
Y	N	Ampicilin
Y	N	Ampicilin

Y	N	Cefazolin
Y	N	Cefazolin
Y	N	Cefazolin
Y	N	Ospen
Y	N	Ampicilin
Y	N	Ceftriaxone
Y	N	Ampicilin
Y	N	Ampicilin
Y	N	Cefazoline
Y	N	Cefazoline
Y	N	Ampicilin
Y	N	Cefazoline
Y	N	Ampicilin
Y	N	Ampicilin
Y	N	Cefazoline
Y	N	Cefazoline
Y	N	Cefazoline
Y	N	Cefazoline
Y	N	Cefazoline
Y	N	Ampicilin
Y	N	Cefotaxime
Y	N	Ceftriaxone
Y	N	Ceftriaxone
Y	N	Ampicilin
Y	N	Cefotaxime
Y	N	Ampicilin
Y	N	Ampicillin
Y	N	Ampicilin
Y	N	Ampicillin
Y	N	Ampicillin
Y	N	Ampicillin
Y	N	Ampicillin
Y	N	Ampicillin
Y	N	Ampicillin
Y	N	Cefazolin
Y	N	Ampicillin
Y	N	Cefazolin
Y	N	Cefazolin
Y	N	Cefazolin
Y	N	Cefotaxime
Y	N	Cefotaxime
Y	N	Ampicilin
Y	N	Ceftriaxone
Y	N	Ampicilin
Y	N	Ospen
Y	N	Ospen

Y	N	Cefazolin
Y	N	Cefazolin
Y	N	Ospen
Y	N	Cefazolin
Y	N	Ospen
Y	N	Cefazolin
Y	N	Cefazolin
Y	N	Cefazolin
Y	N	Cefazolin
Y	N	Cefazolin
Y	N	Cefazolin
Y	N	Cefazolin
Y	N	Clindamisin
Y	N	Cefazolin
Y	N	Cefixime
Y	N	Ampicilin
Y	N	Ceftriaxone
Y	N	Cefazoline
Y	N	Cefazoline
Y	N	Cefazoline
N	N	
N	N	
N	N	
N	N	
N	N	
N	N	
N	N	
N	N	
N	N	
N	N	
N	N	
N	N	
N	N	
N	N	
N	N	
N	N	
N	N	
N	N	
N	N	
N	N	
N	N	
N	N	
N	N	
N	N	
N	N	
N	N	

N	N
N	N
N	N
N	N
N	N
N	N
N	N
N	N
N	N
N	N
N	N
N	N
N	N
N	N
N	N
N	N
N	N
N	N
N	N
N	N
N	N
N	N
N	N
N	N
N	N
N	N
N	N
N	N
N	N
N	N
N	N
N	N
N	N
N	N
N	N
N	N
N	N
N	N
N	N
N	N
N	N
N	N
N	N
N	N
N	N
N	N

N	N
N	N
N	N
N	N
N	N
N	N
N	N
N	N
N	N
N	N
N	N
N	N
N	N
N	N
N	N
N	N
N	N
N	N
N	N
N	N
N	N
N	N
N	N
N	N
N	N
N	N
N	N
N	N
N	N
N	N
N	N
N	N
N	N
N	N
N	N
N	N
N	N
N	N
N	N
N	N
N	N
N	N
N	N
N	N
N	N
N	N

N	N
N	N
N	N
N	N
N	N
N	N
N	N
N	N
N	N
N	N
N	N
N	N
N	N
N	N
N	N
N	N
N	N
N	N
N	N
N	N
N	N
N	N
N	N
N	N
N	N
N	N
N	N
N	N
N	N
N	N
N	N
N	N
N	N
N	N
N	N
N	N
N	N
N	N
N	N
N	N
N	N
N	N
N	N
N	N
N	N
N	N

N	N
N	N
N	N
N	N
N	N
N	N
N	N
N	N
N	N
N	N
N	N
N	N
N	N
N	N
N	N
N	N
N	N
N	N
N	N
N	N
N	N
N	N
N	N
N	N
N	N
N	N
N	N
N	N
N	N
N	N
N	N
N	N
N	N
N	N
N	N
N	N
N	N
N	N
N	N
N	N
N	N
N	N
N	N
N	N
N	N
N	N

N	N
N	N
N	N
N	N
N	N
N	N
N	N
N	N
N	N
N	N
N	N
N	N
N	N
N	N
N	N
N	N
N	N
N	N
N	N
N	N
N	N
N	N
N	N
N	N
N	N
N	N
N	N
N	N
N	N
N	N
N	N
N	N
N	N
N	N
N	N
N	N
N	N
N	N
N	N
N	N
N	N
N	N
N	N
N	N
N	N
N	N

N	N
N	N
N	N
N	N
N	N
N	N
N	N
N	N
N	N
N	N
N	N
N	N
N	N
N	N
N	N
N	N
N	N
N	N
N	N
N	N
N	N
N	N
N	N
N	N
N	N
N	N
N	N
N	N
N	N
N	N
N	N
N	N
N	N
N	N
N	N
N	N
N	N
N	N
N	N
N	N
N	N
N	N
N	N
N	N
N	N
N	N

N	N
N	N
N	N
N	N
N	N
N	N
N	N
N	N
N	N
N	N
N	N
N	N
N	N
N	N
N	N
N	N
N	N
N	N
N	N
N	N
N	N
N	N
N	N
N	N
N	N
N	N
N	N
N	N
N	N
N	N
N	N
N	N
N	N
N	N
N	N
N	N
N	N
N	N
N	N
N	N
N	N
N	N
N	N
N	N
N	N
N	N

N	N
N	N
N	N
N	N
N	N
N	N
N	N
N	N
N	N
N	N
N	N
N	N
N	N
N	N
N	N
N	N
N	N
N	N
N	N
N	N
N	N
N	N
N	N
N	N
N	N
N	N
N	N
N	N
N	N
N	N
N	N
N	N
N	N
N	N
N	N
N	N
N	N
N	N
N	N
N	N
N	N
N	N
N	N
N	N
N	N
N	N

N	N
N	N
N	N
N	N
N	N
N	N
N	N
N	N
N	N
N	N
N	N
N	N
N	N
N	N
N	N
N	N
N	N
N	N
N	N
N	N
N	N
N	N
N	N
N	N
N	N
N	N
N	N
N	N
N	N
N	N
N	N
N	N
N	N
N	N
N	N
N	N
N	N
N	N
N	N
N	N
N	N
N	N
N	N
N	N
N	N
N	N

N	N
N	N
N	N
N	N
N	N
N	N
N	N
N	N
N	N
N	N
N	N
N	N
N	N
N	N
N	N
N	N
N	N
N	N
N	N
N	N
N	N
N	N
N	N
N	N
N	N
N	N
N	N
N	N
N	N
N	N
N	N
N	N
N	N
N	N
N	N
N	N
N	N
N	N
N	N
N	N
N	N
N	N
N	N
N	N
N	N
N	N

N	N
N	N
N	N
N	N
N	N
N	N
N	N
N	N
N	N
N	N
N	N
N	N
N	N
N	N
N	N
N	N
N	N
N	N
N	N
N	N
N	N
N	N
N	N
N	N
N	N
N	N
N	N
N	N
N	N
N	N
N	N
N	N
N	N
N	N
N	N
N	N
N	N
N	N
N	N
N	N
N	N
N	N
N	N
N	N
N	N
N	N

N	N
N	N
N	N
N	N
N	N
N	N
N	N
N	N
N	N
N	N
N	N
N	N
N	N
N	N
N	N
N	N
N	N
N	N
N	N
N	N
N	N
N	N
N	N
N	N
N	N
N	N
N	N
N	N
N	N
N	N
N	N
N	N
N	N
N	N
N	N
N	N
N	N
N	N
N	N
N	N
N	N
N	N
N	N
N	N
N	N
N	N

N	N
N	N
N	N
N	N
N	N
N	N
N	N
N	N
N	N
N	N
N	N
N	N
N	N
N	N
N	N
N	N
N	N
N	N
N	N
N	N
N	N
N	N
N	N
N	N
N	N
N	N
N	N
N	N
N	N
N	N
N	N
N	N
N	N
N	N
N	N
N	N
N	N
N	N
N	N
N	N
N	N
N	N
N	N
N	N
N	N
N	N

N	N
N	N
N	N
N	N
N	N
N	N
N	N
N	N
N	N
N	N
N	N
N	N
N	N
N	N
N	N
N	N
N	N
N	N
N	N
N	N
N	N
N	N
N	N
N	N
N	N
N	N
N	N
N	N
N	N
N	N
N	N
N	N
N	N
N	N
N	N
N	N
N	N
N	N
N	N
N	N
N	N
N	N
N	N
N	N
N	N
N	N

N	N
N	N
N	N
N	N
N	N
N	N
N	N
N	N
N	N
N	N
N	N
N	N
N	N
N	N
N	N
N	N
N	N
N	N
N	N
N	N
N	N
N	N
N	N
N	N
N	N
N	N
N	N
N	N
N	N
N	N
N	N
N	N
N	N
N	N
N	N
N	N
N	N
N	N
N	N
N	N
N	N
N	N
N	N
N	N
N	N
N	N

N	N
N	N
N	N
N	N
N	N
N	N
N	N
N	N
N	N
N	N
N	N
N	N
N	N
N	N
N	N
N	N

Antibiotics
