# Supplementary material for: Multifaceted interventions for healthcare-associated infections and rational use of antibiotics in a low-to-middle-income country: Can they be sustained?
Source: PLoS One. 2020 Jun 16;15(6):e0234233. doi: 10.1371/journal.pone.0234233 (PMC7297356; doi:10.1371/journal.pone.0234233)
Supplement: Attachment — Submitted filename: Response to Reviewer .docx [file pone.0234233.s004.docx]

Dear Reviewers and Editor,

We have revised the manuscript accordingly as suggested. We have addressed the following additional comments:

1. Under Methods section A. Hospital-acquired infection, the authors state that “The definitions of HAI were based on the US Centers for Disease Control and Prevention (CDC) National Healthcare Safety Network (NHSN). Every child in the study was observed each day to see whether he/she fulfilled the CDC criteria for a HAI. If criteria were fulfilled, the treating medical and nursing staff were advised to collect a culture of blood, urine or other sterile sites as appropriate on the same day.”

Please elaborate on this process – how many/what proportion of culture collection was guided by the study surveillance vs. based on clinical suspicion of infection? Could the variability in this process explain any of the observed increase in HAIs during the maintenance phase?

Response to the Reviewer:

We did not have data detailing which cultures were collected based on the study surveillance definition or based on clinical suspicion of infection of the treating doctors. We included those two as one.

But we did an identical data collection to diagnose nosocomial infections in both periods. Those cultures were taken when patients had signs and symptoms of infection. Further, there were no changes in laboratory procedures between the post intervention and maintenance periods that might falsify the culture results. These relatively independent factors suggest that the identification of nosocomial infections between two periods was similar.

2. The authors have clarified that regression analysis was not used and there was no accounting for changes in case-mix over time. I would include in the discussion as a limitation of an analysis that it does not account for potential changes in patient characteristics over time. Please also consider a brief statement on whether any major changes in patient characteristics occurred to your knowledge e.g., change in immunocompromised host population, types of surgery etc.

Response to the Reviewer:

We decided to perform multivariable logistic regression analysis to quantify the relationship between the HAI and the multifaceted intervention allowing for statistical control of potential confounders including age, sex, the presence of syndrome, immunocompromised condition, referral patient, sepsis, and malnutrition. We add this Table 4.

Table 4. Multivariable analysis of the factors affecting intervention on HAI

| Multifaceted evaluation | OR (95% CI) | p value |
| --- | --- | --- |
| Crude OR | 2.15 (1.72-2.68) | <0.001 |
| Adjusted for age | 2.16 (1.73-2.69) | <0.001 |
| Adjusted for gender | 2.15 (1.73-2.69) | <0.001 |
| Adjusted for syndrome | 2.24 (1.79-2.80) | <0.001 |
| Adjusted for immunocompromised | 2.08 (1.66-2.61) | <0.001 |
| Adjusted for referral | 1.94 (1.55-2.42) | <0.001 |
| Adjusted for sepsis | 1.97 (1.58-2.47) | <0.001 |
| Adjusted for malnutrition | 2.18 (1.75-2.73) | <0.001 |
| Adjusted for all above | 1.94 (1.53-2.45) | <0.001 |

3. Please proof-read to ensure that HAI is now spelled out as healthcare-associated infection throughout (some instances of use of hospital-acquired were noted)

Response to the Reviewer: We have revised accordingly.

4. Change header stating “The use of irrational antibiotics” to “The irrational use of antibiotics”

Response to the Reviewer: We have revised accordingly.
