# Supplementary material for: Multifaceted interventions for healthcare-associated infections and rational use of antibiotics in a low-to-middle-income country: Can they be sustained?
Source: PLoS One. 2020 Jun 16;15(6):e0234233. doi: 10.1371/journal.pone.0234233 (PMC7297356; doi:10.1371/journal.pone.0234233)
Supplement: Attachment — Submitted filename: Response to Reviewer .docx [file pone.0234233.s005.docx]

Dear Reviewers and Editor,

We have revised the manuscript accordingly as suggested. We have addressed the following additional comments:

1. Thank you for clarifying the process of culture collection. Please include this information and explanation in the methods section.

Response to the Reviewer:

Yes, thank you. We have included this information in the Methods section.

We did not have data detailing which cultures were collected based on the study surveillance definition or based on clinical suspicion of infection of the treating doctors. We included those two as one. But we did an identical data collection to diagnose nosocomial infections in both periods. Those cultures were taken when patients had signs and symptoms of infection.

2. Thank you for adding the multivariable regression analysis. Please also do the following: a) ensure that variable names in the model are the same as the variable names in Table 2. b) Table 4 is not clear, and  likely not needed. It would be better to describe the model building process in the text itself - were the variables added stepwise, what were the criteria for including the variables in the model etc. c) The statement "We did a multivariable analysis to adjust for differences in patient characteristics." is not necessary in the Results section, it is already explained in the Methods.

Response to the Reviewer:

a. We have revised the variable name in the Table 2 is the same as the variable in the model.
b. We have deleted Table 4 and described the model in the text.
c. Yes, we have deleted and revised accordingly.

3) In the discussion, when discussing major findings, I would state that there were no major differences in the patient population in the two time periods, and even after adjusting for differences, there was an increase in HAIs. Similarly, I would expand on the statement "Staff turnover and health care shortages create problems
because new doctors and nurses were not aware of infection control bundle especially in PICU." Do you think this was the major reason for the increase, please state this more clearly and expand with a couple of additional statements

Response to the Reviewer:

Thank you, we have added the statement as suggested.

Staff turnover and health care shortages create problems because new doctors and nurses were not aware of infection control bundle especially in PICU. Increasing nurse-to-patient ratios have been associated with the spread of infection. Further, nurse shortages are also related to increased risk of healthcare-associated infections.
